# Comparative safety and effectiveness of serotonin receptor antagonists in patients undergoing chemotherapy: a systematic review and network meta-analysis

**DOI:** 10.1186/s12916-016-0761-9

**Published:** 2016-12-23

**Authors:** Andrea C. Tricco, Erik Blondal, Areti Angeliki Veroniki, Charlene Soobiah, Afshin Vafaei, John Ivory, Lisa Strifler, Roberta Cardoso, Emily Reynen, Vera Nincic, Huda Ashoor, Joanne Ho, Carmen Ng, Christy Johnson, Erin Lillie, Jesmin Antony, Derek J. Roberts, Brenda R. Hemmelgarn, Sharon E. Straus

**Affiliations:** 1 Knowledge Translation Program, Li Ka Shing Knowledge Institute, St. Michael’s Hospital, 209 Victoria Street, East Building, Room 716, Toronto, Ontario M5B 1W8 Canada; 2 Epidemiology Division, Dalla Lana School of Public Health, University of Toronto, 6th Floor, 155 College Street, Toronto, Ontario M5T 3M7 Canada; 3 Institute for Health Policy Management & Evaluation, University of Toronto, 4th Floor, 155 College Street, Toronto, Ontario M5T 3M6 Canada; 4 Departments of Medicine and Community Health Sciences, University of Calgary, TRW Building, 3rd Floor, 3280 Hospital Drive NW, Calgary, Alberta T2N 4Z6 Canada; 5 Department of Geriatric Medicine, University of Toronto, 27 King’s College Circle, Toronto, Ontario M5S 1A1 Canada

**Keywords:** Chemotherapy, Systematic review, Network meta-analysis, Serotonin receptor antagonists, Effectiveness, Safety

## Abstract

**Background:**

Although serotonin (5-HT_3_) receptor antagonists are effective in reducing nausea and vomiting, they may be associated with increased cardiac risk. Our objective was to examine the comparative safety and effectiveness of 5-HT_3_ receptor antagonists (e.g., dolasetron, granisetron, ondansetron, palonosetron, tropisetron) alone or combined with steroids for patients undergoing chemotherapy.

**Methods:**

We searched MEDLINE, Embase, and the Cochrane Central Register of Controlled Trials from inception until December 2015 for studies comparing 5-HT_3_ receptor antagonists with each other or placebo in chemotherapy patients. The search results were screened, data were abstracted, and risk of bias was appraised by pairs of reviewers, independently. Random-effects meta-analyses and network meta-analyses (NMAs) were conducted.

**Results:**

After screening 9226 citations and 970 full-text articles, we included 299 studies (*n* = 58,412 patients). None of the included studies reported harms for active treatment versus placebo. For NMAs on the risk of arrhythmia (primary outcome; three randomized controlled trials [RCTs], 627 adults) and mortality (secondary outcome; eight RCTs, 4823 adults), no statistically significant differences were observed between agents. A NMA on the risk of QTc prolongation showed a significantly greater risk for dolasetron + dexamethasone versus ondansetron + dexamethasone (four RCTs, 3358 children and adults, odds ratio 2.94, 95% confidence interval 2.13–4.17).

For NMAs on the number of patients without nausea (44 RCTs, 11,664 adults, 12 treatments), number of patients without vomiting (63 RCTs, 15,460 adults, 12 treatments), and number of patients without chemotherapy-induced nausea or vomiting (27 RCTs, 10,924 adults, nine treatments), all agents were significantly superior to placebo. For a NMA on severe vomiting (10 RCTs, 917 adults), all treatments decreased the risk, but only ondansetron and ramosetron were significantly superior to placebo. According to a rank-heat plot with the surface under the cumulative ranking curve results, palonosetron + steroid was ranked the safest and most effective agent overall.

**Conclusions:**

Most 5-HT_3_ receptor antagonists were relatively safe when compared with each other, yet none of the studies compared active treatment with placebo for harms. However, dolasetron + dexamethasone may prolong the QTc compared to ondansetron + dexamethasone. All agents were effective for reducing risk of nausea, vomiting, and chemotherapy-induced nausea or vomiting.

**Trial registration:**

This study was registered at PROSPERO: (CRD42013003564).

**Electronic supplementary material:**

The online version of this article (doi:10.1186/s12916-016-0761-9) contains supplementary material, which is available to authorized users.

## Background

Cancer is the leading cause of death worldwide, accounting for 8.2 million deaths in 2012. Chemotherapy is a major component of cancer therapy. However chemotherapy-induced nausea and vomiting (CINV) are common, affecting approximately 70–80% of patients who receive chemotherapy, and can be debilitating [1]. CINV cause significant anxiety [2, 3]; decrease quality of life [4, 5]; and can result in dehydration, electrolyte imbalance [6, 7], and hospital admission [8].

Serotonin (5-HT_3_) receptor antagonists are antiemetic medications that act by inhibiting the vagal nerves in the central nervous system and intestinal mucosa that trigger the emetic reflex [6, 9–11]. Examples of first-generation 5-HT_3_ receptor antagonists include dolasetron, granisetron, and ondansetron, while palonosetron is a second-generation receptor antagonist [12]. These treatments can be administered orally, subcutaneously, or intravenously. Previous systematic reviews have found that 5-HT_3_ receptor antagonists are effective for treating nausea and vomiting that occur after chemotherapy [13–16]. As such, 5-HT_3_ receptor antagonists are recommended as the first-line of treatment for CINV in both adults and children [9].

Although 5-HT_3_ receptor antagonists are effective in reducing nausea and vomiting, concerns have been raised that they may be associated with increased risk of arrhythmia. Some evidence suggests that they prolong the QT interval on electrocardiography [17, 18], which is associated with an increased risk of serious ventricular arrhythmias (e.g., torsades de pointes). In vitro studies have indicated that 5-HT_3_ receptor antagonists block voltage-dependent sodium channels and human ether-a-go-go-related gene potassium channels (cardiac ion channels), with the magnitude and type of electrocardiographic change depending on the particular drug. The US Food and Drug Administration [19] and Health Canada (a division of the Canadian federal government) [20] have published warnings on the safety of dolasetron but no warnings have appeared for other 5-HT_3_ receptor antagonists. Information about cardiac risks cannot be gleaned from previous systematic reviews of 5-HT_3_ receptor antagonists [13–16] because cardiac safety was not examined in those studies. This systematic review was undertaken, at the request of Canadian policy-makers from Health Canada, to determine the comparative safety and effectiveness of 5-HT_3_ receptor antagonists for patients undergoing chemotherapy.

## Methods

### Protocol

A systematic review protocol was drafted, revised, registered in the PROSPERO database (CRD42013003564), and published in a peer-reviewed, open-access journal [21]. Because the full methods have already been reported (Additional file 1), they are described only briefly below. We used the PRISMA extension to network meta-analysis (NMA) to report our results (Additional file 2) [22] and our analysis was conducted according to the International Society for Pharmacoeconomics and Outcomes Research (ISPOR) guidance [23].

### Data sources and searches

The primary information sources were MEDLINE, Embase, and the Cochrane Central Register of Controlled Trials, which were searched from inception until 11 December 2015. The full literature search for MEDLINE has been published previously [21]. To supplement the primary sources, we scanned the reference lists of included studies and relevant reviews [24–27], and searched conference abstracts and trial registries.

### Study selection, data extraction, and quality assessment

Studies involving patients of any age undergoing chemotherapy and receiving a 5-HT_3_ receptor antagonist (i.e., dolasetron, granisetron, ondansetron, palonosetron, ramosetron, tropisetron) alone or combined with steroids (dexamethasone, methylprednisolone, prednisone) were included, regardless of publication status, duration of administration of the intervention, or duration of follow-up. Studies published in languages other than English were excluded.

After a calibration exercise, pairs of reviewers independently screened the titles and abstracts of citations and the full-text articles of potentially relevant studies, and then abstracted data from included studies. Disagreements were resolved through consensus or the involvement of an arbitrator (ACT). The following types of data were abstracted from the included studies a priori: study characteristics, patient characteristics, and outcomes. The primary outcome of interest, as identified by policy-makers with Health Canada, was the number of patients experiencing arrhythmia. Secondary outcomes included the numbers of patients experiencing QTc prolongation, QRS interval prolongation, death, sudden cardiac death, delirium, no nausea, no vomiting, no CINV, and severe vomiting.

Randomized controlled trials (RCTs), non-RCTs, and controlled before–after studies were assessed for quality and risk of bias using the Cochrane Effective Practice and Organisation of Care risk-of-bias tool [28]. Cohort studies were assessed using the Newcastle–Ottawa Scale [29]. The quality of reporting of harm outcomes was appraised using the McMaster Quality Assessment Scale of Harm (McHarm) tool [30]. Potential conflicts of interest were recorded for all studies. Pairs of reviewers independently appraised methodological quality and conflicts were resolved through discussion.

### Data synthesis and analysis

Random-effects meta-analysis and NMA were conducted. Meta-analysis utilizes summary point estimates derived from all participants enrolled in a trial, allowing for reliable investigation of treatment effects. NMA allows for the comparison of multiple treatments in a comprehensive analysis and the determination of the best treatment among several competing treatments, including those that have never been compared in a head-to-head study [31].

For outcomes for which two or more studies comparing two interventions were available, we conducted a meta-analysis for each outcome. We assessed dichotomous outcomes and estimated the treatment effect for each pairwise comparison using the odds ratio (OR) and a 95% confidence interval (CI). We excluded studies that reported zero events across all treatment arms. We anticipated that treatment effects would vary according to patient and study characteristics, and therefore used a random-effects model, estimating the between-study variance with the restricted maximum likelihood method [32]. Statistical heterogeneity was quantified with the I^2^ statistic [33], whereas clinical and methodological heterogeneity was assessed subjectively by the study team.

Before embarking on the NMA for each outcome, we drew a network diagram to ensure that the included studies formed a connected network. Potential effect modifiers, specifically, age (adults and elderly versus children) and type of chemotherapy (cisplatin versus other), were identified before NMA was performed and separate network diagrams were drawn with edges colored according to the potential effect modifiers, to ensure balance across treatment comparisons [34]. Random-effects NMA was then conducted using the *network* command in Stata 13.0 [35, 36]. Predictive intervals were calculated to observe the range within which the effect estimate would lie should another study be available [37].

The primary analysis was limited to RCTs, with non-randomized studies included in an additional analysis to evaluate the robustness of the results. Subgroup analysis was conducted to determine whether the results changed according to the potential effect modifiers. We used the design-by-treatment interaction model [38, 39] to evaluate consistency over the entire network, accounting for potential disagreement both between designs (e.g., two-arm versus three-arm trials) and between direct and indirect evidence. When we identified statistically significant global inconsistency, we examined local inconsistency in each closed loop of the network using the loop-specific method [40, 41]. We checked inconsistent loops for potential data abstraction errors, as suggested by the loop-specific method; if such errors were identified, we repeated the analyses. Statistically significant inconsistency and important heterogeneity were explored with subgroup and sensitivity analyses. Similar to the pairwise meta-analysis, all NMAs were performed within a frequentist framework with a random-effects model assuming a common within-network heterogeneity variance across all comparisons, estimated with the restricted maximum likelihood method [40, 41]. Ondansetron was considered usual care in NMAs for which a placebo was missing. Given that it is clinically reasonable to expect the same between-study heterogeneity variance for the same class of interventions, we assumed that all treatment comparisons within the network were associated with the same magnitude of heterogeneity. The surface under the cumulative ranking (SUCRA) curve was used to rank the safety and effectiveness of the various 5-HT_3_ receptor antagonists [42] and displayed using the rank-heat plot [43].

We conducted sensitivity analyses to ensure that poor-quality studies did not bias the results. Specifically, we conducted separate analyses for RCTs with low risk of bias on the randomization component, the allocation concealment component, or the blinding component, as well as analyses in which the RCTs were combined with other study designs. Selective outcome reporting and reporting bias (e.g., small-study effects) were assessed using the comparison-adjusted funnel plot for outcomes with at least 10 studies in the network, coding treatments from oldest to newest [34].

## Results

### Literature search

**Fig. 1:**
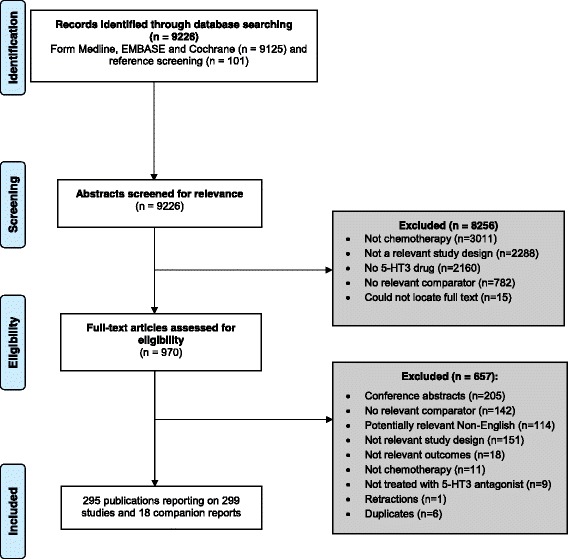
Study flow chart

After screening 9226 citations and 970 full-text articles, we included 299 studies (Additional file 3: Appendix A) that enrolled a total of 58,412 patients (Fig. 1). Six of these studies were conference abstracts that reported relevant unpublished data [Adel et al. 2006, Tabei et al. 2006, Trifilio et al. 2006, Carreca et al. 2007, Kadota et al. 2007, Piyush 2011]. The 299 studies were reported in 295 primary publications. An additional 18 companion reports were used for supplementary material only.

### Study and patient characteristics

The included studies were published between 1985 and 2015, with the largest proportion (based on 5-year intervals) appearing between 1995 and 1999, and nearly half were conducted in Europe (Table 1, Additional file 3: Appendix B). More than 80% of the studies used an RCT design, and more than 40% involved multiple centers. The most commonly examined 5-HT_3_ receptor antagonist was ondansetron. More than 60% of the studies were limited to adults (age ≥ 18 years) (Table 2, Additional file 3: Appendix C). Lung cancer was the most common diagnosis, and more than half of the chemotherapy regimens included cisplatin. Concomitant radiotherapy was reported in less than 5% of the studies.

**Table 1:** Summary of study characteristics

Study characteristics	Number of studies^a^ (*n* = 299)	Percentage of studies
Year of publication		
1985–1989	3	1.00
1990–1994	73	24.41
1995–1999	104	34.78
2000–2004	50	16.72
2005–2009	34	11.37
2010–2015	35	11.71
Geographic region		
Europe	124	41.47
North America	79	26.42
Asia	65	21.74
Multi-continent	19	6.35
South America	4	1.34
Africa	2	0.67
Australia	0	0.00
Not reported	6	2.01
Study design		
RCT	246	82.27
NRCT	33	11.04
CBA	1	0.33
Cohort	19	6.35
Study conduct period		
1980–1989	17	5.69
1990–1999	69	23.08
2000–2009	40	13.38
2010–2013	12	4.01
Not reported	161	53.85
Duration of follow-up^b^		
1	81	27.1
2	10	3.3
3	25	8.4
4	10	3.3
5	73	24.4
6	19	6.4
7	36	12.0
>1 Week	33	11.0
Not reported	12	4.0
Interventions examined: frequency^c^		
Serotonin antagonists: reported as administered alone (administered with corticosteroid)		
Ondansetron	115 (69)	38.46 (23.08)
Granisetron	88 (55)	29.43 (18.39)
Tropisetron	23 (9)	7.69 (3.01)
Dolasetron	19 (7)	6.35 (2.34)
Ramosetron	11 (6)	3.68 (2.01)
Palonosetron	14 (22)	4.68 (7.36)
Comparator antiemetics:		
Metoclopramide	21	7.0
Metoclopramide + steroid	18	6.0
Steroid	3	1.0
Chlorpromazine + steroid	2	0.7
Prochlorperazine	2	0.7
Azasetron + steroid	1	0.3
Chlorpromazine	1	0.3
Metopimazine + steroid	1	0.3
Prochlorperazine + steroid	1	0.3
Serotonin antagonists given with other antiemetic:		
Ondansetron + steroid + metoclopramide	6	2.1
Granisetron + steroid + metoclopramide	6	2.0
Ondansetron + metoclopramide	2	0.7
Granisetron + metoclopramide	1	0.4
Ondansetron + metopimazine	1	0.4
Tropisetron + metopimazine	1	0.4
Granisetron + steroid + prochlorperazine	1	0.4
Ondansetron + steroid + prochlorperazine	1	0.4
Palonosetron + steroid + prochlorperazine	1	0.4
Tropisetron + steroid + metoclopramide	1	0.4
Placebo or no treatment	16	5.67
Outcomes reported: frequency^d^		
Nausea	208	69.6
Vomiting	247	82.6
CINV	117	39.1
Arrhythmia	21	7.0
Delirium	14	4.7
Mortality	41	13.7
Sudden cardiac death	3	1.0
QT interval	25	8.4
Setting		
Multi-center	128	42.8
Single-center	69	23.1
Not specified/not reported	101	33.8

*CBA* controlled before–after, *CINV* chemotherapy-induced nausea and vomiting, *NRCT* non-randomized controlled trial, *RCT* randomized controlled trial

^a^Includes unpublished data from conference abstracts and trial protocols (Adel et al. 2006, Tabei et al. 2006, Trifilio et al. 2006, Carreca et al. 2007, Kadota et al. 2007, Piyush 2011)

^b^ Duration is in days unless otherwise noted

^c^ Multiple interventions and comparators examined across the studies

^d^ Multiple interventions and outcomes reported per study

**Table 2:** Summary of patient characteristics

Patient characteristics	Number of studies (*n* = 299)^a, b^	Percentage of studies
Number of patients	58,412	
Mean (median sample size)	197 (105)^c^	
Mean % female	53.1^d^	
Age category		
Adults and elderly (aged ≥18 years)	192	64.21
Adults only (aged ≥18 to ≤65 years)	56	18.73
Children only (aged <18 years)	25	8.36
All ages	10	3.34
Children and adults (aged ≤65 years)	8	2.68
Not reported	8	2.68
Cancer type^e^		
Lung and respiratory	144	48.16
Breast	110	36.79
Gynecological	98	32.78
Hematological	91	30.43
Unspecified or unknown	87	29.10
Head and neck	87	29.10
Gastrointestinal	86	28.76
Genitourinary	81	27.09
Sarcoma	47	15.72
Not reported	43	14.38
Nervous system	13	4.35
Skin	11	3.68
Optic	5	1.67
Germ cell	5	1.67
Other	3	1.00
Adenocarcinoma	2	0.67
Musculoskeletal	1	0.33
Chemotherapy details		
Cisplatin administered^f^	177	59.20
Cisplatin dose ≥ 50 mg/m^2^	116	38.80
Concomitant radiotherapy	12	4.01
Adjuvant	14	4.68
Neoadjuvant	4	1.34
Patient background details		
History of motion sickness	19	6.35
History of CINV	29	9.70

*CINV* chemotherapy induced nausea and vomiting

^a^Includes unpublished data from conference abstracts and trial protocols (Adel et al. 2006, Tabei et al. 2006, Trifilio et al. 2006, Carrec et al. 2007, Kadota et al. 2007, Piyush 2011)

^b^Except where indicated otherwise

^c^One study did not report sample size (*n* = 1)

^d^Eighteen studies did not report female population size (*n* = 18)

^e^The majority of studies included patients with different diagnoses

^f^Includes the 116 studies with a cisplatin dose ≥ 50 mg/m^2^

### Methodological quality and risk of bias results

Two hundred and forty-six of the studies were assessed with the Cochrane Effective Practice and Organisation of Care risk-of-bias tool (Additional file 3: Appendices D, E). Overall, more than half of the studies were assessed as unclear on all of the following components: random sequence generation, allocation concealment, baseline outcome measure similarities between treatment groups, blinding, contamination, selective outcome reporting, and other bias.

The 19 cohort studies were assessed using the Newcastle–Ottawa Scale. More than half of the studies did not ensure the outcome of interest (e.g., incidence of nausea) was present at the beginning of the study, control for potential confounders, or report the follow-up duration (Additional file 3: Appendix F).

With regard to sources of funding, 153 of the 299 studies did not report the source of funding, 127 were funded by pharmaceutical companies, 17 were publicly funded, and two reported no funding was received. Reporting bias and small-study effects were not observed across the comparison-adjusted funnel plots for all outcomes (Additional file 3: Appendix G).

### Results of statistical analysis

#### Number of patients experiencing harms

**Fig. 2:**
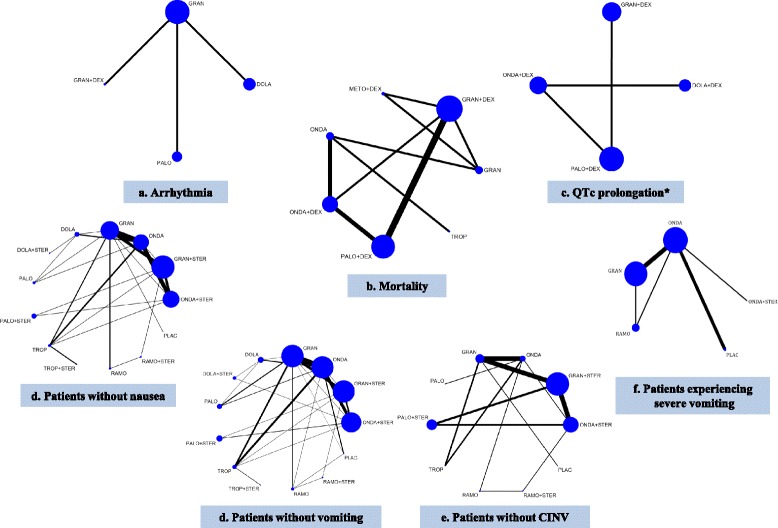
Network plots for all network meta-analyses of the primary analysis. *Abbreviations*: *DOLA* dolasetron, *STER* steroid, *GRAN* granisetron, *DEX* dexamethasone, *METO* metoclopramide, *ONDA* ondansetron, *PALO* palonosetron, *PLAC* placebo, *RAMO* ramosetron, *TROP* tropisetron. *ONDA + DEX and PALO + DEX included only children

**Fig. 3:**
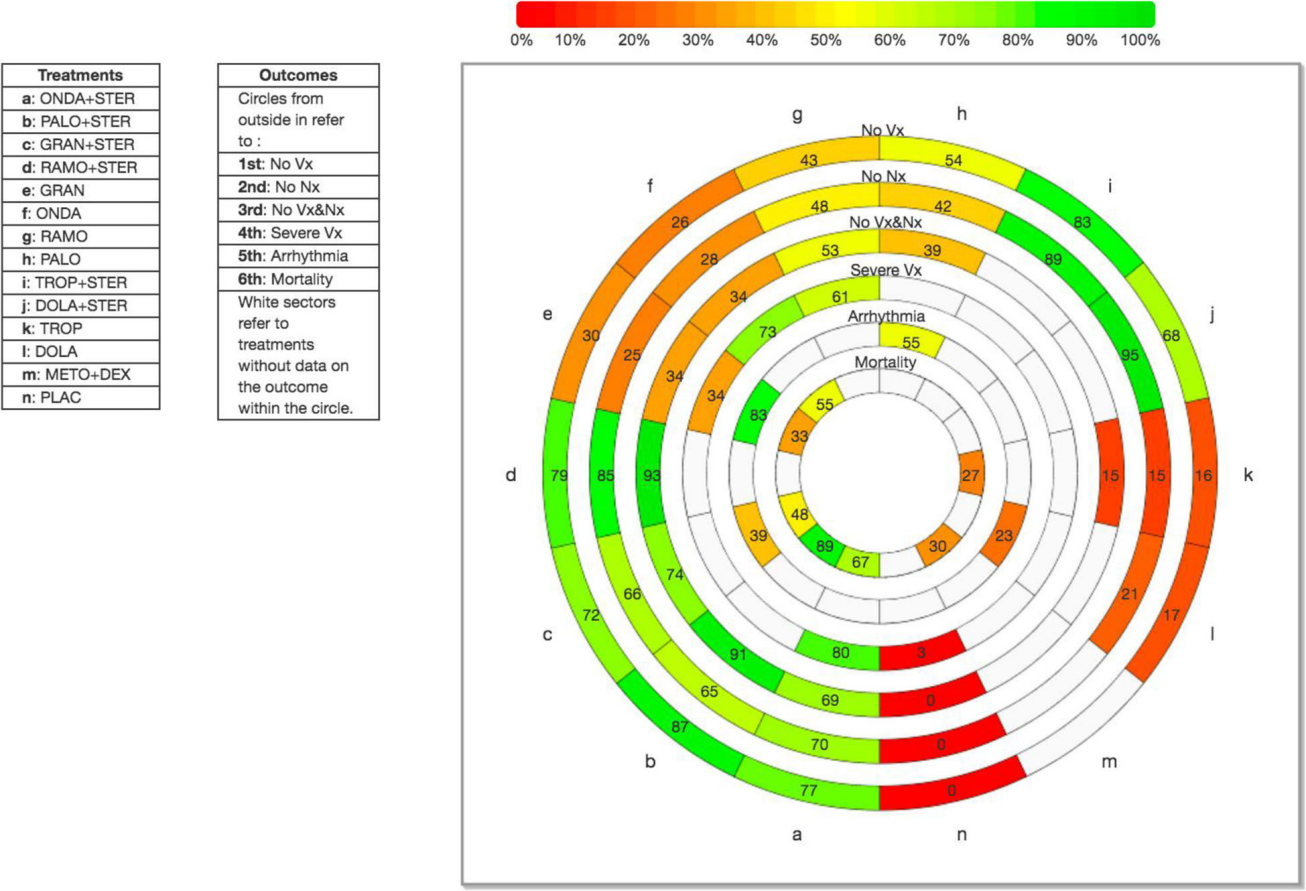
Rank-heat plot including adults in randomized controlled trials. *Abbreviations*: *DOLA* dolasetron, *STER* steroid, *GRAN* granisetron, *DEX* dexamethasone, *METO* metoclopramide, *ONDA* ondansetron, *PALO* palonosetron, *PLAC* placebo, *RAMO* ramosetron, *TROP* tropisetron, *No Vx* number of patients without vomiting, *No Nx* number of patients without nausea, *No Vx&Nx* number of patients without chemotherapy-induced nausea and vomiting, *Severe Vx* number of patients experiencing severe vomiting

NMA for the primary outcome of arrhythmia was attempted using three RCTs (*n* = 627 adults) investigating dolasetron, granisetron, palonosetron, and/or granisetron + dexamethasone (Fig. 2, Additional file 3: Appendix H). A fourth RCT was excluded from the analysis because it reported zero events in all treatment arms [44]. The NMA showed no statistically significant effects (Table 3, Fig. 3, Additional file 3: Appendix H). The safest treatment according to the SUCRA was granisetron (83% probability, Fig. 3). None of the three RCTs examined the same intervention so pairwise meta-analysis was not feasible. The individual results of each trial were not statistically significant, as follows: granisetron versus dolasetron (*n* = 311 patients, OR = 0.21, 95% CI = 0.02–1.84), granisetron + dexamethasone versus granisetron (*n* = 266 patients, OR = 3.12, 95% CI = 0.12–80.39), and palonosetron versus granisetron (*n* = 50 patients, OR = 1.59, 95% CI = 0.58–4.30).

**Table 3:** Statistically significant results of network meta-analysis in randomized controlled trials

Treatment comparison	Number of studies	NMA estimate OR (95% CI)^a^	MA estimate OR (95% CI)
Arrhythmia – 3 RCTs, 4 (inconsistency not applicable^b^)			
No statistically significant results			
Mortality – 8 RCTs, 7 treatments, 4823 adults			
No statistically significant results			
Evaluation of inconsistency using the design-by-treatment interaction model	Chi-squared test: 1.06	*P-*value: 0.79	
	Degrees of freedom: 3	Heterogeneity: 0.00	
QTc prolongation – 4 RCTs, 7 treatments, 3358 children and adults (inconsistency not applicable^b^)			
Ondansetron + dexamethasone vs dolasetron + dexamethasone	1	0.34 (0.24*–*0.47)	0.34 (0.24*–*0.47)
Number of patients without nausea – 44 RCTS, 12 treatments plus placebo, 11,664 adults			
Ondansetron + steroid vs ondansetron	8	1.96 (1.59*–*2.41)	2.16 (1.45*–*3.23)
Ondansetron + steroid vs granisetron	1	2.00 (1.63*–*2.45)	21.00 (3.35*–*131.51)
Ondansetron + steroid vs dolasetron	NA	2.09 (1.47*–*2.97)	NA
Ondansetron + steroid vs dolasetron + steroid	NA	0.44 (0.20*–*0.96)	NA
Ondansetron + steroid vs palonosetron	NA	1.60 (1.06*–*2.41)	NA
Ondansetron + steroid vs tropisetron + steroid	1	2.40 (1.49*–*3.88)	7.20 (2.03*–*25.51)
Ondansetron + steroid vs placebo	NA	337.68 (18.89*–*6035.72)	NA
Granisetron + steroid vs ondansetron	NA	1.91 (1.55*–*2.34)	NA
Granisetron + steroid vs granisetron	6	1.95 (1.62*–*2.34)	1.96 (1.54*–*2.50)
Granisetron + steroid vs dolasetron	NA	2.04 (1.44*–*2.87)	NA
Granisetron + steroid vs dolasetron + steroid	NA	0.43 (0.20*–*0.93)	NA
Granisetron + steroid vs palonosetron	NA	1.55 (1.04*–*2.33)	NA
Granisetron + steroid vs tropisetron + steroid	1	2.34 (1.45*–*3.77)	7.20 (1.29*–*40.05)
Granisetron + steroid vs placebo	NA	328.52 (18.40*–*5864.44)	NA
Ondansetron vs dolasetron + steroid	NA	0.22 (0.10*–*0.48)	NA
Ondansetron vs palonosetron + steroid	NA	0.53 (0.39*–*0.73)	NA
Ondansetron vs tropisetron + steroid	NA	0.29 (0.13*–*0.62)	NA
Ondansetron vs ramosetron + steroid	NA	0.35 (0.19*–*0.64)	NA
Ondansetron vs placebo	NA	172.34 (9.67*–*3070.30)	NA
Granisetron vs dolasetron + steroid	NA	0.22 (0.10*–*0.47)	NA
Granisetron vs palonosetron + steroid	NA	0.52 (0.39*–*0.70)	NA
Granisetron vs tropisetron + steroid	NA	0.28 (0.13*–*0.61)	NA
Granisetron vs ramosetron + steroid	NA	0.34 (0.19*–*0.62)	NA
Granisetron vs placebo	1	168.85 (9.51*–*2996.71)	169.00 (9.52*–*2999.80)
Dolasetron vs dolasetron + steroid	1	0.21 (0.10*–*0.42)	0.21 (0.10*–*0.42)
Dolasetron vs palonosetron + steroid	NA	0.50 (0.33*–*0.76)	NA
Dolasetron vs tropisetron + steroid	NA	0.27 (0.12*–*0.61)	NA
Dolasetron vs ramosetron + steroid	NA	0.33 (0.17*–*0.63)	NA
Dolasetron vs placebo	NA	161.31 (8.95*–*2907.94)	NA
Dolasetron + steroid vs palonosetron	NA	3.65 (1.68*–*7.94)	NA
Dolasetron + steroid vs palonosetron + steroid	NA	2.39 (1.06*–*5.39)	NA
Dolasetron + steroid vs tropisetron + steroid	NA	5.49 (2.27*–*13.27)	NA
Dolasetron + steroid vs ramosetron + steroid	NA	3.19 (1.29*–*7.85)	NA
Dolasetron + steroid vs placebo	NA	771.35 (39.35*–*15,120.97)	NA
Palonosetron vs tropisetron + steroid	NA	0.35 (0.15*–*0.83)	NA
Palonosetron vs ramosetron + steroid	NA	0.43 (0.22*–*0.86)	NA
Palonosetron vs placebo	NA	211.46 (11.64*–*3841.31)	NA
Palonosetron + steroid vs tropisetron + steroid	NA	2.30 (1.35*–*3.91)	NA
Palonosetron + steroid vs placebo	NA	323.08 (17.93*–*5821.31)	NA
Tropisetron + steroid vs tropisetron + steroid	2	0.24 (0.13*–*0.44)	0.24 (0.13*–*0.44)
Tropisetron + steroid vs ramosetron + steroid	NA	0.29 (0.14*–*0.60)	NA
Tropisetron + steroid vs placebo	NA	140.45 (7.63*–*2585.40)	NA
Tropisetron + steroid vs placebo	NA	597.18 (30.37*–*11,744.01)	NA
Ramosetron + steroid vs ramosetron + steroid	1	0.49 (0.31*–*0.78)	0.48 (0.29*–*0.79)
Ramosetron + steroid vs placebo	NA	242.02 (13.10*–*4471.01)	NA
Ramosetron + steroid vs placebo	NA	490.92 (26.08*–*9241.08)	NA
Evaluation of inconsistency using the design-by-treatment interaction model	Chi-squared test: 15.91	*P-*value: 0.1955	
	Degrees of freedom: 12	Heterogeneity: 0.00	
Number of patients without vomiting – 63 RCTs, 12 treatments plus placebo, 15,460 adults			
Ondansetron + steroid vs ondansetron	9	2.46 (1.80*–*3.37)	2.47 (1.52*–*4.03)
Ondansetron + steroid vs granisetron	1	2.35 (1.70*–*3.25)	21.00 (3.35*–*131.51)
Ondansetron + steroid vs dolasetron	NA	2.83 (1.78*–*4.51)	NA
Ondansetron + steroid vs tropisetron	1	2.95 (1.76*–*4.94)	7.20 (2.03*–*25.51)
Ondansetron + steroid vs placebo	NA	31.56 (11.42*–*87.23)	NA
Granisetron + steroid vs ondansetron	NA	2.31 (1.66*–*3.21)	NA
Granisetron + steroid vs granisetron	8	2.21 (1.64*–*2.96)	2.40 (1.21*–*4.75)
Granisetron + steroid vs dolasetron	NA	2.66 (1.66*–*4.27)	NA
Granisetron + steroid vs tropisetron	1	2.77 (1.65*–*4.66)	34.52 (1.83*–*650.54)
Granisetron + steroid vs placebo	NA	29.64 (10.74*–*81.82)	NA
Ondansetron vs dolasetron + steroid	NA	0.46 (0.24*–*0.89)	NA
Ondansetron vs palonosetron	2	0.59 (0.39*–*0.89)	0.44 (0.27*–*0.73)
Ondansetron vs palonosetron + steroid	NA	0.33 (0.18*–*0.59)	NA
Ondansetron vs tropisetron + steroid	NA	0.32 (0.12*–*0.85)	NA
Ondansetron vs ramosetron + steroid	NA	0.37 (0.17*–*0.83)	NA
Ondansetron vs placebo	3	12.82 (4.85*–*33.94)	11.02 (3.30*–*36.84)
Granisetron vs dolasetron + steroid	NA	0.48 (0.25*–*0.94)	NA
Granisetron vs palonosetron	3	0.62 (0.42*–*0.92)	0.69 (0.48*–*0.98)
Granisetron vs palonosetron + steroid	NA	0.35 (0.20*–*0.62)	NA
Granisetron vs tropisetron + steroid	NA	0.34 (0.13*–*0.90)	NA
Granisetron vs ramosetron + steroid	NA	0.39 (0.18*–*0.87)	NA
Granisetron vs placebo	1	13.44 (5.02*–*35.96)	78.00 (6.24*–*974.67)
Dolasetron vs dolasetron + steroid	1	0.40 (0.20*–*0.80)	0.21 (0.09*–*0.50)
Dolasetron vs palonosetron	1	0.51 (0.32*–*0.83)	0.55 (0.36*–*0.85)
Dolasetron vs palonosetron + steroid	NA	0.29 (0.15*–*0.57)	NA
Dolasetron vs tropisetron + steroid	NA	0.28 (0.10*–*0.79)	NA
Dolasetron vs ramosetron + steroid	NA	0.32 (0.14*–*0.78)	NA
Dolasetron vs placebo	NA	11.14 (3.94*–*31.49)	NA
Dolasetron + steroid vs tropisetron	NA	2.61 (1.20*–*5.69)	NA
Dolasetron + steroid vs placebo	NA	27.92 (8.62*–*90.43)	NA
Palonosetron vs tropisetron	NA	2.02 (1.13*–*3.63)	NA
Palonosetron vs placebo	NA	21.65 (7.60*–*61.72)	NA
Palonosetron + steroid vs tropisetron	NA	3.62 (1.78*–*7.37)	NA
Palonosetron + steroid vs ramosetron	NA	2.24 (1.02*–*4.91)	NA
Palonosetron + steroid vs placebo	NA	38.78 (12.56*–*119.76)	NA
Tropisetron vs tropisetron + steroid	2	0.27 (0.11*–*0.64)	0.27 (0.13*–*0.57)
Tropisetron vs ramosetron + steroid	NA	0.31 (0.13*–*0.77)	NA
Tropisetron vs placebo	NA	10.70 (3.69*–*31.01)	NA
Tropisetron + steroid vs placebo	NA	39.86 (10.09*–*157.37)	NA
Ramosetron vs placebo	1	17.30 (6.20*–*48.30)	10.45 (2.21*–*49.38)
Ramosetron + steroid vs placebo	NA	34.31 (10.33*–*113.92)	NA
Evaluation of inconsistency using the design-by-treatment interaction model	Chi-squared test: 22.14	*P-*value: 0.1387	
	Degrees of freedom: 16	Heterogeneity: 0.10	
Number of patients without CINV – 27 RCTs, 10 treatments, 10,924 adults			
Ondansetron + steroid vs ondansetron	1	2.16 (1.62*–*2.87)	1.96 (1.12*–*3.42)
Ondansetron + steroid vs granisetron	NA	2.17 (1.73*–*2.72)	NA
Ondansetron + steroid vs palonosetron + steroid	NA	0.81 (0.67*–*0.98)	NA
Ondansetron + steroid vs tropisetron	NA	4.18 (1.99*–*8.77)	NA
Ondansetron + steroid vs placebo	NA	365.96 (20.44*–*6553.02)	NA
Granisetron + steroid vs ondansetron	NA	2.23 (1.69*–*2.93)	NA
Granisetron + steroid vs granisetron	6	2.24 (1.83*–*2.74)	2.35 (1.88*–*2.94)
Granisetron + steroid vs palonosetron + steroid	NA	0.84 (0.70*–*0.99)	NA
Granisetron + steroid vs tropisetron	NA	4.31 (2.07*–*9.01)	NA
Granisetron + steroid vs placebo	NA	378.03 (21.15*–*6756.45)	NA
Ondansetron vs palonosetron + steroid	NA	0.38 (0.27*–*0.52)	NA
Ondansetron vs ramosetron + steroid	NA	0.31 (0.16*–*0.59)	NA
Ondansetron vs placebo	NA	169.61 (9.48*–*3035.34)	NA
Granisetron vs palonosetron + steroid	NA	0.37 (0.29*–*0.49)	NA
Granisetron vs ramosetron + steroid	NA	0.31 (0.16*–*0.60)	NA
Granisetron vs placebo	1	168.85 (9.51*–*2996.64)	169.00 (9.52*–*2999.80)
Palonosetron vs placebo	NA	165.47 (6.55*–*4182.08)	NA
Palonosetron + steroid vs tropisetron	NA	5.16 (2.43*–*10.97)	NA
Palonosetron + steroid vs placebo	NA	452.19 (25.18*–*8121.41)	NA
Tropisetron vs ramosetron	NA	0.35 (0.14*–*0.83)	NA
Tropisetron vs ramosetron + steroid	NA	0.16 (0.06*–*0.42)	NA
Tropisetron vs placebo	NA	87.64 (4.53*–*1696.76)	NA
Ramosetron vs ramosetron + steroid	1	0.46 (0.29*–*0.73)	0.55 (0.34*–*0.89)
Ramosetron vs placebo	NA	253.30 (13.55*–*4734.53)	NA
Ramosetron + steroid vs placebo	NA	546.71 (28.58*–*10,456.90)	NA
Evaluation of inconsistency using the design-by-treatment interaction model	Chi-squared test: 11.95	*P*-value: 0.0631	
	Degrees of freedom: 6	Heterogeneity: 0.00	
Number of patients experiencing severe vomiting – 10 RCTs, 5 treatments, 917 adults			
Ondansetron vs granisetron	4	0.65 (0.45*–*0.94)	0.63 (0.43*–*0.93)
Ondansetron vs placebo	3	0.16 (0.04*–*0.70)	0.16 (0.04*–*0.70)
Ramosetron vs placebo	NA	0.18 (0.03*–*0.95)	NA
Evaluation of inconsistency using the design-by-treatment interaction model	Chi-squared test: 0.33	*P*-value: 0.5628	
	Degrees of freedom: 1	Heterogeneity: 0.00	

*CI* confidence interval, *CINV* chemotherapy-induced nausea and vomiting, *MA* meta-analysis, *NA* not applicable, *NMA* network meta-analysis, *OR* odds ratio, *RCT* randomized controlled trial

^a^ Meta-analysis was not conducted for treatment comparisons where only one trial was included; in that situation, the direct estimate was obtained from the single trial

^b^ Number of interventions included in NMA is greater than number of RCTs

NMA for the secondary outcome of mortality was conducted with eight RCTs (*n* = 4823 adults) investigating dolasetron, granisetron, ondansetron (usual care), palonosetron, tropisetron, granisetron + dexamethasone, metoclopramide + dexamethasone, ondansetron + dexamethasone, and/or palonosetron + dexamethasone (Fig. 2). A ninth study was excluded from the analysis because it reported zero events in all treatment arms [45], and another study was excluded because it was not connected to the network [46]. The NMA showed no statistically significant effects (Table 3, Additional file 3: Appendix H). The safest treatment according to the SUCRA was palonosetron + dexamethasone (93% probability, Fig. 3). In addition, three pairwise meta-analyses were possible for the following treatment comparisons, which were not statistically significant: palonosetron + dexamethasone versus granisetron + dexamethasone (three RCTs, *n =* 2638 adults, OR = 0.33, 95% CI = 0.09–1.18), ondansetron + dexamethasone versus ondansetron (two RCTs, *n =* 313 adults, OR = 0.53, 95% CI = 0.11–2.58), and palonosetron + dexamethasone versus ondansetron + dexamethasone (two RCTs, *n =* 1101 adults, OR = 0.33, 95% CI = 0.04–2.78).

NMA for the secondary outcome QTc prolongation included four RCTs investigating dolasetron + dexamethasone, granisetron, granisetron + dexamethasone, ondansetron (usual care), ondansetron + dexamethasone, palonosetron, and palonosetron + dexamethasone (*n =* 3358 children and adults, Fig. 2). A fourth RCT was excluded from the analysis because it reported zero events in all treatment arms [38]. The NMA showed that patients taking ondansetron + dexamethasone administration had statistically significantly lower odds of QTc prolongation compared to patients taking dolasetron + dexamethasone (OR = 0.34, 95% CI = 0.24–0.47) (Table 3, Additional file 3: Appendix H). The safest treatment according to the SUCRA was palonosetron + dexamethasone (83% probability). None of the four RCTs examined the same intervention so pairwise meta-analysis was not feasible. Consistent with the NMA results, only one of the individual results of the four RCTs was statistically significant (ondansetron + dexamethasone versus dolasetron + dexamethasone, *n =* 696 patients*,* OR = 0.34, 95% CI = 0.24–0.47). The other results were as follows: ondansetron versus granisetron (*n =* 1056 patients, OR = 7.92, 95% CI = 0.44–143.67), palonosetron versus granisetron (*n =* 597 patients, OR = 2.80, 95% CI = 0.06–141.54), palonosetron + dexamethasone versus granisetron + dexamethasone (*n =* 1119 patients, OR = 0.84, 95% CI = 0.42–1.68), palonosetron versus ondansetron (*n =* 773 patients, OR = 0.35, 95% CI = 0.02–6.42), and palonosetron + dexamethasone versus ondansetron + dexamethasone (*n =* 330 children, OR = 0.20, 95% CI = 0.01–4.10).

NMA and pairwise meta-analysis were not feasible for the secondary outcomes of sudden cardiac death, delirium, and QRS interval prolongation because only one RCT was available for each of these outcomes. For sudden cardiac death, the results were not statistically significant between ondansetron and ondansetron + dexamethasone (*n =* 213 patients, OR = 2.73, 95% CI = 0.11–67.80). For delirium, there were no statistically significant results for any of the treatment comparisons, including metoclopramide + dexamethasone versus granisetron (*n =* 361 patients, OR = 4.14, 95% CI = 0.86–19.93), metoclopramide + dexamethasone versus granisetron + dexamethasone (*n =* 478 patients, OR = 4.07, 95% CI = 0.85–19.59), or granisetron versus granisetron + dexamethasone (*n =* 597 patients, OR = 0.98, 95% CI = 0.01–7.10). For QRS interval prolongation, the available RCT only reported results for more than 24 h of chemotherapy; ondansetron administered on days 1–7 plus dexamethasone resulted in statistically significantly fewer patients experiencing a prolonged QRD interval when compared to dolasetron + dexamethasone (OR = 0.29, 95% CI = 0.19–0.48).

When the studies reporting these harms were appraised using the McHarm tool (Additional file 3: Appendices I, J), the majority were assessed as unclear or partially fulfilling all 15 criteria other than active collection of mode of harm, timing and frequency of harms collection, use of standard scales for collection of harms data, reporting of whether harms encompassed all patients or a sample of patients, and number of harmful events reported for each group, which were commonly scored unclear overall.

#### Number of patients without nausea

NMA for the secondary outcome of the number of patients without nausea within 24 h was attempted with 47 RCTs and 11,778 patients, as well as a NMA including 51 studies with 12,188 patients (including randomized and non-randomized studies), yet statistically significant inconsistency was observed for both NMAs.

NMA for the 44 RCTs (*n* = 11,664 adults) investigating 12 treatments plus placebo (Fig. 2, Additional file 4: Appendix A) showed that all of the treatments were significantly superior to placebo in increasing the proportion of patients without nausea (Table 3, Additional file 3: Appendices K, L). In this analysis, dolasetron + steroid had the highest SUCRA (Fig. 3, Additional file 3: Appendix M), with a 95% probability of being the most effective agent, followed closely by tropisetron + steroid (89% probability). The same results were observed in another subgroup analysis for patients who received cisplatin chemotherapy (23 RCTs and 6259 patients) (Additional file 3: Appendix N). In an analysis of three pediatric RCTs of four treatments (granisetron, ondansetron, palonosetron, tropisetron; *n* = 293 children), palonosetron was statistically significantly superior to ondansetron, and granisetron was statistically significantly superior to tropisetron. Palonosetron had the highest SUCRA (88% probability).

A sensitivity analysis was conducted with 14 RCTs assessed as having a low risk of randomization bias (*n* = 4970 patients, 10 treatments). Ondansetron was superior to dolasetron and granisetron, while all other comparisons were superior to ondansetron. However, only palonosetron + steroid, granisetron + steroid, ondansetron + steroid, and ramosetron + steroid were statistically significantly superior to ondansetron (Additional file 3: Appendix N). Ramosetron + steroid had the highest SUCRA (94% probability). Another sensitivity analysis was conducted that included 14 RCTs with a low risk of allocation concealment bias (*n* = 4199 patients, nine treatments). All treatments except dolasetron increased the proportion of patients without nausea versus ondansetron. However, only palonosetron + steroid, granisetron + steroid, ondansetron + steroid, and dolasetron + steroid were statistically significant. Dolasetron + steroid had the highest SUCRA (95% probability). A third sensitivity analysis was conducted including 11 RCTs with a low risk of blinding bias (*n* = 3858 patients, seven treatments). Compared to ondansetron, all treatments except dolasetron and granisetron increased the proportion of patients without nausea. However, only granisetron + steroid, ondansetron + steroid, and palonosetron were statistically significant. Palonosetron had the highest SUCRA (99% probability).

NMA was conducted with 31 RCTs (*n =* 8108 patients, 30 treatments plus placebo) for the number of patients without nausea more than 24 h after chemotherapy. All of the dosing schedules and medications were superior to placebo, but only the following schedules were statistically significant﻿: ondansetron + steroid on day 1 of chemotherapy and at least one subsequent day, palonosetron on day 1 of chemotherapy, ondansetron + steroid on day 1 of chemotherapy and at least one subsequent day + metoclopramide on days 2–5 of chemotherapy, and tropisetron + steroid on day 1 of chemotherapy and at least one subsequent day. The last of these schedules had the highest SUCRA (96% probability). Notably, dolasetron + steroid was not included in this NMA because none of the studies reported this intervention.

#### Number of patients without vomiting

NMA for the secondary outcome of number of patients without vomiting within 24 h after chemotherapy was conducted with 71 RCTs (*n* = 16,300 adults, 12 treatments plus placebo; Additional file 4: Appendix B). All of the treatments were statistically significantly superior to placebo for this outcome (Additional file 3: Appendix O). Palonosetron + steroid had the highest SUCRA (90% probability; Additional file 3: Appendix P), followed closely by tropisetron + steroid (79% probability). The same results were observed in another analysis that included 75 randomized and non-randomized studies (*n* = 16,710 patients, 12 treatments plus placebo), as well as subgroup analysis including 63 RCTs with 15,460 adults, and 12 treatments plus placebo (Figs 2 and 3, Table 3, Additional file 3: Appendix Q). Similar results were observed in another subgroup analysis including 69 RCTs and 15,742 patients who received cisplatin chemotherapy (12 treatments plus placebo); however, in this subgroup analysis, dolasetron + steroid and tropisetron + steroid both had a higher SUCRA (89% probability) than palonosetron + steroid (78% probability). In an analysis of five pediatric RCTs including a total of 411 children and testing six treatments plus placebo (granisetron, ondansetron, ondansetron + steroid, palonosetron, tropisetron, placebo), granisetron and tropisetron were statistically significantly superior to placebo, and granisetron had the highest SUCRA (84% probability).

A sensitivity analysis was conducted including 21 RCTs assessed as having a low risk of randomization bias (*n =* 6549 patients, 10 treatments plus placebo). Relative to placebo, all treatments increased the proportion of patients without vomiting, although none of the results were statistically significant (Additional file 3: Appendix Q). The highest SUCRA values were found in palonosetron + steroid (84% probability) and ramosetron + steroid (81% probability). Another sensitivity analysis was conducted including 21 RCTs with a low risk of allocation concealment bias (*n =* 6315 patients, 11 treatments). Relative to ondansetron, all treatments except dolasetron increased the proportion of patients without vomiting. The proportion of patients without vomiting was statistically significantly higher with palonosetron, palonosetron + steroid, and ondansetron + steroid versus ondansetron. Palonosetron + steroid had the highest SUCRA (73% probability). A third sensitivity analysis was conducted including 20 RCTs with a low risk of blinding bias (*n =* 6232 patients, nine treatments plus placebo). Compared with placebo, all treatments statistically significantly increased the proportion of patients without vomiting. The highest SUCRA values were found for palonosetron (81% probability), palonosetron + steroid (81% probability), and ondansetron + steroid (79% probability).

NMA was attempted with 48 RCTs (*n* = 9425 patients) that reported the number of patients without vomiting more than 24 h after chemotherapy. However, there was inconsistency between direct and indirect evidence. Therefore, a subgroup analysis was conducted with 45 RCTs involving only adults (*n* = 8845 patients, 26 treatments plus placebo). All of the dosing schedules and medications were numerically superior to placebo ﻿and there were 21 treatments that were also statistically superior to placebo. However, the following schedules were not statistically significant﻿﻿: ramosetron only on day 1 of chemotherapy, dolasetron only on day 1 and at least one subsequent day, ramosetron + steroid on day 1 and at least one subsequent day, and granisetron + steroid on day 1 and at least one subsequent day. Dolasetron + steroid on day 1 had the highest SUCRA (94% probability) along with palonosetron + steroid on day 1 (94% probability).

#### Number of patients without CINV

NMA was attempted for the secondary outcome of number of patients without CINV within 24 h of chemotherapy including 28 RCTs with 11,252 patients, as well as a second NMA including 26 studies with 10,014 patients (including randomized and non-randomized studies), yet statistically significant inconsistency was observed in both NMAs.

NMA for 27 RCTs involving 10,924 adults (nine treatments plus placebo) was conducted (Fig. 2, Additional file 4: Appendix C). All of the treatments were statistically significantly superior to placebo for this outcome (Table 3, Additional file 3: Appendices K, R). Ramosetron + steroid had the highest SUCRA (93% probability), followed by palonosetron + steroid (91% probability; Fig. 3, Additional file 3: Appendix S). Similar results were observed in another subgroup analysis including 15 RCTs and 5250 patients receiving cisplatin chemotherapy (nine treatments plus placebo; Additional file 3: Appendix T). In another subgroup analysis including eight RCTs for 3066 patients who did not receive cisplatin chemotherapy and seven treatments, palonosetron + steroid, ondansetron + steroid, and granisetron + steroid were statistically significantly superior to ondansetron and had the highest SUCRAs (Additional file 3: Appendix T).

A sensitivity analysis was conducted including eight RCTs assessed as having a low risk of randomization bias (*n* = 3677 patients, five treatments). Compared to ondansetron, all treatments except granisetron significantly increased the proportion of patients without CINV (Additional file 3: Appendix T). Palonosetron + steroid had the highest SUCRA (85% probability). A second sensitivity analysis was conducted including five RCTs with a low risk of allocation concealment bias (*n* = 2771 patients, four treatments). Granisetron + steroid, palonosetron + steroid, and ondansetron + steroid increased the proportion of patients without CINV versus ondansetron, yet granisetron + steroid was not statistically significant. Ondansetron + steroid had the highest SUCRA (89% probability).

NMA was conducted with 26 RCTs (*n =* 8851 patients, 22 treatments plus placebo) that reported the number of patients without CINV at more than 24 h after chemotherapy. All treatments reduced the risk of CINV relative to placebo, and the reduction was statistically significant for the following regimens: ondansetron on day 1 of chemotherapy only + steroid on day 1 and at least one subsequent day, granisetron on day 1 only, ondansetron + steroid on day 1 and at least one subsequent day, granisetron on day 1 only + steroid on day 1 and at least one subsequent day, palonosetron on day 1 only, dolasetron on day 1 and at least one subsequent day + steroid on day 1 only, ondansetron + steroid on day 1 and at least one subsequent day with metoclopramide on days 2–5, palonosetron on day 1 only + steroid on day 1 and at least one subsequent day, tropisetron + steroid on day 1 and at least one subsequent day, and ramosetron on day 1 and at least one subsequent day. Ramosetron on day 1 and at least one subsequent day had the highest SUCRA (90% probability), followed by tropisetron + steroid on day 1 and at least one subsequent day (88% probability).

#### Number of patients experiencing severe vomiting

NMA was conducted for the secondary outcome of number of patients experiencing severe vomiting (defined as vomiting five times or more) within 24 h after chemotherapy. In this analysis, 11 RCTs, 1364 adults, and six treatments plus placebo were included (Fig. 2, Additional file 4: Appendix D). All treatments were superior to placebo in reducing the risk of severe vomiting, but only ondansetron and ramosetron were statistically significantly superior (Table 3, Additional file 3: Appendices K, U). Ondansetron + steroid had the highest SUCRA (80% probability), followed closely by ondansetron (73% probability; Additional file 3: Appendix V). Similar results were observed in a secondary analysis including 13 randomized and non-randomized study designs (*n =* 1600 patients, eight treatments plus placebo), except that tropisetron + steroid had the highest SUCRA (83% probability). The same results as the primary analysis were observed in a subgroup analysis including seven RCTs and 677 adults receiving cisplatin chemotherapy (six treatments plus placebo) (Additional file 3: Appendix W).

## Discussion

We conducted a systematic review and NMA on 5-HT_3_ receptor antagonists for patients undergoing chemotherapy. Our results suggest that all treatments are relatively safe, but we were unable to conduct an NMA on sudden cardiac death, prolonged QRS interval, or delirium because of a dearth of data. Future RCTs to examine the safety of these treatments should include these important outcomes. As well, the studies included in our NMAs of arrhythmia, QTc prolongation, and mortality did not have a placebo comparator, and we are therefore unable to comment on the safety of these treatments relative to placebo.

Overall, our results suggest that all of these treatments are effective for reducing nausea and vomiting experienced by patients undergoing chemotherapy. According to the rank-heat plot, the treatment that is most likely the safest and most effective is palonosetron + steroid. Our findings can be used by patients and their clinicians to tailor their choice of treatments. For example, if a patient is most concerned about CINV during the first 24 h after chemotherapy, then ramosetron + steroid may be the best choice. Across the effectiveness outcomes, the following treatments ranked as most superior on three effectiveness outcomes during the first 24 h after chemotherapy for adults: ondansetron + steroid, palonosetron + steroid, granisetron + steroid, and ramosetron + steroid. If a patient is most concerned about CINV occurring more than 24 h after chemotherapy, then ramosetron given on day 1 and at least one subsequent day, tropisetron + steroid given on day 1 and at least one subsequent day, or palonosetron given on day 1 + steroid given on day 1 and at least one subsequent day are potentially effective options.

For the outcome of the number of patients without vomiting, some nuances in the results are worth mentioning. In the NMA for the proportion of patients without vomiting more than 24 h after chemotherapy, dolasetron + steroid administered on day 1 of chemotherapy ranked high in the SUCRA analysis. However, dolasetron + steroid did not rank highest in the SUCRA analysis for the proportion of patients without vomiting within 24 h after chemotherapy. This apparent discrepancy might be due to the structure of the different networks. For example, different interventions were included between the studies assessing treatment within 24 h of chemotherapy versus those targeting vomiting more than 24 h after chemotherapy, which might have affected the results. The differing results might also be attributable to heterogeneity, given that some treatment comparisons will have different magnitudes than others included in each NMA.

For the NMA on severe vomiting, the combination of ondansetron + steroid was numerically superior to all of the other treatments, but the results were not statistically significant. This might be due to a lack of power because only one small RCT (*n* = 20 patients) examined ondansetron + steroid versus ondansetron. However, because of the large effect sizes, this treatment ranked high on the SUCRA analysis. Therefore, the SUCRA results for the outcome of severe vomiting should be interpreted with caution.

We are aware of four previous systematic reviews that examined 5-HT_3_ receptor antagonists for nausea and vomiting [24–27]. Only two of these reviews examined harms, such as dizziness, fever, headaches, and constipation [26, 27]. We included more studies (*n* = 200) and more patients (*n* = 30,864) than any of these reviews (Additional file 3: Appendix X), but we also excluded some of the studies that were included in those earlier reviews; reasons for those exclusions are presented in Additional file 3: Appendix Y.

The studies included in our NMAs had some limitations. Most of the studies were small, with an average sample size of 197 patients. This limitation is particularly problematic for assessing harms, because larger sample sizes are required to draw definitive conclusions. Approximately 40% of the included studies were funded by pharmaceutical companies, which may have resulted in funding bias. In addition, random sequence generation, allocation concealment, and blinding were unclear for more than half of the RCTs. All of the studies failed to ensure that the outcome of interest (e.g., no nausea) was present at the start of the study. In addition, few of the included studies reported on the emetogenicity setting (e.g., highly emetogenic chemotherapy). Despite these methodological shortcomings, we did not observe reporting bias or small-study effects in our comparison-adjusted funnel plot analysis for all outcomes.

Our systematic review process also had some limitations. Because of the large number of included studies, revisions to our original protocol [21] were necessary. For example, we were unable to include all conference abstracts and reports written in languages other than English. Moreover, we did not conduct a sensitivity analysis around the node selection for the NMA, as we assumed that the effects of different doses and durations were identical across treatments. We are now exploring these assumptions in another study [47]. In addition, clinical practice guidelines recommend prophylaxis with a 5-HT_3_ receptor antagonist, steroid, and a NK1 receptor antagonist, yet none of the included studies examined this combination of treatments. As such, our effectiveness results should be interpreted with caution. As well, NMA should only be attempted when the studies are sufficiently homogenous. As such, we explored whether the transitivity assumption was upheld and found that confounding variables were generally well balanced across the treatment comparisons in our NMA. However, some of the studies may not have reported all important confounding variables so this is a limitation of our study. Although we planned to include non-randomized studies in our analyses of harms, we found only RCTs reporting the outcomes of interest. Finally, we were unable to conduct an analysis stratified by emetogenicity, because of varied reporting of chemotherapy regimens and classification of chemotherapy regimens by type of emetogenic agent over time [9].

## Conclusions

From this study, we conclude that most 5-HT_3_ receptor antagonists alone or combined with steroids decrease the occurrence of nausea and/or vomiting. Most 5-HT_3_ receptor antagonists were relatively safe when compared with each other, yet none of the studies compared active treatment with placebo for harms. Dolasetron + dexamethasone may prolong the QTc compared to ondansetron + dexamethasone. Additional studies are needed to characterize the cardiac and cognitive safety of these treatments. Until then, it would be prudent for clinicians to obtain baseline electrocardiographic tracings before prescribing these common, effective antiemetics to any patients who are undergoing chemotherapy.

## Additional files

Additional file 1: Protocol (PDF 121 kb)
Additional file 2: PRISMA checklist (PDF 3237 kb)
Additional file 3: Appendices A–Y (PDF 1123 kb)
Additional file 4: Appendices A–D (PDF 211 kb)

## Abbreviations

5-HT_3_: Serotonin
CI: Confidence interval
CINV: Chemotherapy-induced nausea and vomiting
NMA: Network meta-analysis
OR: Odds ratio
RCTs: Randomized controlled trials
SUCRA: Surface under the cumulative ranking
