# Supplementary material for: Comparative safety and effectiveness of serotonin receptor antagonists in patients undergoing chemotherapy: a systematic review and network meta-analysis
Source: BMC Med. 2016 Dec 23;14:216. doi: 10.1186/s12916-016-0761-9 (PMC5180412; doi:10.1186/s12916-016-0761-9)
Supplement: Additional file 1: — Protocol (PDF 121 kb) [file 12916_2016_761_MOESM1_ESM.pdf]

PROSPERO International prospective register of systematic reviews

Safety of serotonin (5-HT3) receptor antagonists in patients undergoing surgery and chemotherapy: a systematic review and network meta-analysis

Andrea Tricco, Charlene Soobiah, Jesmin Antony, Brenda Hemmelgarn, David Moher, Brian Hutton, Sharon Straus

Citation

Andrea Tricco, Charlene Soobiah, Jesmin Antony, Brenda Hemmelgarn, David Moher, Brian Hutton, Sharon Straus. Safety of serotonin (5-HT3) receptor antagonists in patients undergoing surgery and chemotherapy: a systematic review and network meta-analysis. PROSPERO 2013:CRD42013003564 Available from http://www.crd.york.ac.uk/PROSPERO_REBRANDING/display_record.asp?ID=CRD42013003564

Review question(s)

In patients undergoing surgery or chemotherapy, what is the comparative safety of serotonin (5-HT3) receptor antagonists?

Searches

The databases searched will include MEDLINE (OVID interface, 1970 to September 2012), EMBASE (OVID interface, 1970 to September 2012), and The Cochrane Library (Wiley interface, Issue 1, 2012). The database search will be supplemented by searching for grey literature which will include searching of public health, drug regulatory and trial registry websites (e.g., Public Health Agency of Canada, Health Canada, FDA, Clinical Trials.gov), websites of organizations that produce guidelines (e.g., Canadian Agency for Drugs and Technologies in Health, Centre for Disease Control and Prevention, World Health Organization, AHRQ, National Institute for Health and Clinical Excellence), and conference abstracts (International Pharmaceutical conference). In addition, we will also conduct general Internet searches in Google.

Types of study to be included

We will include experimental (e.g., randomized clinical trials (RCTs), quasi-RCTs, controlled clinical trials), quasi-experimental (interrupted time series, controlled before after studies) and observational (cohort) studies.

Condition or domain being studied

Patients undergoing surgery or chemotherapy who received 5-HT3 receptor antagonists.

Participants/ population

We will include studies of adults and children undergoing surgery or chemotherapy. We will analyse these populations separately.

Intervention(s), exposure(s)

First generation 5-HT3 receptor antagonists approved for use in many countries internationally will be included. These include ondansetron, granisetron, and dolasetron. Second generation antagonists, such as palonosetron, will also be considered for inclusion. We will include studies of monotherapy of any dose, formulation, and duration.

Comparator(s)/ control

We will explore the safety of each of the 5-HT3 receptor antagonists against each other, against placebo, and/or other antiemetic agents including benzamides, phenothiazines, butyrophenones, antihistamines, and anticholinergics.

Context

All medical contexts will be included.

Outcome(s)

Primary outcomes

The primary outcome is arrhythmia.

Secondary outcomes
Secondary outcomes include sudden cardiac death, QT prolongation, PR prolongation, all-cause mortality, nausea, vomiting, and delirium.

Data extraction, (selection and coding)
After a training exercise, two reviewers will screen all titles and abstracts for inclusion, independently (Level 1 screening). They will then independently review the full-text article of potentially relevant articles to determine inclusion (Level 2 screening). Another training exercise will be conducted on the data abstraction form and subsequently, all data will be abstracted in duplicate. The data abstracted will include study characteristics (e.g., study design, year of conduct), participant characteristics (e.g., number of patients, age mean and standard deviation), and outcome results (e.g. arrhythmia, QT prolongation). Discrepancies will be resolved by discussion or the involvement of a third reviewer.

Risk of bias (quality) assessment
We will appraise the methodological quality/risk of bias using standardized quality assessment tools for design-specific internal validity, as follows:

•RCTs (Cochrane Risk of Bias Tool).

•Controlled clinical trials, interrupted time series, and controlled before-after studies (Cochrane Effective Practice and Organisation of Care Risk of Bias (EPOC) Tool).

•Cohort studies (Newcastle-Ottawa Scale).

Publication bias will be assessed using funnel plots.

Strategy for data synthesis
We will first describe our results narratively and then will conduct meta-analysis (and network meta-analysis), if deemed appropriate.

Analysis of subgroups or subsets
We will analyse chemotherapy separate from those undergoing surgery. In addition we will also analyse adults (aged <=18 years) and children (aged < 18 years) separately.

Dissemination plans
Our results will be disseminated through dissemination meetings, executive summaries for clinicians and policy-makers, conference presentations, and publications in open access journals.

Contact details for further information
Sharon E. Straus

sharon.straus@utoronto.ca

Organisational affiliation of the review
Li Ka Shing Knowledge Institute of St. Michael's Hospital

http://www.stmichaelshospital.com/knowledgeinstitute/

Review team
Dr Andrea Tricco, Li Ka Shing Knowledge institute of St. Michael's Hospital
Ms Charlene Soobiah, Li Ka Shing Knowledge institute of St. Michael's Hospital
Ms Jesmin Antony, Li Ka Shing Knowledge institute of St. Michael's Hospital
Dr Brenda Hemmelgarn, University of Calgary
Dr David Moher, University of Ottawa
Dr Brian Hutton, University of Ottawa
Dr Sharon Straus, Li Ka Shing Knowledge institute of St. Michael's Hospital

Collaborators
Dr Joseph Beyene, University of Toronto
Dr Maggie Hong Chen, Li Ka Shing Knowledge institute of St. Michael's Hospital

Anticipated or actual start date
14 January 2013

Anticipated completion date
13 December 2013

Funding sources/sponsors
Funded by the Canadian Institute of Health Research (CIHR) Drug Safety and Effectiveness Network (DSEN). ACT is funded by a CIHR/DSEN New Investigator Award in Knowledge Synthesis. SES is supported by a Tier 1 Canada Research Chair.

Conflicts of interest
None known

Language
English

Country
Canada

Subject index terms status
Subject indexing assigned by CRD

Subject index terms
Antiemetics; Antineoplastic Agents; Drug Therapy, Combination; Humans; Receptors, Serotonin, 5-HT3; Serotonin 5-HT3 Receptor Antagonists

Stage of review
Ongoing

Date of registration in PROSPERO
04 January 2013

Date of publication of this revision
04 January 2013

DOI
10.15124/CRD42013003564

Stage of review at time of this submission	Started	Completed
Preliminary searches	Yes	No
Piloting of the study selection process	No	No
Formal screening of search results against eligibility criteria	No	No
Data extraction	No	No
Risk of bias (quality) assessment	No	No
Data analysis	No	No

The information in this record has been provided by the named contact for this review. CRD has accepted this information in good faith and registered the review in PROSPERO. CRD bears no responsibility or liability for the content of this registration record, any associated files or external websites.
