# Supplementary material for: Comparative safety and effectiveness of serotonin receptor antagonists in patients undergoing chemotherapy: a systematic review and network meta-analysis
Source: BMC Med. 2016 Dec 23;14:216. doi: 10.1186/s12916-016-0761-9 (PMC5180412; doi:10.1186/s12916-016-0761-9)
Supplement: Additional file 2: — PRISMA checklist (PDF 3237 kb) [file 12916_2016_761_MOESM2_ESM.pdf]

Supplementary Online Content

Appendix A. References for Included Studies 2
Appendix B. Study Characteristics 20
Appendix C. Patient Characteristics 32
Appendix D. Aggregate Cochrane EPOC risk-of-bias appraisal results 47
Appendix E. Cochrane EPOC risk-of-bias appraisal results 48
Appendix F. Newcastle-Ottawa Scale Appraisal Results 53
Appendix G. Comparison Adjusted Funnel Plots for patients without nausea, patients without vomiting, patients without CINV, and patients experiencing severe vomiting outcomes 54
Appendix H. Results for harms outcomes analyses 55
Appendix I. Aggregate Mcharms appraisal results 58
Appendix J. Results for Mcharms appraisal 60
Appendix K. Results of the primary Network meta-analysis for effectiveness outcomes 62
Appendix L. No. of patients without nausea – RCTs and adults only 67
Appendix M. No. of patients without nausea – RCTs and adults only – SUCRA values for each treatment 68
Appendix N. No. of patients without nausea sub-group analysis for Network meta-analysis 69
Appendix O. No. of patients without vomiting – RCTs only 72
Appendix P. No. of patients without vomiting – RCTs only – SUCRA values for each treatment 73
Appendix Q. No. of patients without vomiting sub-group analysis for Network meta-analysis 74
Appendix R. No. of patients without CINV – RCTs only 77
Appendix S. No. of patients without CINV – RCTs only – SUCRA values for each treatment 78
Appendix T. No. of patients without CINV sub-group analysis for Network meta-analysis 79
Appendix U. No. of patients experiencing severe vomiting – RCTs only 81
Appendix V. No. of patients experiencing severe vomiting – RCTs only – SUCRA values for each treatment 82
Appendix W. No. of patients experiencing severe vomiting sub-group analysis for Network meta-analysis 83
Appendix X. Included studies in our review versus previous systematic reviews 85
Appendix Y. List of excluded studies from previous systematic reviews 90

Appendix A. References for Included Studies

Primary studies (295 publications reporting on 299 studies): [1-250] [251-289] [290-295]

Companion Reports (n =18): [296-313]

1. Ondansetron versus granisetron, both combined with dexamethasone, in the prevention of cisplatin-induced emesis. Italian Group of Antiemetic Research. Ann Oncol. 1995;6(8):805-10.
2. A double-blind randomized study comparing intramuscular (i.m.) granisetron with i.m. granisetron plus dexamethasone in the prevention of delayed emesis induced by cisplatin. The Italian Multicenter Study Group. Anticancer Drugs. 1999;10(5):465-70.
3. Aapro MS, Grunberg SM, Manikhas GM, Olivares G, Suarez T, Tjulandin SA, et al. A phase III, double-blind, randomized trial of palonosetron compared with ondansetron in preventing chemotherapy-induced nausea and vomiting following highly emetogenic chemotherapy. Ann Oncol. 2006;17(9):1441-9.
4. Aapro MS, Thuerlimann B, Sessa C, De Pree C, Bernhard J, Maibach R, et al. A randomized double-blind trial to compare the clinical efficacy of granisetron with metoclopramide, both combined with dexamethasone in the prophylaxis of chemotherapy-induced delayed emesis. Ann Oncol. 2003;14(2):291-7.
5. Abali H, Celik I. Tropisetron, ondansetron, and granisetron for control of chemotherapy-induced emesis in Turkish cancer patients: a comparison of efficacy, side-effect profile, and cost. Cancer Invest. 2007;25(3):135-9.
6. Abang AM, Takemoto MH, Pham T, Mandanas RA, Roy V, Selby GB, et al. Efficacy and safety of oral granisetron versus i.v. granisetron in patients undergoing peripheral blood progenitor cell and bone marrow transplantation. Anticancer Drugs. 2000;11(2):137-42.
7. Adams M, Soukop M, Barley V, Yosef H, Anderson H, Boesen E, et al. Tropisetron alone or in combination with dexamethasone for the prevention and treatment of emesis induced by non-cisplatin chemotherapy: a randomized trial. Anticancer Drugs. 1995;6(4):514-21.
8. Addelman M, Erlichman C, Fine S, Warr D, Murray C. Phase I/II trial of granisetron: a novel 5-hydroxytryptamine antagonist for the prevention of chemotherapy-induced nausea and vomiting. J Clin Oncol. 1990;8(2):337-41.
9. Advani SH, Gopal R, Dhar AK, Lal HM, Cooverji ND. Comparative evaluation of the clinical efficacy and safety of ondansetron and metoclopramide in the prophylaxis of emesis induced by cancer chemotherapy regimens including cisplatin. J Assoc Physicians India. 1996;44(2):127-30.
10. Agura ED, Brown MC, Schaffer R, Donaldson G, Shen DD. Antiemetic efficacy and pharmacokinetics of intravenous ondansetron infusion during chemotherapy conditioning for bone marrow transplant. Bone Marrow Transplant. 1995;16(2):213-22.
11. Ahn MJ, Lee JS, Lee KH, Suh C, Choi SS, Kim SH. A randomized double-blind trial of ondansetron alone versus in combination with dexamethasone versus in combination with dexamethasone and lorazepam in the prevention of emesis due to cisplatin-based chemotherapy. Am J Clin Oncol. 1994;17(2):150-6.
12. Aksoylar S, Akman SA, Ozgenc F, Kansoy S. Comparison of tropisetron and granisetron in the control of nausea and vomiting in children receiving combined cancer chemotherapy. Pediatr Hematol Oncol. 2001;18(6):397-406.
13. Alfieri AB, Cubeddu LX. Comparative efficacy of a single oral dose of ondansetron and of buspirone against cisplatin-induced emesis in cancer patients. Br J Cancer. 1995;72(4):1013-5.
14. Alvarez O, Freeman A, Bedros A, Call SK, Volsch J, Kalbermatter O, et al. Randomized double-blind crossover ondansetron-dexamethasone versus ondansetron-placebo study for the treatment of chemotherapy-induced nausea and vomiting in pediatric patients with malignancies. J Pediatr Hematol Oncol. 1995;17(2):145-50.
15. Audhuy B, Cappelaere P, Martin M, Cervantes A, Fabbro M, Riviere A, et al. A double-blind, randomised comparison of the anti-emetic efficacy of two intravenous doses of dolasetron mesilate and granisetron in patients receiving high dose cisplatin chemotherapy. European journal of cancer. 1996;32A(5):807-13.
16. Babaoglu MO, Bayar B, Aynacioglu AS, Kerb R, Abali H, Celik I, et al. Association of the ABCB1 3435C>T polymorphism with antiemetic efficacy of 5-hydroxytryptamine type 3 antagonists. Clin Pharmacol Ther. 2005;78(6):619-26.

17. Barrajon E, de las Penas R. Randomised double blind crossover study comparing ondansetron, granisetron and tropisetron. A cost-benefit analysis. Support Care Cancer. 2000;8(4):323-33.
18. Barrenetxea G, Schneider J, Centeno MM, Romero H, de la Rica M, Rodriguez-Escudero FJ. Chemotherapy-induced emesis: management of early and delayed emesis in milder emetogenic regimens. Cancer Chemother Pharmacol. 1996;38(5):471-5.
19. Beck TM, Ciociola AA, Jones SE, Harvey WH, Tchekmedyian NS, Chang A, et al. Efficacy of oral ondansetron in the prevention of emesis in outpatients receiving cyclophosphamide-based chemotherapy. The Ondansetron Study Group. Ann Intern Med. 1993;118(6):407-13.
20. Beck TM, Hesketh PJ, Madajewicz S, Navari RM, Pendergrass K, Lester EP, et al. Stratified, randomized, double-blind comparison of intravenous ondansetron administered as a multiple-dose regimen versus two single-dose regimens in the prevention of cisplatin-induced nausea and vomiting. J Clin Oncol. 1992;10(12):1969-75.
21. Beck TM, York M, Chang A, Navari R, Harvey WH, Meshad M, et al. Oral ondansetron 8 mg twice daily is as effective as 8 mg three times daily in the prevention of nausea and vomiting associated with moderately emetogenic cancer chemotherapy. S3A-376 Study Group. Cancer Invest. 1997;15(4):297-303.
22. Belkacemi Y, Ozsahin M, Pene F, Rio B, Sutton L, Laporte JP, et al. Total body irradiation prior to bone marrow transplantation: efficacy and safety of granisetron in the prophylaxis and control of radiation-induced emesis. Int J Radiat Oncol Biol Phys. 1996;36(1):77-82.
23. Berrak SG, Ozdemir N, Bakirci N, Turkkan E, Canpolat C, Beker B, et al. A double-blind, crossover, randomized dose-comparison trial of granisetron for the prevention of acute and delayed nausea and emesis in children receiving moderately emetogenic carboplatin-based chemotherapy. Support Care Cancer. 2007;15(10):1163-8.
24. Bhatia A, Tripathi KD, Sharma M. Comparison of ondansetron with metoclopramide in prevention of acute emesis associated with low dose & high dose cisplatin chemotherapy. Indian J Med Res. 2003;118:33-41.
25. Birch R, Weaver CH, Carson K, Buckner CD. A randomized trial of once vs twice daily administration of intravenous granisetron with dexamethosone in patients receiving high-dose cyclophosphamide, thiotepa and carboplatin. Bone Marrow Transplant. 1998;22(7):685-8.
26. Blazer M, Phillips G, Reardon J, Efries D, Smith Y, Weatherby L, et al. Antiemetic control with palonosetron in patients with gastrointestinal cancer receiving a fluoropyrimidine-based regimen in addition to either irinotecan or oxaliplatin: a retrospective study. Oncology. 2012;83(3):135-40.
27. Bleiberg HH, Spielmann M, Falkson G, Romain D. Antiemetic treatment with oral granisetron in patients receiving moderately emetogenic chemotherapy: a dose-ranging study. Clin Ther. 1995;17(1):38-51.
28. Blijham GH. Dose granisetron remain effective over multiple cycles? The Granisetron Study Group. European journal of cancer. 1992;28A Suppl 1:S17-21.
29. Boccia R, Grunberg S, Franco-Gonzales E, Rubenstein E, Voisin D. Efficacy of oral palonosetron compared to intravenous palonosetron for the prevention of chemotherapy-induced nausea and vomiting associated with moderately emetogenic chemotherapy: a phase 3 trial. Support Care Cancer. 2013;21(5):1453-60.
30. Boccia RV, Gordan LN, Clark G, Howell JD, Grunberg SM, Sancuso Study G. Efficacy and tolerability of transdermal granisetron for the control of chemotherapy-induced nausea and vomiting associated with moderately and highly emetogenic multi-day chemotherapy: a randomized, double-blind, phase III study. Support Care Cancer. 2011;19(10):1609-17.
31. Bonneterre J, Chevallier B, Metz R, Fargeot P, Pujade-Lauraine E, Spielmann M, et al. A randomized double-blind comparison of ondansetron and metoclopramide in the prophylaxis of emesis induced by cyclophosphamide, fluorouracil, and doxorubicin or epirubicin chemotherapy. J Clin Oncol. 1990;8(6):1063-9.
32. Bonneterre J, Hecquet B. Granisetron (IV) compared with ondansetron (IV plus oral) in the prevention of nausea and vomiting induced by moderately-emetogenic chemotherapy. A cross-over study. Bull Cancer. 1995;82(12):1038-43.
33. Bosi A, Guidi S, Messori A, Saccardi R, Lombardini L, Vannucchi AM, et al. Ondansetron versus chlorpromazine for preventing emesis in bone marrow transplant recipients: a double-blind randomized study. J Chemother. 1993;5(3):191-6.

34. Bots MW, Dijkstra HJ, Blijham GH, van der Wall E, Fehmers MC, Keizer HJ, et al. A randomized trial of two doses of granisetron in the treatment of chemotherapy-induced emesis. Dutch results within a multinational study. Neth J Med. 1992;40(5-6):221-6.
35. Brock P, Brichard B, Rechnitzer C, Langeveld NE, Lanning M, Soderhall S, et al. An increased loading dose of ondansetron: a north european, double-blind randomised study in children, comparing 5 mg/m2 with 10 mg/m2. European journal of cancer. 1996;32A(10):1744-8.
36. Brown RS, Brown TK, Hoare D, Gaze MN. An audit of antiemetic use with CMF chemotherapy. Clin Oncol (R Coll Radiol). 1998;10(5):313-7.
37. Buser KS, Joss RA, Piquet D, Aapro MS, Cavalli F, Haefliger JM, et al. Oral ondansetron in the prophylaxis of nausea and vomiting induced by cyclophosphamide, methotrexate and 5-fluorouracil (CMF) in women with breast cancer. Results of a prospective, randomized, double-blind, placebo-controlled study. Ann Oncol. 1993;4(6):475-9.
38. Buyukavci M, Olgun H, Ceviz N. The effects of ondansetron and granisetron on electrocardiography in children receiving chemotherapy for acute leukemia. Am J Clin Oncol. 2005;28(2):201-4.
39. Carmichael J, Bessell EM, Harris AL, Hutcheon AW, Dawes PJ, Daniels S, et al. Comparison of granisetron alone and granisetron plus dexamethasone in the prophylaxis of cytotoxic-induced emesis. Br J Cancer. 1994;70(6):1161-4.
40. Carmichael J, Harris AL. High-dose i.v. granisetron for the prevention of chemotherapy-induced emesis: cardiac safety and tolerability. Anticancer Drugs. 2003;14(9):739-44.
41. Chang CS, Chen LT, Huang SM, Liu TC, Lin SF, Chen TP, et al. Comparison of intravenous granisetron with metoclopramide plus dexamethasone in the prevention of nausea and vomiting associated with emetogenic cytotoxic chemotherapy. Kaohsiung J Med Sci. 1997;13(2):97-102.
42. Cheirsilpa A, Sinthusake T, Songsakkaesorn A, Visawaprasit S, Chulaka K, Changkuingdee N. Comparison of ramosetron and granisetron for the prevention of acute and delayed emesis in Cisplatin-based chemotherapy: a randomized controlled trial. Jpn J Clin Oncol. 2005;35(12):695-9.
43. Chen PT, Liaw CC. Intravenous ondansetron plus intravenous dexamethasone with different ondansetron dosing schedules during multiple cycles of cisplatin-based chemotherapy. Chang Gung Med J. 2008;31(2):167-74.
44. Chevallier B. The control of acute cisplatin-induced emesis--a comparative study of granisetron and a combination regimen of high-dose metoclopramide and dexamethasone. Granisetron Study Group. Br J Cancer. 1993;68(1):176-80.
45. Chevallier B, Cappelaere P, Splinter T, Fabbro M, Wendling JL, Cals L, et al. A double-blind, multicentre comparison of intravenous dolasetron mesilate and metoclopramide in the prevention of nausea and vomiting in cancer patients receiving high-dose cisplatin chemotherapy. Support Care Cancer. 1997;5(1):22-30.
46. Chevallier B, Marty M, Paillarse JM. Methylprednisolone enhances the efficacy of ondansetron in acute and delayed cisplatin-induced emesis over at least three cycles. Ondansetron Study Group. Br J Cancer. 1994;70(6):1171-5.
47. Chiou TJ, Tzeng WF, Wang WS, Yen CC, Fan FS, Liu JH, et al. Comparison of the efficacy and safety of oral granisetron plus dexamethasone with intravenous ondansetron plus dexamethasone to control nausea and vomiting induced by moderate/severe emetogenic chemotherapy. Zhonghua Yi Xue Za Zhi (Taipei). 2000;63(10):729-36.
48. Chiou TJ, Wei CH, Hsieh RK, Fan FS, Liu JH, Chen PM. Comparison of intravenous granisetron with metoclopramide in the treatment of chemotherapy-induced emesis. Zhonghua Yi Xue Za Zhi (Taipei). 1995;56(1):23-30.
49. Chiu EW, Liang R, Lie A, Todd D, Chan TK. Comparison of Ondansetron with Metoclopramide in the Control of Emesis Induced by Moderately Emetogenic Chemotherapy Used for Lymphoma and Leukaemia Patients. Drug Investigation. 1994;8(2):104-9.
50. Chua DT, Sham JS, Kwong DL, Kwok CC, Yue A, Foo YC, et al. Comparative efficacy of three 5-HT3 antagonists (granisetron, ondansetron, and tropisetron) plus dexamethasone for the prevention of cisplatin-induced acute emesis: a randomized crossover study. Am J Clin Oncol. 2000;23(2):185-91.
51. Conroy T, Cappelaere P, Fabbro M, Fauser AA, Splinter TA, Spielmann M, et al. Acute antiemetic efficacy and safety of dolasetron mesylate, a 5-HT3 antagonist, in cancer patients treated with cisplatin. European Dolasetron Study Group. Am J Clin Oncol. 1994;17(2):97-102.

52. Contu A, Olmeo N, Piro S, Sabbatini R, Depenni R, Silingardi V, et al. A comparison of the antiemetic efficacy and safety of intramuscular and intravenous formulations of granisetron in patients receiving moderately emetogenic chemotherapy. Anticancer Drugs. 1995;6(5):652-6.
53. Coppes MJ, Lau R, Ingram LC, Wiernikowski JT, Grant R, Howard DR, et al. Open-label comparison of the antiemetic efficacy of single intravenous doses of dolasetron mesylate in pediatric cancer patients receiving moderately to highly emetogenic chemotherapy. Med Pediatr Oncol. 1999;33(2):99-105.
54. Coppes MJ, Yanofsky R, Pritchard S, Leclerc JM, Howard DR, Perrotta M, et al. Safety, tolerability, antiemetic efficacy, and pharmacokinetics of oral dolasetron mesylate in pediatric cancer patients receiving moderately to highly emetogenic chemotherapy. J Pediatr Hematol Oncol. 1999;21(4):274-83.
55. Corapcioglu F, Sarper N. A prospective randomized trial of the antiemetic efficacy and cost-effectiveness of intravenous and orally disintegrating tablet of ondansetron in children with cancer. Pediatr Hematol Oncol. 2005;22(2):103-14.
56. Crucitt MA, Hyman W, Grote T, Tester W, Madajewicz S, Yee S, et al. Efficacy and tolerability of oral ondansetron versus prochlorperazine in the prevention of emesis associated with cyclophosphamide-based chemotherapy and maintenance of health-related quality of life. Clin Ther. 1996;18(4):778-88.
57. Cubeddu LX, Hoffman IS, Fuenmayor NT, Finn AL. Antagonism of serotonin S3 receptors with ondansetron prevents nausea and emesis induced by cyclophosphamide-containing chemotherapy regimens. J Clin Oncol. 1990;8(10):1721-7.
58. Cubeddu LX, Hoffmann IS, Fuenmayor NT, Finn AL. Efficacy of ondansetron (GR 38032F) and the role of serotonin in cisplatin-induced nausea and vomiting. N Engl J Med. 1990;322(12):810-6.
59. Cubeddu LX, Pendergrass K, Ryan T, York M, Burton G, Meshad M, et al. Efficacy of oral ondansetron, a selective antagonist of 5-HT3 receptors, in the treatment of nausea and vomiting associated with cyclophosphamide-based chemotherapies. Ondansetron Study Group. Am J Clin Oncol. 1994;17(2):137-46.
60. Cunningham D, Gore M, Davidson N, Miocevich M, Manchanda M, Wells N. The real costs of emesis--an economic analysis of ondansetron vs. metoclopramide in controlling emesis in patients receiving chemotherapy for cancer. European journal of cancer. 1993;29A(3):303-6.
61. Cunningham D, Turner A, Hawthorn J, Rosin RD. Ondansetron with and without dexamethasone to treat chemotherapy-induced emesis. Lancet. 1989;1(8650):1323.
62. Cupissol DR, Serrou B, Caubel M. The efficacy of granisetron as a prophylactic anti-emetic and intervention agent in high-dose cisplatin-induced emesis. European journal of cancer. 1990;26 Suppl 1:S23-7.
63. Davidson N, Rapoport B, Erikstein B, L'Esperance B, Ruff P, Paska W, et al. Comparison of an orally disintegrating ondansetron tablet with the conventional ondansetron tablet for cyclophosphamide-induced emesis in cancer patients: a multicenter, double-masked study. Ondansetron Orally Disintegrating Tablet Emesis Study Group. Clin Ther. 1999;21(3):492-502.
64. Davidson NG, Paska W, Van Belle S, Goedhals L, McQuade B, McRae J. Ondansetron suppository: a randomised, double-blind, double-dummy, parallel-group comparison with oral ondansetron for the prevention of cyclophosphamide-induced emesis and nausea. The Ondansetron Suppository emesis study group. Oncology. 1997;54(5):380-6.
65. de Haan LD, de Mulder PH, Beex LV, Debruyne FM, Challoner T, de Pauw BE. The efficacy of GR38032F, an antagonist of 5-hydroxytryptamine-3 (5-HT3) in the prophylaxis of cisplatin (CDDP)-induced nausea and vomiting. Eur J Cancer Clin Oncol. 1988;24(8):1383-4.
66. De Mulder PH, Seynaeve C, Vermorken JB, van Liessum PA, Mols-Jevdevic S, Allman EL, et al. Ondansetron compared with high-dose metoclopramide in prophylaxis of acute and delayed cisplatin-induced nausea and vomiting. A multicenter, randomized, double-blind, crossover study. Ann Intern Med. 1990;113(11):834-40.
67. de Wet M, Falkson G, Rapoport BL. Repeated use of granisetron in patients receiving cytostatic agents. Cancer. 1993;71(12):4043-9.
68. de Wit R, Beijnen JH, van Tellingen O, Schellens JH, de Boer-Dennert M, Verweij J. Pharmacokinetic profile and clinical efficacy of a once-daily ondansetron suppository in cyclophosphamide-induced emesis: a double blind comparative study with ondansetron tablets. Br J Cancer. 1996;74(2):323-6.
69. de Wit R, de Boer AC, vd Linden GH, Stoter G, Sparreboom A, Verweij J. Effective cross-over to granisetron after failure to ondansetron, a randomized double blind study in patients failing ondansetron

plus dexamethasone during the first 24 hours following highly emetogenic chemotherapy. Br J Cancer. 2001;85(8):1099-101.
70. Dempsey CL, Coop AJ, Shillington A, Farley PA, Eberhardt DR, O'Briant S. Antiemetic effectiveness of ondansetron and granisetron in patients with breast cancer treated with cyclophosphamide. Am J Health Syst Pharm. 2004;61(8):781-6.
71. DiBenedetto J, Jr., Cubeddu LX, Ryan T, Kish JA, Sciortino D, Beall C, et al. Ondansetron for nausea and vomiting associated with moderately emetogenic cancer chemotherapy. Clin Ther. 1995;17(6):1091-8.
72. Dicato MA, Kaasa S, Campora E, Bleiberg H, Warnier P, Vindevoghel A, et al. Efficacy of twice daily versus three times daily oral ondansetron in the prevention of chemotherapy induced emesis: a randomized, single-blind, multicentre study. The Ondansetron International Emesis Study Group. Clin Oncol (R Coll Radiol). 1992;4(5):275-9.
73. Dick GS, Meller ST, Pinkerton CR. Randomised comparison of ondansetron and metoclopramide plus dexamethasone for chemotherapy induced emesis. Arch Dis Child. 1995;73(3):243-5.
74. Dong X, Huang J, Cao R, Liu L. Palonosetron for prevention of acute and delayed nausea and vomiting in non-small-cell lung carcinoma patients. Med Oncol. 2011;28(4):1425-9.
75. du Bois A, McKenna CJ, Andersson H, Lahousen M, Kitchener H, Pinter T, et al. A randomised, double-blind, parallel-group study to compare the efficacy and safety of ondansetron (GR38032F) plus dexamethasone with metoclopramide plus dexamethasone in the prophylaxis of nausea and emesis induced by carboplatin chemotherapy. Oncology. 1997;54(1):7-14.
76. Dundee JW, McMillan CM, Yang J, Wright PM. Is ondansetron a less effective antiemetic against moderately emetic as compared with highly emetic chemotherapy? Br J Clin Pharmacol. 1992;33(2):200-1.
77. Eisenberg P, Figueroa-Vadillo J, Zamora R, Charu V, Hajdenberg J, Cartmell A, et al. Improved prevention of moderately emetogenic chemotherapy-induced nausea and vomiting with palonosetron, a pharmacologically novel 5-HT3 receptor antagonist: results of a phase III, single-dose trial versus dolasetron. Cancer. 2003;98(11):2473-82.
78. Eisenberg P, MacKintosh FR, Ritch P, Cornett PA, Macciocchi A. Efficacy, safety and pharmacokinetics of palonosetron in patients receiving highly emetogenic cisplatin-based chemotherapy: a dose-ranging clinical study. Ann Oncol. 2004;15(2):330-7.
79. Endo J, Iihara H, Yamada M, Yanase K, Kamiya F, Ito F, et al. A randomized controlled non-inferiority study comparing the antiemetic effect between intravenous granisetron and oral azasetron based on estimated 5-HT3 receptor occupancy. Anticancer Res. 2012;32(9):3939-47.
80. Fabi A, Ciccarese M, Metro G, Savarese A, Giannarelli D, Nuzzo CM, et al. Oral ondansetron is highly active as rescue antiemetic treatment for moderately emetogenic chemotherapy: results of a randomized phase II study. Support Care Cancer. 2008;16(12):1375-80.
81. Falkson HC, Falkson CI, Falkson G. High versus low dose granisetron, a selective 5HT3 antagonist, for the prevention of chemotherapy-induced nausea and vomiting. Invest New Drugs. 1990;8(4):407-9.
82. Farley PA, Dempsey CL, Shillington AA, Kulis-Robitaille C, Colgan K, Bernstein G. Patients' self-reported functional status after granisetron or ondansetron therapy to prevent chemotherapy-induced nausea and vomiting at six cancer centers. Am J Health Syst Pharm. 1997;54(21):2478-82.
83. Fauser AA, Bleiberg H, Chevallier B, Favre R, Fabbro M, Noble A, et al. A double-blind, randomized, parallel study of IV dolasetron mesilate versus IV metoclopramide in patients receiving moderately emetogenic chemotherapy. The Cancer Journal. 1996;9(4):196-202.
84. Fauser AA, Duclos B, Chemaissani A, Del Favero A, Cognetti F, Diaz-Rubio E, et al. Therapeutic equivalence of single oral doses of dolasetron mesilate and multiple doses of ondansetron for the prevention of emesis after moderately emetogenic chemotherapy. European Dolasetron Comparative Study Group. European journal of cancer. 1996;32A(9):1523-9.
85. Fauser AA, Pizzocaro G, Schueller J, Khayat D, Wilkinson P. A double-blind, randomised, parallel study comparing intravenous dolasetron plus dexamethasone and intravenous dolasetron alone for the management of fractionated cisplatin-related nausea and vomiting. Support Care Cancer. 2000;8(1):49-54.
86. Fedele P, Spina M, Valentini M, Collini D, Lucia C, Manicone M, et al. Ondansetron versus granisetron in the prevention of chemotherapy-induced nausea and vomiting. Cancer. 1995;76(3):535-6.

87. Feng F, Zhang P, He Y, Li Y, Zhou M, Chen G, et al. Clinical comparison of the selective serotonin3 antagonists ramosetron and granisetron in treating acute chemotherapy-induced emesis, nausea and anorexia. Chin Med Sci J. 2002;17(3):168-72.
88. Feng FY, Zhang P, He YJ, Li YH, Zhou MZ, Cheng G, et al. Oral formulations of the selective serotonin3 antagonists ramosetron (intraoral disintegrator formulation) and granisetron hydrochloride (standard tablet) in treating acute chemotherapy-induced emesis, nausea, and anorexia: A multicenter, randomized, single-blind, crossover, comparison study. Current therapeutic research, clinical and experimental. 2002;63(11):725-35.
89. Forni C, Ferrari S, Loro L, Mazzei T, Beghelli C, Biolchini A, et al. Granisetron, tropisetron, and ondansetron in the prevention of acute emesis induced by a combination of cisplatin-Adriamycin and by high-dose ifosfamide delivered in multiple-day continuous infusions. Support Care Cancer. 2000;8(2):131-3.
90. Fox-Geiman MP, Fisher SG, Kiley K, Fletcher-Gonzalez D, Porter N, Stiff P. Double-blind comparative trial of oral ondansetron versus oral granisetron versus IV ondansetron in the prevention of nausea and vomiting associated with highly emetogenic preparative regimens prior to stem cell transplantation. Biol Blood Marrow Transplant. 2001;7(11):596-603.
91. Fraschini G, Ciociola A, Esparza L, Templeton D, Holmes FA, Walters RS, et al. Evaluation of three oral dosages of ondansetron in the prevention of nausea and emesis associated with cyclophosphamide-doxorubicin chemotherapy. J Clin Oncol. 1991;9(7):1268-74.
92. Friedman CJ, Burris HA, 3rd, Yocom K, Blackburn LM, Gruben D. Oral granisetron for the prevention of acute late onset nausea and vomiting in patients treated with moderately emetogenic chemotherapy. Oncologist. 2000;5(2):136-43.
93. Fujimoto TH, T.; Shimizu, H.; Oka, T.; Kikuta, A.; Mugishima, H.; Tonouchi, T.; Koizumi, S.; Takaue, Y.; Okamura, J. A controlled, dose- comparison study of granisetron for nausea and vomiting induced by cancer chemotherapy in children. . Int J Clin Oncol. 1996;1:57-60.
94. Fumoleau P, Giovannini M, Rolland F, Votan B, Paillarse JM. Ondansetron suppository: an effective treatment for the prevention of emetic disorders induced by cisplatin-based chemotherapy. French Ondansetron Study Group. Oral Oncol. 1997;33(5):354-8.
95. Gaedicke G, Erttmann R, Henze G, Hartmann W, Drechsler S, Grass P, et al. Pharmacokinetics of the 5HT3 receptor antagonist tropisetron in children. Pediatr Hematol Oncol. 1996;13(5):405-16.
96. Gandara DR. Progress in the control of acute and delayed emesis induced by cisplatin. Eur J Cancer. 1991;27 Suppl 1:S9-11; discussion S22.
97. Gebbia V, Cannata G, Testa A, Curto G, Valenza R, Cipolla C, et al. Ondansetron versus granisetron in the prevention of chemotherapy-induced nausea and vomiting. Results of a prospective randomized trial. Cancer. 1994;74(7):1945-52.
98. Gebbia V, Testa A, Valenza R, Cannata G, Tirrito ML, Gebbia N. Oral granisetron with or without methylprednisolone versus metoclopramide plus methylprednisolone in the management of delayed nausea and vomiting induced by cisplatin-based chemotherapy. A prospective randomized trial. Cancer. 1995;76(10):1821-8.
99. Ghosh S, Dey S. Comparing different antiemetic regimens for chemotherapy induced nausea and vomiting. International Journal of Collaborative Research on Internal Medicine and Public Health. 2010;2(5):142-56.
100. Giralt SA, Mangan KF, Maziarz RT, Bubalo JS, Beveridge R, Hurd DD, et al. Three palonosetron regimens to prevent CINV in myeloma patients receiving multiple-day high-dose melphalan and hematopoietic stem cell transplantation. Ann Oncol. 2011;22(4):939-46.
101. Gralla R, Lichinitser M, Van Der Vegt S, Sleeboom H, Mezger J, Peschel C, et al. Palonosetron improves prevention of chemotherapy-induced nausea and vomiting following moderately emetogenic chemotherapy: results of a double-blind randomized phase III trial comparing single doses of palonosetron with ondansetron. Ann Oncol. 2003;14(10):1570-7.
102. Gralla RJ, Navari RM, Hesketh PJ, Popovic W, Strupp J, Noy J, et al. Single-dose oral granisetron has equivalent antiemetic efficacy to intravenous ondansetron for highly emetogenic cisplatin-based chemotherapy. J Clin Oncol. 1998;16(4):1568-73.
103. Gridelli C, Ianniello G, Ambrosini G, Mustacchi G, Pedicini T, Farris A, et al. A multicentre, double-blind, randomised trial comparing ondansetron versus ondansetron plus dexamethasone in the prophylaxis of cisplatin-induced delayed emesis. Int J Oncol. 1997;10(2):395-400.

104. Grote TH, Pineda LF, Figlin RA, Pendergrass KB, Hesketh PJ, Karlan BY, et al. Oral dolasetron mesylate in patients receiving moderately emetogenic platinum-containing chemotherapy. Oral Dolasetron Dose Response Study Group. Cancer J Sci Am. 1997;3(1):45-51.
105. Grunberg SM, Lane M, Lester EP, Sridhar KS, Mortimer J, Murphy W, et al. Randomized double-blind comparison of three dose levels of intravenous ondansetron in the prevention of cisplatin-induced emesis. Cancer Chemother Pharmacol. 1993;32(4):268-72.
106. Grunberg SM, Stevenson LL, Russell CA, McDermed JE. Dose ranging phase I study of the serotonin antagonist GR38032F for prevention of cisplatin-induced nausea and vomiting. J Clin Oncol. 1989;7(8):1137-41.
107. Hahlen K, Quintana E, Pinkerton CR, Cedar E. A randomized comparison of intravenously administered granisetron versus chlorpromazine plus dexamethasone in the prevention of ifosfamide-induced emesis in children. J Pediatr. 1995;126(2):309-13.
108. Hainsworth J, Harvey W, Pendergrass K, Kasimis B, Oblon D, Monaghan G, et al. A single-blind comparison of intravenous ondansetron, a selective serotonin antagonist, with intravenous metoclopramide in the prevention of nausea and vomiting associated with high-dose cisplatin chemotherapy. J Clin Oncol. 1991;9(5):721-8.
109. Hamadani M, Chaudhary L, Awan FT, Khan JK, Kojouri K, Ozer H, et al. Management of platinum-based chemotherapy-induced acute nausea and vomiting: is there a superior serotonin receptor antagonist? J Oncol Pharm Pract. 2007;13(2):69-75.
110. Handberg J, Wessel V, Larsen L, Herrstedt J, Hansen HH. Randomized, double-blind comparison of granisetron versus granisetron plus prednisolone as antiemetic prophylaxis during multiple-day cisplatin-based chemotherapy. Support Care Cancer. 1998;6(1):63-7.
111. Harris EM, Shillington AC, Dempsey CL, Blackwell B, A. FP. Benchmarking evaluation of 5-HT3-receptor antagonist use in emetogenic chemotherapy. Formulary. 1997;32(5):515-22.
112. Heron JF, Goedhals L, Jordaan JP, Cunningham J, Cedar E. Oral granisetron alone and in combination with dexamethasone: a double-blind randomized comparison against high-dose metoclopramide plus dexamethasone in prevention of cisplatin-induced emesis. The Granisetron Study Group. Ann Oncol. 1994;5(7):579-4.
113. Herrington JD, Kwan P, Young RR, Lagow E, Lagrone L, Riggs MW. Randomized, multicenter comparison of oral granisetron and oral ondansetron for emetogenic chemotherapy. Pharmacotherapy. 2000;20(11):1318-23.
114. Herrstedt J, Sigsgaard T, Boesgaard M, Jensen TP, Dombernowsky P. Ondansetron plus metopimazine compared with ondansetron alone in patients receiving moderately emetogenic chemotherapy. N Engl J Med. 1993;328(15):1076-80.
115. Herrstedt J, Sigsgaard TC, Nielsen HA, Handberg J, Langer SW, Ottesen S, et al. Randomized, double-blind trial comparing the antiemetic effect of tropisetron plus metopimazine with tropisetron plus placebo in patients receiving multiple cycles of multiple-day cisplatin-based chemotherapy. Support Care Cancer. 2007;15(4):417-26.
116. Hesketh P, Navari R, Grote T, Gralla R, Hainsworth J, Kris M, et al. Double-blind, randomized comparison of the antiemetic efficacy of intravenous dolasetron mesylate and intravenous ondansetron in the prevention of acute cisplatin-induced emesis in patients with cancer. Dolasetron Comparative Chemotherapy-induced Emesis Prevention Group. J Clin Oncol. 1996;14(8):2242-9.
117. Hesketh PJ, Beck T, Uhlenhopp M, Kris MG, Hainsworth JD, Harker WG, et al. Adjusting the dose of intravenous ondansetron plus dexamethasone to the emetogenic potential of the chemotherapy regimen. J Clin Oncol. 1995;13(8):2117-22.
118. Hesketh PJ, Crews JR, Cohen R, Blackburn LM, Friedman CJ. Antiemetic efficacy of single-dose oral granisetron (1 mg vs 2 mg) with moderately emetogenic chemotherapy. Cancer J. 2000;6(3):157-61.
119. Hesketh PJ, Gandara DR, Hesketh AM, Facada A, Perez EA, Webber LM, et al. Dose-ranging evaluation of the antiemetic efficacy of intravenous dolasetron in patients receiving chemotherapy with doxorubicin or cyclophosphamide. Support Care Cancer. 1996;4(2):141-6.
120. Hesketh PJ, Harvey WH, Harker WG, Beck TM, Ryan T, Bricker LJ, et al. A randomized, double-blind comparison of intravenous ondansetron alone and in combination with intravenous dexamethasone in the prevention of high-dose cisplatin-induced emesis. J Clin Oncol. 1994;12(3):596-600.
121. Ho CL, Su WC, Hsieh RK, Lin ZZ, Chao TY. A randomized, double-blind, parallel, comparative study to evaluate the efficacy and safety of ramosetron plus dexamethasone injection for the prevention of acute chemotherapy-induced nausea and vomiting. Jpn J Clin Oncol. 2010;40(4):294-301.

122. Hulstaert F, Van Belle S, Bleiberg H, Canon JL, Dewitte M, Buyse M, et al. Optimal combination therapy with tropisetron in 445 patients with incomplete control of chemotherapy-induced nausea and vomiting. J Clin Oncol. 1994;12(11):2439-46.
123. Hunter B, Aapro M, Piguet D, Giger K, Bauer J, Haefliger JM, et al. The antiemetic efficacy and safety of granisetron compared with metoclopramide plus dexamethasone in patients receiving fractionated chemotherapy over 5 days. The Granisetron Study Group. J Cancer Res Clin Oncol. 1993;119(9):555-9.
124. Ithimakin S, Runglodvatana K, Nimmannit A, Akewanlop C, Srimuninnimit V, Keerativitayanan N, et al. Randomized, double-blinded, placebo-controlled trial of ondansetron plus dexamethasone with or without metoclopramide as antiemetic prophylaxis in patients receiving high-dose cisplatin in medical practice. Support Care Cancer. 2012;20(4):849-55.
125. Jaing TH, Tsay PK, Hung IJ, Yang CP, Hu WY. Single-dose oral granisetron versus multidose intravenous ondansetron for moderately emetogenic cyclophosphamide-based chemotherapy in pediatric outpatients with acute lymphoblastic leukemia. Pediatr Hematol Oncol. 2004;21(3):227-35.
126. Jantunen IT, Kataja VV, Johansson RT. Ondansetron and tropisetron with dexamethasone in the prophylaxis of acute vomiting induced by non-cisplatin-containing chemotherapy. Acta Oncol. 1992;31(5):573-5.
127. Jantunen IT, Muhonen TT, Kataja VV, Flander MK, Teerenhovi L. 5-HT3 receptor antagonists in the prophylaxis of acute vomiting induced by moderately emetogenic chemotherapy--a randomised study. European journal of cancer. 1993;29A(12):1669-72.
128. Jones AL, Hill AS, Soukop M, Hutcheon AW, Cassidy J, Kaye SB, et al. Comparison of dexamethasone and ondansetron in the prophylaxis of emesis induced by moderately emetogenic chemotherapy. Lancet. 1991;338(8765):483-7.
129. Joss RA, Bacchi M, Buser K, Kirchner V, Neuenschwander H, Orth B, et al. Ondansetron plus dexamethasone is superior to ondansetron alone in the prevention of emesis in chemotherapy-naive and previously treated patients. Swiss Group for Clinical Cancer Research (SAKK). Ann Oncol. 1994;5(3):253-8.
130. Kaasa S, Kvaloy S, Dicato MA, Ries F, Huys JV, Royer E, et al. A comparison of ondansetron with metoclopramide in the prophylaxis of chemotherapy-induced nausea and vomiting: a randomized, double-blind study. International Emesis Study Group. European journal of cancer. 1990;26(3):311-4.
131. Kaizer L, Warr D, Hoskins P, Latreille J, Lofters W, Yau J, et al. Effect of schedule and maintenance on the antiemetic efficacy of ondansetron combined with dexamethasone in acute and delayed nausea and emesis in patients receiving moderately emetogenic chemotherapy: a phase III trial by the National Cancer Institute of Canada Clinical Trials Group. J Clin Oncol. 1994;12(5):1050-7.
132. Kalaycio M, Mendez Z, Pohlman B, Overmoyer B, Boparai N, Jones E, et al. Continuous-infusion granisetron compared to ondansetron for the prevention of nausea and vomiting after high-dose chemotherapy. J Cancer Res Clin Oncol. 1998;124(5):265-9.
133. Kandemir EG, Yaylaci M, Uskent N. Comparison of ondansetron plus dexamethasone with metoclopramide plus dexamethasone in the control of cisplatin-induced delayed emesis. The Official Journal of Balkan Union of Oncology. 1999;4(3):289-93.
134. Kang YK, Park YH, Ryoo BY, Bang YJ, Cho KS, Shin DB, et al. Ramosetron for the prevention of cisplatin-induced acute emesis: a prospective randomized comparison with granisetron. J Int Med Res. 2002;30(3):220-9.
135. Kasimis BS, Tapazoglou E, Schulman P, Stewart WH, Hahne WF, Cramer MB. A double-blind, randomized study of two different dosage regimens of intravenous dolasetron in patients receiving high-dose cisplatin chemotherapy. Cancer Invest. 1997;15(4):304-10.
136. Kaushal J, Gupta MC, Kaushal V, Bhutani G, Dhankar R, Atri R, et al. Clinical evaluation of two antiemetic combinations palonosetron dexamethasone versus ondansetron dexamethasone in chemotherapy of head and neck cancer. Singapore Med J. 2010;51(11):871-5.
137. Keyhanian S, Taziki O, Saravi MM, Fotokian Z. A randomized comparison of granisetron plus dexamethason with granisetron alone for the control of acute chemotherapy-induced emesis and nausea. International Journal of Hematology-Oncology and Stem Cell Research. 2009;3(2):27-30.
138. Khojasteh A, Sartiano G, Tapazoglou E, Lester E, Gandara D, Bernard S, et al. Ondansetron for the prevention of emesis induced by high-dose cisplatin. A multi-center dose-response study. Cancer. 1990;66(6):1101-5.

139. Kigawa J, Minagawa Y, Itamochi H, Cheng X, Okada M, Terakawa N. Combination effect of granisetron and methylprednisolone for preventing emesis induced by cytotoxic agents. Gynecol Obstet Invest. 1997;43(3):195-9.
140. Kim JS, Baek JY, Park SR, Choi IS, Kim SI, Kim DW, et al. Open-label, randomized comparison of the efficacy of intravenous dolasetron mesylate and ondansetron in the prevention of acute and delayed cisplatin-induced emesis in cancer patients. Cancer Res Treat. 2004;36(6):372-6.
141. Kleisbauer JP, Garcia-Giron C, Antimi M, Azevedo MC, Balmes H, Massuti-Sureda B, et al. Granisetron plus methylprednisolone for the control of high-dose cisplatin-induced emesis. Anticancer Drugs. 1998;9(5):387-92.
142. Koenig HJ, Drechsler S, Kohnen R, Faerber L. Acceptance and Tolerability of Tropisetron and Metoclopramide in Outpatients – a Randomized Crossover Study. Onkologie. 2000;23(3):258-61.
143. Koizumi W, Tanabe S, Nagaba S, Higuchi K, Nakayama N, Saigenji K, et al. A double-blind, crossover, randomized comparison of granisetron and ramosetron for the prevention of acute and delayed cisplatin-induced emesis in patients with gastrointestinal cancer: is patient preference a better primary endpoint? Chemotherapy. 2003;49(6):316-23.
144. Komada Y, Matsuyama T, Takao A, Hongo T, Nishimura Y, Horibe K, et al. A randomised dose-comparison trial of granisetron in preventing emesis in children with leukaemia receiving emetogenic chemotherapy. European journal of cancer. 1999;35(7):1095-101.
145. Koralewski P, Karczmarek-Borowska B, Cegielski W, Nawara I, Urbanska-Gasiorowska M. Effectiveness of oral ondansetron in the management of nausea and vomiting induced by moderately emetogenic chemotherapy. Nowotwory. 2001;51(6):579-83.
146. Kris MG, Gralla RJ, Clark RA, Tyson LB. Dose-ranging evaluation of the serotonin antagonist GR-C507/75 (GR38032F) when used as an antiemetic in patients receiving anticancer chemotherapy. J Clin Oncol. 1988;6(4):659-62.
147. Kris MG, Grunberg SM, Gralla RJ, Baltzer L, Zaretsky SA, Lifsey D, et al. Dose-ranging evaluation of the serotonin antagonist dolasetron mesylate in patients receiving high-dose cisplatin. J Clin Oncol. 1994;12(5):1045-9.
148. Kris MG, Pendergrass KB, Navari RM, Grote TH, Nelson AM, Thomas V, et al. Prevention of acute emesis in cancer patients following high-dose cisplatin with the combination of oral dolasetron and dexamethasone. J Clin Oncol. 1997;15(5):2135-8.
149. Krzakowski M, Graham E, Goedhals L, Joly F, Pawlicki M, Rapoport B, et al. A multicenter, double-blind comparison of i.v. and oral administration of ondansetron plus dexamethasone for acute cisplatin-induced emesis. Ondansetron Acute Emesis Study Group. Anticancer Drugs. 1998;9(7):593-8.
150. Kurnianda J, Hisyam B, Wahyuningsih E, Hutajulu SH. The efficacy of granisetron for cancer patients undergoing platinum-based chemotherapy: comparison of 1 miligram versus 3 miligram doses in preventing nausea and vomiting. Acta Med Indones. 2005;37(4):210-3.
151. Labar B, Mrsic M, Nemet D, Francetic I, Bogdanic V, Radman I, et al. Ondansetron for prophylaxis of nausea and vomiting after bone marrow transplantation. Libri Oncologici (Croatina Journal of Oncology). 1995;24(3):131-5.
152. Latreille J, Pater J, Johnston D, Laberge F, Stewart D, Rusthoven J, et al. Use of dexamethasone and granisetron in the control of delayed emesis for patients who receive highly emetogenic chemotherapy. National Cancer Institute of Canada Clinical Trials Group. J Clin Oncol. 1998;16(3):1174-8.
153. Latreille J, Stewart D, Laberge F, Hoskins P, Rusthoven J, McMurtrie E, et al. Dexamethasone improves the efficacy of granisetron in the first 24 h following high-dose cisplatin chemotherapy. Support Care Cancer. 1995;3(5):307-12.
154. Lazarus HM, O'Riordan MA, Creger RJ, Myers CM, Blumer JL, Reed MD. Pharmacokinetics, tolerability and efficacy of bolus-dose vs continuous-infusion granisetron in the prevention of vomiting in patients undergoing haematopoietic stem-cell transplantation: A randomised, double-blind evaluation. Clinical Drug Investigation. 1999;17(2):155-65.
155. Lemerle J, Amaral D, Southall DP, Upward J, Murdoch RD. Efficacy and safety of granisetron in the prevention of chemotherapy-induced emesis in paediatric patients. European journal of cancer. 1991;27(9):1081-3.
156. Leonardi V, Iannito E, Meli M, Palmeri S. Ondansetron (OND) vs granisetron (GRA) in the control of chemotherapy induced acute emesis. Oncol Rep. 1996;3(5):919-23.

157. Levitt M, Warr D, Yelle L, Rayner HL, Lofters WS, Perrault DJ, et al. Ondansetron compared with dexamethasone and metoclopramide as antiemetics in the chemotherapy of breast cancer with cyclophosphamide, methotrexate, and fluorouracil. N Engl J Med. 1993;328(15):1081-4.
158. Liaw CC, Wang CH, Chang HK, Liau CT, Yeh KY, Huang JS, et al. Gender discrepancy observed between chemotherapy-induced emesis and hiccups. Support Care Cancer. 2001;9(6):435-41.
159. Lim AK, Haron MR, Yap TM. Ondansetron against metoclopramide/dexamethasone--a comparative study. Med J Malaysia. 1994;49(3):231-8.
160. Lindley C, Goodin S, McCune J, Kane M, Amamoo MA, Shord S, et al. Prevention of delayed chemotherapy-induced nausea and vomiting after moderately high to highly emetogenic chemotherapy: comparison of ondansetron, prochlorperazine, and dexamethasone. Am J Clin Oncol. 2005;28(3):270-6.
161. Lofters WS, Pater JL, Zee B, Dempsey E, Walde D, Moquin JP, et al. Phase III double-blind comparison of dolasetron mesylate and ondansetron and an evaluation of the additive role of dexamethasone in the prevention of acute and delayed nausea and vomiting due to moderately emetogenic chemotherapy. J Clin Oncol. 1997;15(8):2966-73.
162. Madej G, Krzakowski M, Pawiriski A, Lasota W, Rogowski W, Skoneczna I. A Comparative Study of the Use of Navoban (ICS 205-930), a 5-HT3 Antagonist, versus a Standard Antiemetic Regimen of Dexamethasone and Metoclopramide in the Treatment of Cisplatin-Containing Chemotherapy. Drug Investigation. 1993;6(3):162-9.
163. Maemondo M, Masuda N, Sekine I, Kubota K, Segawa Y, Shibuya M, et al. A phase II study of palonosetron combined with dexamethasone to prevent nausea and vomiting induced by highly emetogenic chemotherapy. Ann Oncol. 2009;20(11):1860-6.
164. Mandanas RA, Beveridge R, Rifkin RM, Wallace H, Greenspan A, Asmar L. A randomized, multicenter, open-label comparison of the antiemetic efficacy of dolasetron versus ondansetron for the prevention of nausea and vomiting during high-dose myeloablative chemotherapy. Support Cancer Ther. 2005;2(2):114-21.
165. Mantovani G, Maccio A, Bianchi A, Curreli L, Ghiani M, Proto E, et al. Comparison of granisetron, ondansetron, and tropisetron in the prophylaxis of acute nausea and vomiting induced by cisplatin for the treatment of head and neck cancer: a randomized controlled trial. Cancer. 1996;77(5):941-8.
166. Marschner NW, Adler M, Nagel GA, Christmann D, Fenzl E, Upadhyaya B. Double-blind randomised trial of the antiemetic efficacy and safety of ondansetron and metoclopramide in advanced breast cancer patients treated with epirubicin and cyclophosphamide. European journal of cancer. 1991;27(9):1137-40.
167. Martoni A, Angelelli B, Guaraldi M, Strocchi E, Pannuti F. An open randomised cross-over study on granisetron versus ondansetron in the prevention of acute emesis induced by moderate dose cisplatin-containing regimens. European journal of cancer. 1996;32A(1):82-5.
168. Marty M. A comparative study of the use of granisetron, a selective 5-HT3 antagonist, versus a standard anti-emetic regimen of chlorpromazine plus dexamethasone in the treatment of cytostatic-induced emesis. The Granisetron Study Group. European journal of cancer. 1990;26 Suppl 1:S28-32.
169. Marty M, Kleisbauer JP, Fournel P, Vergnenegre A, Carles P, Loria-Kanza Y, et al. Is Navoban (tropisetron) as effective as Zofran (ondansetron) in cisplatin-induced emesis? The French Navoban Study Group. Anticancer Drugs. 1995;6 Suppl 1:15-21.
170. Marty M, Pouillart P, Scholl S, Droz JP, Azab M, Brion N, et al. Comparison of the 5-hydroxytryptamine3 (serotonin) antagonist ondansetron (GR 38032F) with high-dose metoclopramide in the control of cisplatin-induced emesis. N Engl J Med. 1990;322(12):816-21.
171. Massidda B, Ionta MT. Prevention of delayed emesis by a single intravenous bolus dose of 5-HT3-receptor-antagonist in moderately emetogenic chemotherapy. J Chemother. 1996;8(3):237-42.
172. Matsui K, Fukuoka M, Takada M, Kusunoki Y, Yana T, Tamura K, et al. Randomised trial for the prevention of delayed emesis in patients receiving high-dose cisplatin. Br J Cancer. 1996;73(2):217-21.
173. Matsuoka S, Okamoto S, Watanabe R, Mori T, Nagayama H, Hamano Y, et al. Granisetron plus dexamethasone versus granisetron alone in the prevention of vomiting induced by conditioning for stem cell transplantation: a prospective randomized study. Int J Hematol. 2003;77(1):86-90.
174. Mattiuzzi GN, Cortes JE, Blamble DA, Bekele BN, Xiao L, Cabanillas M, et al. Daily palonosetron is superior to ondansetron in the prevention of delayed chemotherapy-induced nausea and vomiting in patients with acute myelogenous leukemia. Cancer. 2010;116(24):5659-66.

175. Merrouche Y, Catimel G, Rebattu P, Dumortier A, Guastalla JP, O'Grady P, et al. A phase I antiemetic study of MDL 73,147EF, a novel 5-hydroxytryptamine antagonist in cancer patients receiving emetogenic chemotherapy. Ann Oncol. 1994;5(6):549-51.
176. Mustacchi G, Ceccherini R, Leita ML, Sandri P, Milani S, Carbonara T. The combination of metoclopramide, methylprednisolone and ondansetron against antiblastic-delayed emesis: a randomised phase II study. Anticancer Res. 1997;17(2B):1345-8.
177. Nagel K, Willan AR, Lappan J, Korz L, Buckley N, Barr RD. Pediatric oncology sedation trial (POST): A double-blind randomized study. Pediatric Blood and Cancer. 2008;51(5):634-8.
178. Navari R, Gandara D, Hesketh P, Hall S, Mailliard J, Ritter H, et al. Comparative clinical trial of granisetron and ondansetron in the prophylaxis of cisplatin-induced emesis. The Granisetron Study Group. J Clin Oncol. 1995;13(5):1242-8.
179. Navari RM, Kaplan HG, Gralla RJ, Grunberg SM, Palmer R, Fitts D. Efficacy and safety of granisetron, a selective 5-hydroxytryptamine-3 receptor antagonist, in the prevention of nausea and vomiting induced by high-dose cisplatin. J Clin Oncol. 1994;12(10):2204-10.
180. Navari RM, Madajewicz S, Anderson N, Tchekmedyian NS, Whaley W, Garewal H, et al. Oral ondansetron for the control of cisplatin-induced delayed emesis: a large, multicenter, double-blind, randomized comparative trial of ondansetron versus placebo. J Clin Oncol. 1995;13(9):2408-16.
181. Needles B, Miranda E, Garcia Rodriguez FM, Diaz LB, Spector J, Craig J, et al. A multicenter, double-blind, randomized comparison of oral ondansetron 8 mg b.i.d., 24 mg q.d., and 32 mg q.d. in the prevention of nausea and vomiting associated with highly emetogenic chemotherapy. S3AA3012 Study Group. Support Care Cancer. 1999;7(5):347-53.
182. Noble A, Bremer K, Goedhals L, Cupissol D, Dilly SG. A double-blind, randomised, crossover comparison of granisetron and ondansetron in 5-day fractionated chemotherapy: assessment of efficacy, safety and patient preference. The Granisetron Study Group. European journal of cancer. 1994;30A(8):1083-8.
183. Noda K, Ikeda M, Taguchi T, Kanamaru R, Ichijyo M, Okada K, et al. Clinical assessment of ramosetron HCl oral preparation in the treatment of nausea and vomiting induced by cisplatin: A multicenter, randomized, parallel-design, double-blind comparative study with ondansetron HCl. Current therapeutic research, clinical and experimental. 2002;63(10):636-48.
184. Noda K, Ikeda M, Yoshida O, Yano S, Taguchi T, Shimoyama T, et al. Clinical evaluation of Ramosetron injections in the treatment of cisplatin-induced nausea and vomiting. J Int Med Res. 2002;30(3):211-9.
185. Noor R, Bedikian AY, Mahoney S, Bassett R, Jr., Kim K, Papadopoulos N, et al. Comparison of two dosing schedules of palonosetron for the prevention of nausea and vomiting due to interleukin-2-based biochemotherapy. Support Care Cancer. 2012;20(10):2583-8.
186. Oge A, Alkis N, Oge O, Kartum A. Comparison of granisetron, ondansetron and tropisetron for control of vomiting and nausea induced by cisplatin. J Chemother. 2000;12(1):105-8.
187. Olver I, Paska W, Depierre A, Seitz JF, Stewart DJ, Goedhals L, et al. A multicentre, double-blind study comparing placebo, ondansetron and ondansetron plus dexamethasone for the control of cisplatin-induced delayed emesis. Ondansetron Delayed Emesis Study Group. Ann Oncol. 1996;7(9):945-52.
188. Orchard PJ, Rogosheske J, Burns L, Rydholm N, Larson H, DeFor TE, et al. A prospective randomized trial of the anti-emetic efficacy of ondansetron and granisetron during bone marrow transplantation. Biol Blood Marrow Transplant. 1999;5(6):386-93.
189. Ozgur Tan M, Sandikci Z, Uygur MC, Arik AI, Erol D. Combination of transcutaneous electrical nerve stimulation and ondansetron in preventing cisplatin-induced emesis. Urol Int. 2001;67(1):54-8.
190. Park JO, Rha SY, Yoo NC, Kim JH, Roh JK, Min JS, et al. A comparative study of intravenous granisetron versus intravenous and oral ondansetron in the prevention of nausea and vomiting associated with moderately emetogenic chemotherapy. Am J Clin Oncol. 1997;20(6):569-72.
191. Parker RI, Prakash D, Mahan RA, Giugliano DM, Atlas MP. Randomized, double-blind, crossover, placebo-controlled trial of intravenous ondansetron for the prevention of intrathecal chemotherapy-induced vomiting in children. J Pediatr Hematol Oncol. 2001;23(9):578-81.
192. Pectasides D, Dafni U, Aravantinos G, Timotheadou E, Skarlos DV, Pavlidis N, et al. A randomized trial to compare the efficacy and safety of antiemetic treatment with ondansetron and ondansetron zydis in patients with breast cancer treated with high-dose epirubicin. Anticancer Res. 2007;27(6C):4411-7.

193. Pectasides D, Mylonakis A, Varthalitis J, Kostopoulou M, Constantinou A, Papazachariou K, et al. Comparison of two different doses of ondansetron plus dexamethasone in the prophylaxis of cisplatin-induced emesis. Oncology. 1997;54(1):1-6.
194. Perez EA, Hesketh P, Sandbach J, Reeves J, Chawla S, Markman M, et al. Comparison of single-dose oral granisetron versus intravenous ondansetron in the prevention of nausea and vomiting induced by moderately emetogenic chemotherapy: a multicenter, double-blind, randomized parallel study. J Clin Oncol. 1998;16(2):754-60.
195. Perez EA, Lembersky B, Kaywin P, Kalman L, Yocom K, Friedman C. Comparable safety and antiemetic efficacy of a brief (30-second bolus) intravenous granisetron infusion and a standard (15-minute) intravenous ondansetron infusion in breast cancer patients receiving moderately emetogenic chemotherapy. Cancer J Sci Am. 1998;4(1):52-8.
196. Perez EA, Navari RM, Kaplan HG, Gralla RJ, Grunberg SM, Palmer RH, et al. Efficacy and safety of different doses of granisetron for the prophylaxis of cisplatin-induced emesis. Support Care Cancer. 1997;5(1):31-7.
197. Peterson C, Hursti TJ, Borjeson S, Avall-Lundqvist E, Fredrikson M, Furst CJ, et al. Single high-dose dexamethasone improves the effect of ondansetron on acute chemotherapy-induced nausea and vomiting but impairs the control of delayed symptoms. Support Care Cancer. 1996;4(6):440-6.
198. Pielichowski W, Gawronski K, Mlot B, Oborska S, Wasko-Grabowska A, Rzepecki P. Triple drug combination in the prevention of nausea and vomiting following busulfan plus cyclophosphamide chemotherapy before allogeneic hematopoietic stem cell transplantation. Journal of BUOn. 2011;16(3):541-6.
199. Pinarli FG, Elli M, Dagdemir A, Baysal K, Acar S. Electrocardiographic findings after 5-HT3 receptor antagonists and chemotherapy in children with cancer. Pediatric Blood and Cancer. 2006;47(5):567-71.
200. Pisters KM, Kris MG, Tyson LB, Clark RA, Gralla RJ. Dose-ranging antiemetic evaluation of the serotonin antagonist tropisetron in patients receiving anti-cancer chemotherapy. Cancer. 1993;71(1):226-30.
201. Poon RT, Chow LW. Comparison of antiemetic efficacy of granisetron and ondansetron in Oriental patients: a randomized crossover study. Br J Cancer. 1998;77(10):1683-5.
202. Rath U, Upadhyaya BK, Arechavala E, Bockmann H, Dearnaley D, Droz JP, et al. Role of ondansetron plus dexamethasone in fractionated chemotherapy. Oncology. 1993;50(3):168-72.
203. Raynov J, Danon S, Valerianova Z. Control of acute emesis in repeated courses of moderately emetogenic chemotherapy. J BUON. 2002;7(1):57-60.
204. Raynov J, Raynova P, Kancheva T, Georgiev G. Antiemetic control in cancer patients treated with highly emetogenic chemotherapy. Journal of B U ON. 2000;5(3):287-91.
205. Raynov J, Stanchev R, Radinov A, Popova T, Damjanov I. Efficacy of different doses of ondansetron in cancer patients treated with cisplatin-containing chemotherapy. Archive of Oncology. 1999;7(4):149-51.
206. Research TIGfA. Dexamethasone, granisetron, or both for the prevention of nausea and vomiting during chemotherapy for cancer. The Italian Group for Antiemetic Research. N Engl J Med. 1995;332(1):1-5.
207. Ribiero M, de Faria L, dos Reis F, Fráguas A, de Matos E, Uva S, et al. Ondansetron in the treatment of nausea and vomiting induced by chemotherapy. Anti-Cancer Drugs. 1993;4(3):23-7.
208. Ritter HL, Jr., Gralla RJ, Hall SW, Wada JK, Friedman C, Hand L, et al. Efficacy of intravenous granisetron to control nausea and vomiting during multiple cycles of cisplatin-based chemotherapy. Cancer Invest. 1998;16(2):87-93.
209. Riviere A. Dose finding study of granisetron in patients receiving high-dose cisplatin chemotherapy. The Granisetron Study Group. Br J Cancer. 1994;69(5):967-71.
210. Roila EB, E.; Ruggeri, B.; Angelis, V.; Tonato, M.; Bosnjak, S.; Favero, A.; Ciccarese, G.; Basurto, C.; Ciccarese, G.; Palladino, M. A.; Porrozzi, S.; Marinkovic, Z.; Neskovic-Konstantinovic, Z.; Susnjar, S.; Vasovic, S.; Colakovic, S.; Lukic, V.; Radosavljevic, D.; Fava, S.; Grimi, E.; Calcagno, A.; Paoli, A.; Luoni, M.; Tocci, A.; Nuzzo, A.; Laudadio, L.; Blasio, A.; Sacco, M.; Contu, A.; Olmeo, N.; Pazzola, A.; Baldino, G.; Picece, V.; Nicodemo, M.; Cirillo, M.; Recaldin, E.; Daz. Dexamethasone alone or in combination with ondansetron for the prevention of delayed nausea and vomiting induced by chemotherapy. The Italian Group for Antiemetic Research. N Engl J Med. 2000;342(21):1554-9.

211. Roila F. Ondansetron versus metoclopramide, both combined with dexamethasone, in the prevention of cisplatin-induced delayed emesis. The Italian Group for Antiemetic Research. J Clin Oncol. 1997;15(1):124-30.
212. Roila F, Tonato M, Cognetti F, Cortesi E, Favalli G, Marangolo M, et al. Prevention of cisplatin-induced emesis: a double-blind multicenter randomized crossover study comparing ondansetron and ondansetron plus dexamethasone. J Clin Oncol. 1991;9(4):675-8.
213. Roscoe JA, Heckler CE, Morrow GR, Mohile SG, Dakhil SR, Wade JL, et al. Prevention of delayed nausea: a University of Rochester Cancer Center Community Clinical Oncology Program study of patients receiving chemotherapy. J Clin Oncol. 2012;30(27):3389-95.
214. Rubenstein EB, Gralla RJ, Hainsworth JD, Hesketh PJ, Grote TH, Modiano MR, et al. Randomized, double blind, dose-response trial across four oral doses of dolasetron for the prevention of acute emesis after moderately emetogenic chemotherapy. Oral Dolasetron Dose-Response Study Group. Cancer. 1997;79(6):1216-24.
215. Ruff P, Paska W, Goedhals L, Pouillart P, Riviere A, Vorobiof D, et al. Ondansetron compared with granisetron in the prophylaxis of cisplatin-induced acute emesis: a multicentre double-blind, randomised, parallel-group study. The Ondansetron and Granisetron Emesis Study Group. Oncology. 1994;51(1):113-8.
216. Rzepecki P, Pielichowski W, Oborska S, Barzal J, Mlot B. Palonosetron in prevention of nausea and vomiting after highly emetogenic chemotherapy before haematopoietic stem cell transplantation-single center experience. Transplant Proc. 2009;41(8):3247-9.
217. Sagae S, Ishioka S, Fukunaka N, Terasawa K, Kobayashi K, Sugimura M, et al. Combination therapy with granisetron, methylprednisolone and droperidol as an antiemetic prophylaxis in CDDP-induced delayed emesis for gynecologic cancer. Oncology. 2003;64(1):46-53.
218. Saito M, Aogi K, Sekine I, Yoshizawa H, Yanagita Y, Sakai H, et al. Palonosetron plus dexamethasone versus granisetron plus dexamethasone for prevention of nausea and vomiting during chemotherapy: a double-blind, double-dummy, randomised, comparative phase III trial. Lancet Oncol. 2009;10(2):115-24.
219. Sandoval C, Corbi D, Strobino B, Fevzi Ozkaynak M, Tugal O, Jayabose S. Randomized double-blind comparison of single high-dose ondansetron and multiple standard-dose ondansetron in chemotherapy-naive pediatric oncology patients. Cancer Invest. 1999;17(5):309-13.
220. Scoponi CAT, U.; Di Giuseppe, M.; Giustozzi, M. Are 5-HT3 antagonists a standard antiemetic treatment also in slightly and moderately emetogenic regimens? Oncologia. 1998;21(9):40-4.
221. Segawa Y, Aogi K, Inoue K, Sano M, Sekine I, Tokuda Y, et al. A phase II dose-ranging study of palonosetron in Japanese patients receiving moderately emetogenic chemotherapy, including anthracycline and cyclophosphamide-based chemotherapy. Ann Oncol. 2009;20(11):1874-80.
222. Sepulveda-Vildosola AC, Betanzos-Cabrera Y, Lastiri GG, Rivera-Marquez H, Villasis-Keever MA, Del Angel VW, et al. Palonosetron hydrochloride is an effective and safe option to prevent chemotherapy-induced nausea and vomiting in children. Arch Med Res. 2008;39(6):601-6.
223. Seynaeve C, de Mulder PH, Lane-Allman E, van Liessum PA, Verweij J. The 5-HT3 receptor antagonist ondansetron re-establishes control in refractory emesis induced by non-cisplatin chemotherapy. Clin Oncol (R Coll Radiol). 1991;3(4):199-203.
224. Seynaeve C, Schuller J, Buser K, Porteder H, Van Belle S, Sevelda P, et al. Comparison of the anti-emetic efficacy of different doses of ondansetron, given as either a continuous infusion or a single intravenous dose, in acute cisplatin-induced emesis. A multicentre, double-blind, randomised, parallel group study. Ondansetron Study Group. Br J Cancer. 1992;66(1):192-7.
225. Shi Y, He X, Yang S, Ai B, Zhang C, Huang D, et al. Ramosetron versus ondansetron in the prevention of chemotherapy-induced gastrointestinal side effects: A prospective randomized controlled study. Chemotherapy. 2007;53(1):44-50.
226. Shikimori M, Komatsu H, Hashimoto K, Yamashita Y. Granisetron reduces clinical and histological response to intra-arterial neoadjuvant chemotherapy in oral squamous cell carcinoma. Int J Oral Maxillofac Surg. 2006;35(9):800-2.
227. Siddique R, Hafiz MG, Rokeya B, Jamal CY, Islam A. Ondansetron versus granisetron in the prevention of chemotherapy induced nausea and vomiting in children with acute lymphoblastic leukemia. Mymensingh Med J. 2011;20(4):680-8.

228. Sigsgaard T, Herrstedt J, Andersen LJ, Havsteen H, Langer SW, Kjaerbol AG, et al. Granisetron compared with prednisolone plus metopimazine as anti-emetic prophylaxis during multiple cycles of moderately emetogenic chemotherapy. Br J Cancer. 1999;80(3-4):412-8.
229. Slaby J, Trneny M, Prochazka B, Klener P. Antiemetic efficacy of three serotonin antagonists during high-dose chemotherapy and autologous stem cell transplantation in malignant lymphoma. Neoplasma. 2000;47(5):319-22.
230. Sledge GW, Jr., Einhorn L, Nagy C, House K. Phase III double-blind comparison of intravenous ondansetron and metoclopramide as antiemetic therapy for patients receiving multiple-day cisplatin-based chemotherapy. Cancer. 1992;70(10):2524-8.
231. Smith DB, Newlands ES, Rustin GJ, Begent RH, Howells N, McQuade B, et al. Comparison of ondansetron and ondansetron plus dexamethasone as antiemetic prophylaxis during cisplatin-containing chemotherapy. Lancet. 1991;338(8765):487-90.
232. Smith IE. A dose-finding study of granisetron, a novel antiemetic, in patients receiving cytostatic chemotherapy. The Granisetron Study Group. J Cancer Res Clin Oncol. 1993;119(6):350-4.
233. Smyth JF, Coleman RE, Nicolson M, Gallmeier WM, Leonard RC, Cornbleet MA, et al. Does dexamethasone enhance control of acute cisplatin induced emesis by ondansetron? BMJ. 1991;303(6815):1423-6.
234. Sorbe B, Hogberg T, Himmelmann A, Schmidt M, Raisanen I, Stockmeyer M, et al. Efficacy and tolerability of tropisetron in comparison with a combination of tropisetron and dexamethasone in the control of nausea and vomiting induced by cisplatin-containing chemotherapy. European journal of cancer. 1994;30A(5):629-34.
235. Sorbe BG, Berglind AM, Andersson H, Boman K, Hogberg T, Hallgren M, et al. A study evaluating the efficacy and tolerability of tropisetron in combination with dexamethasone in the prevention of delayed platinum-induced nausea and emesis. Cancer. 1998;83(5):1022-32.
236. Soukop M, McQuade B, Hunter E, Stewart A, Kaye S, Cassidy J, et al. Ondansetron compared with metoclopramide in the control of emesis and quality of life during repeated chemotherapy for breast cancer. Oncology. 1992;49(4):295-304.
237. Spector JI, Lester EP, Chevlen EM, Sciortino D, Harvey JH, Whaley W, et al. A Comparison of Oral Ondansetron and Intravenous Granisetron for the Prevention of Nausea and Emesis Associated with Cisplatin-Based Chemotherapy. Oncologist. 1998;3(6):432-8.
238. Steiner MA, Yorgason RZ, Vermeulen LC, Theisen J. Patient outcomes after therapeutic interchange of dolasetron for granisetron. Am J Health Syst Pharm. 2003;60(10):1023-8.
239. Stewart A, McQuade B, Cronje JD, Goedhals L, Gudgeon A, Corette L, et al. Ondansetron compared with granisetron in the prophylaxis of cyclophosphamide-induced emesis in out-patients: a multicentre, double-blind, double-dummy, randomised, parallel-group study. Emesis Study Group for Ondansetron and Granisetron in Breast Cancer Patients. Oncology. 1995;52(3):202-10.
240. Stewart L, Crawford SM, Taylor PA. The comparative effectiveness of ondansetron and granisetron in a once daily dosage in the prevention of nausea and vomiting caused by cisplatin: A double-blind clinical trial. Pharmaceutical Journal. 2000;265(7104):59-62.
241. Stiakaki E, Savvas S, Lydaki E, Bolonaki I, Kouvidi E, Dimitriou H, et al. Ondansetron and tropisetron in the control of nausea and vomiting in children receiving combined cancer chemotherapy. Pediatr Hematol Oncol. 1999;16(2):101-8.
242. Suarez A, Stettler ER, Rey E, Pons G, Simonetta-Chateauneuf C, de Bruijn KM, et al. Safety, tolerability, efficacy and plasma concentrations of tropisetron after administration at five dose levels to children receiving cancer chemotherapy. European journal of cancer. 1994;30A(10):1436-41.
243. Taguchi K, Iihara H, Ishihara M, Komori Y, Tanizawa K, Matsuura K, et al. Comparison of antiemetic efficacy between single and repeated treatments with a 5-HT3 receptor antagonist in breast cancer patients with high-risk emetogenic chemotherapy. Anticancer Res. 2009;29(5):1721-5.
244. Tan M, Xu R, Seth R. Granisetron vs dolasetron for acute chemotherapy-induced nausea and vomiting (CINV) in high and moderately high emetogenic chemotherapy: an open-label pilot study. Curr Med Res Opin. 2004;20(6):879-82.
245. Tantipalakorn C, Srisomboon J, Thienthong H, Pantusart A, Suprasert P, Saereesongkhun C, et al. Comparison of oral versus intravenous ramosetron in prevention of acute cisplatin-induced emesis: a randomized controlled trial. J Med Assoc Thai. 2004;87(2):119-25.
246. Tatsumi N, Masaoka T. A comparative study of administration methods of granisetron injection used to treat nausea/vomiting induced by cancer chemotherapy without cisplatin in tumors of hematopoietic

organs. Keihanshin Study Group of Hematological Malignancies. Cancer Chemother Pharmacol. 1999;43(5):379-84.
247. Tian W, Wang Z, Zhou J, Zhang S, Wang J, Chen Q, et al. Randomized, double-blind, crossover study of palonosetron compared with granisetron for the prevention of chemotherapy-induced nausea and vomiting in a Chinese population. Med Oncol. 2011;28(1):71-8.
248. Tsavaris N, Charalambidis G, Ganas N, Pagou M, Karabellis A, Mylonakis N, et al. Ondansetron versus metoclopramide as antiemetic treatment during cisplatin-based chemotherapy. A prospective study with special regard to regard to electrolyte imbalance. Acta Oncol. 1995;34(2):243-6.
249. Tsavaris N, Fountzilas G, Mylonakis N, Athanassiadis A, Kosmas C, Karakousis C, et al. A randomized comparative study of antiemetic prophylaxis with ondansentron in a single 32-mg loading dose versus 8 mg every 6 h in patients undergoing cisplatin-based chemotherapy. Oncology. 1998;55(6):513-6.
250. Tsavaris N, Kosmas C, Vadiaka M, Kanelopoulos P, Kontos A, Katsorida M, et al. Ondansentron and dexamethasone treatment with different dosing schedules (24 versus 32 mg) in patients with non-small-cell lung cancer under cisplatin-based chemotherapy: a randomized study. Chemotherapy. 2000;46(5):364-70.
251. Tsavaris N, Kosmas CH, Vadiaka M, Kontos A, Katsorida M, Dimitrakopoulos A, et al. Efficacy of ondansentron treatment for acute emesis with different dosing schedules 8 vs 32 mg. A randomized study. J Exp Clin Cancer Res. 2001;20(1):29-34.
252. Tsavaris N, Mylonakis N, Bacoyiannis C, Karvounis N, Charalambidis G, Pagou M, et al. Efficacy of ondansetron treatment with different timing schedules: a randomized double-blind study. Oncology. 1995;52(4):315-8.
253. Tsavaris NB, Koufos C, Katsikas M, Dimitrakopoulos A, Athanasiou E, Linardaki G. Antiemetic prophylaxis with ondansetron and methylprednisolone vs metoclopramide and methylprednisolone in mild and moderately emetogenic chemotherapy. J Pain Symptom Manage. 1999;18(3):218-22.
254. Tsavaris NB, Tsaroucha-Noutsou E, Karvounis N, Bacoyannis C, Pagou M, Kosmidis P. Comparison of different schedules of ondansetron (GR 38032F) administration during cisplatin-based chemotherapy: a randomized trial. Chemotherapy. 1995;41(1):70-6.
255. Tsuchida Y, Hayashi Y, Asami K, Yamamoto K, Yokoyama J, Mugishima H, et al. Effects of granisetron in children undergoing high-dose chemotherapy: a multi-institutional, cross-over study. Int J Oncol. 1999;14(4):673-9.
256. Tsuji D, Kim YI, Taku K, Nakagaki S, Ikematsu Y, Tsubota H, et al. Comparative trial of two intravenous doses of granisetron (1 versus 3 mg) in the prevention of chemotherapy-induced acute emesis: a double-blind, randomized, non-inferiority trial. Support Care Cancer. 2012;20(5):1057-64.
257. Tyson LB, Kris MG, Baltzer L, Pisters KM, Rigas JR, Clark RA. Randomized phase II trial comparing two versus three doses of ondansetron when used in combination with dexamethasone in patients receiving cisplatin > or = 100 mg/m2. Am J Clin Oncol. 1994;17(3):269-72.
258. Uchida K, Akaza H, Hattori K, Noguchi R, Kondo F, Ishikawa S, et al. Antiemetic efficacy of granisetron: a randomized crossover study in patients receiving cisplatin-containing intraarterial chemotherapy. Jpn J Clin Oncol. 1999;29(2):87-91.
259. Van Belle SJ, Stamatakis L, Bleiberg H, Cocquyt VF, Michel J, de Bruijn KM. Dose-finding study of tropisetron in cisplatin-induced nausea and vomiting. Ann Oncol. 1994;5(9):821-5.
260. Veroniki AA, Del Giovane C, Blondal E, Thavorn K, Straus SE, Tricco A. How to Model Different Dose-effects in Networks of Interventions? 2015 Canadian Agency for Drugs and Technologies in Health (CADTH) Symposium; Saskatoon, Saskatchewan.
261. Villalon A, Chan V. Multicenter, randomized trial of ramosetron plus dexamethasone versus ramosetron alone in controlling cisplatin-induced emesis. Support Care Cancer. 2004;12(1):58-63.
262. Warr D, Willan A, Fine S, Wilson K, Davis A, Erlichman C, et al. Superiority of granisetron to dexamethasone plus prochlorperazine in the prevention of chemotherapy-induced emesis. J Natl Cancer Inst. 1991;83(16):1169-73.
263. Watters J, Riley M, Pedley I, Whitehead A, Overend M, Goss I, et al. The development of a protocol for the use of 5-HT3 antagonists in chemotherapy-induced nausea and vomiting. Clin Oncol (R Coll Radiol). 2001;13(6):422-6.
264. White L, Daly SA, McKenna CJ, Zhestkova N, Leal C, Breatnach F, et al. A comparison of oral ondansetron syrup or intravenous ondansetron loading dose regimens given in combination with dexamethasone for the prevention of nausea and emesis in pediatric and adolescent patients receiving moderately/highly emetogenic chemotherapy. Pediatr Hematol Oncol. 2000;17(6):445-55.

265. Yalcin S, Tekuzman G, Baltali E, Ozisik Y, Barista I. Serotonin receptor antagonists in prophylaxis of acute and delayed emesis induced by moderately emetogenic, single-day chemotherapy: a randomized study. Am J Clin Oncol. 1999;22(1):94-6.
266. Yamac DC, G.; Coskun, U.; Abubakar, A.; Gunel, N. A comperative study of the efficacy, cost and adverse effects of the third generation antiemetic agents in cancer patients. Turk Hematoloji-Onkoloji Dergisi. 2002;12(1):12-5.
267. Yano S, Makino K, Nakamura H, Kai Y, Morioka M, Hamada J, et al. Comparative clinical study of the anti-emetic effects of oral ramosetron and injected granisetron in patients with malignant glioma undergoing ACNU chemotherapy. Neurol Med Chir (Tokyo). 2005;45(6):294-8; discussion 8-9.
268. Yeilding A, Bertoli L, Eisenberg P, Plezia P, Modiano MR, Alberts DS, et al. Antiemetic efficacy of two different single intravenous doses of dolasetron in patients receiving high-dose cisplatin-containing chemotherapy. Am J Clin Oncol. 1996;19(6):619-23.
269. Yonemura M, Katsumata N, Hashimoto H, Satake S, Kaneko M, Kobayashi Y, et al. Randomized controlled study comparing two doses of intravenous granisetron (1 and 3 mg) for acute chemotherapy-induced nausea and vomiting in cancer patients: a non-inferiority trial. Jpn J Clin Oncol. 2009;39(7):443-8.
270. Yu Z, Liu W, Wang L, Liang H, Huang Y, Si X, et al. The efficacy and safety of palonosetron compared with granisetron in preventing highly emetogenic chemotherapy-induced vomiting in the Chinese cancer patients: a phase II, multicenter, randomized, double-blind, parallel, comparative clinical trial. Support Care Cancer. 2009;17(1):99-102.
271. Zeidman A, Ben Dayan D, Ben Zion T, Kaufman O, Cohen AM, Mittelman M. Granisetron and ondansetron for chemotherapy-related nausea and vomiting. Haematologia (Budap). 1998;29(1):25-31.
272. Zeng XY, Wang AH, Liu YF, Chen Y, Shen Y, Shen ZX. Ramosetron for the management of chemotherapy-induced gastrointestinal events in patients with hematological malignancies. Methods Find Exp Clin Pharmacol. 2001;23(4):191-5.
273. Jorgensen M, Victor MA. Antiemetic efficacy of ondansetron and metoclopramide, both combined with corticosteroid, in malignant lymphoma patients receiving non-cisplatin chemotherapy. Acta Oncol. 1996;35(2):159-63.
274. Keat CH, Phua G, Abdul Kassim MS, Poh WK, Sriraman M. Can granisetron injection used as primary prophylaxis improve the control of nausea and vomiting with low- emetogenic chemotherapy? Asian Pac J Cancer Prev. 2013;14(1):469-73.
275. Huang J, Wang XJ, Yu D, Jin YN, Zhen LZ, Xu N, et al. The effect of palonosetron hydrochloride in the prevention of chemotherapy-induced moderate and severe nausea and vomiting. Exp Ther Med. 2013;5(5):1418-26.
276. Wenzell CM, Berger MJ, Blazer MA, Crawford BS, Griffith NL, Wesolowski R, et al. Pilot study on the efficacy of an ondansetron- versus palonosetron-containing antiemetic regimen prior to highly emetogenic chemotherapy. Support Care Cancer. 2013;21(10):2845-51.
277. Murakami M, Hashimoto H, Yamaguchi K, Yamaguchi I, Senba S, Siraishi T. Effectiveness of palonosetron for preventing delayed chemotherapy-induced nausea and vomiting following moderately emetogenic chemotherapy in patients with gastrointestinal cancer. Support Care Cancer. 2014;22(4):905-9.
278. Kim JS, Kim JY, Lee SJ, Park DK, Namgung H, Kim CN, et al. Multicenter nonrandomized trial of ramosetron versus palonosetron in controlling chemotherapy-induced nausea and vomiting for colorectal cancer. Ann Surg Treat Res. 2014;87(1):9-13.
279. Sanmukhani JJ, Pawar P, Mittal R. Ramosetron hydrochloride for the prevention of cancer chemotherapy induced nausea and vomiting: The Indian experience. South Asian J Cancer. 2014;3(2):132-7.
280. Ohzawa H, Miki A, Hozumi Y, Miyazaki C, Sagara Y, Tanaka Y, et al. Comparison between the antiemetic effects of palonosetron and granisetron in breast cancer patients treated with anthracycline-based regimens. Oncol Lett. 2015;9(1):119-24.
281. Anvari K, Seilanian-Toussi M, Hosseinzad-Ashkiki H, Shahidsales S. A Comparison of 5-HT3 Receptor Antagonist and Metoclopramide in the Patients Receiving Chemotherapeutic Regimens Including CMF, CAF and CHOP. Iran J Cancer Prev. 2015;8(2):84-8.
282. Raftopoulos H, Cooper W, O'Boyle E, Gabrail N, Boccia R, Gralla RJ. Comparison of an extended-release formulation of granisetron (APF530) versus palonosetron for the prevention of chemotherapy-induced nausea and vomiting associated with moderately or highly emetogenic chemotherapy: results of a prospective, randomized, double-blind, noninferiority phase 3 trial. Support Care Cancer. 2015;23(3):723-32.

283. Karthaus M, Tibor C, Lorusso V, Singh-Arora R, Filippov A, Rizzi G, et al. Efficacy and safety of oral palonosetron compared with IV palonosetron administered with dexamethasone for the prevention of chemotherapy-induced nausea and vomiting (CINV) in patients with solid tumors receiving cisplatin-based highly emetogenic chemotherapy (HEC). Support Care Cancer. 2015;23(10):2917-23.
284. Kimura H, Yamamoto N, Shirai T, Nishida H, Hayashi K, Tanzawa Y, et al. Efficacy of triplet regimen antiemetic therapy for chemotherapy-induced nausea and vomiting (CINV) in bone and soft tissue sarcoma patients receiving highly emetogenic chemotherapy, and an efficacy comparison of single-shot palonosetron and consecutive-day granisetron for CINV in a randomized, single-blinded crossover study. Cancer Med. 2015;4(3):333-41.
285. Zoto T, Kilickap S, Yasar U, Celik I, Bozkurt A, Babaoglu MO. Improved anti-emetic efficacy of 5-HT3 receptor antagonists in cancer patients with genetic polymorphisms of ABCB1 (MDR1) drug transporter. Basic Clin Pharmacol Toxicol. 2015;116(4):354-60.
286. Kim KR, Kang G, Ki MS, Shim HJ, Hwang JE, Bae WK, et al. A Randomized, Double-Blind Pilot Study of Dose Comparison of Ramosetron to Prevent Chemotherapy-Induced Nausea and Vomiting. Biomed Res Int. 2015;2015:523601.
287. Kim JE, Hong YS, Lee JL, Kim KP, Park SJ, Sym SJ, et al. A randomized study of the efficacy and safety of transdermal granisetron in the control of nausea and vomiting induced by moderately emetogenic chemotherapy in Korean patients. Support Care Cancer. 2015;23(6):1769-77.
288. Gabrail N, Yanagihara R, Spaczynski M, Cooper W, O'Boyle E, Smith C, et al. Pharmacokinetics, safety, and efficacy of APF530 (extended-release granisetron) in patients receiving moderately or highly emetogenic chemotherapy: results of two Phase II trials. Cancer Manag Res. 2015;7:83-92.
289. Kovacs G, Wachtel AE, Basharova EV, Spinelli T, Nicolas P, Kabickova E. Palonosetron versus ondansetron for prevention of chemotherapy-induced nausea and vomiting in paediatric patients with cancer receiving moderately or highly emetogenic chemotherapy: a randomised, phase 3, double-blind, double-dummy, non-inferiority study. Lancet Oncol. 2016;17(3):332-44.
290. Carreca I BF, Bronte G, Burgio M, Piazza D, Rizzo S. Impact of the new 5-HT3 receptor antagonist palonosetron (P) in chemotherapy-induced nausea and vomiting (CINV) control in elderly advanced breast cancer (EABC) patients: Comparison with the classic antiemetic agent ondansetron (O). In The 2007 Breast Cancer Symposium Proceedings Book. 2007.
291. Kadota R SV, Messinger Y. Safety, pharmacokinetics, and efficacy of palonosetron in pediatric patients: A multicenter, stratified, double-blind, phase 3, randomized study. Journal of Clinical Oncology, 2007 ASCO Annual Meeting Proceedings (Post-Meeting Edition) Vol 25, No 18S (June 20 Supplement),page 9570. 2007.
292. Piyush S. To compare efficacy and cost effectiveness of different 5th 3 blockers in acute and delayed nausea and vomiting: A randomized study. Journal of Cancer Science and Therapy. 2011(3):234.
293. Tabei T, Inoue K, Segawa Y, Sano M, Sekine I, Tokuda Y, et al. A phase II dose-response study of palonosetron (PALO) in Japanese patients receiving moderately emetogenic chemotherapy (MEC)— Palo Japanese Cooperative Study Group. In Annals of Oncology, Vol 17, pp 302-303 2006.
294. Trifilio S, Kaniecki K, Zook J, Pi J, Carson K, Fishman M, et al. Long-Acting Serotonin Antagonist Palonosteron To Prevent Nausea and Vomiting after High-Dose Melphalan and Autograft in Myeloma." In ASH Annual Meeting Abstracts. 2006;108(11):5239.
295. Adel NG KA, Lucarelli C. . Use of palonosetron 0.25 mg IV daily and incidence of nausea and vomiting in patients undergoing bone marrow transplantation (BMT). Journal of Clinical Oncology. 2006;24(18 Suppl): 664S (Abstr 16510).
296. Persistence of efficacy of three antiemetic regimens and prognostic factors in patients undergoing moderately emetogenic chemotherapy. Italian Group for Antiemetic Research. J Clin Oncol. 1995;13(9):2417-26.
297. Delayed emesis induced by moderately emetogenic chemotherapy: do we need to treat all patients? The Italian Group for Antiemetic Research. Ann Oncol. 1997;8(6):561-7.
298. Bhatia A, Tripathi KD, Sharma M. Efficacy & tolerability of ondansetron compared to metoclopramide in dose dependent cisplatin-induced delayed emesis. Indian J Med Res. 2004;120(3):183-93.
299. Chevallier B. Efficacy and safety of granisetron compared with high-dose metoclopramide plus dexamethasone in patients receiving high-dose cisplatin in a single-blind study. The Granisetron Study Group. Eur J Cancer. 1990;26 Suppl 1:S33-6.

300. Dicato MA. Oral treatment with ondansetron in an outpatient setting. Eur J Cancer. 1991;27 Suppl 1:S18-9; discussion S22.
301. Hacking A. Oral granisetron--simple and effective: a preliminary report. The Granisetron Study Group. Eur J Cancer. 1992;28A Suppl 1:S28-32.
302. Hainsworth JD, Hesketh PJ. Single-dose ondansetron for the prevention of cisplatin-induced emesis: efficacy results. Semin Oncol. 1992;19(6 Suppl 15):14-9.
303. Hamadani M, Awab A, Chaudhary L, Tfayli A. Relative efficacy of ondansetron, granisetron, dolasetron and palonosetron in controlling acute nausea and vomiting associated with platinum-based chemotherapy. J Oncol Pharm Pract. 2006;12(2):67-8.
304. Liaw CC, Chang HK, Liau CT, Huang JS, Lin YC, Chen JS. Reduced maintenance of complete protection from emesis for women during chemotherapy cycles. Am J Clin Oncol. 2003;26(1):12-5.
305. Liaw CC, Wang CH, Chang HK, Kao CY, Huang JS. Control of cisplatin-induced emesis with intravenous ondansetron plus intravenous dexamethasone: a crossover study of triple 8-mg dose of ondansetron. Am J Clin Oncol. 2000;23(3):253-7.
306. Madej G, Krzakowski M, Pawinski A, Lasota W, Rogowski W, Skoneczna I. A report comparing the use of tropisetron (Navoban), a 5-HT3 antagonist, with a standard antiemetic regimen of dexamethasone and metoclopramide in cisplatin-treated patients under conditions of severe emesis. Semin Oncol. 1994;21(5 Suppl 9):3-6.
307. Pielichowski W, Gawronski K, Mlot B, Oborska S, Wasko-Grabowska A, P R. Three-drug regimen (aprepitant + palonosetron + dexamethasone) for prevention of nausea and vomiting associated with BEAM chemotherapy preceding transplantation of autologous hematopoietic stem cells. Current Gynecologic Oncology. 2011;9(2):95-103.
308. Pielichowski W, Barzal J, Gawronski K, Mlot B, Oborska S, Wasko-Grabowska A, et al. A triple-drug combination to prevent nausea and vomiting following BEAM chemotherapy before autologous hematopoietic stem cell transplantation. Transplant Proc. 2011;43(8):3107-10.
309. Smith IE. A comparison of two dose levels of granisetron in patients receiving moderately emetogenic cytostatic chemotherapy. The Granisetron Study Group. Eur J Cancer. 1990;26 Suppl 1:S19-23.
310. Sorbe BG, Hogberg T, Glimelius B, Schmidt M, Wernstedt L, Andersson H, et al. Navoban (tropisetron) alone and in combination with dexamethasone in the prevention of chemotherapy-induced nausea and vomiting: the Nordic experience. The Nordic Antiemetic Trial Group. Anticancer Drugs. 1995;6 Suppl 1:31-6.
311. Soukop M. A comparison of two dose levels of granisetron in patients receiving high-dose cisplatin. The Granisetron Study Group. Eur J Cancer. 1990;26 Suppl 1:S15-9.
312. Soukop M. A dose-finding study of granisetron, a novel antiemetic, in patients receiving high-dose cisplatin. Granisetron Study Group. Support Care Cancer. 1994;2(3):177-83.
313. Soukop M. Management of cyclophosphamide-induced emesis over repeat courses. Oncology. 1996;53 Suppl 1:39-45.

Appendix B. Study Characteristics

Author, year	Country of conduct	Study conduct period	Duration of Study (Days)	Setting	Interventions examined	Outcomes Reported
Randomized clinical trials (n=246)						
Kovacs, 2016	USA, Latin America, Europe, Russia	September 2011 - Oct ober 2012	5	Multi-centre	Palonosetron + Steroid, Ondansetron + Steroid	Nausea, CINV, , Mortality, Use of rescue medication, QT prolongation
Gabrail, 2015(b)	Poland	NR	7	Multi-centre	Granisetron + Steroid	CINV
Karthaus, 2015	USA, Latin America, Europe, Asia, Commonwealth of Independent States	June, 21 2011 - November, 14 2012	5	Multi-centre	Palonosetron + Steroid	Nausea, Vomiting, Mortality, Use of rescue medication, Arrhythmia
Kim K, 2015	South Korea	October 2012 - October 2013	8	Single centre	Ramosetron + Steroid	CINV, Use of rescue medication, QT prolongation
Kim JE, 2015	South Korea	February 2012 - March 2013	5	Multi-centre	Granisetron + Steroid	CINV
Kimura, 2015	Japan	1 April 2011-31 March 2013	10	NR	Palonosetron + Steroid, Granisetron + Steroid	CINV
Ohzawa, 2015	Japan	NR	21	Single centre	Granisetron + Steroid, Palonosetron + Steroid	CINV
Raftopolous, 2015	USA, India, Poland	June 2006 - August 2008	5	Multi-centre	Granisetron + Steroid, Palonosetron + Steroid	Nausea, Vomiting, CINV, Mortality
Sanmukhani, 2014	India	NR	5	Multi-centre	Ramosetron, Ondansetron	CINV, Use of rescue medication
Huang, 2013	China	September 2009 - September 2010	5	Multi-centre	Palonosetron +Granisetron	Nausea, Vomiting
Wenzell, 2013	USA	January 2011-July 2011	5	Single centre	Ondansetron + Steroid ,Palonosetron + Steroid	Nausea, Vomiting, CINV, Use of rescue medication
Boccia, 2013	Europe, USA, Mexico	NR	5	Multi-centre	Palonosetron + Steroid	Nausea, Vomiting, CINV
Endo, 2012	Japan	November 2010 - March 2012,	5	Single centre	Azasetron + Steroid, Granisetron + Steroid	Vomiting, CINV
Ithimakin, 2012	Thailand	March 2009 - March 2010	5	NR	Ondansetron + Steroid, Ondansetron + Steroid + Metoclopramide	Nausea, Vomiting, CINV
Noor, 2012	USA	December 2006 - August 2008	21	Single centre	Palonosetron	Nausea, Vomiting
Roscoe, 2012	USA	May 2007 - September 2010	3	Multi-centre	Granisetron + Steroid + Prochloroperazine, Palonosetron + Steroid + Prochloroperazine	Nausea
Tsuji, 2012	Japan	December 2007 - January 2009	1	Multi-centre	Granisetron + Steroid	Nausea, Vomiting
Boccia, 2011	Europe, Mexico, India and USA	January - October 2006	22	Multi-centre	Granisetron	Vomiting, CINV, Mortality, QT Interval
Dong, 2011	China	May 2009 - March 2010	7	Single centre	Ondansetron, Palonosetron,	Nausea, Vomiting, CINV, Delirium
Giralt, 2011	USA	NR	8	NR	Palonosetron + Steroid	Nausea, Vomiting

Piyush, 2011*****	NR	NR	3	NR	Ondansetron, Granisetron, Palonosetron	CINV
Siddique, 2011	Bangladesh	January 2008 - April 2009	4	Single centre	Granisetron, Ondansetron	Nausea, Vomiting, CINV
Tian, 2011	China	NR	5	Multi-centre	Granisetron, Palonosetron	Vomiting, CINV, Arrhythmia
Ghosh, 2010	India	November 05 2007 - September 30 - 2009	5	Single centre	Granisetron + Steroid, Ondansetron + Steroid, Palonosetron + Steroid	Vomiting, CINV, Mortality, QT Interval
Ho, 2010	Taiwan	January 2006 - December 2007	1	Multi-centre	Granisetron + Steroid, Ramosetron + Steroid	Nausea, Vomiting, CINV
Kaushal, 2010	India	NR	5	Single centre	Ondansetron + Steroid, Palonosetron + Steroid	Nausea, Vomiting
Mattiuzzi, 2010	USA	October 2005 - April 2008	7	NR	Ondansetron + Steroid, Palonosetron + Steroid	Nausea, Vomiting, Arrhythmia
Keyhanian, 2009	Iran	2003-2004	1	NR	Granisetron, Granisetron + Steroid	Nausea, Vomiting
Maemondo, 2009	Japan	April-October 2005	5	Multi-centre	Palonosetron + Steroid	Vomiting, CINV
Saito, 2009	Japan	July 5, 2006 to May 31, 2007	5	Multi-centre	Granisetron + Steroid, Palonosetron + Steroid	Nausea, Vomiting, CINV, Mortality, QT Interval
Segawa, 2009	Japan	April - November 2005	5	Multi-centre	Palonosetron + Steroid	Vomiting, QT Interval
Yonemura, 2009	Japan	January 2006 - October 2006	5	NR	Granisetron	Vomiting, CINV
Yu, 2009	China	May 2006 - December 2006	5	Multi-centre	Granisetron, Palonosetron	Vomiting
Chen, 2008	Taiwan	December 1995 - March 2001	6	Single centre	Ondansetron + Steroid	Nausea, Vomiting, Mortality
Fabi, 2008	Italy	December 2002 - January 2005,	6	NR	Ondansetron + Steroid	Nausea, Vomiting
Nagel, 2008	Canada	April 1999 - February 2007	2	Single centre	Ondansetron, Placebo	Nausea, Vomiting
Sepúlveda-Vildósola, 2008	Mexico	August 2007 - December 2007	7	Single centre	Ondansetron, Palonosetron	Nausea, Vomiting
Berrak, 2007	Turkey	June 2003 - September 2006	5	Single centre	Granisetron	CINV
Herrstedt, 2007	Denmark	NR	9	NR	Tropisetron, Tropisetron + Metopimazine	Nausea, Vomiting
Kadota, 2007*****	USA	NR	1	NR	Palonosetron	Vomiting, Arrhythmia
Pectasides, 2007	Greece	NR	3	Multi-centre	Ondansetron	Nausea, Vomiting
Shi, 2007	China	March 2003 - March 2004	5	NR	Ondansetron, Ramosetron	Nausea, Vomiting
Aapro, 2006	Switzerland, USA, Russia, Mexico, Italy, Germany	July 2000 - December 2001	5	Multi-centre	Ondansetron + Steroid, Palonosetron + Steroid	Nausea, Vomiting, CINV, QT Interval
Pinarli, 2006	Turkey	NR	1	Single centre	Granisetron, Ondansetron	Arrhythmia, QT Interval
Tabei, 2006*****	Japan	NR	5	Multi-centre	Palonosetron	Vomiting
Buyukavci, 2005	Turkey	NR	1	NR	Granisetron, Ondansetron	QT Interval
Cheirsilpa, 2005	Thailand	February 2003 - August 2003	5	Single centre	Granisetron + Steroid, Ramosetron + Steroid	Nausea, Vomiting, CINV
Corapcioglu, 2005	Turkey	October 2003 - March 2004	1	Single centre	Ondansetron + Steroid	CINV
Kurnianda, 2005	Indonesia	June 2003- November 2003	1	Single centre	Granisetron + Steroid	Nausea, Vomiting
Lindley, 2005	USA	NR	5	Multi-centre	Ondansetron + Steroid + Prochlorperazine, Ondansetron + Steroid	Nausea, Vomiting, CINV
Mandanas, 2005	USA	May 1997 - March 2001	6	Multi-centre	Dolasetron + Steroid, Ondansetron + Steroid	Nausea, Vomiting, CINV
Yano, 2005	Japan	NR	2	NR	Granisetron, Ramosetron	Nausea, Vomiting

Eisenberg, 2004	NR	NR	7	Multi-centre	Palonosetron	Vomiting, CINV, QT Interval
Jaing, 2004	Taiwan	July 2000 - June 2002	1	Single centre	Granisetron, Ondansetron	Vomiting, CINV
Kim, 2004	Korea	April 2002 - October 2002	7	NR	Dolasetron, Ondansetron	Nausea, Vomiting, QT Interval
Tantipalakorn, 2004	Thailand	February 1, 2003 - July 31, 2003	1	NR	Ramosetron	Nausea, Vomiting
Villalon, 2004	Philippines	NR	3	Multi-centre	Ramosetron + Dexamethasone, Ramosetron	Nausea, Vomiting, CINV
Aapro, 2003	Switzerland, Italy	May 28, 1996 - April 30, 1999	6	Multi-centre	Granisetron + Steroid, Granisetron + Steroid + Metoclopramide	Vomiting, CINV
Bhatia, 2003	India	April 1998 - February 1999	1	Multi-centre	Ondansetron, Ondansetron + Steroid, Metoclopramide, Metoclopramide + Steroid	Nausea, Vomiting
Eisenberg, 2003	USA, Mexico	May 2000 - December 2001	5	Multi-centre	Dolasetron, Palonosetron	Nausea, Vomiting, CINV, Mortality, QT Interval
Gralla, 2003	Germany, Italy, UK, The Netherlands, Russia.	August 01, 2000 - October 02, 2001	5	Multi-centre	Ondansetron, Palonosetron	Vomiting, CINV, Mortality, QT Interval
Koizumi, 2003	Japan	March 1998 - June 1999	7	Single centre	Granisetron + Steroid, Ramosetron + Steroid	Nausea, Vomiting
Matsuoka, 2003	Japan	May 1997 to June 1999	10	NR	Granisetron, Granisetron + Steroid	Nausea, Vomiting, Arrhythmia
Sagae, 2003	Japan	January 1996 - December 1998	7	Single centre	Granisetron, Granisetron + Steroid	Nausea, Vomiting, CINV
Feng, 2002a	China	NR	1	Multi-centre	Granisetron, Ramosetron	Nausea, Vomiting
Feng, 2002b	China	NR	1	Multi-centre	Granisetron, Ramosetron	Nausea, Vomiting
Kang, 2002	Korea	July 1998 - January 1999	1	Multi-centre	Granisetron, Ramosetron	Nausea, Vomiting
Noda, 2002	Japan	NR	1	Multi-centre	Ondansetron, Ramosetron	Nausea, Vomiting
Noda, 2002	Japan	NR	1	Multi-centre	Ramosetron, Placebo	Nausea, Vomiting, CINV
Aksoylar, 2001	Turkey	NR	8	Single centre	Granisetron, Tropisetron	Nausea, Vomiting, CINV
de Wit, 2001	The Netherlands	NR	NR	NR	Granisetron + Steroid, Ondansetron + Steroid	Vomiting, CINV
Fox-Geiman, 2001	USA	September 1997 - September 1998	8	NR	Granisetron + Steroid, Ondansetron + Steroid	Nausea, Vomiting, CINV
Koralewski, 2001	Poland	NR	7	Multi-centre	Ondansetron + Steroid, Metoclopramide + Steroid	Nausea, Vomiting, Mortality
Ozgur Tan, 2001	Turkey	NR	less than one day	Single centre	Ondansetron, Placebo	Nausea, Vomiting
Parker, 2001	USA	NR	2	Single centre	Ondansetron, Placebo	Vomiting
Tsavaris, 2001a	Greece	November 1995 - May 1997	4	NR	Ondansetron + Steroid	Nausea, Vomiting
Tsavaris, 2001b	Greece	NR	4	NR	Ondansetron + Steroid	Nausea, Vomiting
Abang, 2000	USA	January 1996 - October 1997	10	NR	Granisetron	Vomiting, Delirium, Mortality
Barrajon, 2000	Spain	January 16, 1995 - August 19, 1996	7	Single centre	Granisetron + Steroid, Ondansetron + Steroid, Tropisetron + Steroid	Nausea, Vomiting
Chiou, 2000	Taiwan	NR	7	NR	Granisetron + Steroid, Ondansetron + Steroid	Nausea, Vomiting
Chua, 2000	China	March 1996 - May 1998	3	NR	Granisetron + Steroid, Ondansetron + Steroid, Tropisetron + Steroid	CINV
Fauser , 2000	Germany, Italy, Austria, France, UK	December 1995 - November 1997	5	Multi-centre	Dolasetron, Dolasetron + Steroid	Nausea, Vomiting

Forni, 2000	Italy	November 1995 - December 1997	717	NR	Granisetron + Steroid, Ondansetron + Steroid, Tropisetron + Steroid	Vomiting
Friedman, 2000	USA	from 1992 - 1993	10	Multi-centre	Granisetron, Prochlorperazine	Nausea, Vomiting, Mortality, Sudden Caridac Death
Herrington, 2000	USA	July 1997 - February1999	1	Single centre	Granisetron + Steroid, Ondansetron + Steroid	Nausea, Vomiting, CINV
Hesketh, 2000	USA	NR	1	Multi-centre	Granisetron + Steroid	Nausea, Vomiting, CINV
Koenig, 2000	Germany	NR	10	NR	Metoclopramide, Tropisetron	CINV
Oge, 2000	Turkey	October 1997 - March 1998	3	Single centre	Granisetron, Ondansetron, Tropisetron	Vomiting, CINV
Raynov, 2000	Bulgaria	1992 - 1997	6	Single centre	Granisetron, Granisetron + Steroid, Metoclopramide, Metoclopramide + Steroid, Ondasetron, Ondansetron + Steroid	Nausea, Vomiting
Roila [The Italian Group for Antiemetic Research], 2000	Italy, Yugoslavia	September 1997 - June 1999	5	Multi-centre	Ondansetron, Ondansetron + Steroid, Dexamethasone, Placebo	Nausea, Vomiting, CINV, Mortality
Slaby, 2000	Czech Republic	December 1996 - March 1998	10	NR	Granisetron, Ondansetron, Tropisetron	Vomiting, CINV
Stewart, 2000	UK	NR	NR	Single centre	Ondansetron + Steroid and/or Metoclopramide, Granisetron + Steroid and/or Metoclopramide	Nausea, Vomiting
White, 2000	Austrailia, UK, Russia, Mexico, Ireland, Czech Republic, Taiwan, Poland, Belgium,…13 countries	NR	8	Multi-centre	Ondansetron + Steroid	Nausea, Vomiting
Davidson, 1999	Australia, Austria, Belgium, Canada, France, Germany, Holland, New Zealand, Norway, South Africa, Spain, Switzerland, UK	NR	3	Multi-centre	Ondansetron	Nausea, Vomiting
Kandemir, 1999	Turkey	NR	5	NR	Ondansetron + Steroid, Ondansetron + Steroid + Metoclopramide	CINV
Komada, 1999	Japan	March 1994 - June 1996	3	NR	Granisetron	Vomiting
Lazarus, 1999	USA	NR	2	Single centre	Granisetron	Vomiting
Needles, 1999	USA, Puerto Rico, Mexico	NR	8	Multi-centre	Ondansetron	Nausea, Vomiting
Orchard, 1999	USA	NR	10	Single centre	Granisetron + Steroid, Ondansetron + Steroid	Nausea, Vomiting, Delirium
Raynov, 1999	Bulgaria	NR	NR	Single centre	Ondansetron	Nausea, Vomiting
Sandoval, 1999	USA	NR	2	NR	Ondansetron	CINV
Sigsgaard, 1999	Denmark	NR	5	NR	Granisetron, Metopimazine + Steroid	CINV
Stiakaki, 1999	Greece	July 1995 - June 1996	1	NR	Ondansetron, Tropisetron	CINV
Tatsumi, 1999	Japan	July 1995 - March 1997	1	Multi-centre	Granisetron	Nausea, Vomiting
The Italian Multicenter Study Group, 1999	Italy	NR	3	Multi-centre	Granisetron, Granisetron + Steroid	Nausea, Vomiting, CINV

Tsuchida, 1999	Japan	February 1994 - July 1996	1	NR	Granisetron	Vomiting
Uchida, 1999	Japan	January 1994 - April 1995	5	NR	Granisetron, Placebo	Nausea, Vomiting, CINV
Yalcn, 1999	Turkey	NR	5	NR	Granisetron, Ondansetron, Tropisetron	Vomiting
Birch, 1998	USA	April 1995 - May 1997	5	Multi-centre	Granisetron + Steroid	CINV
Gralla, 1998	USA	NR	1	Multi-centre	Granisetron + Steroid, Ondansetron + Steroid	Nausea, Vomiting, CINV
Handberg, 1998	Denmark	NR	3	NR	Granisetron, Granisetron + Steroid	Nausea, Vomiting
Kalaycio, 1998	USA	September 1994 - April 1996	7	NR	Granisetron + Steroid, Ondansetron + Steroid	Vomiting
Kleisbauer, 1998	NR	NR	11	NR	Granisetron, Granisetron + Steroid	Nausea, Vomiting, CINV, Mortality
Krzakowski, 1998	Canada, France,Germany, Iceland, Italy, Poland, South Africa, UK	NR	1	Multi-centre	Ondansetron + Steroid	Nausea, Vomiting
Latreille, 1998	Canada	April 1991- June 1994	7	Multi-centre	Granisetron + Steroid	Nausea, Vomiting, CINV
Martoni, 1998	Italy	July 1994 - February 1997	56	Single centre	Granisetron + Steroid	Nausea, Vomiting, CINV
Perez, 1998	USA	NR	30	Multi-centre	Granisetron + Steroid, Ondansetron + Steroid	Nausea, Vomiting, CINV
Perez, 1998	USA	May 1994 - August 1995	2	NR	Granisetron + Steroid, Ondansetron + Steroid	Nausea, Vomiting, CINV, Arrhythmia
Poon, 1998	Hong Kong	NR	7	NR	Granisetron, Ondansetron	Nausea, Vomiting
Ritter, 1998	USA	August 1992 - February 1994	7	Multi-centre	Granisetron	CINV
Scoponi, 1998	Italy	Jan 4, 1994 - Jan 10, 1996	NR	NR	Granisetron + Steroid, Ondansetron + Steroid	Nausea, CINV
Sorbe, 1998	Sweden	January 01, 1995 - June 30, 1996	6	Multi-centre	Tropisetron + Steroid, Dexamethasone	Nausea, Vomiting, CINV
Spector, 1998	USA	NR	1	Multi-centre	Granisetron, Ondansetron	Nausea, Vomiting
Tsavaris, 1998	Greece	NR	4	NR	Ondansetron	Vomiting
Zeidman, 1998	Israel	April 1994 - October 1994	1	Single centre	Granisetron, Ondansetron	Nausea, Vomiting
Beck, 1997	USA	NR	3	Multi-centre	Ondansetron	Nausea, Vomiting, Mortality
Chevallier, 1997	France	January 1992 - February 1994	1	Multi-centre	Dolasetron, Metoclopramide	Nausea, Vomiting, Mortality
Davidson, 1997	Australia, Austria, Belgium, Denmark, France, Germany, Holland, Israel, Norway, South Africa, Sweden, Switzerland, UK	NR	3	Multi-centre	Ondansetron	Nausea, Vomiting
du Bois, 1997	Austria, Belgium, Canada, Germany, Hungary, Ireland, Italy, Spain, Sweden, United Arab Emirates/Bahrain, United Kingdom	NR	3	NR	Ondansetron + Steroid, Metoclopramide + Steroid	Nausea, Vomiting, Delirium, Mortality, Sudden Caridac Death
Fumoleau, 1997	France	NR	3	Multi-centre	Ondansetron	Nausea, Vomiting
Gridelli, 1997	Italy	NR	5	NR	Ondansetron, Ondansetron + Steroid	Nausea, Vomiting
Grote, 1997	USA	NR	2	Multi-centre	Dolasetron	Nausea, Vomiting, Arrhythmia, QT

						Interval
Kasimis, 1997	USA	NR	1	Multi-centre	Dolasetron	Nausea, Vomiting, QT Interval
Kigawa, 1997	Japan	January 1993 - December 1995	1	Single centre	Granisetron, Granisetron + Steroid, Metoclopramide	Vomiting, CINV
Kris, 1997	USA	March 1995 - November 1995	1	Multi-centre	Dolasetron + Steroid	Nausea, Vomiting, Mortality
Lofters, 1997	Canada	May 1993 - January 1995	7	Multi-centre	Dolasetron, Dolasetron + Steroid, Ondansetron, Ondansetron + Steroid	Nausea, Vomiting, Mortality, QT Interval, QRS interval
Mustacchi, 1997	Italy	NR	6	Single centre	Ondansetron + Steroid + Metoclopramide	CINV
Park, 1997	Korea	December 1994 - May 1995	7	Single centre	Granisetron, Ondansetron	Vomiting, CINV, Delirium
Pectasides, 1997	Greece	October 1992 - May 1994	5	Single centre	Ondansetron + Steroid	Nausea, Vomiting
Perez, 1997	USA	NR	7	Multi-centre	Granisetron	Nausea, Vomiting, Arrhythmia
Roila [The Italian Group for Antiemetic Research], 1997	Italy	December 1994 - September 1995	6	Multi-centre	Ondansetron + Steroid, Ondansetron + Steroid + Metoclopramide	Nausea, Vomiting, CINV
Rubenstein, 1997	USA	NR	1	Multi-centre	Dolasetron	Nausea, Vomiting, Arrhythmia, QT Interval
Advani, 1996	India	NR	5	Single centre	Ondansetron, Metoclopramide	Nausea, Vomiting
Audhuy, 1996	France, Spain, Belgium	NR	1	Multi-centre	Dolasetron, Granisetron	Nausea, Vomiting, Arrhythmia, QT Interval
Barrenetxea, 1996	Spain	NR	5	Single centre	Ondansetron, Ondansetron + Metoclopramide	Vomiting
Brock, 1996	Belgium, Netherlands, Denmark, Sweden and Finland	November 1992 - June 1994	9	NR	Ondansetron	Nausea, Vomiting
Crucitt, 1996	USA	NR	3	Multi-centre	Ondansetron, Prochlorperazine	Nausea, Vomiting
de Wit, 1996	The Netherlands	NR	1	NR	Ondansetron	Nausea, Vomiting
Fauser, 1996a	NR	NR	1	Multi-centre	Dolasetron, Metoclopramide	Nausea, Vomiting, CINV, Arrhythmia, Mortality, QT Interval
Fauser, 1996b	Italy, France, Belgium, Germany, Spain,	NR	1	Multi-centre	Dolasetron, Ondansetron	Nausea, Vomiting, Mortality
Fujimoto, 1996	Japan	NR	1	Single centre	Granisetron, Placebo	Vomiting, CINV
Hesketh, 1996b	USA	NR	1	Multi-centre	Dolasetron, Ondansetron	Nausea, Vomiting, Arrhythmia, Mortality, QT Interval
Jorgensen, 1996	Denmark	NR	5	Single centre	Ondansetron + Steroid, Metoclopramide + Steroid	Nausea, Vomiting, CINV
Leonardi, 1996	Italy	NR	NR	NR	Granisetron, Ondansetron	CINV
Martoni, 1996	Italy	March 1993 - June 1994	1	Single centre	Granisetron, Ondansetron	Nausea, Vomiting, CINV
Massida & Ionta, 1996	Italy	NR	5	NR	Granisetron, Ondansetron, Tropisetron	Nausea, Vomiting, CINV
Matsui, 1996	Japan	April 1993 - January 1995	5	NR	Granisetron, Granisetron + Steroid + Prochlorperazine	Nausea, Vomiting
Olver, 1996	Austria, Australia, Belgium, Canada, France, Germany, Ireland, Israel, Netherlands,	NR	6	Multi-centre	Ondansetron, Placebo	Nausea, Vomiting, CINV

	Protugal, South Africa, UK					
Peterson, 1996	Sweden	NR	14	Single centre	Ondansetron, Ondansetron + Steroid	Nausea, Vomiting
Yeilding, 1996	USA	NR	1	Multi-centre	Dolasetron	Nausea, Vomiting
Adams, 1995	UK	NR	5	Multi-centre	Tropisetron, Tropisetron + Steroid	Nausea, Vomiting, CINV
Agura, 1995a	USA	July 1991 - September 1992	35	Single centre	Ondansetron	Nausea, Vomiting, QT Interval
Agura, 1995b	USA	July 1991 - September 1992	35	Single centre	Ondansetron	Nausea, Vomiting
Alfieri, 1995	Venezuela	NR	1	NR	Ondansetron	Vomiting
Alvarez, 1995	USA	October 1991 - December 1993	1	Multi-centre	Ondansetron, Ondansetron + Steroid	Vomiting
Bleiberg, 1995	France, Belgium, Italy, Switzerland, Netherlands, Germany, Norway, South Africa, United Kingdom	NR	14	Multi-centre	Granisetron	CINV
Bonneterre, 1995	France	June 1992 - March 1993	5	NR	Granisetron, Ondansetron	Nausea, Vomiting, CINV, Mortality
Chiou, 1995	Taiwan	NR	6	NR	Granisetron, Metoclopramide	CINV
Contu, 1995	Italy	NR	1	Multi-centre	Granisetron	Nausea, Vomiting, CINV
DiBenedetto, 1995	USA	NR	3	NR	Ondansetron, Placebo	Vomiting
Dick, 1995	UK	NR	4	NR	Ondansetron, Metoclopramide + Steroid	Vomiting
Fedele, 1995	Italy	June 1993 - August 1994	NR	Single centre	Granisetron, Ondansetron	Nausea, Vomiting, CINV
Gebbia, 1995	Italy	NR	5	NR	Granisetron, Granisetron + Steroid, Granisetron + Steroid + Metoclopramide	Nausea, Vomiting
Hahlen, 1995	France, The Netherlands, UK	NR	1	Multi-centre	Granisetron, Chlorpromazine + Steroid	Nausea, Vomiting, CINV
Italian Group for Antiemetic Research, 1995	Italy	December 1992 - July 1994	6	NR	Granisetron + Steroid + Metoclopramide, Ondansetron + Steroid + Metoclopramide	Nausea, Vomiting, CINV, Mortality
Labar, 1995	Croatia	January 1992 - December 1993	15	Single centre	Ondansetron, Metoclopramide	Nausea, Vomiting
Latreille, 1995	Canada	NR	3	Multi-centre	Granisetron, Granisetron + Steroid	Nausea, Vomiting
Mantovani, 1995	Italy	NR	1	NR	Granisetron, Ondansetron, Tropisetron	CINV
Marty, 1995	France, Switzerland	NR	6	Multi-centre	Ondansetron, Tropisetron	Nausea, Vomiting, CINV, Mortality
Navari, 1995a	USA	NR	6	Multi-centre	Ondansetron	Nausea, Vomiting
Navari, 1995b	USA	NR	11	Multi-centre	Granisetron, Ondansetron	Nausea, Vomiting, CINV
Stewart, 1995	Denmark, France, Germany, Holland, UK, South Africa	NR	5	Multi-centre	Granisetron, Ondansetron	Nausea, Vomiting
The Italian Group for Antiemetic Research, 1995	Italy	December 1992 - January 1994	5	Multi-centre	Granisetron, Dexamethasone, Granisetron + Steroid	Nausea, Vomiting, CINV
Tsavaris, 1995a	Greece	NR	1	NR	Ondansetron, Metoclopramide	Nausea, Vomiting, Delirium
Tsavaris, 1995b	Greece	November 1990 - May 1992	4	NR	Ondansetron	Nausea, Vomiting
Ahn, 1994	South Korea	October 1991- April 1992	5	NR	Ondansetron, Ondansetron + Steroid	Nausea, Vomiting
Carmichael, 1994	UK	NR	2	Multi-centre	Granisetron, Granisetron + Steroid	Nausea, Vomiting, CINV

Chevallier, 1994	France	NR	7	Multi-centre	Ondansetron, Ondansetron + Metoclopramide	Nausea, Vomiting
Chiu, 1994	Hong Kong	February 1990 - September 1991	5	NR	Ondansetron, Metoclopramide	Nausea, Vomiting
Cubeddu, 1994	Venezuela	NR	3	Multi-centre	Ondansetron, Placebo	Nausea, Vomiting
Gebbia, 1994a	Italy	NR	5	NR	Granisetron, Ondansetron	Nausea, Vomiting
Gebbia, 1994b	Italy	NR	5	NR	Granisetron, Ondansetron	Nausea, Vomiting
Heron, 1994	Belgium, France, Germany, Iceland, Netherlands, Norway, South Aftica, Spain, Switzerland, UK	NR	7	Multi-centre	Granisetron, Granisetron + Steroid, Metoclopramide + Steroid	Nausea, Vomiting, CINV, Delirium, Mortality
Hesketh, 1994	USA	NR	6	Multi-centre	Ondansetron, Ondansetron + Steroid	Nausea, Vomiting
Hulstaert, 1994	Belgium	March 1992 - June 1993	6	Multi-centre	Tropisetron, Tropisetron + Steroid	CINV
Joss, 1994	Switzerland	July 1990-November 1991	4	Multi-centre	Ondansetron, Ondansetron + Steroid	Nausea, Vomiting, CINV, Mortality, Sudden Caridac Death
Kaizer, 1994	Canada	March 1, 1991 - August 2, 1991	5	Multi-centre	Ondansetron + Steroid	Nausea, Vomiting
Lim, 1994	Malaysia	January 1991-June 1991	6	Single centre	Ondansetron, Metoclopramide + Steroid	Nausea, Vomiting
Navari, 1994	USA	November 1989-February 1991	1	NR	Granisetron	Nausea, Vomiting
Noble, 1994	Austria, France, Germany, The Netherlands, South Africa, UK	July 1991 - November 1992	5	Multi-centre	Granisetron, Ondansetron	Vomiting, CINV, Mortality
Riviere, 1994	France, Germany, South Africa	NR	7	Multi-centre	Granisetron	Nausea, Vomiting, CINV, Mortality
Ruff, 1994	Denmark, France, Germany, Netherlands, South Africa, Switzerland, UK	December 1991 - November 1992	1	Multi-centre	Granisetron, Ondansetron	Nausea, Vomiting
Sorbe, 1994	Sweden and Finland	February 1, 1991- February 29, 1992	6	Multi-centre	Tropisetron, Tropisetron + Steroid	Nausea, Vomiting
Suarez, 1994	France	NR	5	NR	Tropisetron, Placebo	Nausea, Vomiting
Tsavaris, 1994	Greece	October 1990 - May 1992	4	NR	Ondansetron	Nausea, Vomiting
Tyson, 1994	USA	December 1989 - December 1990	1	NR	Ondansetron + Steroid	Nausea, Vomiting
Van Belle, 1994a	Switzerland, Belgium	August 1987 - November 1989	7	NR	Tropisetron	Nausea, Vomiting, CINV, Mortality
Van Belle, 1994b	Switzerland, Belgium	August 1987 - November 1989	7	Multi-centre	Tropisetron	Nausea, Vomiting, CINV, Mortality
Beck, 1993	USA	March 1989 and January 1990	3	Multi-centre	Ondansetron, Placebo	Nausea, Vomiting
Bosi, 1993	Italy	NR	8	NR	Ondansetron, Chlorpromazine	Nausea, Vomiting
Buser, 1993	Switzerland	May 1988 - September 1989	15	NR	Ondansetron, Placebo	Nausea, Vomiting, CINV
Chevallier, 1993	France, Switzerland, UK and West Germany	NR	7	NR	Granisetron, Metoclopramide + Steroid	CINV, Arrhythmia, Delirium
Cunningham, 1993	UK	NR	1	Multi-centre	Ondansetron, Metoclopramide	Vomiting
Grunberg, 1993	USA	NR	1	Multi-centre	Ondansetron	Nausea, Vomiting
Herrstedt, 1993	Denmark	NR	5	NR	Ondansetron, Ondansetron + Metopimazine	Nausea, Vomiting

Hunter, 1993	France, Germany, Holland, Switzerland, UK	NR	5	Multi-centre	Granisetron, Metoclopramide + Steroid	CINV, Delirium
Jantunen, 1993	Finland	NR	1	Multi-centre	Ondansetron, Tropisetron, Granisetron	Vomiting
Levitt, 1993	Canada	July 1989 - November 1990	8	Multi-centre	Ondansetron, Metoclopramide + Steroid	Nausea, Vomiting
Madej, 1993	Poland	NR	5	NR	Metoclopramide, Tropisetron + Steroid	Nausea, Vomiting
Rath, 1993	France, Germany, Mexico, Norway, Sweden, UK	NR	5	Multi-centre	Ondansetron, Metoclopramide + Steroid	Nausea, Vomiting
Smith (Granisetron Study Group), 1993	Belgium, Switzerland, The Netherlands, France, UK, South Africa.	NR	7	Multi-centre	Granisetron	Nausea, CINV, Arrhythmia, Mortality
Beck, 1992	USA	NR	1	Multi-centre	Ondansetron	Vomiting
Bots, 1992	Netherlands	September 1988 - July 1989	6	NR	Granisetron	CINV
Dicato, 1992	Norway, Italy, Luxembourg, Austria, Belgium, Portugal, Spain, United Kingdom	NR	5	NR	Ondansetron	Nausea, Vomiting
Jantunen, 1992	Finland	NR	1	NR	Ondansetron + Steroid, Tropisetron + Steroid	Vomiting
Seynaeve, 1992	Austria, Belgium, Finland, Denmark, Holland, Iceland, Israel, Luxembourg, Spain, UK, Germany.	September 1989 - June 1990	1	Multi-centre	Ondansetron	Nausea, Vomiting, QT Interval
Sledge, 1992	USA	October 1988 - September 1989	5	Single centre	Ondansetron, Metoclopramide	Nausea, Vomiting
Soukop, 1992	UK, Ireland	NR	5	Multi-centre	Ondansetron + Steroid, Metoclopramide + Steroid	Nausea, Vomiting
Gandara, 1991	USA	NR	1	Multi-centre	Ondansetron, Metoclopramide	Vomiting
Hainsworth, 1991	USA	June 1988 - June 1989	1	Multi-centre	Ondansetron, Metoclopramide	Nausea, Vomiting, CINV
Jones, 1991	UK, Ireland	NR	5	NR	Ondansetron, Dexamethasone	Nausea, Vomiting, Delirium
Marschner, 1991	Germany	NR	3	Multi-centre	Ondansetron, Metoclopramide	Nausea, Vomiting
Roila, 1991	Italy	June 1989- November 1989	1	Multi-centre	Ondansetron, Ondansetron + Steroid	Nausea, Vomiting, CINV
Smith, 1991	UK	NR	8	NR	Ondansetron, Ondansetron + Steroid	Nausea, Vomiting, Mortality
Smyth, 1991	UK, Germany	NR	1	Multi-centre	Ondansetron, Ondansetron + Steroid	Nausea, Vomiting, Mortality
Warr, 1991	Canada	April 1988 - March 1989	1	Multi-centre	Granisetron, Prochlorperazine + Steroid	Nausea, Vomiting, Delirium
Bonneterre, 1990	France	NR	3	NR	Ondansetron, Metoclopramide	Nausea, Vomiting
Cubeddu, 1990a	Venezuela	NR	4	Single centre	Ondansetron, Placebo	Nausea, Vomiting
Cubeddu, 1990b	Venezuela	NR	4	Single centre	Ondansetron, Placebo	Nausea, Vomiting
Cupissol, 1990	France	NR	1	Single centre	Granisetron, Placebo	Nausea, Vomiting, CINV, Mortality
De Mulder, 1990	The Netherlands	May 1988 -February 1989	6	Multi-centre	Ondansetron, Metoclopramide	Nausea, Vomiting, Delirium, Mortality
Falkson, 1990	South Africa	NR	1	NR	Granisetron	Vomiting
Kaasa, 1990	Norway, Luxembourg,	NR	3	NR	Ondansetron, Metoclopramide	Nausea, Vomiting, Delirium

	Belgium, UK					
Khojasteh, 1990	USA	NR	1	Multi-centre	Ondansetron	Nausea, Vomiting
Marty, 1990a	France, Germany, UK, Switzerland, Belgium	NR	7	Single centre	Granisetron, Chlorpromazine + Steroid	Nausea, Vomiting, CINV
Marty, 1990b	France, UK	NR	7	Multi-centre	Ondansetron, Metoclopramide	Nausea, Vomiting

Non-Randomized Clinical Trials (n=33)

Gabrail, 2015(a)	USA	NR	7	NR	Granisetron	
Kim, 2014	Korea	September 2010 - October 2011	7	Multi-centre	Ramosetron + Steroid, Tropisetron	
Abali, 2007	Turkey	NR	5	Single centre	Granisetron + Steroid, Ondansetron + Steroid, Tropisetron + Steroid	Nausea, Vomiting
Tan, 2004	USA	February 1, 2001 - March 31, 2001	1	Single centre	Dolasetron + Steroid, Granisetron + Steroid	Nausea, Vomiting, CINV
Carmichael, 2003	UK	NR	2	NR	Granisetron	Arrhythmia, Delirium
Raynov, 2002	Bulgaria	NR	NR [3 cycles]	Single centre	Ondasetron, Ondansetron + Steroid, Metoclopramide, Metoclopramide + Steroid	CINV
Yamac, 2002	Turkey	NR	NR	NR	Granisetron, Ondansetron, Tropisetron	CINV
Liaw, 2001	Taiwan	December 1995 to July 2000	6	Single centre	Ondansetron + Steroid	Nausea, Vomiting
Watters, 2001	UK	NR	7	Single centre	Granisetron + Steroid + Metoclopramide, Ondansetron + Steroid + Metoclopramide, Tropisetron + Steroid + Metoclopramide	Nausea, Vomiting
Zeng, 2001	China	August 2000 - October 2000	1	NR	Ramosetron, Tropisetron	Nausea, Vomiting
Coppes, 1999a	Canada	NR	1	Multi-centre	Dolasetron	Vomiting, Mortality, Arrhythmia, QT Interval
Coppes, 1999b	Canada, USA	NR	1	Multi-centre	Dolasetron	Vomiting, Mortality, Arrhythmia, QT Interval
Tsavaris, 1999	Greece	NR	3	NR	Ondansetron + Steroid, Metoclopramide + Steroid	Vomiting
Kris, 1998	USA	December 1986-October 1987	1	NR	Ondansetron	Nausea, Mortality
Chang, 1997	China	NR	7	Single centre	Granisetron, Metoclopramide + Steroid	CINV
Belkacemi, 1996	France	March 1992 - February 1994	less than one day	Multi-centre	Granisetron	CINV
Gaedicke, 1996	Germany, Switzerland	NR	7	Multi-centre	Tropisetron	Nausea, Vomiting, CINV
Hesketh, 1996a	USA	NR	2	Multi-centre	Dolasetron	Nausea, Vomiting, QT Interval
Hesketh, 1995	USA	NR	1	Multi-centre	Ondansetron + Steroid	Nausea, Vomiting
Conroy, 1994	France, Germany, The Netherlands	August 1989 - August 1991	1	Multi-centre	Dolasetron	Nausea, Vomiting
Kris, 1994	USA	February 1991 - March 1992	1	Multi-centre	Dolasetron	Nausea, Vomiting, Arrhythmia, Mortality, QT interval

Merrouche, 1994	France	NR	5	NR	Dolasetron	Nausea, Vomiting
de Wet, 1993	South Africa	NR	1	NR	Granisetron	CINV
Pisters, 1993	USA	March 1988 - August 1988	1	NR	Tropisetron	Nausea, Vomiting, Mortality
Ribiero, 1993	Portugal	NR	5	Multi-centre	Ondansetron	Nausea, Vomiting
Dundee, 1992	Northern Ireland	NR	5	Single centre	Ondansetron, Placebo	CINV
Fraschini, 1991	USA	October 1988 - December 1989	2	NR	Ondansetron	Nausea, Vomiting
Lemerle, 1991	France	NR	1	Single centre	Granisetron	CINV, Arrhythmia
Seynaeve, 1991	The Netherlands, UK.	NR	5	NR	Ondansetron	Nausea, Vomiting, QT Interval
Addelman, 1990	Canada	NR	NR	NR	Granisetron	Nausea, Vomiting, Arrhythmia, Mortality
Cunningham, 1989	UK	NR	7	NR	Ondansetron, Ondansetron + Steroid	Nausea, Vomiting, CINV
Grunberg, 1989	USA	NR	1	NR	Ondansetron	Nausea, Vomiting
De Hann, 1988	The Netherlands	NR	1	NR	Ondansetron	Nausea, Vomiting

Cohort Studies (n=19)

Anvari, 2015	Iran	June 2009 - July 2010	7	Multi-centre	Metoclopramide + Steroid, Granisetron + Steroid	
Zoto, 2015	Turkey	NR	5	Single centre	Granisetron + Steroid, Ondansetron + Steroid	
Murakami, 2014	Japan	July 2010 - November 2012	5	Single centre	Granisetron + Steroid, Palonosetron + Steroid	
Keat, 2013	Malaysia	September 2011 - December 2011	5	Single centre	Granisetron + Metoclopramide + Steroid, Metoclopramide + Steroid	
Blazer, 2012	USA	January 2009 – June 2009 for ondansetron group; April 2010 – November 2010 for palonestron group.	5	Single centre	Ondansetron + Steroid, Palonosetron + Steroid	CINV
Pielichowski, 2011	Poland	NR	5	Single centre	Ondansetron + Steroid, Palonosetron + Steroid	CINV
Taguchi, 2009	Japan	January 2007 - December 2007	4	Multi-centre	Granisetron + Steroid	Vomiting, Nausea
Carreca, 2007*****	NR	NR	NR	NR	Palonosetron + Steroid, Ondansetron + Steroid	CINV
Hamadani, 2007	USA	NR	1	Single centre	Dolasetron + Steroid, Granisetron + Steroid, Ondansetron + Steroid	Nausea, Vomiting
Adel, 2006 *****	USA	January 2005 - December 2005	12	Single centre	Palonosetron, Palonosetron + Steroid	Nausea, Vomiting
Shikimori, 2006	Japan	NR	NR	Single centre	Granisetron, Metoclopramide	CINV
Trifilio, 2006*****	USA	NR	7	NR	Palonosetron, Ondansetron	Nausea, Vomiting
Babaoglu, 2005	Turkey	April 2002 - October 2004	5	Single centre	Granisetron, Ondansetron, Tropisetron	CINV
Dempsey, 2004	USA	January 1998 - June 2002	3	Multi-centre	Granisetron + Steroid,	Nausea, Vomiting, CINV

					Ondansetron + Steroid	
Steiner, 2003	USA	pre and post November 2000	3	NR	Granisetron + Steroid, Dolasetron + Steroid	CINV
Brown, 1998	UK	December 1993 - June 1996	392 to 469	NR	Granisetron + Steroid + Metoclopramide, Metoclopramide + Steroid	Vomiting
Farley, 1997	USA	June 1995 - November 1995	3	Multi-centre	Granisetron, Ondansetron	Nausea, Vomiting, CINV
Harris, 1997	USA	NR	3	Multi-centre	Granisetron, Granisetron + Steroid, Ondansetron, Ondansetron + Steroid	Vomiting, CINV
Blijham, 1992	NR	Study 1: April 1988 and June 1989; Study 2: from July 1989-May 1991	1	NR	Granisetron	CINV
Controlled Before and After Study (n=1)						
Rzepecki, 2009	Poland	NR	5	NR	Ondansetron + Steroid, Palonosetron + Steroid	CINV

Abbreviations: CINV – chemotherapy-induced nausea and vomiting; NR - not reported; UK - United Kingdom; USA - United States of America
* Includes unpublished data (Adel 2006, Tabei 2006, Trifilio 2006, Carreca 2007, Kadota 2007, Piyush 2011); full citations are provided in Appendix A.

Appendix C. Patient Characteristics

Author, year	Age Category	Sample Size	% female	Cancer Diagnosis	Use of CDDP	Treatment Details	H/o motion sickness	H/o CINV	Co-morbidities
Randomized Controlled Trials (n=246)									
Kovacs, 2016	Children (≤18)	331	46.86	Acute lymphocytic Leukaemia, Nephroblastoma, Rhabdomyosarcoma, Medulloblastoma, B precursor type acute leukaemia, Ewings sarcoma, Hodgkin's disease	NO	NR	NR	NR	NR
Gabrail, 2015(b)	Aduts (18-65)	35	100	Ovarian, breast, lung, lymphoma/leukemia, endometrial/cervical/vulvar, colorectal, bladder	Partial	NR	NR	NR	NR
Karthaus, 2015	Adults & Elderly (18-99)	739	41	Gastric, Head and neck, Lung/respiratory tract, Ovarian, Bladder, Others	NO	NR	NR	NR	NR
Kim K, 2015	Adults & Elderly (18-99)	32	34	Colon cancer stage III and IV	NO	NR	NR	NR	NR
Kim JE, 2015	Adults & Elderly (18-99)	261	37.5	Gasterointestinal, Lung, Others	NO	NR	NR	NR	NR
Kimura, 2015	Adults & Elderly (18-99)	24	50	Osteosarcoma, Malignant fibrous histiosarcoma, Synovial sarcoma, Leiomyosarcoma, Rhabdomyosarcoma, De-differenciated liposarcoma and pleomorphic sarcoma, MYLPS	NO	NR	NR	NR	NR
Ohzawa, 2015	Adults & Elderly (18-99)	40	100	Breast	NO	Adjuvant/Neoadjuvant	NR	NR	NR
Raftopolous, 2015	Adults & Elderly (18-99)	1395	74.6	Lung, Breast, Ovarian, Lymphoma	NO	NR	NR	NR	NR
Sanmukhani, 2014	Adults & Elderly (18-99)	214	65.9	Breast, Head and Neck, Ovary, Lung and bronchus, Gastrointestinal tract, Hematopoietic system, Musculoskeletal system	NO	NR	NR	NR	NR
Huang, 2013	Adults & Elderly (18-99)	236	73.3	Non-small cell lung, Colorectal, Gastric, Breast, Esophageal, Head and Neck, Ovarian	NO	NR	NR	NR	NR
Wenzell, 2013	Adults & Elderly (18-99)	40	100	Breast, Lymphoma	NO	NR	YES	NR	NR
Boccia, 2013	Adults & Elderly (18-99)	639	73.1	Breast, Colon, Lung	NO	NR	NR	YES	NR
Endo, 2012	Adults & Elderly (18-99)	105	14.1	Lung, Respiratory	NO	NR	NR	NR	NR
Ithimakin, 2012	Adults & Elderly (18-99)	162	27.0	Head and Neck, Lung, Sarcoma, Gastrointestinal, Other	YES	Radiotherapy	YES	NR	NR
Noor, 2012	Adults (18-65)	30	36.4	Melanoma	YES	NR	NR	NR	NR

Roscoe, 2012	Adults & Elderly (18-99)	944	80.0	Gastrointestinal, Breast, Gastrointestinal, Genitourinary, Gynecologic, Head And Neck, Hematologic	Partial	NR	YES	NR	NR
Tsuji, 2012	Adults & Elderly (18-99)	359	54.9	Gastrointestinal, Breast, Lung, Other	YES	NR	NR	NR	NR
Boccia, 2011	Adults & Elderly (18-99)	621	51.5	NR	YES	NR	NR	NR	NR
Dong, 2011	Adults & Elderly (18-99)	89	36.0	NR	YES	NR	NR	YES	NR
Giralt, 2011	Adults & Elderly (18-99)	73	35.6	Hematological	NO	NR	NR	YES	NR
Piyush, 2011*	NR	90	NR	Head and Neck	YES	NR	NR	NR	NR
Siddique, 2011	Children (≤18)	60	NR	Hematological	NO	NR	NR	NR	NR
Tian, 2011	Adults & Elderly (18-99)	144	39.6	Lung, Breast, Gastrointestinal	YES	NR	NR	YES	NR
Ghosh, 2010	Adults (18-65)	1213	54.2	NR	NR	NR	NR	NR	NR
Ho, 2010	Adults & Elderly (18-99)	285	61.4	Breast, Lung, Head and Neck, Gastrointestinal, Genitourinary	YES	NR	NR	NR	NR
Kaushal, 2010	Adults (18-65)	30	NR	Head and Neck	NO	NR	NR	NR	NR
Mattiuzzi, 2010	Adults & Elderly (18-99)	143	47.6	Hematological	NO	NR	NR	YES	NR
Keyhanian, 2009	Adults & Elderly (18-99)	138	63.2	Gastrointestinal, Breast, Hematological	Partial	NR	NR	NR	NR
Maemondo, 2009	Adults & Elderly (18-99)	231	26.8	Lung, Other	YES	NR	NR	NR	NR
Saito, 2009	Adults & Elderly (18-99)	1114	58.3	Lung, Breast, Other	Partial	NR	NR	NR	NR
Segawa, 2009	Adults & Elderly (18-99)	204	56.9	Lung, Breast, Lung, Other	NO	NR	NR	NR	NR
Yonemura, 2009	Adults & Elderly (18-99)	179	97.2	Breast , Gynecological, Gentiourinary, Sarcoma, Unknown/Other	NO	NR	NR	NR	NR
Yu, 2009	Adults (18-65)	208	36.5	Lung, Gastrointestinal, Breast, Head and Neck, Other	YES	NR	NR	NR	NR
Chen, 2008	All groups	424	36.0	Lung, Breast, Head and Neck, Genitourinary, Gastrointestinal, Other	YES	NR	NR	NR	NR
Fabi, 2008	Adults & Elderly (18-99)	89	85.5	Breast, Lung, Gynecological, Other	NO	NR	NR	NR	NR
Nagel, 2008	Children (≤18)	25	48.0	NR	NO	NR	NR	NR	NR
Sepúlveda-Vildósola, 2008	Children (≤18)	100	31.3	Sarcoma, Nervous System, Optic	Partial	NR	YES	YES	NR

Berrak, 2007	Adults & Children (0 - <65)	18	27.8	Optic	NO	NR	NR	NR	NR
Herrstedt, 2007	Aduts (18-65)	82	7.0	NR	YES	NR	NR	NR	NR
Kadota, 2007*	Children (≤18)	60	NR	NR	NR	NR	NR	NR	NR
Pectasides, 2007	Adults & Elderly (18-99)	134	100.0	Breast	NO	NR	NR	NR	NR
Shi, 2007	Adults & Elderly (18-99)	50	52.0	Lung, Breast, Gastrointestinal, Nervous System, Sarcoma, Head and Neck	Partial	NR	NR	NR	NR
Aapro, 2006	Adults & Elderly (18-99)	667	51.1	Gynecologic, Lung, Hematological, Gastrointestinal, Breast	YES	NR	NR	NR	NR
Pinarli, 2006	Adults & Children (0 - <65)	38	47.4	Sarcoma, Nervous System, Hematological, Germ Cell Tumor, Genitourinary	NO	NR	NR	NR	NR
Tabei, 2006†	NR	204	56.9	NR	NO	NR	NR	NR	NR
Buyukavci, 2005	Children (≤18)	22	13.6	Hematological	NO	NR	NR	NR	NR
Cheirsilpa, 2005	Adults & Elderly (18-99)	73	50.7	Head and Neck, Gynecological, Lung, Gastrointestinal, Genitourinary	YES	NR	NR	NR	NR
Corapcioglu, 2005	Children (≤18)	22	50.0	NR	Partial	NR	NR	NR	NR
Kurnianda, 2005	Adults (18-65)	39	21.0	Head and Neck, Lung, Head and Neck, Gynecological, Genitourinary	YES	NR	NR	YES	NR
Lindley, 2005	Adults & Elderly (18-99)	232	72.8	Breast, Lung, Hematological, Gynecological, Head and Neck, Genitourinary	YES	NR	NR	NR	NR
Mandanas, 2005	Adults & Elderly (18-99)	197	66.5	Breast	NO	NR	NR	NR	NR
Yano, 2005	Adults & Elderly (18-99)	39	35.9	Nervous System	NO	Radiotherapy	NR	NR	NR
Eisenberg, 2004	Adults & Elderly (18-99)	161	20.0	NR	YES	NR	NR	NR	NR
Jaing, 2004	Children (≤18)	66	36.4	Hematological	NO	NR	NR	NR	NR
Kim, 2004	Adults & Elderly (18-99)	105	37.0	Lung, Gastrointestinal, Gynecological, Head and Neck, Other	YES	NR	NR	NR	NR
Tantipalakorn, 2004	Adults (18-65)	109	100.0	Gynecologic	YES	NR	NR	NR	NR
Villalon, 2004	Adults & Elderly (18-99)	283	43.5	Head and Neck, Lung, Gynecological, Other	YES	NR	NR	NR	NR
Aapro, 2003	Adults & Elderly (18-99)	267	64.3	Breast, Lung, Gastrointestinal, Gentiourinary, Gynaecological, Head and Neck	Partial	NR	NR	NR	NR
Bhatia, 2003	Adults (18-65)	80	51.3	Head and Neck, Gynecological, Other	YES	NR	NR	NR	NR
Eisenberg, 2003	Adults (18-65)	569	82.1	Breast, Lung, Hematological, ,	Partial	NR	NR	NR	NR

Gralla, 2003	Adults & Elderly (18-99)	563	72.1	Breast , Lung, Gentiourinary, Gastrointestinal	Partial	NR	NR	NR	NR	
Koizumi, 2003	Adults & Elderly (18-99)	30	23.3	Gastrointestinal	YES	NR	NR	NR	NR	
Matsuoka, 2003	Adults & Children (0 to <65)	50	36.0	Hematological, Germ Cell Cancer	NO	Radiotherapy	NR	NR	NR	
Sagae, 2003	Adults (18-65)	138	100.0	Gynecologic	Partial	NR	NR	NR	NR	
Feng, 2002a	Adults & Elderly (18-99)	73	37.0	Lung, Hematological, Breast, Gastrointestinal, Genitourinary, Mediastinum (Cancer Of), Other	Partial	NR	NR	NR	NR	
Feng, 2002b	All groups	111	34.7	Lung, Breast, Hematological, Gastrointestinal, Head and Neck, Osteosarcoma, Other	Partial	NR	NR	NR	NR	
Kang, 2002	Adults & Elderly (18-99)	194	28.4	Gastrointestinal, Other	YES	NR	NR	NR	NR	
Noda, 2002	Adults & Elderly (18-99)	136	55.2	Lung, Gynecological, Gentiourinary, Gastrointestinal	YES	NR	NR	NR	NR	
Noda, 2002	Adults & Elderly (18-99)	84	46.4	Lung, Gastrointestinal, Gentiourinary, Gynecological	YES	NR	NR	YES	NR	
Aksoylar, 2001	Children (≤18)	51	37.3	Hematological, Sarcoma, Nervous System, Gentiourinary	NR	NR	NR	NR	NR	
de Wit, 2001	Adults & Elderly (18-99)	40	90.0	Breast, Gynecological, Lung	Partial	NR	NR	NR	NR	
Fox-Geiman, 2001	Adults (18-65)	102	71.6	NR	NO	NR	YES	YES	NR	
Koralewski, 2001	Adults & Elderly (18-99)	76	65.8	Breast, Gastrointestinal, Hematological, Lung	NO	NR	NR	NR	NR	
Ozgur Tan, 2001	Adults & Elderly (18-99)	25	0.0	Genitourinary	YES	NR	NR	NR	NR	
Parker, 2001	Children (≤18)	26	53.8	Hematological	NO	NR	NR	NR	NR	
Tsavaris, 2001a	Adults & Elderly (18-99)	106	13.2	Lung	YES	NR	NR	NR	NR	
Tsavaris, 2001b	Adults & Elderly (18-99)	100	0.0	Lung	YES	NR	NR	NR	NR	
Abang, 2000	Adults & Elderly (18-99)	60	65.0	Hematological, Breast	NR	NR	NR	NR	NR	
Barrajon, 2000	Adults & Elderly (18-99)	120	67.5	Breast, Lung, Head and Neck, Gynecological, Gastrointestinal, Other	Partial	NR	NR	NR	NR	
Chiou, 2000	All groups	51	37.3	Hematological, Unkown Pimary, Gentiourinary, Gastrointestinal, Breast, Lung (Nscl), Head And Neck	Partial	NR	NR	NR	NR	
Chua, 2000	Adults & Elderly (18-99)	89	13.5	Head and Neck	YES	Adjuvant/ Radiotherapy	NR	NR	NR	

Fauser , 2000	Aduts (18-65)	210	20.5	Genitourinary, Lung, Head and Neck, Gynecological, Gastrointestinal, Breast, Lymphoma	YES	NR	NR	NR	NR
Forni, 2000	Adults & Children (0 - <65)	90	31.1	Sarcoma	YES	NR	NR	NR	NR
Friedman, 2000	Adults & Elderly (18-99)	230	80.9	Breast	Partial	NR	NR	NR	NR
Herrington, 2000	Adults & Elderly (18-99)	61	75.4	Breast, Hematological, Other	YES	NR	NR	NR	NR
Hesketh, 2000	Adults & Elderly (18-99)	97	86.0	NR	NO	NR	NR	NR	NR
Koenig, 2000	Adults (18-65)	40	55.0	Gastrointestinal	NO	NR	NR	NR	NR
Oge, 2000	Adults (18-65)	106	35.9	Lung, Head and Neck, Gynecological, Genitourinary	YES	NR	NR	NR	NR
Raynov, 2000	Adults (18-65)	72	11.1	Lung, Genitourinary, Gynecological, Head and Neck	Partial	NR	NR	NR	NR
Roila [The Italian Group for Antiemetic Research], 2000	Adults & Elderly (18-99)	708	94.6	Breast, Other	NO	NR	YES	NR	NR
Slaby, 2000	Adults (18-65)	45	33.3	Hematological	NO	NR	NR	YES	NR
Stewart, 2000	Adults & Elderly (18-99)	16	0.6	NR	YES	NR	NR	NR	NR
White, 2000	Children (≤18)	428	41.6	Hematological, Sarcoma, Nervous System	Partial	NR	YES	NR	NR
Davidson, 1999	Adults & Elderly (18-99)	427	84.8	Breast, Hematological, Lung, Gynecological, Gastrointestinal, Bone And Soft Tissue, Gentrourinary	NO	NR	NR	NR	NR
Kandemir, 1999	Adults & Elderly (18-99)	89	57.3	Lung, Head and Neck, Gynecological, Genitourinary	YES	NR	NR	NR	NR
Komada, 1999	Children (≤18)	36	55.6	Hematological	NO	NR	NR	NR	NR
Lazarus, 1999	Adults (18-65)	43	47.0	Breast, Hematological, Gentiourinary	YES	NR	NR	NR	NR
Needles, 1999	All groups	357	32.2	Lung, Head and Neck, Gynecological, Gastrointestinal, Genitourinary, Other	YES	NR	NR	NR	NR
Orchard, 1999	Adults & Children (0 - <65)	187	42.8	Hematological, Breast	NR	Radiotherapy	NR	NR	NR
Raynov, 1999	Adults & Elderly (18-99)	35	40.0	Lung, Genitourinary, Gynecological	YES	NR	NR	NR	NR
Sandoval, 1999	Children (≤18)	31	48.4	Hematological, Nervous System	Partial	NR	NR	NR	NR
Sigsgaard, 1999	Adults & Elderly (18-99)	223	100.0	Breast	NO	Adjuvant/ Radiotherapy	NR	NR	NR

Stiakaki, 1999	Children (≤18)	23	18.2	Nervous System, Genitourinary, Hematological, Gynecological, Sarcoma	NO	NR	NR	NR	NR
Tatsumi, 1999	Adults (18-65)	186	37.1	Hematological	NO	NR	NR	NR	NR
The Italian Multicenter Study Group, 1999	Adults & Elderly (18-99)	532	33.5	Lung, Other	YES	NR	NR	NR	NR
Tsuchida, 1999	Children (≤18)	44	47.7	Nervous System, Gastrointestinal, Sarcoma, Optic	Partial	NR	NR	NR	NR
Uchida, 1999	Adults & Elderly (18-99)	29	24.1	Genitourinary	YES	NR	NR	NR	NR
Yalcn, 1999	Adults & Elderly (18-99)	54	98.2	Breast	NO	NR	NR	NR	NR
Birch, 1998	Adults (18-65)	70	100.0	Breast	NO	Adjuvant	NR	NR	NR
Gralla, 1998	Adults & Elderly (18-99)	1054	34.4	Lung, Genitourinary, Head and Neck, Unknown/Other	YES	NR	NR	NR	NR
Handberg, 1998	Adults & Elderly (18-99)	90	93.3	Gynecologic, Lung, Gentiourinary, Other	YES	NR	NR	NR	NR
Kalaycio, 1998	Adults (18-65)	45	100.0	Breast	NO	Adjuvant	NR	YES	NR
Kleisbauer, 1998	Adults & Elderly (18-99)	308	17.5	Lung, Head and Neck, Gynecological	YES	NR	NR	NR	NR
Krzakowsk, 1998	Adults & Elderly (18-99)	530	39.0	Lung, Gynecological, Head and Neck, Genitourinary, Gastrointestinal, Other	YES	NR	NR	YES	NR
Latreille, 1998	Adults (18-65)	435	60.0	Lung, Gynecological, Head and Neck, Other	YES	NR	YES	NR	NR
Martoni, 1998	Adults & Elderly (18-99)	198	18.0	Lung, Genitourinary, Other	Partial	NR	NR	NR	NR
Perez, 1998	Adults & Elderly (18-99)	1085	79.8	Breast, Hematological, Lung/Respiratory	NO	NR	NR	NR	NR
Perez, 1998	Adults & Elderly (18-99)	573	99.7	Breast	NO	NR	NR	NR	NR
Poon, 1998	Adults & Elderly (18-99)	20	100.0	Breast	NO	Adjuvant/ Radiotherapy	NR	NR	NR
Ritter, 1998	Adults & Elderly (18-99)	393	34.0	NR	YES	NR	NR	NR	NR
Scoponi, 1998	Adults & Elderly (18-99)	134	59.7	Breast, Gastrointestinal, Lung, Hematological, Other	NO	NR	NR	NR	NR
Sorbe, 1998	Adults & Elderly (18-99)	282	100.0	Gynecologic	Partial	NR	YES	NR	NR
Spector, 1998	Adults & Elderly (18-99)	371	44.5	Lung, Gynecological, Gentiourinary, Gastrointestinal, Head and Neck, Other	Partial	NR	NR	NR	NR

Tsavaris, 1998	Adults & Elderly (18-99)	104	36.5	Lung, Gynecological, Head and Neck, Genitourinary, Sarcoma, Mesothelioma, Other	YES	NR	NR	NR	NR
Zeidman, 1998	Adults (18-65)	58	29.3	Hematological, Gastrointestinal, Gentiourinary	Partial	NR	NR	NR	NR
Beck, 1997	Adults & Elderly (18-99)	402	90.1	Breast, Hematological, Lung, Gynecological, Genitourinary, Skin, Other	NO	NR	NR	NR	NR
Chevallier, 1997	Adults (18-65)	226	29.2	Breast, Gastrointestinal, Skin, Genitourinary, Gynecologic, Head And Neck, Musculoskeletal	YES	NR	NR	NR	NR
Davidson, 1997	Adults & Elderly (18-99)	406	86.0	Head and Neck, Lung, Breast, Gastrointestinal, Gynecologic, Haematopoietic/Immunological	NO	NR	NR	NR	NR
du Bois, 1997	Adults (18-65)	189	100.0	Gynecologic	NO	NR	Yes	Yes	NR
Fumoleau, 1997	Adults (18-65)	420	22.6	Lung, Head and Neck, Gastrointestinal, Genitourinary, Other	YES	NR	NR	NR	NR
Gridelli, 1997	Adults & Elderly (18-99)	230	37.8	NR	YES	NR	NR	NR	NR
Grote, 1997	Adults & Elderly (18-99)	307	46.3	Gastrointestinal, Gynecological, Head and Neck, Lung, Other	Partial	NR	NR	NR	NR
Kasimis, 1997	Adults & Elderly (18-99)	30	23.0	Lung, Other	YES	NR	NR	NR	NR
Kigawa, 1997	Adults & Elderly (18-99)	85	100.0	Gynecologic	YES	NR	NR	NR	NR
Kris, 1997	Adults & Elderly (18-99)	75	35.0	Lung, Gastrointestinal, Head and Neck, Unknown/Other	YES	NR	NR	YES	NR
Lofters, 1997	Adults & Elderly (18-99)	696	70.5	Breast, Lung, Gynecological, Other	Partial	NR	NR	NR	NR
Mustacchi, 1997	Adults & Elderly (18-99)	105	NR	NR	NR	NR	NR	NR	NR
Park, 1997	Adults & Elderly (18-99)	97	42.4	Head and Neck, Gastrointestinal, Breast, Gynecological, Sarcoma	Partial	NR	NR	NR	NR
Pectasides, 1997	Adults & Elderly (18-99)	80	43.8	Lung, Gynecological, Gentiourinary, Head and Neck, Melanoma, Other	YES	NR	NR	NR	NR
Perez, 1997	Adults & Elderly (18-99)	353	41.9	Lung, Head and Neck, Gynecological	YES	NR	NR	NR	NR
Roila [The Italian Group for Antiemetic Research], 1997	Adults & Elderly (18-99)	318	35.9	Gynecologic, Lung, Gentiourinary, Head and Neck, Other	YES	NR	YES	NR	NR
Rubenstein, 1997	Adults & Elderly (18-99)	320	81.3	Breast, Lung, Hematological, Other	NO	NR	NR	NR	NR
Advani, 1996	Adults & Elderly (18-99)	91	39.5	NR	YES	NR	NR	NR	NR

Audhuy, 1996	Adults & Elderly (18-99)	474	33.5	Head and Neck, Lung, Gastrointestinal, Gynecological	YES	NR	NR	NR	NR
Barrenetxea, 1996	Adults (18-65)	NR	100.0	Breast	NO	NR	NR	NR	NR
Brock, 1996	Children (≤18)	187	45.5	NR	Partial	NR	NR	NR	NR
Crucitt, 1996	Adults & Elderly (18-99)	133	90.2	Breast, Hematological, Lung	NO	NR	NR	NR	NR
de Wit, 1996	Adults & Elderly (18-99)	20	85.0	Breast, Lung, Hematological	NO	NR	NR	NR	NR
Fauser, 1996a	Adults & Elderly (18-99)	309	69.6	Breast, Hematological, Sarcoma, Lung, Other	NO	NR	NR	YES	NR
Fauser, 1996b	Adults & Elderly (18-99)	399	61.2	Breast, Lung	NO	NR	NR	NR	NR
Fujimoto, 1996	Children (≤18)	40	45.0	Nervous System, Optic, Gastrointestinal, Sarcoma	Partial	NR	NR	YES	NR
Hesketh, 1996b	Adults & Elderly (18-99)	609	38.1	Lung, Gastrointestinal, Gynecological, Head and Neck, Other	YES	NR	NR	YES	NR
Jorgensen, 1996	Adults & Elderly (18-99)	100	32.0	Hematological	NO	NR	NR	NR	NR
Leonardi, 1996	Adults & Elderly (18-99)	118	59.0	Breast, Lung, Hematological, Other,	Partial	NR	NR	NR	NR
Martoni, 1996	Adults & Elderly (18-99)	124	25.0	Lung, Genitourinary, Gynecological, Other	Partial	NR	NR	NR	NR
Massida & Ionta, 1996	Adults & Elderly (18-99)	60	100.0	Breast	NO	NR	YES	NR	NR
Matsui, 1996	Adults & Elderly (18-99)	70	22.9	Lung, Gastrointestinal	YES	NR	NR	NR	NR
Olver, 1996	Adults & Children (0 - <65)	604	37.8	Lung, Gynecological, Head and Neck, Gastrointestinal , Genitourinary	YES	NR	NR	NR	NR
Peterson, 1996	Adults & Elderly (18-99)	104	100.0	Gynecologic	YES	NR	YES	NR	NR
Yeilding, 1996	Adults & Elderly (18-99)	62	36.0	NR	YES	NR	NR	NR	NR
Adams, 1995	Adults (18-65)	126	86.5	NR	NO	NR	NR	NR	NR
Agura, 1995a	Adults (18-65)	30	36.7	Hematological	NO	Radiotherapy	NR	NR	NR
Agura, 1995b	Adults (18-65)	30	36.7	Hematological	NO	Radiotherapy	NR	NR	NR
Alfieri, 1995	Adults (18-65)	28	39.0	NR	YES	NR	NR	NR	NR
Alvarez, 1995	Children (≤18)	34	42.0	Sarcoma, CNS, Nervous System	Partial	NR	NR	YES	NR

Bleiberg, 1995	Adults & Elderly (18-99)	930	87.4	Breast, Gynecological, Lung, Hematological, Other	Partial	NR	NR	NR	NR
Bonneterre, 1995	Adults & Elderly (18-99)	150	90.0	NR	NO	Adjuvant/ Neoadjuvant	NR	NR	NR
Chiou, 1995	Adults & Elderly (18-99)	40	22.5	Lung, Gastrointestinal, Gentiourinary, Other	Partial	NR	NR	NR	NR
Contu, 1995	Adults & Elderly (18-99)	120	85.0	Breast, Lung, Gynecological, Sarcoma	NO	NR	NR	NR	NR
DiBenedetto, 1995	Adults (18-65)	81	86.4	Hematological, Lung, Sarcoma, Gynecological	NO	NR	NR	NR	NR
Dick, 1995	Children (≤18)	30	46.7	Hematological	NO	NR	NR	NR	NR
Fedele, 1995	Adults & Elderly (18-99)	25	NR	Hematological	NO	NR	NR	NR	HIV
Gebbia, 1995	Adults & Elderly (18-99)	262	30.9	Head and Neck, Lung, Gynecological, Breast, Genitourinary	YES	NR	NR	NR	NR
Hahlen, 1995	Children (≤18)	88	NR	Sarcoma	NO	NR	NR	NR	NR
Italian Group for Antiemetic Research, 1995	Adults & Elderly (18-99)	973	32.0	Gynecologic, Lung, Head and Neck, Genitourinary	YES	NR	NR	NR	NR
Labar, 1995	Adults (18-65)	52	44.2	Hematological	NO	Radiotherapy	NR	NR	NR
Latreille, 1995	Adults & Elderly (18-99)	292	64.0	Genitourinary, Lung, Gynecological, Head and Neck, Other	YES	NR	YES	YES	NR
Mantovani, 1995	Adults & Elderly (18-99)	117	3.4	Head and Neck	YES	NR	NR	NR	NR
Marty, 1995	Adults & Elderly (18-99)	231	28.6	Lung, Gastrointestinal, Gentiourinary, Other	Partial	NR	NR	NR	NR
Navari, 1995a	Adults & Elderly (18-99)	536	16.2	Lung, Head and Neck, Gastrointestinal, Genitourinary, Gynecologic, Other	YES	NR	NR	NR	NR
Navari, 1995b	Adults & Elderly (18-99)	987	36.5	Lung	YES	NR	NR	NR	NR
Stewart, 1995	Adults & Elderly (18-99)	488	100.0	Breast	NO	NR	NR	NR	NR
The Italian Group for Antiemetic Research, 1995	Adults & Elderly (18-99)	408	84.1	Gynecologic, Lung, Breast	NO	NR	NR	NR	NR
Tsavaris, 1995b	Adults & Elderly (18-99)	84	27.4	Lung, Gynecological, Head and Neck, Other	YES	NR	NR	NR	NR
Tsavaris, 1995a	Adults & Elderly (18-99)	80	32.5	Lung, Gynecological, Head and Neck, Sarcoma, Other	YES	NR	NR	NR	NR

Ahn, 1994	Adults & Elderly (18-99)	49	34.7	Gastrointestinal, Gynecological, Adenocariconma	YES	NR	NR	NR	NR
Carmichael, 1994	Adults & Elderly (18-99)	278	81.6	Breast, Other	Partial	NR	NR	NR	NR
Chevallier, 1994	Aduts (18-65)	101	NR	NR	YES	NR	NR	NR	NR
Chiu, 1994	Adults (18-65)	56	23.0	Hematological	NO	NR	NR	NR	NR
Cubeddu, 1994	Adults & Elderly (18-99)	324	86.4	Breast, Lung, Hematological	NO	NR	NR	NR	NR
Gebbia, 1994a	Adults & Elderly (18-99)	166	36.1	Head and Neck, Lung, Gentiourinary, Gynecological, Gastrointestinal, Endometrium, Vulva	YES	NR	NR	NR	NR
Gebbia, 1994b	Adults & Elderly (18-99)	158	79.1	Breast, Lung, Gynecological, Gastrointestinal, Hematological, Melanoma	NO	NR	NR	NR	NR
Heron, 1994	Adults & Elderly (18-99)	357	59.9	Gynecologic, Head and Neck, Lung, Genitourinary, Gastrointestinal	YES	NR	NR	NR	NR
Hesketh, 1994	Adults & Elderly (18-99)	275	28.0	Lung, Head and Neck, Gastrointestinal, Other	YES	NR	NR	NR	NR
Hulstaert, 1994	All groups	445	68.0	Breast, Lung, Head and Neck, Gynecological	Partial	NR	NR	NR	NR
Joss, 1994	Adults & Elderly (18-99)	207	42.5	Head and Neck, Lung, Gynecological, Genitourinary, Other	Partial	NR	NR	YES	NR
Kaizer, 1994	Adults & Elderly (18-99)	295	75.3	Breast, Lung, Gynecological, Other	Partial	NR	YES	NR	NR
Lim, 1994	Adults & Elderly (18-99)	55	43.6	Head and Neck, Gynecological, Lung, Gastrointestinal, Other	YES	NR	NR	NR	NR
Navari, 1994	Adults & Elderly (18-99)	184	30.4	Lung, Head and Neck	YES	NR	NR	NR	NR
Noble, 1994	Adults & Elderly (18-99)	359	22.5	Head and Neck, Lung, Gynecological, Genitourinary, Other	YES	NR	NR	NR	NR
Riviere, 1994	Adults & Elderly (18-99)	157	44.6	Head and Neck, Gynecological, Lung, Genitourinary, Melanoma, Gastrointestinal	YES	NR	NR	NR	NR
Ruff, 1994	Adults (18-65)	496	43.8	Gynecologic, Lung, Head and Neck, Genitourinary, Gastrointestinal, Bone And Soft Tissue	YES	NR	NR	NR	NR
Sorbe, 1994	Adults & Elderly (18-99)	63	100.0	Gynecologic	YES	NR	NR	YES	NR
Suarez, 1994	Children (≤18)	44	15.9	Hematological, Sarcoma, CNS, Optic	NO	NR	NR	NR	NR
Tsavaris, 1994	Adults & Elderly (18-99)	90	28.9	Lung, Gynecological, Head and Neck, Sarcoma, Other	YES	NR	NR	YES	NR
Tyson, 1994	Adults & Elderly (18-99)	44	34.1	Lung, Other	YES	NR	NR	NR	NR

Van Belle, 1994a	Adults & Elderly (18-99)	143	36.4	Lung, Genitourinary, Gynecological, Breast, Other	YES	NR	NR	NR	NR
Van Belle, 1994b	Adults & Elderly (18-99)	74	29.7	Lung, Genitourinary, Other	YES	NR	NR	NR	NR
Beck, 1993	Adults & Elderly (18-99)	349	84.5	Breast, Lung, Hematological, Other	NO	NR	NR	NR	NR
Bosi, 1993	Adults & Children (0 to <65)	40	52.5	Hematological, Other	NO	NR	NR	NR	NR
Buser, 1993	Adults & Elderly (18-99)	82	100.0	Breast	NO	Adjuvant/ Neoadjuvant	NR	NR	NR
Chevallier, 1993	Adults & Elderly (18-99)	281	34.9	NR	YES	NR	NR	NR	NR
Cunningham, 1993	Adults (18-65)	32	25.0	Hematological, Gastrointestinal, Sarcoma, Head and Neck, Gynecologic	Partial	NR	NR	NR	NR
Grunberg, 1993	Adults & Elderly (18-99)	125	32.0	Lung, Head and Neck, Gastrointestinal, Genitourinary, Gynecologic, Bone And Soft Tissue, Other	YES	NR	NR	NR	NR
Herrstedt, 1993	Aduts (18-65)	30	93.3	Breast	NO	Adjuvant	NR	NR	NR
Hunter, 1993	Adults (18-65)	284	18.0	NR	YES	NR	NR	NR	NR
Jantunen, 1993	Adults & Elderly (18-99)	166	83.7	Breast, Gastrointestinal, Hematological, Lung, Head and Neck, Mesothelioma, Other	Partial	NR	NR	NR	NR
Levitt, 1993	Adults & Elderly (18-99)	164	100.0	Breast	NO	NR	NR	NR	NR
Madej, 1993	All groups	115	83.5	Genitourinary, Head and Neck	YES	NR	NR	NR	NR
Rath, 1993	Adults & Elderly (18-99)	113	0.0	Genitourinary	YES	NR	NR	NR	NR
Smith (Granisetron Study Group), 1993	Adults (18-65)	504	66.1	NR	YES	NR	NR	NR	NR
Beck, 1992	Adults & Elderly (18-99)	699	33.5	Lung, Head and Neck, Gastrointestinal, Genitourinary, Gynecologic, Bone And Soft Tissue, Other	YES	NR	NR	NR	NR
Bots, 1992	Adults & Elderly (18-99)	125	52.0	Genitourinary, Lung, Breast, Hematological	Partial	NR	NR	NR	NR
Dicato, 1992	Adults (18-65)	324	72.2	Breast, Hematological, Lung	NO	NR	NR	NR	NR
Jantunen, 1992	Adults & Elderly (18-99)	47	70.2	Breast, Lung, Melanoma, Other	NO	NR	NR	NR	NR
Seynaeve, 1992	Adults & Elderly (18-99)	535	50.8	Head and Neck, Lung, Gastrointestinal, Genitourinary, Gynecologic, Bone And Soft Tissue, Other	YES	NR	NR	NR	NR

Sledge, 1992	Adults & Children (0 - <65)	46	0.0	Genitourinary, Adenocarcinoma	YES	Adjuvant	NR	NR	NR
Soukop, 1992	Adults & Elderly (18-99)	187	100.0	Breast	NO	Adjuvant	NR	NR	NR
Gandara, 1991	NR	274	84.1	NR	YES	NR	NR	NR	NR
Hainsworth, 1991	Adults & Elderly (18-99)	274	31.3	Head and Neck, Lung, Gastrointestinal, Gynecological, Genitourinary	YES	NR	NR	NR	NR
Jones, 1991	Adults & Elderly (18-99)	112	65.0	Lung, Breast, Gastrointestinal, Hematological, Other	NO	NR	NR	NR	NR
Marschner, 1991	Adults & Elderly (18-99)	122	100.0	NR	NO	NR	NR	NR	NR
Roila, 1991	Adults & Elderly (18-99)	102	43.3	Genitourinary, Lung, Head and Neck, Other	YES	NR	NR	NR	NR
Smith, 1991	Adults (18-65)	31	3.2	Germ Cell Tumor	YES	NR	NR	NR	NR
Smyth, 1991	Adults & Elderly (18-99)	100	47.0	NR	YES	NR	NR	NR	NR
Warr, 1991	Adults (18-65)	152	61.2	NR	Partial	NR	NR	NR	NR
Bonneterre, 1990	Adults & Elderly (18-99)	75	NR	NR	NO	Adjuvant	NR	NR	NR
Cubeddu, 1990a	Adults & Elderly (18-99)	20	85.0	Breast, Head and Neck, Sarcoma, Hematological	NO	NR	NR	NR	NR
Cubeddu, 1990b	Adults & Elderly (18-99)	28	71.4	Lung, Head and Neck, Gentiourinary	YES	NR	NR	NR	NR
Cupissol, 1990	Adults & Elderly (18-99)	28	28.6	NR	YES	NR	NR	NR	NR
De Mulder, 1990	Adults & Elderly (18-99)	121	56.2	Head and Neck, Lung, Gastrointestinal, Genitourinary, Gynecologic	YES	NR	NR	NR	NR
Falkson, 1990	NR	56	46.4	NR	Partial	NR	NR	NR	NR
Kaasa, 1990	Adults & Elderly (18-99)	93	79.6	Breast, Hematological, Other	NO	NR	NR	NR	NR
Khojasteh, 1990	Adults & Elderly (18-99)	36	19.4	Head and Neck, Lung, Gastrointestinal, Other	YES	NR	NR	NR	NR
Marty, 1990a	Adults & Elderly (18-99)	228	65.4	NR	Partial	NR	NR	NR	NR
Marty, 1990b	Adults & Elderly (18-99)	97	57.7	Gynecologic, Lung, Gastrointestinal, Genitourinary, Breast, Bone And Soft Tissue, Other	YES	NR	NR	NR	NR

Non-Randomized Control Trials (n=33)

Gabrail, 2015(a)	Aduts (18-65)	45	60	Ovarian, Breast, Lung, Lymphoma/leukemia, Endometrial/cervical/vulvar, Colorectal, Bladder	Partial	NR	NR	NR	NR
Kim JS, 2014	Adults & Elderly (18-99)	180	40.5	Colorectal	NO	NR	NR	YES	NR
Abali, 2007	Adults & Elderly (18-99)	158	72.8	Breast, Hematological, Lung, Other	Partial	NR	YES	YES	NR
Tan, 2004	Adults & Elderly (18-99)	26	61.5	Hematological, Lung, Head and Neck, Gynecological, Other	NR	NR	NR	NR	NR
Carmichael, 2003	Adults (18-65)	41	39.0	Lung, Gastrointestinal, Gynecological, Breast, Melanoma	Partial	NR	NR	NR	NR
Raynov, 2002	Adults (18-65)	101	45.5	Breast, Hematological	NO	NR	NR	NR	NR
Yamac, 2002	Adults & Elderly (18-99)	97	69.1	Breast, Lung, Gastrointestinal	Partial	NR	NR	NR	NR
Liaw, 2001	All groups	721	36.1	Lung, Breast, Head and Neck, Genitourinary, Gastrointestinal, Other	YES	NR	NR	NR	NR
Watters, 2001	Adults & Elderly (18-99)	274	63.9	Breast, Gastrointestinal, Gynecological, Head and Neck, Hematological, Lung	YES	NR	NR	NR	NR
Zeng, 2001	Aduts (18-65)	69	34.8	Hematological	NO	NR	NR	NR	NR
Coppes, 1999a	Children (≤18)	46	37.0	NR	NO	NR	NR	YES	NR
Coppes, 1999b	Children (≤18)	32	53.1	NR	NR	NR	NR	YES	NR
Tsavaris, 1999	Adults & Elderly (18-99)	76	78.9	Breast, Lung, Gynecological, Unknown/Other	NO	NR	NR	NR	NR
Kris, 1998	Adults & Elderly (18-99)	44	47.7	Lung, Sarcoma	YES	NR	NR	YES	NR
Chang, 1997	Adults & Elderly (18-99)	51	49.0	Hematological, Head and Neck, Gynecological, Genitourinary, Breast, Lung, Embryonic Carcinoma	Partial	NR	NR	NR	NR
Belkacemi, 1996	Aduts (18-65)	36	39.0	Hematological	NO	Radiotherapy	NR	NR	NR
Gaedicke, 1996	Children (≤18)	49	NR	Hematological, Sarcoma	NR	NR	NR	NR	NR
Hesketh, 1996a	Adults & Elderly (18-99)	69	71.0	Breast, Other	NO	NR	NR	NR	NR
Hesketh, 1995	Adults & Elderly (18-99)	146	63.0	Lung, Breast, Hematological, Other	NO	NR	NR	NR	NR
Conroy, 1994	Adults & Elderly (18-99)	164	40.2	Genitourinary, Head and Neck, Gastrointestinal, Lung, Breast, Bone, Other	YES	NR	NR	NR	NR
Kris, 1994	Adults & Elderly (18-99)	89	35.0	Lung, Head and Neck, Other	YES	NR	NR	NR	NR
Merrouche, 1994	Adults & Elderly (18-99)	25	60.0	Gynecologic, Head and Neck, Melanoma, Genitourinary, Gynecologic, Hematological	YES	NR	NR	NR	NR
de Wet, 1993	Adults & Elderly (18-99)	91	51.7	Breast, Gynecological, Gastrointestinal, Hematological, Genitourinary, Sarcoma, Unknown Primary	Partial	NR	NR	NR	NR
Pisters, 1993	Adults & Elderly (18-99)	22	36.4	Lung, Sarcoma	YES	NR	NR	NR	NR

Ribiero, 1993	Adults & Elderly (18-99)	145	53.1	NR	Partial	NR	NR	NR	NR
Dundee, 1992	Adults & Elderly (18-99)	233	NR	NR	NR	NR	NR	NR	NR
Fraschini, 1991	Adults & Elderly (18-99)	80	100.0	Breast	NO	Adjuvant	NR	NR	NR
Lemerle, 1991	Children (≤18)	24	29.0	Sarcoma, Hematological, Nervous System, Genitourinary	YES	NR	NR	NR	NR
Seynaeve, 1991	Adults & Elderly (18-99)	35	68.6	Hematological, Breast, Gynecological, Sarcoma, Other	NO	NR	NR	NR	NR
Addelman, 1990	NR	24	NR	NR	Partial	NR	NR	NR	NR
Cunningham, 1989	All groups	16	NR	NR	Partial	NR	NR	NR	NR
Grunberg, 1989	Adults & Elderly (18-99)	43	23.3	Lung, Germ Cell Cancer, Unknown Primary, Genitourinary, Head and Neck, Skin Cancer	YES	NR	NR	NR	NR
De Hann, 1988	Aduts (18-65)	13	NR	NR	YES	NR	NR	NR	NR

Cohort studies (n=19)

Anvari, 2015	Adults & Elderly (18-99)	117	86.9	Breast, Lymphoma, Others	NO	NR	NR	NR	NR
Zoto, 2015	Aduts (18-65)	155	54	Gastrointestinal, Breast, Lymphoma (Hodgkin and NHL), Lung, Genitourinary	NO	YES	YES	NR	NR
Murakami, 2014	Adults & Elderly (18-99)	46	30.43	Gastro-intestinal	NO	NR	NR	NR	NR
Keat, 2013	Aduts (18-65)	52	48.9	NR	NO	NR	YES	NR	NR
Blazer, 2012	Aduts (18-65)	305	47.0	Gastrointestinal	NO	NR	NR	NR	NR
Pielichowski, 2011	Aduts (18-65)	60	45.0	Hematological	NO	NR	NR	NR	NR
Taguchi, 2009	Adults & Elderly (18-99)	59	100.0	Breast	NO	NR	NR	NR	NR
Carreca, 2007*	NR	67	NR	Breast	NO	NR	NR	NR	NR
Hamadani, 2007	Adults & Elderly (18-99)	126	41.3	Lung, Head and Neck, Gastrointestinal, Genitourinary, Other	Partial	NR	NR	NR	NR
Adel, 2006*	Adults & Elderly (18-99)	176	42.6	NR	NR	NR	NR	NR	NR
Shikimori, 2006	Adults & Elderly (18-99)	63	41.3	Head and Neck	YES	Neoadjuvant	NR	NR	NR
Trifilio, 2006*	NR	69	NR	Myeloma	NO	NR	NR	NR	NR
Babaoglu, 2005	Adults (18-65)	216	75.0	Breast, Hematological, Lung, Other	Partial	NR	YES	NR	NR
Dempsey, 2004	Adults (18-65)	224	100.0	Breast	NO	Radiotherapy	NR	NR	NR
Steiner, 2003	Adults & Elderly (18-99)	62	71.0	Breast, Gynecological, Lung, Hematological, Melanoma	Partial	Radiotherapy	NR	NR	GI disorders
Brown, 1998	All groups	49	NR	Breast	NO	Adjuvant	NR	NR	NR

Blijham, 1992	Adults (18-65)	909	55.23	NR	Partial	NR	NR	NR	NR
Farley, 1997	Adults & Elderly (18-99)	119	74.79	Breast, Lung, Colon, Female Reproductive Organs, Lymphoma, Others	Partial	NR	NR	YES	NR
Harris, 1997	All groups	608	61.74	Breast, Gynecological, Lymphoma, Lung	Partial	NR	NR	YES	NR

Controlled Before and After study (n=1)

Rzepecki, 2009	NR	46	NR	Lymphoma, Relapsed Germ Cell Tumors, Acute Myeloid Leukemia	NR		NR	NR	NR

Abbreviations: CDDP – cisplatin; CINV – chemotherapy-induced nausea and vomiting; H/o, history of; NR - not reported; UK - United Kingdom; USA - United States of America
*****Includes Unpublished data (Adel 2006, Tabei 2006, Trifilio 2006, Carreca 2007, Kadota 2007, Piyush 2011)

Appendix D. Aggregate Cochrane EPOC risk-of-bias appraisal results

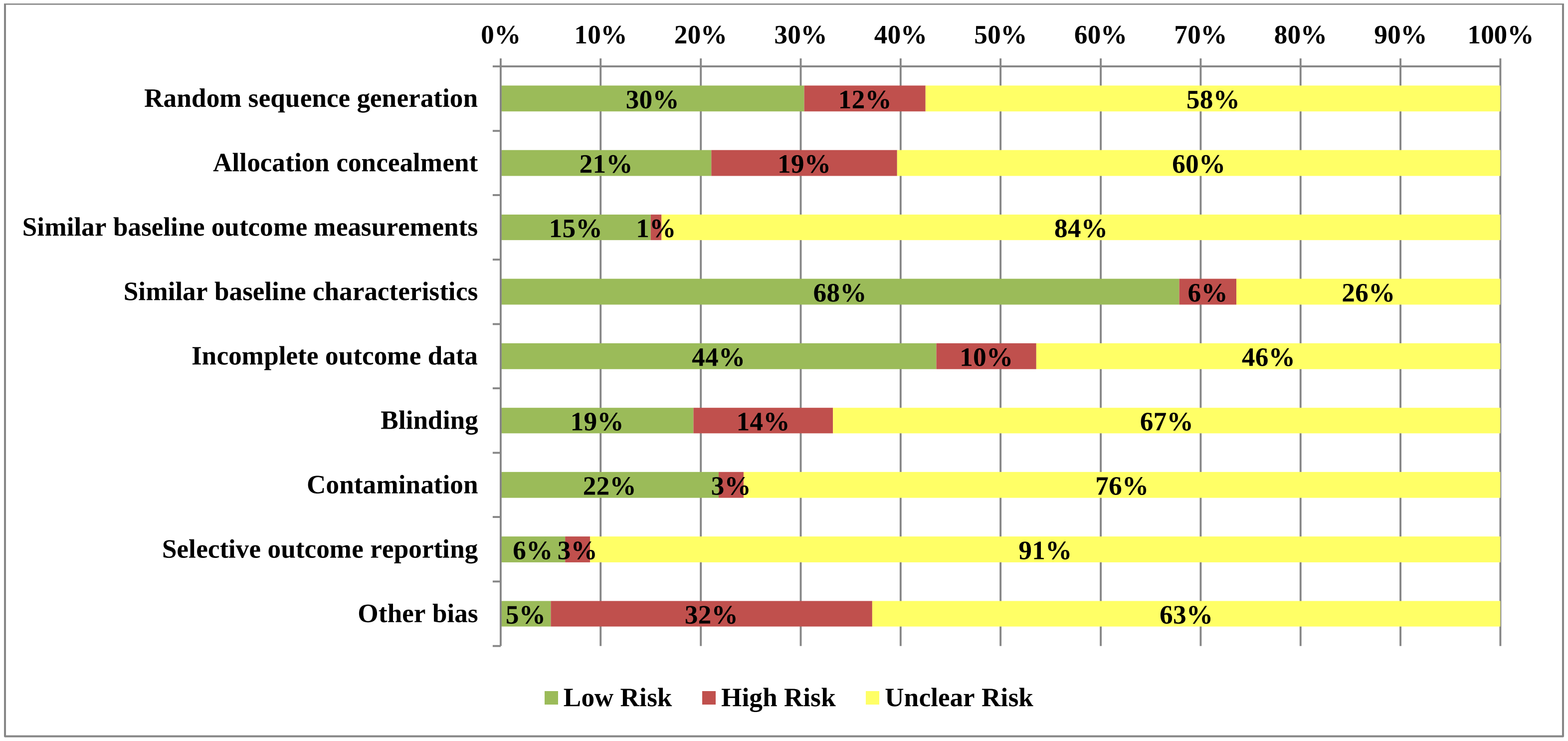

Abbreviations: EPOC - Effective Practice and Organisation of Care; High Risk - high risk of bias; Low Risk - low risk of bias; Unclear Risk - unclear risk of bias

Appendix E. Cochrane EPOC risk-of-bias appraisal results

Author, year	1	2	3	4	5	6	7	8	9
Randomized Clinical Trials (n=246)									
Kovacs, 2016	Low	Low	Unclear	High	Low	Low	High	Low	Unclear
Gabrail, 2015(b)	Low	Low	Low	Unclear	Low	Unclear	Unclear	Unclear	Unclear
Karthaus, 2015	Unclear	Unclear	Unclear	Low	Low	Unclear	High	Low	Unclear
Kim K, 2015	Low	Low	Unclear	High	Unclear	Unclear	High	Low	Unclear
Kim JE, 2015	Unclear	Unclear	Low	Low	High	Unclear	Unclear	Unclear	Unclear
Kimura, 2015	Unclear	Unclear	Unclear	High	Unclear	Unclear	High	Unclear	Unclear
Ohzawa, 2015	Low	Unclear	Unclear	High	Low	Unclear	High	Unclear	Unclear
Raftopolous, 2015	Unclear	Unclear	Unclear	High	Low	Unclear	High	Unclear	High
Sanmukhani, 2014	Unclear	Unclear	Low	Unclear	Unclear	Unclear	Unclear	Unclear	Unclear
Huang, 2013	Low	Low	Low	Unclear	Low	Unclear	Unclear	Unclear	Unclear
Wenzell, 2013	Unclear	Low	Low	High	Low	Unclear	Unclear	Unclear	Unclear
Boccia, 2013	Low	Unclear	Unclear	Low	Low	Unclear	Low	Unclear	Unclear
Endo, 2012	Low	Unclear	Unclear	Low	Unclear	Unclear	Unclear	Unclear	Unclear
Ithimakin, 2012	Low	Low	Low	Low	Low	Unclear	Unclear	Unclear	Low
Noor, 2012	Unclear	Unclear	Unclear	Low	Unclear	Unclear	Unclear	Unclear	Unclear
Roscoe, 2012	Low	Unclear	Unclear	Low	Unclear	Low	Low	Unclear	High
Tsuji, 2012	Low	Low	Unclear	Low	Low	Low	Low	Unclear	Low
Boccia, 2011	Low	Unclear	Unclear	Low	Low	Unclear	Unclear	High	Unclear
Dong, 2011	Unclear	Unclear	Low	Low	Unclear	Unclear	Unclear	Unclear	Unclear
Giralt, 2011	Unclear	Unclear	Low	Low	Unclear	Low	Low	Unclear	High
Piyush, 2011*	Unclear	Unclear	Unclear	Unclear	Unclear	Unclear	Unclear	Unclear	Unclear
Siddique, 2011	Unclear	Low	Unclear	Unclear	Unclear	Low	Unclear	Unclear	Unclear
Tian, 2011	Low	Low	Unclear	Low	Low	Low	Unclear	Low	Unclear
Ghosh, 2010	Unclear	Unclear	Unclear	Low	Low	Low	Low	Unclear	Unclear
Ho, 2010	Unclear	Unclear	Unclear	Low	Low	Unclear	Low	Unclear	Unclear
Kaushal, 2010	Low	High	Unclear	Unclear	Low	High	Unclear	Unclear	High
Mattiuzzi, 2010	Unclear	High	Unclear	Low	Low	Unclear	Low	Unclear	Unclear
Keyhanian, 2009	Low	Low	Unclear	Low	Low	Low	Unclear	Unclear	Unclear
Maemondo, 2009	Unclear	Unclear	Unclear	Low	Unclear	Low	Unclear	Unclear	High
Saito, 2009	Low	Low	Low	Low	Low	Low	Low	Low	Unclear
Segawa, 2009	Unclear	Unclear	Unclear	Low	Low	Unclear	Low	Unclear	High
Yonemura, 2009	Low	High	Unclear	High	Low	High	Unclear	High	Unclear
Yu, 2009	Unclear	Unclear	Unclear	Low	Low	Unclear	Unclear	Low	Unclear
Chen, 2008	High	Unclear	Low	Low	Low	Unclear	Unclear	Unclear	Unclear
Fabi, 2008	Unclear	Unclear	Unclear	Low	Unclear	Unclear	Low	Unclear	Unclear
Nagel, 2008	Unclear	Unclear	Unclear	Unclear	High	Unclear	Unclear	Unclear	High
Sepúlveda-Vildósola, 2008	Unclear	Unclear	Unclear	Low	Unclear	Low	Unclear	Unclear	Low
Berrak, 2007	Unclear	Low	Unclear	Unclear	High	Unclear	Unclear	Unclear	Unclear
Herrstedt, 2007	Low	Low	Unclear	Low	Low	Unclear	Unclear	Unclear	Unclear
Kadota, 2007*	Unclear	High	Unclear	Unclear	Unclear	Unclear	Unclear	Unclear	Unclear
Pectasides, 2007	Unclear	Unclear	Unclear	Low	Unclear	High	Unclear	Unclear	Unclear
Shi, 2007	Unclear	Unclear	Low	Low	Low	Unclear	Low	Low	Unclear
Aapro, 2006	Low	Unclear	Unclear	Unclear	Low	Unclear	Unclear	Unclear	Unclear
Pinarli, 2006	Unclear	Unclear	Unclear	Unclear	Unclear	Low	Unclear	Unclear	Unclear
Tabei, 2006*	Unclear	Unclear	Unclear	Unclear	Unclear	Unclear	Unclear	Unclear	Unclear
Buyukavci, 2005	Unclear	Unclear	Low	Low	Unclear	Low	Unclear	Unclear	Unclear
Cheirsilpa, 2005	Unclear	Low	Unclear	Low	Unclear	Unclear	Unclear	Unclear	Unclear
Corapcioglu, 2005	Unclear	High	Unclear	Unclear	Low	Low	Unclear	Unclear	Low
Kurnianda, 2005	Unclear	Low	Unclear	Low	Unclear	Low	Unclear	Unclear	Unclear
Lindley, 2005	Unclear	Low	Unclear	Low	Unclear	Low	Unclear	Unclear	Unclear
Mandanas, 2005	Unclear	High	Unclear	Low	Unclear	High	Unclear	Unclear	Unclear
Yano, 2005	Unclear	Unclear	Unclear	Low	Unclear	Unclear	Unclear	Unclear	Unclear
Eisenberg, 2004	Unclear	Unclear	Unclear	Low	Low	Unclear	Unclear	Unclear	High
Jaing, 2004	Unclear	High	Unclear	Low	Low	High	Unclear	Unclear	Unclear
Kim, 2004	Unclear	High	Unclear	Low	Unclear	High	Unclear	Unclear	Unclear
Tantipalakorn, 2004	Unclear	Unclear	Unclear	Unclear	Unclear	Unclear	Unclear	Unclear	Unclear
Villalon, 2004	Low	Unclear	Unclear	Low	Unclear	Unclear	Unclear	Unclear	Unclear
Aapro, 2003	Low	Unclear	Unclear	Low	Unclear	Unclear	Unclear	Low	Unclear

Bhatia, 2003	Unclear	Unclear	Unclear	Low	High	Unclear	Unclear	Unclear	Unclear
Eisenberg, 2003	Low	Unclear	Unclear	Low	Unclear	Unclear	Unclear	Unclear	Unclear
Gralla, 2003	Low	Low	Unclear	Low	Low	Low	Low	Unclear	High
Koizumi, 2003	Low	Low	Unclear	Low	Low	Unclear	Unclear	Unclear	Unclear
Matsuoka, 2003	Unclear	Unclear	Unclear	Low	Low	Unclear	Unclear	Unclear	Unclear
Sagae, 2003	Unclear	Unclear	Unclear	Low	Low	Unclear	Unclear	Unclear	Unclear
Feng, 2002a	Unclear	Unclear	Unclear	Unclear	Low	High	Unclear	Unclear	High
Feng, 2002b	Unclear	Unclear	Unclear	Low	Low	Unclear	Unclear	Unclear	Unclear
Kang, 2002	Low	Unclear	Unclear	Low	Low	High	Low	Unclear	Unclear
Noda, 2002a	Unclear	Unclear	Unclear	Low	Low	Unclear	Unclear	Unclear	Unclear
Noda, 2002b	Unclear	Low	Unclear	Low	Low	Low	Unclear	Unclear	Unclear
Aksoylar, 2001	Unclear	Unclear	Unclear	Low	Unclear	Unclear	Unclear	Unclear	Unclear
de Wit, 2001	Unclear	Low	Unclear	Low	Unclear	Unclear	Unclear	Unclear	Unclear
Fox-Geiman, 2001	Low	Low	Unclear	Low	Low	Low	Low	Unclear	Unclear
Koralewski, 2001	High	Unclear	Unclear	Unclear	Unclear	Unclear	Unclear	Unclear	Unclear
Ozgur Tan, 2001	Unclear	High	Unclear	Unclear	Unclear	High	Unclear	Unclear	Unclear
Parker, 2001	Unclear	Unclear	Low	Low	Low	Unclear	Unclear	Unclear	Unclear
Tsavaris, 2001a	Low	Unclear	Unclear	Low	Low	Unclear	Unclear	Unclear	Unclear
Tsavaris, 2001b	Low	Unclear	Unclear	Low	Unclear	Unclear	Unclear	Unclear	Unclear
Abang, 2000	Low	Unclear	Unclear	Low	Low	Unclear	Unclear	Unclear	Unclear
Barrajon, 2000	Low	Low	Unclear	High	High	Unclear	Unclear	Unclear	Unclear
Chiou, 2000	Unclear	High	Unclear	Low	Low	High	Unclear	Unclear	Unclear
Chua, 2000	Low	High	Unclear	Low	Low	High	Unclear	Unclear	Unclear
Fauser , 2000	Unclear	Low	Unclear	Low	Low	Unclear	Unclear	Unclear	Low
Forni, 2000	Unclear	Unclear	Unclear	Low	Unclear	Unclear	Unclear	Unclear	Unclear
Friedman, 2000	Unclear	Low	Unclear	Low	Low	Low	Low	Unclear	High
Herrington, 2000	Unclear	High	Unclear	Unclear	Low	High	Low	Unclear	High
Hesketh, 2000	Unclear	Unclear	Unclear	Low	Unclear	Unclear	Unclear	Unclear	Unclear
Koenig, 2000	Unclear	Unclear	Unclear	Unclear	Low	Unclear	Unclear	Unclear	High
Oge, 2000	Unclear	Unclear	Unclear	Unclear	Unclear	Unclear	Unclear	Unclear	Unclear
Raynov, 2000	Unclear	High	Unclear	Unclear	Unclear	High	Unclear	Unclear	High
Roila [The Italian Group for Antiemetic Research], 2000	Low	Low	Unclear	Low	Low	Low	Low	Low	High
Slaby, 2000	Unclear	Unclear	Unclear	Low	Low	Unclear	Low	Unclear	Low
Stewart, 2000	Low	Unclear	Unclear	Unclear	Unclear	Unclear	Unclear	Unclear	High
White, 2000	Low	Unclear	Unclear	Low	Unclear	Unclear	Unclear	Unclear	High
Davidson, 1999	Low	Unclear	Unclear	Low	High	Unclear	Unclear	Unclear	High
Kandemir, 1999	Unclear	Unclear	Low	Low	Unclear	Unclear	Unclear	Unclear	Unclear
Komada, 1999	Unclear	Unclear	Unclear	Low	Unclear	Unclear	Unclear	Unclear	Unclear
Lazarus, 1999	Low	Low	Unclear	Low	Unclear	Low	Unclear	High	Unclear
Needles, 1999	Unclear	Unclear	Low	Low	High	Unclear	Unclear	Unclear	High
Orchard, 1999	Unclear	Unclear	Unclear	Low	Low	Unclear	Unclear	Unclear	Low
Raynov, 1999	Unclear	Unclear	Unclear	Unclear	Low	Unclear	Low	Unclear	Unclear
Sandoval, 1999	Low	Low	Unclear	Low	Low	Unclear	Low	Unclear	Low
Sigsgaard, 1999	Unclear	Unclear	Unclear	Low	Low	Unclear	Low	Unclear	Unclear
Stiakaki, 1999	Unclear	Unclear	Unclear	Unclear	Unclear	Unclear	Low	Unclear	Unclear
Tatsumi, 1999	Unclear	Unclear	Unclear	Low	Unclear	Unclear	Unclear	Unclear	Unclear
The Italian Multicenter Study Group, 1999	Unclear	Unclear	Unclear	Low	Low	Unclear	Unclear	Unclear	Unclear
Tsuchida, 1999	Low	Unclear	Unclear	Low	Unclear	Unclear	Unclear	Unclear	Unclear
Uchida, 1999	Low	Unclear	Unclear	Low	Unclear	Low	Low	Unclear	Unclear
Yalcn, 1999	Unclear	Unclear	Unclear	Unclear	Unclear	Unclear	Unclear	Unclear	Unclear
Birch, 1998	Unclear	Unclear	Unclear	Low	Unclear	Unclear	Unclear	Unclear	Unclear
Gralla, 1998	Unclear	Low	Low	Low	Low	Low	Low	Unclear	High
Handberg, 1998	Unclear	Low	Unclear	Low	Low	Unclear	Unclear	Unclear	Unclear
Kalaycio, 1998	Low	Low	Unclear	Low	High	Low	Low	Unclear	Unclear
Kleisbauer, 1998	Unclear	Unclear	Unclear	Low	Low	Unclear	Unclear	Unclear	Unclear
Krzakowski, 1998	Low	Low	Unclear	Low	Unclear	Low	Unclear	Unclear	High
Latreille, 1998	Low	Low	Unclear	Low	Low	Low	Unclear	Unclear	High
Martoni, 1998	Unclear	Unclear	Unclear	Unclear	High	Unclear	Unclear	Low	Low
Perez, 1998	Unclear	Unclear	Unclear	Low	Low	Unclear	Unclear	Unclear	High
Perez, 1998	Low	Low	Unclear	Low	Low	Low	Low	Unclear	Unclear
Poon, 1998	Unclear	Unclear	Unclear	Unclear	Unclear	Unclear	Unclear	Unclear	Unclear
Ritter, 1998	Unclear	Unclear	Low	Low	Unclear	Unclear	Unclear	Unclear	High
Scoponi, 1998	Unclear	unclear	Unclear	Unclear	Low	Unclear	Unclear	High	Unclear
Sorbe, 1998	Unclear	Unclear	Low	Low	Low	Unclear	Low	Unclear	Unclear

Spector, 1998	Low	Unclear	Low	Low	Low	Low	Low	High	Unclear
Tsavaris, 1998	Low	Unclear	Unclear	Low	Unclear	Unclear	Unclear	Unclear	Unclear
Zeidman, 1998	Unclear	Unclear	Unclear	low	Unclear	Unclear	Unclear	Unclear	Unclear
Beck, 1997	Unclear	Unclear	High	Low	Unclear	Unclear	Unclear	Unclear	Unclear
Chevallier, 1997	Unclear	Low	Low	Low	Low	Unclear	Unclear	Unclear	High
Davidson, 1997	Low	Unclear	Unclear	Low	High	Unclear	Unclear	Unclear	High
du Bois, 1997	Low	Unclear	Low	Low	Unclear	Unclear	Unclear	Unclear	Unclear
Fumoleau, 1997	Unclear	Unclear	Unclear	Low	Low	Low	Low	Unclear	High
Gridelli, 1997	Unclear	Low	Low	Low	High	Unclear	Unclear	Unclear	Unclear
Grote, 1997	Unclear	Unclear	Low	Low	Low	Unclear	Unclear	Unclear	Unclear
Kasimis, 1997	Unclear	Unclear	Unclear	Low	Unclear	Unclear	Unclear	Unclear	High
Kigawa, 1997	Unclear	Unclear	Unclear	Unclear	Unclear	Unclear	Unclear	Unclear	High
Kris, 1997	Unclear	Unclear	Unclear	Low	Unclear	Unclear	Unclear	Unclear	High
Lofters, 1997	Unclear	Low	Unclear	Low	Unclear	Low	Unclear	Unclear	High
Mustacchi, 1997	Unclear	Unclear	High	Unclear	Unclear	High	Unclear	Unclear	Unclear
Park, 1997	Unclear	High	Unclear	Low	Low	High	Unclear	Unclear	Unclear
Pectasides, 1997	Low	Unclear	Unclear	Low	Unclear	Unclear	Unclear	Unclear	Unclear
Perez, 1997	Unclear	Unclear	Unclear	Low	Unclear	Unclear	Unclear	Unclear	Unclear
Roila [The Italian Group for Antiemetic Research], 1997	Low	Unclear	Low	Low	Low	Unclear	Low	Unclear	Unclear
Rubenstein, 1997	Unclear	Unclear	Low	Low	Unclear	Unclear	Unclear	Unclear	High
Advani, 1996	Unclear	Low	Unclear	Low	Low	Low	Unclear	Unclear	High
Audhuy, 1996	Unclear	Unclear	Unclear	Low	Low	Unclear	Unclear	Unclear	Unclear
Barrenetxea, 1996	Unclear	Unclear	Unclear	Unclear	Unclear	Unclear	Unclear	Unclear	Unclear
Brock, 1996	Unclear	Unclear	Unclear	Unclear	Unclear	Unclear	Low	Unclear	High
Crucitt, 1996	Unclear	Unclear	Unclear	Low	Low	Unclear	Unclear	Unclear	High
de Wit, 1996	Unclear	Unclear	Low	Low	Unclear	Unclear	Unclear	Unclear	Unclear
Fauser, 1996a	Unclear	Low	Low	Low	Unclear	Low	Low	Unclear	Unclear
Fauser, 1996b	Unclear	Low	Unclear	Low	Low	Low	Low	Unclear	Unclear
Fujimoto, 1996	Unclear	Unclear	Unclear	Unclear	Unclear	Unclear	Unclear	Unclear	Unclear
Hesketh, 1996b	Unclear	Low	Low	Low	Low	Low	Low	Unclear	Unclear
Jorgensen, 1996	Low	High	Unclear	Low	Low	High	Unclear	Unclear	Unclear
Leonardi, 1996	Unclear	Unclear	Unclear	Unclear	Low	Unclear	Low	Unclear	Unclear
Martoni, 1996	Unclear	High	Unclear	Unclear	Unclear	High	Unclear	Unclear	Unclear
Massida & Ionta, 1996	Unclear	Unclear	Low	Low	Low	Unclear	Unclear	Low	Unclear
Matsui, 1996	Unclear	Unclear	Unclear	Low	Low	Unclear	Unclear	Unclear	Unclear
Olver, 1996	Low	Low	Unclear	Low	Unclear	Unclear	Unclear	Unclear	High
Peterson, 1996	Unclear	Unclear	Unclear	Unclear	Low	Unclear	Unclear	Unclear	Unclear
Yeilding, 1996	Unclear	Unclear	Unclear	Unclear	Unclear	Unclear	Unclear	Unclear	Unclear
Adams, 1995	Unclear	Unclear	Unclear	Low	High	Unclear	Unclear	Unclear	High
Agura, 1995a	Low	Unclear	Unclear	Low	High	High	Unclear	Unclear	High
Agura, 1995b	Low	High	Unclear	High	High	High	Unclear	Unclear	Unclear
Alfieri, 1995	Unclear	Unclear	Unclear	High	Unclear	Unclear	Unclear	Unclear	Low
Alvarez, 1995	Low	Low	Unclear	Unclear	Low	Low	Low	Unclear	Unclear
Bleiberg, 1995	Unclear	Unclear	Unclear	Low	Unclear	Unclear	Unclear	Unclear	Unclear
Bonneterre, 1995	Unclear	High	Unclear	Low	High	Unclear	Unclear	Unclear	Unclear
Chiou, 1995	Unclear	Unclear	Unclear	Low	Unclear	Unclear	Unclear	Unclear	Unclear
Contu, 1995	Unclear	Unclear	Unclear	Low	Low	Unclear	Low	Unclear	High
DiBenedetto, 1995	Unclear	Unclear	Unclear	Low	High	Unclear	Unclear	Unclear	High
Dick, 1995	Unclear	Unclear	Unclear	Unclear	Unclear	Unclear	Unclear	Unclear	Unclear
Fedele, 1995	Unclear	Unclear	Unclear	Unclear	Unclear	Unclear	Unclear	Unclear	Unclear
Gebbia, 1995	Unclear	High	Low	Low	Low	Unclear	Unclear	Unclear	Unclear
Hahlen, 1995	Low	Unclear	Unclear	Unclear	Unclear	Unclear	Unclear	Unclear	High
Italian Group for Antiemetic Research, 1995	Low	Unclear	Unclear	Low	Low	Unclear	Unclear	Unclear	Unclear
Labar, 1995	Unclear	Unclear	Unclear	Low	Unclear	Unclear	Unclear	Unclear	Unclear
Latreille, 1995	Low	Low	Unclear	Low	Unclear	Low	Unclear	Unclear	High
Mantovani, 1995	Unclear	High	Unclear	Low	Unclear	High	Unclear	Unclear	Unclear
Marty, 1995	Low	Unclear	Unclear	Low	Unclear	Unclear	Low	Low	High
Navari, 1995a	Low	Unclear	Unclear	Low	High	Unclear	Unclear	Unclear	High
Navari, 1995b	Unclear	Unclear	Unclear	Low	High	Unclear	Unclear	Unclear	High
Stewart, 1995	Low	Low	Unclear	Low	Low	Low	Low	Unclear	High
The Italian Group for Antiemetic Research, 1995	Low	Unclear	Unclear	Low	Low	Unclear	Unclear	Unclear	Unclear
Tsavaris, 1995a	Unclear	Unclear	Unclear	Low	Unclear	Unclear	Unclear	Unclear	Unclear
Tsavaris, 1995b	Unclear	Unclear	Unclear	Low	Unclear	Low	Unclear	Unclear	Unclear
Ahn, 1994	Unclear	Low	Unclear	Low	Low	Unclear	Unclear	Unclear	Unclear

Carmichael, 1994	Low	Unclear	Unclear	High	Unclear	Unclear	Low	Unclear	High
Chevallier, 1994	Unclear	Unclear	Unclear	Low	Low	Unclear	Low	Unclear	Unclear
Chiu, 1994	Unclear	Unclear	Unclear	Unclear	Unclear	Unclear	Unclear	Unclear	Unclear
Cubeddu, 1994	Low	Low	Unclear	High	Unclear	Unclear	Unclear	Unclear	High
Gebbia, 1994a	Unclear	High	Unclear	Low	Low	Unclear	Low	Unclear	Unclear
Gebbia, 1994b	Unclear	High	Unclear	Low	Low	Unclear	Low	Unclear	Unclear
Heron, 1994	Unclear	Unclear	Unclear	Low	Unclear	Low	Unclear	Unclear	High
Hesketh, 1994	Low	Low	Unclear	Low	Low	Low	Unclear	Unclear	High
Hulstaert, 1994	Low	Unclear	Unclear	High	Unclear	Unclear	Unclear	Unclear	High
Joss, 1994	Low	Low	Unclear	Low	Low	Low	Unclear	Unclear	Unclear
Kaizer, 1994	Low	Low	Unclear	Low	Low	Unclear	Unclear	Unclear	High
Lim, 1994	Unclear	High	Unclear	Low	Unclear	High	Unclear	Unclear	High
Navari, 1994	Unclear	Unclear	Unclear	Unclear	Unclear	Unclear	Unclear	Unclear	Unclear
Noble, 1994	Unclear	Unclear	Unclear	Low	Low	Unclear	Unclear	Unclear	High
Riviere, 1994	Unclear	Unclear	Unclear	Low	Low	Unclear	Unclear	Unclear	Unclear
Ruff, 1994	Unclear	Unclear	Low	Low	Low	Unclear	Low	Low	High
Sorbe, 1994	Unclear	Unclear	Unclear	Low	Low	Low	Unclear	Unclear	High
Suarez, 1994	Unclear	Low	Unclear	Low	High	Low	Unclear	Unclear	Unclear
Tsavaris, 1994	Unclear	Unclear	Unclear	Low	Unclear	Unclear	Unclear	Unclear	Unclear
Tyson, 1994	Low	Unclear	Low	Low	Low	Unclear	Unclear	Unclear	Unclear
Van Belle, 1994a	Low	Unclear	Unclear	Unclear	Unclear	Unclear	Unclear	Unclear	Unclear
Van Belle, 1994b	Low	Unclear	Unclear	Unclear	Unclear	Unclear	Unclear	Unclear	Unclear
Beck, 1993	Unclear	Unclear	Low	Low	High	Unclear	Unclear	Unclear	Unclear
Bosi, 1993	Low	Unclear	Unclear	Low	Unclear	Low	Unclear	Unclear	Low
Buser, 1993	Unclear	Unclear	Unclear	Low	Unclear	Unclear	Unclear	Unclear	High
Chevallier, 1993	Low	High	Unclear	Low	Unclear	High	Unclear	Unclear	Low
Cunningham, 1993	Unclear	Unclear	Unclear	Low	Unclear	Unclear	Unclear	Unclear	High
Grunberg, 1993	Low	Low	Unclear	Low	Low	Low	Low	Unclear	High
Herrstedt, 1993	Unclear	Low	Unclear	Unclear	Low	Unclear	Unclear	Unclear	Low
Hunter, 1993	Unclear	Low	Unclear	Low	Unclear	Unclear	Low	Unclear	High
Jantunen, 1993	Unclear	High	Unclear	Low	Unclear	High	Unclear	Unclear	Unclear
Levitt, 1993	Low	Low	Low	Low	Unclear	Low	Unclear	Unclear	Unclear
Madej, 1993	Unclear	Unclear	Unclear	Low	Unclear	Unclear	Unclear	Unclear	Unclear
Rath, 1993	Unclear	Unclear	Unclear	Low	Unclear	Unclear	Unclear	Unclear	High
Smith (Granisetron Study Group), 1993	Unclear	Unclear	Unclear	Low	Low	Unclear	Low	Unclear	Unclear
Beck, 1992	Low	Unclear	Unclear	Low	Unclear	Unclear	Unclear	Unclear	Unclear
Bots, 1992	Unclear	Unclear	Unclear	Low	Unclear	Unclear	Unclear	Unclear	High
Dicato, 1992	Low	Unclear	Unclear	Low	Low	Unclear	Unclear	Unclear	High
Jantunen, 1992	Unclear	Unclear	Unclear	Low	Low	Unclear	Unclear	Unclear	Unclear
Seynaeve, 1992	Low	Unclear	Low	Low	Low	Unclear	Low	Unclear	High
Sledge, 1992	Low	Unclear	Unclear	Low	Low	Unclear	Low	Unclear	High
Soukop, 1992	Low	Unclear	Unclear	Low	Unclear	Low	Unclear	Unclear	Unclear
Gandara, 1991	Unclear	Unclear	Unclear	Unclear	Unclear	Unclear	Low	Unclear	Unclear
Hainsworth, 1991	Low	Low	Low	Low	Low	Low	Low	Unclear	High
Jones, 1991	Low	Low	Unclear	Low	High	Low	Unclear	Unclear	High
Marschner, 1991	Unclear	Low	Low	Low	Low	Unclear	Unclear	Low	Unclear
Roila, 1991	Unclear	Unclear	Unclear	Low	Unclear	Unclear	Unclear	Unclear	Unclear
Smith, 1991	Unclear	Unclear	Unclear	Unclear	Unclear	Unclear	Unclear	Unclear	High
Smyth, 1991	Low	Low	Unclear	Low	Low	Unclear	Unclear	Unclear	High
Warr, 1991	Low	Unclear	Unclear	Unclear	High	Unclear	Unclear	Unclear	Unclear
Bonneterre, 1990	Unclear	Unclear	Unclear	Unclear	High	Unclear	Unclear	Unclear	High
Cubeddu, 1990a	Unclear	Unclear	Unclear	Low	Unclear	Low	Unclear	Unclear	High
Cubeddu, 1990b	Low	Unclear	Unclear	Low	Unclear	Unclear	Unclear	Unclear	High
Cupissol, 1990	Unclear	Unclear	Unclear	Unclear	Unclear	Unclear	Unclear	Unclear	Unclear
De Mulder, 1990	Low	Low	Unclear	Low	Unclear	Unclear	Low	Unclear	High
Falkson, 1990	Unclear	Unclear	Unclear	Unclear	Unclear	Unclear	Unclear	Unclear	Unclear
Kaasa, 1990	Unclear	Unclear	Unclear	Low	Low	Unclear	Low	Unclear	High
Khojasteh, 1990	Low	High	Unclear	Low	High	High	Unclear	Unclear	High
Marty, 1990a	Unclear	Unclear	Unclear	Low	Unclear	Unclear	Unclear	Low	Unclear

Non-Randomized Controlled Trials (n=33)

Gabrail, 2015(a)	High	Unclear	Unclear	High	High	Unclear	Unclear	Unclear	Unclear
Kim JS, 2014	Unclear	Unclear	Low	Low	Low	High	Unclear	Unclear	Unclear
Abali, 2007	High	High	Unclear	Unclear	Unclear	High	Unclear	Unclear	Unclear
Tan, 2004	High	High	Unclear	Low	Low	High	Low	Unclear	High

Carmichael, 2003	Unclear	Unclear	Unclear	Unclear	Low	Unclear	Unclear	Unclear	High
Raynov, 2002	High	Unclear	Unclear	Unclear	Low	High	Unclear	Unclear	High
Yamac, 2002	High	High	Unclear	Unclear	Unclear	Unclear	Unclear	Unclear	Unclear
Liaw, 2001	High	High	Unclear	Unclear	Unclear	High	Low	Low	Unclear
Watters, 2001	High	Unclear	Unclear	Low	High	Unclear	Unclear	Unclear	Unclear
Zeng, 2001	High	High	Unclear	Unclear	Unclear	Unclear	Unclear	Unclear	Unclear
Coppes, 1999a	High	High	Unclear	Unclear	Unclear	Unclear	Unclear	Unclear	High
Coppes, 1999b	High	High	Low	Low	Unclear	Unclear	Unclear	Unclear	High
Tsavaris, 1999	High	High	High	Unclear	Unclear	High	Unclear	Unclear	Unclear
Kris, 1998	High	High	Unclear	Unclear	Low	Unclear	Low	Unclear	Unclear
Chang, 1997	High	High	Unclear	Unclear	Low	Unclear	Unclear	Unclear	Low
Belkacemi, 1996	High	High	Unclear	Unclear	Low	Unclear	Unclear	Unclear	Unclear
Gaedicke, 1996	High	High	Low	Low	High	Low	Low	Unclear	High
Hesketh, 1996a	High	High	Low	Low	Low	High	Low	Unclear	High
Hesketh, 1995	High	High	Unclear	High	Low	High	Unclear	Unclear	High
Conroy, 1994	High	High	Unclear	High	Unclear	High	Unclear	Unclear	High
Kris, 1994	High	High	Unclear	Unclear	Unclear	Unclear	Unclear	Unclear	Unclear
Merrouche, 1994	High	High	Unclear	Unclear	Low	Unclear	Low	Low	High
de Wet, 1993	High	High	Unclear	Unclear	Unclear	High	Unclear	Unclear	Unclear
Pisters, 1993	High	Unclear	Unclear	Unclear	Low	Low	Unclear	Unclear	Unclear
Ribiero, 1993	High	High	Unclear	High	Unclear	Unclear	Low	Unclear	High
Dundee, 1992	High	High	Unclear	Unclear	Unclear	High	Unclear	Unclear	Unclear
Fraschini, 1991	High	High	Low	Low	Low	High	Unclear	Unclear	High
Lemerle, 1991	High	High	Unclear	Unclear	Unclear	High	Unclear	Unclear	Unclear
Seynaeve, 1991	High	High	Unclear	Unclear	Low	Unclear	Unclear	Unclear	High
Addelman, 1990	High	Unclear	Unclear	Unclear	Low	Unclear	Unclear	Unclear	Unclear
Cunningham, 1989	High	High	Unclear	Unclear	Unclear	High	Unclear	Unclear	Unclear
Grunberg, 1989	High	High	Low	Low	Low	Unclear	Low	Unclear	Unclear
De Hann, 1988	High	Unclear	Unclear	Unclear	Unclear	Unclear	Unclear	High	High
Controlled Before and After Study (n=1)									
Rzepecki, 2009	High	High	Unclear	Unclear	Unclear	High	Unclear	High	Unclear

Abbreviations: EPOC - Effective Practice and Organisation of Care; High - high risk of bias; Low - low risk of bias; Unclear - unclear risk of bias

* Includes unpublished data (Tabei 2006, Kadota 2007, Piyush 2011)

Items:

1. Random sequence generation
2. Allocation concealment
3. Similar baseline outcome measures
4. Similar baseline characteristics
5. Incomplete outcome data
6. Blinding of outcome assessor
7. Contamination
8. Selective outcome reporting
9. Other bias

Appendix F. Newcastle-Ottawa Scale Appraisal Results

Author, year	1	2	3	4	5	6	7
Anvari, 2015	A	A	A	B	A	D	D
Zoto, 2015	B	A	A	A	B	C	C
Murakami, 2014	B	A	A	B	C	C	B
Keat, 2013	B	A	A	B	A	C	C
Blazer, 2012	B	A	A	B	B	A	D
Pielichowski, 2011	C	A	D	B	A	D	D
Taguchi, 2009	B	A	A	B	C	B	D
Carreca, 2007*	B	A	D	B	C	C	D
Hamadani, 2007	C	A	A	B	C	B	B
Adel, 2006*	B	A	A	B	C	A	D
Shikimori, 2006	A	A	D	B	A	D	D
Trifilio, 2006*	B	A	A	B	C	A	D
Babaoglu, 2005	A	A	A	B	C	C	C
Dempsey, 2004	B	C	A	B	A	A	D
Steiner, 2003	C	A	A	A	A	B	A
Brown, 1998	C	A	A	B	C	B	D
Farley, 1997	B	A	C	B	A	B	D
Harris, 1997	A	A	A	B	A	D	A
Blijham, 1992	A	C	A	B	C	D	D

* Includes unpublished data (Adel 2006, Trifilio 2006, Carreca 2007)

Items:

1. Representativeness of the exposed cohort: A - truly representative of the average participant undergoing chemotherapy; B - somewhat representative of the average participant undergoing chemotherapy; C - selected group of users
2. Selection of the non-exposed cohort: A - drawn from the same community as the exposed cohort; C - no description of the derivation of the non-exposed cohort
3. Ascertainment of exposure: A - secure record; C - written self report; D - no description
4. Demonstration that outcome of interest was not present at start of study: A - yes; B - no
5. Comparability of cohorts on the basis of the design or analysis: A - study controls for most important factors (e.g., age, gender); B - study controls for any additional factor; C - nothing is controlled for
6. Assessment of outcome: A – independent blind assessment; B – record linkage; C – self-report; D – no description
7. Adequacy of follow up of cohorts: A – complete follow up, all subjects accounted for; B - subjects lost to follow up unlikely to introduce bias - small number lost (<10%) and description of those lost; C - large number lost (>10%) and no description of those lost; D – no statement

Appendix G. Comparison Adjusted Funnel Plots for patients without nausea, patients without vomiting, patients without CINV, and patients experiencing severe vomiting outcomes

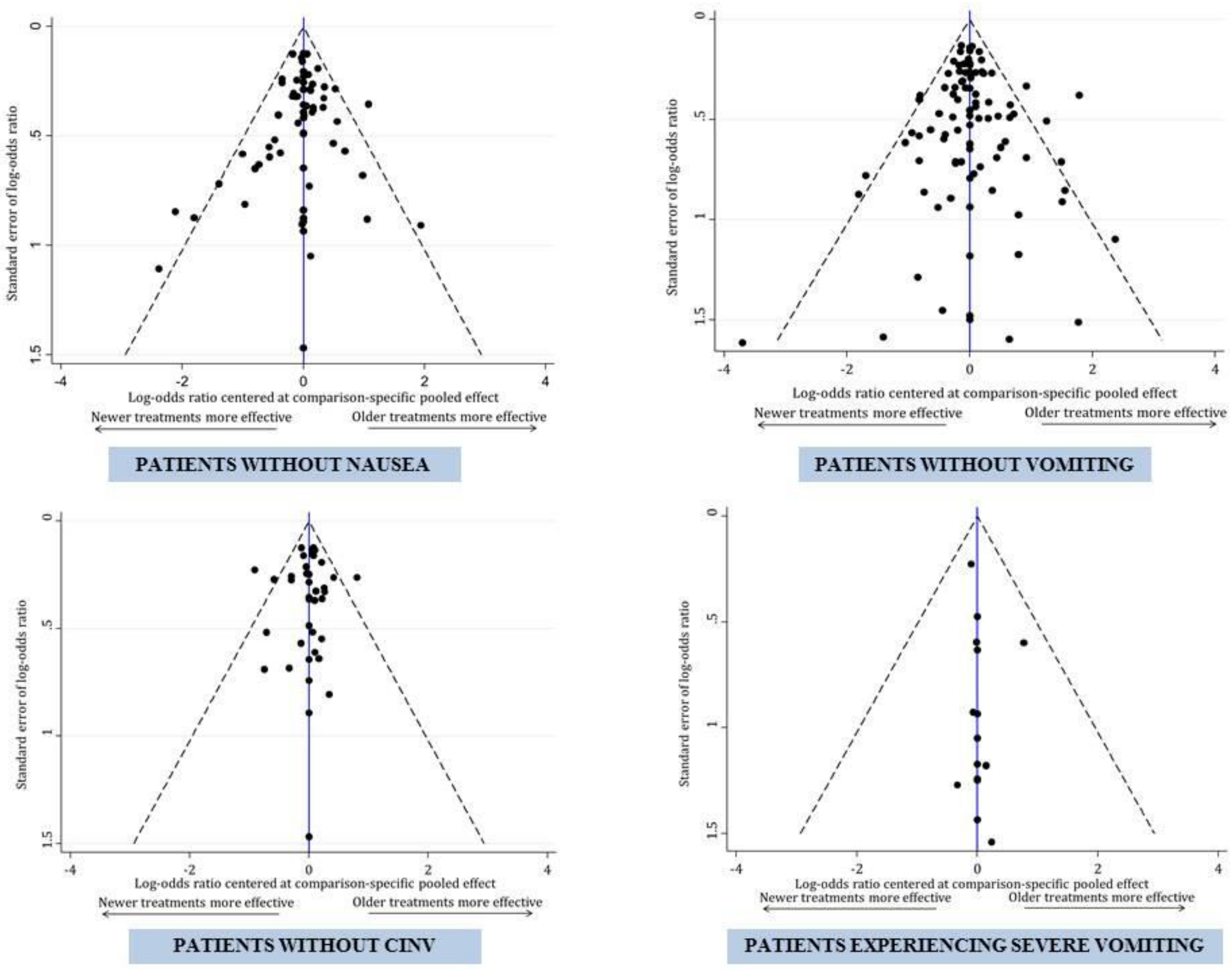

Abbreviations: CINV – chemotherapy-induced nausea and vomiting

Appendix H. Results for harms outcomes analyses

Treatment Comparison	# of head-to-head studies	OR (95% CI)*	OR (95% CI)†
**Arrhythmia: 3 studies and 627 patients were included in NMA**			
Granisetron vs Dolasetron	1	0.21 (0.02-1.84)	0.21 (0.02-1.84)
Granisetron+Dexamethasone vs Dolasetron	NA	0.66 (0.01-32.72)	NA
Palonosetron vs Dolasetron	NA	0.34 (0.03-3.63)	NA
Granisetron+Dexamethasone vs Granisetron	1	3.12 (0.12-80.39)	3.12 (0.12-80.39)
Palonosetron vs Granisetron	1	1.59 (0.58-4.30)	1.59 (0.58-4.30)
Palonosetron vs Granisetron+Dexamethasone	NA	0.51 (0.02-15.19)	NA
Between-study variance estimated in NMA model		NA	NA
Design-by-treatment interaction model for inconsistency χ^2 (d.f., P-value, Between-study variance)		NA (star-network)	
**Mortality: 8 studies and 4,823 adults were included in NMA and pairwise MA**			
Granisetron+Dexamethasone vs Granisetron	1	0.71 (0.18-2.83)	1.02 (0.20-5.15)
Metoclopramide+Dexamethasone vs Granisetron	1	1.11 (0.26-4.76)	1.32 (0.29-6.04)
Ondansetron vs Granisetron	1	0.63 (0.18-2.19)	0.49 (0.12-1.96)
Ondansetron+Dexamethasone vs Granisetron	NA	0.47 (0.10-2.19)	NA
Palonosetron+Dexamethasone vs Granisetron	NA	0.23 (0.04-1.42)	NA
Tropisetron vs Granisetron	NA	1.86 (0.06-58.30)	NA
Metoclopramide+Dexamethasone vs Granisetron+Dexamethasone	1	1.55 (0.36-6.66)	1.30 (0.28-5.93)
Ondansetron vs Granisetron+Dexamethasone	NA	0.88 (0.19-4.12)	NA
Ondansetron+Dexamethasone vs Granisetron+Dexamethasone	1	0.66 (0.17-2.53)	1.07 (0.18-6.44)
Palonosetron+Dexamethasone vs Granisetron+Dexamethasone	3	0.32 (0.09-1.14)	0.33 (0.09-1.18)
Tropisetron vs Granisetron+Dexamethasone	NA	2.61 (0.07-91.76)	NA
Ondansetron vs Metoclopramide+Dexamethasone	NA	0.57 (0.10-3.35)	NA
Ondansetron+Dexamethasone vs Metoclopramide+Dexamethasone	NA	0.43 (0.07-2.59)	NA
Palonosetron+Dexamethasone vs Metoclopramide+Dexamethasone	NA	0.20 (0.03-1.39)	NA
Tropisetron vs Metoclopramide+Dexamethasone	NA	1.68 (0.04-65.79)	NA
Ondansetron+Dexamethasone vs Ondansetron	2	0.75 (0.19-2.88)	0.53 (0.11-2.58)
Palonosetron+Dexamethasone vs	NA	0.36 (0.05-2.47)	NA

Ondansetron			
Tropisetron vs Ondansetron	1	2.95 (0.12-73.13)	2.95 (0.12-73.13)
Palonosetron+Dexamethasone vs Ondansetron+Dexamethasone	1	0.48 (0.08-2.74)	0.56 (0.03-10.83)
Tropisetron vs Ondansetron+Dexamethasone	NA	3.95 (0.12-128.78)	NA
Tropisetron vs Palonosetron+Dexamethasone	NA	8.25 (0.19-350.11)	NA
Between-study variance estimated in NMA model		0.00	0.00
Design-by-treatment interaction model for inconsistency χ^2 (d.f., P-value, Between-study variance)		0.8 (2, 0.67, 0.00)	
**QTc prolongation: 4 studies and 3358 patients were included in NMA**			
Granisetron + Dexamethasone vs Dolasetron ± Dexamethasone	NA	0.08 (0.00-1.82)	NA
Ondansetron ± Dexamethasone IV vs Dolasetron ± Dexamethasone	1	0.34 (0.24-0.47)	0.34 (0.24-0.47)
Palonosetron + Dexamethasone vs Dolasetron ± Dexamethasone	NA	0.07 (0.00-1.41)	NA
Ondansetron ± Dexamethasone IV vs Granisetron + Dexamethasone	NA	4.28 (0.19-97.30)	NA
Palonosetron + Dexamethasone vs Granisetron + Dexamethasone	1	0.84 (0.42-1.68)	0.84 (0.42-1.68)
Palonosetron + Dexamethasone vs Ondansetron ± Dexamethasone IV	1	0.20 (0.01-4.10)	0.20 (0.01-4.10)
Between-study variance estimated in NMA model		0.00	NA
Design-by-treatment interaction model for inconsistency χ^2 (d.f., P-value, Between-study variance)		NA	
**Sudden Cardiac Death: 1 study and 696 patients; NMA and pairwise MA were not feasible**			
Ondansetron vs Ondansetron +Dexamethasone	1	NA	2.73 (0.11-67.80)
Between-study variance estimated in NMA model		NA	NA
**QRS interval: 1 study and 696 patients; NMA and pairwise MA were not feasible**			
Ondansetron _d1-7 ± Dexamethasone _d1-7 vs Dolasetron± Dexamethasone	1	NA	0.29 (0.19-0.48)
Between-study variance estimated in NMA model		NA	NA
**Delirium: 1 study and 357 patients; NMA and pairwise MA were not feasible**			
Metoclopramide+Dexamethasone vs Granisetron	1	NA	4.14 (0.86-19.93)
Metoclopramide+Dexamethasone vs Granisetron+Dexamethasone	1	NA	4.07 (0.85-19.59)
Granisetron vs Granisetron+Dexamethasone	1	NA	0.98 (0.01-7.10)
Between-study variance estimated in NMA model		NA	NA

Abbreviations: CI - confidence interval; MA - meta-analysis; NA - not applicable; NMA - network meta-analysis; OR - odds ratio; RCT - randomized controlled trial
*Network Meta-analysis results
†Meta-analysis results

Appendix I. Aggregate Mcharms appraisal results

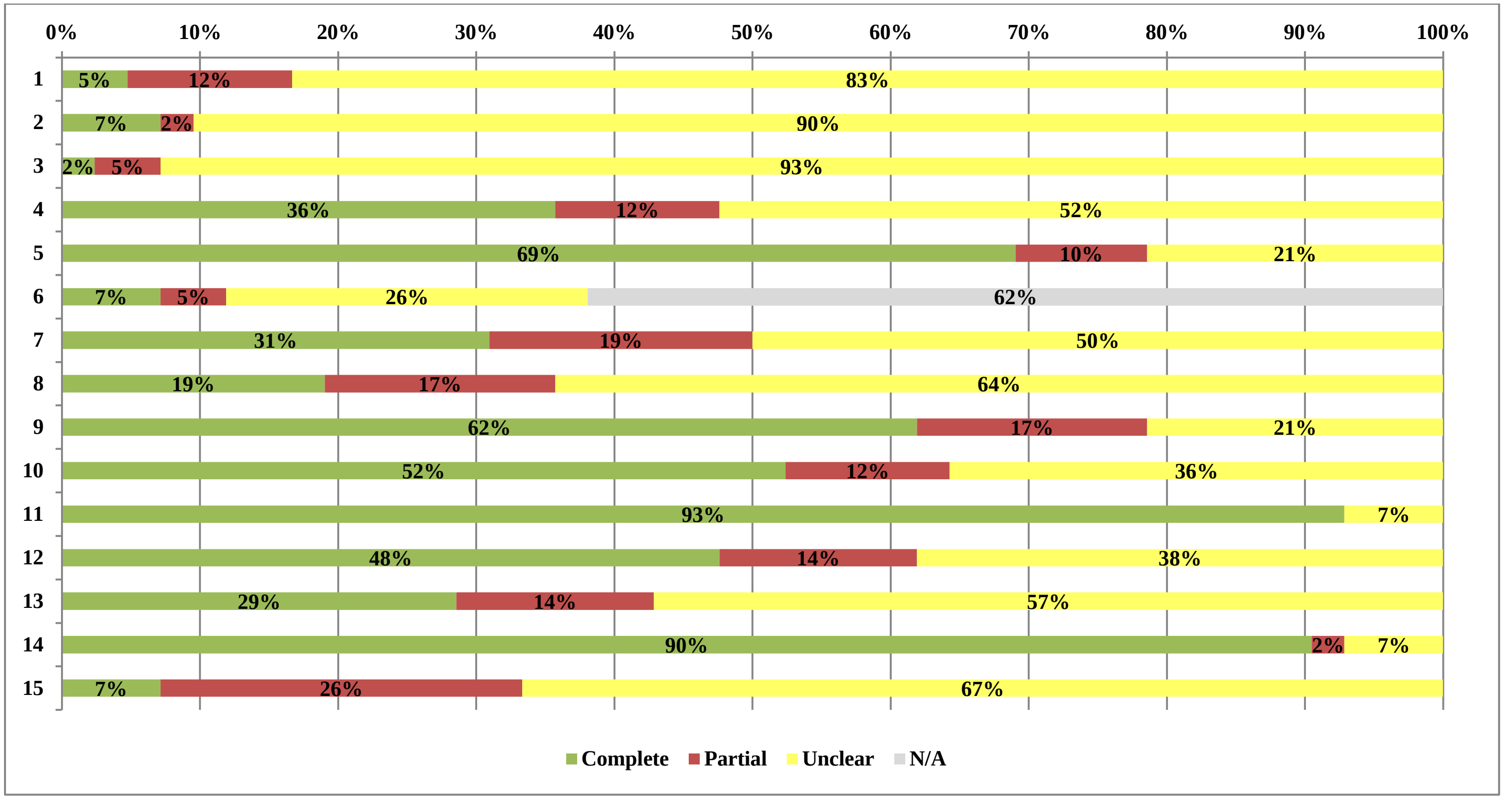

Abbreviations: N/A – not applicable

Items:

1. Were the harms PREDEFINED using standard or precise definitions?
2. Were SERIOUS events precisely defined?
3. Were SEVERE events precisely defined?
4. Were the number of DEATHS in each study group specified or were the reason(s) for not specifying given?
5. Was the mode of harms collection specified as ACTIVE?
6. Was the mode of harms collection specified as PASSIVE?
7. Did the study specify WHO collected the harms?

8. Did the study specify the TRAINING or BACKGROUND of who ascertained the harms?
9. Did the study specify the TIMING and FREQUENCY of collection of the harms?
10. Did the authors use STANDARD scales or checklists for harms collection?
11. Did the authors specify if the harms reported encompass ALL the events collected or a selected SAMPLE?
12. Was the overall number of participants that withdrew or were lost to follow-up, including each reason why, specified for each study group?
13. Was the NUMBER of participants who withdrew due to harms specified for each study group?
14. Did the author(s) specify the NUMBER and type of harmful events for each study group?
15. Did the author(s) specify the type of analyses undertaken for harms data?

Appendix J. Results for Mcharms appraisal

Author, year	1	2	3	4	5	6	7	8	9	10	11	12	13	14	15
Randomized clinical trials (n=39)															
Abang, 2000	U	U	U	C	P	U	U	U	C	U	C	C	U	C	U
Addelman, 1990	U	U	U	P	C	NA	C	C	C	C	C	P	P	P	U
Audhuy, 1996	U	U	U	U	C	NA	P	U	C	C	C	U	U	C	P
Boccia, 2011	U	U	U	C	C	NA	C	P	U	C	C	C	C	C	U
Chevallier, 1993	P	C	U	C	C	NA	C	P	C	C	C	U	U	C	P
De Mulder, 1990	U	U	U	P	P	P	P	U	C	U	C	P	P	C	P
Dong, 2011	U	U	U	U	U	U	U	U	U	P	U	U	U	C	U
du Bois, 1997	U	U	U	C	U	U	U	U	U	U	C	C	C	C	U
Eisenberg, 2003	U	U	U	C	P	U	U	U	C	U	C	C	C	C	U
Fauser, 1996a	U	U	U	C	C	NA	U	U	C	C	C	U	U	C	P
Ghosh, 2010	U	U	U	C	C	NA	U	U	U	C	C	U	U	C	U
Grote, 1997	U	U	U	U	C	NA	C	C	C	C	C	C	C	U	P
Heron, 1994	U	U	U	U	U	P	U	U	C	U	C	C	U	C	P
Hesketh, 1996b	U	U	U	P	C	NA	U	U	P	C	C	C	U	C	U
Hunter, 1993	U	U	U	C	U	U	U	U	U	U	C	C	P	C	P
Jones, 1991	U	U	U	P	C	C	C	C	P	U	C	P	P	C	U
Kaasa, 1990	U	U	U	U	C	U	U	U	U	U	C	U	U	C	U
Kasimis, 1997	U	U	U	U	C	NA	U	U	C	C	C	U	U	C	U
Kim, 2015	U	U	U	U	C	NA	P	P	C	C	C	P	U	C	U
Kovacs, 2016	C	C	C	C	NA	C	P	C	C	C	C	C	C	C	P
Lemerle, 1991	U	U	U	U	C	NA	U	U	C	C	C	U	U	C	U
Lofters, 1997	U	U	U	C	C	NA	U	U	C	C	C	C	U	C	U
Marschner, 1991	U	U	U	U	U	C	U	U	U	U	U	C	C	C	U
Matsuoka, 2003	U	U	U	U	C	NA	C	C	C	C	C	C	C	C	U
Mattiuzzi, 2010	U	U	U	U	U	U	U	U	U	U	C	C	P	C	U
Park, 1997	U	U	U	U	U	U	U	U	P	U	C	U	U	C	U
Perez, 1998b	U	U	U	U	C	NA	C	P	C	P	C	C	C	C	U
Perez, 1997	U	U	U	U	C	NA	U	U	P	U	C	U	U	C	U
Pinarli, 2006	C	U	U	U	C	NA	C	C	C	C	C	U	U	C	C
Raftopolous, 2015	P	P	C	C	NA	P	U	P	P	C	C	C	C	C	P
Rubenstein, 1997	U	U	U	U	C	NA	U	U	C	C	C	U	U	C	U
Saito, 2009	U	U	U	C	C	NA	P	U	C	C	C	C	U	C	U
Sanmukhani, 2014	P	U	P	U	C	NA	C	P	P	P	P	C	C	C	P
Smith (Granisetron Study Group), 1993	U	C	U	C	C	NA	C	P	C	C	C	C	C	C	C
Tian, 2011	U	U	U	U	C	NA	P	U	C	C	C	C	C	C	C
Tsavaris, 1995b	P	U	U	U	C	U	U	U	C	U	C	U	U	C	P
Warr, 1991	U	U	U	U	U	U	U	U	P	U	C	U	U	C	U
Wenzell, 2013	U	U	U	U	U	U	U	U	U	U	U	P	U	U	U
Van Belle, 1994b	U	U	U	P	C	NA	P	C	C	C	C	P	P	C	U
Non Randomized Clinical trials (n=3)															
Carmichael, 2003	U	U	U	U	P	C	P	U	C	P	C	U	U	C	U
Coppes, 1999b	P	U	U	C	C	NA	C	C	C	C	C	U	U	U	U
Coppes, 1999a	U	U	U	C	C	NA	C	C	C	C	C	C	U	C	U

Abbreviations: C - complete; NA - not applicable; P - partial; U - unclear

Items:

1. Were the harms PREDEFINED using standard or precise definitions?
2. Were SERIOUS events precisely defined?
3. Were SEVERE events precisely defined?
4. Were the number of DEATHS in each study group specified or were the reason(s) for not specifying given?
5. Was the mode of harms collection specified as ACTIVE?
6. Was the mode of harms collection specified as PASSIVE?
7. Did the study specify WHO collected the harms?
8. Did the study specify the TRAINING or BACKGROUND of who ascertained the harms?
9. Did the study specify the TIMING and FREQUENCY of collection of the harms?
10. Did the authors use STANDARD scales or checklists for harms collection?
11. Did the authors specify if the harms reported encompass ALL the events collected or a selected SAMPLE?
12. Was the overall number of participants that withdrew or were lost to follow-up, including each reason why, specified for each study

	group?
13.	Was the NUMBER of participants who withdrew due to harms specified for each study group?
14.	Did the author(s) specify the NUMBER and type of harmful events for each study group?
15.	Did the author(s) specify the type of analyses undertaken for harms data?

Appendix K. Results of the primary Network meta-analysis for effectiveness outcomes

Treatment Comparison	# of head to head studies	NMA estimate: OR (95% CI)	MA estimate: OR (95% CI)*
**No. of patients without nausea - 44 RCTs and 11,664 adults**			
Ondansetron+Steroid vs Granisetron+Steroid	5	1.03 (0.90-1.18)	1.04 (0.90-1.20)[a]
Ondansetron+Steroid vs Ondansetron	8	1.96 (1.59-2.41)	2.16 (1.45-3.23)[b]
Ondansetron+Steroid vs Granisetron	1	2.00 (1.63-2.45)	21.00 (3.35-131.51)
Ondansetron+Steroid vs Dolasetron	NA	2.09 (1.47-2.97)	NA
Ondansetron+Steroid vs Dolasetron+Steroid	NA	0.44 (0.20-0.96)	NA
Ondansetron+Steroid vs Palonosetron	NA	1.60 (1.06-2.41)	NA
Ondansetron+Steroid vs Palonosetron+Steroid	1	1.05 (0.80-1.36)	0.56 (0.20-1.59)
Ondansetron+Steroid vs Tropisetron	1	2.40 (1.49-3.88)	7.20 (2.03-25.51)
Ondansetron+Steroid vs Tropisetron+Steroid	NA	0.57 (0.26-1.24)	NA
Ondansetron+Steroid vs Ramosetron	NA	1.40 (0.84-2.33)	NA
Ondansetron+Steroid vs Ramosetron+Steroid	NA	0.69 (0.38-1.26)	NA
Ondansetron+Steroid vs Placebo	NA	337.68 (18.89-6,035.72)	NA
Granisetron+Steroid vs Ondansetron	1	1.91 (1.55-2.34)	3.39 (0.66-17.56)
Granisetron+Steroid vs Granisetron	6	1.95 (1.62-2.34)	1.96 (1.54-2.50)[c]
Granisetron+Steroid vs Dolasetron	NA	2.04 (1.44-2.87)	NA
Granisetron+Steroid vs Dolasetron+Steroid	NA	0.43 (0.20-0.93)	NA
Granisetron+Steroid vs Palonosetron	NA	1.55 (1.04-2.33)	NA
Granisetron+Steroid vs Palonosetron+Steroid	1	1.02 (0.81-1.28)	1.05 (0.83-1.33)
Granisetron+Steroid vs Tropisetron	1	2.34 (1.45-3.77)	7.20 (1.29-40.05)
Granisetron+Steroid vs Tropisetron+Steroid	NA	0.55 (0.25-1.21)	NA
Granisetron+Steroid vs Ramosetron	NA	1.36 (0.82-2.24)	NA
Granisetron+Steroid vs Ramosetron+Steroid	1	0.67 (0.37-1.21)	0.73 (0.28-1.90)
Granisetron+Steroid vs Placebo	NA	328.52 (18.40-5,864.44)	NA
Ondansetron vs Granisetron	11	1.02 (0.88-1.18)	1.03 (0.87-1.22)[d]
Ondansetron vs Dolasetron	2	1.07 (0.79-1.45)	1.12 (0.69-1.82)[e]
Ondansetron vs Dolasetron+Steroid	NA	0.22 (0.10-0.48)	NA
Ondansetron vs Palonosetron	1	0.82 (0.56-1.19)	1.03 (0.33-3.22)
Ondansetron vs Palonosetron+Steroid	NA	0.53 (0.39-0.73)	NA
Ondansetron vs Tropisetron	3	1.23 (0.79-1.91)	1.76 (0.65-4.76)[f]
Ondansetron vs Tropisetron+Steroid	NA	0.29 (0.13-0.62)	NA
Ondansetron vs Ramosetron	NA	0.71 (0.43-1.18)	NA
Ondansetron vs Ramosetron+Steroid	NA	0.35 (0.19-0.64)	NA
Ondansetron vs Placebo	NA	172.34 (9.67-3,070.30)	NA
Granisetron vs Dolasetron	1	1.05 (0.78-1.41)	0.97 (0.62-1.53)
Granisetron vs Dolasetron+Steroid	NA	0.22 (0.10-0.47)	NA
Granisetron vs Palonosetron	1	0.80 (0.55-1.15)	0.79 (0.45-1.40)
Granisetron vs Palonosetron+Steroid	NA	0.52 (0.39-0.70)	NA
Granisetron vs Tropisetron	2	1.20 (0.76-1.90)	0.93 (0.15-5.60)[g]
Granisetron vs Tropisetron+Steroid	NA	0.28 (0.13-0.61)	NA
Granisetron vs Ramosetron	2	0.70 (0.43-1.13)	0.79 (0.31-2.01)[h]
Granisetron vs Ramosetron+Steroid	NA	0.34 (0.19-0.62)	NA
Granisetron vs Placebo	1	168.85 (9.51-2,996.71)	169.00 (9.52-2,999.80)
Dolasetron vs Dolasetron+Steroid	1	0.21 (0.10-0.42)	0.21 (0.10-0.42)
Dolasetron vs Palonosetron	1	0.76 (0.54-1.07)	0.74 (0.50-1.12)
Dolasetron vs Palonosetron+Steroid	NA	0.50 (0.33-0.76)	NA
Dolasetron vs Tropisetron	NA	1.15 (0.67-1.96)	NA

Dolasetron vs Tropisetron+Steroid	NA	0.27 (0.12-0.61)	NA
Dolasetron vs Ramosetron	NA	0.67 (0.38-1.17)	NA
Dolasetron vs Ramosetron+Steroid	NA	0.33 (0.17-0.63)	NA
Dolasetron vs Placebo	NA	161.31 (8.95-2,907.94)	NA
Dolasetron+Steroid vs Palonosetron	NA	3.65 (1.68-7.94)	NA
Dolasetron+Steroid vs Palonosetron+Steroid	NA	2.39 (1.06-5.39)	NA
Dolasetron+Steroid vs Tropisetron	NA	5.49 (2.27-13.27)	NA
Dolasetron+Steroid vs Tropisetron+Steroid	NA	1.29 (0.44-3.81)	NA
Dolasetron+Steroid vs Ramosetron	NA	3.19 (1.29-7.85)	NA
Dolasetron+Steroid vs Ramosetron+Steroid	NA	1.57 (0.60-4.10)	NA
Dolasetron+Steroid vs Placebo	NA	771.35 (39.35-15,120.97)	NA
Palonosetron vs Palonosetron+Steroid	NA	0.65 (0.41-1.04)	NA
Palonosetron vs Tropisetron	NA	1.51 (0.84-2.68)	NA
Palonosetron vs Tropisetron+Steroid	NA	0.35 (0.15-0.83)	NA
Palonosetron vs Ramosetron	NA	0.87 (0.48-1.60)	NA
Palonosetron vs Ramosetron+Steroid	NA	0.43 (0.22-0.86)	NA
Palonosetron vs Placebo	NA	211.46 (11.64-3,841.31)	NA
Palonosetron+Steroid vs Tropisetron	NA	2.30 (1.35-3.91)	NA
Palonosetron+Steroid vs Tropisetron+Steroid	NA	0.54 (0.24-1.23)	NA
Palonosetron+Steroid vs Ramosetron	NA	1.33 (0.77-2.32)	NA
Palonosetron+Steroid vs Ramosetron+Steroid	NA	0.66 (0.35-1.24)	NA
Palonosetron+Steroid vs Placebo	NA	323.08 (17.93-5,821.31)	NA
Tropisetron vs Tropisetron+Steroid	2	0.24 (0.13-0.44)	0.24 (0.13-0.44)[i]
Tropisetron vs Ramosetron	NA	0.58 (0.30-1.13)	NA
Tropisetron vs Ramosetron+Steroid	NA	0.29 (0.14-0.60)	NA
Tropisetron vs Placebo	NA	140.45 (7.63-2,585.40)	NA
Tropisetron+Steroid vs Ramosetron	NA	2.47 (0.99-6.14)	NA
Tropisetron+Steroid vs Ramosetron+Steroid	NA	1.22 (0.46-3.20)	NA
Tropisetron+Steroid vs Placebo	NA	597.18 (30.37-11,744.01)	NA
Ramosetron vs Ramosetron+Steroid	1	0.49 (0.31-0.78)	0.48 (0.29-0.79)
Ramosetron vs Placebo	NA	242.02 (13.10-4,471.01)	NA
Ramosetron+Steroid vs Placebo	NA	490.92 (26.08-9,241.08)	NA
Between-study variance estimated in NMA model	0.00		
Design-by-treatment interaction model for inconsistency (χ2, d.f., P-value, Between-study variance)	(15.91, 12, 0.1955, 0.00)		
**No. of patients without vomiting: 71 RCTs and 16,300 adults**			
Ondansetron+Steroid vs Granisetron+Steroid	6	1.05 (0.78-1.41)	1.11 (0.94-1.30)[j]
Ondansetron+Steroid vs Ondansetron	10	2.62 (1.93-3.56)	2.65 (1.66-4.23[k]
Ondansetron+Steroid vs Granisetron	1	2.35 (1.71-3.23)	21.00 (3.35-131.51)
Ondansetron+Steroid vs Dolasetron	NA	2.90 (1.82-4.60)	NA
Ondansetron+Steroid vs Dolasetron+Steroid	1	1.13 (0.61-2.12)	1.49 (1.10-2.03)
Ondansetron+Steroid vs Palonosetron	NA	1.39 (0.87-2.23)	NA
Ondansetron+Steroid vs Palonosetron+Steroid	3	0.77 (0.49-1.21)	0.69 (0.51-0.94)[l]
Ondansetron+Steroid vs Tropisetron	1	3.35 (2.05-5.47)	7.20 (2.03-25.51)
Ondansetron+Steroid vs Tropisetron+Steroid	NA	0.90 (0.33-2.44)	NA
Ondansetron+Steroid vs Ramosetron	NA	1.89 (1.02-3.52)	NA
Ondansetron+Steroid vs Ramosetron+Steroid	NA	0.94 (0.41-2.15)	NA
Ondansetron+Steroid vs Placebo	NA	36.93 (14.95-91.23)	NA
Granisetron+Steroid vs Ondansetron	1	2.50 (1.80-3.46)	16.66 (0.92-302.02)
Granisetron+Steroid vs Granisetron	9	2.25 (1.68-3.00)	2.49 (1.32-4.69)[m]
Granisetron+Steroid vs Dolasetron	NA	2.77 (1.72-4.44)	NA

Granisetron+Steroid vs Dolasetron+Steroid	NA	1.08 (0.55-2.12)	NA
Granisetron+Steroid vs Palonosetron	NA	1.33 (0.83-2.13)	NA
Granisetron+Steroid vs Palonosetron+Steroid	1	0.74 (0.46-1.18)	0.94 (0.71-1.24)
Granisetron+Steroid vs Tropisetron	1	3.20 (1.95-5.24)	34.52 (1.83-650.54)
Granisetron+Steroid vs Tropisetron+Steroid	NA	0.86 (0.31-2.34)	NA
Granisetron+Steroid vs Ramosetron	NA	1.81 (0.98-3.32)	NA
Granisetron+Steroid vs Ramosetron+Steroid	1	0.90 (0.40-2.01)	0.79 (0.28-2.21)
Granisetron+Steroid vs Placebo	NA	35.27 (14.31-86.95)	NA
Ondansetron vs Granisetron	17	0.90 (0.72-1.12)	0.91 (0.70-1.17)[n]
Ondansetron vs Dolasetron	3	1.11 (0.76-1.62)	1.19 (0.79-1.79)[o]
Ondansetron vs Dolasetron+Steroid	NA	0.43 (0.22-0.85)	NA
Ondansetron vs Palonosetron	3	0.53 (0.36-0.79)	0.40 (0.25-0.63)[p]
Ondansetron vs Palonosetron+Steroid	NA	0.29 (0.17-0.50)	NA
Ondansetron vs Tropisetron	6	1.28 (0.84-1.94)	1.02 (0.62-1.69)[q]
Ondansetron vs Tropisetron+Steroid	NA	0.34 (0.13-0.90)	NA
Ondansetron vs Ramosetron	1	0.72 (0.41-1.27)	0.89 (0.45-1.74)
Ondansetron vs Ramosetron+Steroid	NA	0.36 (0.16-0.80)	NA
Ondansetron vs Placebo	3	14.12 (5.97-33.40)	11.02 (3.30-36.84)[r]
Granisetron vs Dolasetron	1	1.23 (0.82-1.84)	0.79 (0.50-1.23)
Granisetron vs Dolasetron+Steroid	NA	0.48 (0.25-0.94)	NA
Granisetron vs Palonosetron	3	0.59 (0.40-0.87)	0.69 (0.48-0.98)[s]
Granisetron vs Palonosetron+Steroid	NA	0.33 (0.20-0.55)	NA
Granisetron vs Tropisetron	6	1.42 (0.93-2.18)	1.55 (0.97-2.49)[t]
Granisetron vs Tropisetron+Steroid	NA	0.38 (0.14-1.01)	NA
Granisetron vs Ramosetron	2	0.80 (0.46-1.41)	0.61 (0.35-1.07)[u]
Granisetron vs Ramosetron+Steroid	NA	0.40 (0.18-0.89)	NA
Granisetron vs Placebo	2	15.70 (6.61-37.28)	33.60 (8.33-135.52)[v]
Dolasetron vs Dolasetron+Steroid	1	0.39 (0.19-0.79)	0.21 (0.09-0.50)
Dolasetron vs Palonosetron	1	0.48 (0.30-0.77)	0.55 (0.36-0.85)
Dolasetron vs Palonosetron+Steroid	NA	0.27 (0.14-0.50)	NA
Dolasetron vs Tropisetron	NA	1.16 (0.66-2.01)	NA
Dolasetron vs Tropisetron+Steroid	NA	0.31 (0.11-0.87)	NA
Dolasetron vs Ramosetron	NA	0.65 (0.33-1.27)	NA
Dolasetron vs Ramosetron+Steroid	NA	0.32 (0.13-0.78)	NA
Dolasetron vs Placebo	NA	12.75 (5.01-32.43)	NA
Dolasetron+Steroid vs Palonosetron	NA	1.23 (0.58-2.58)	NA
Dolasetron+Steroid vs Palonosetron+Steroid	NA	0.68 (0.32-1.46)	NA
Dolasetron+Steroid vs Tropisetron	NA	2.95 (1.37-6.37)	NA
Dolasetron+Steroid vs Tropisetron+Steroid	NA	0.79 (0.25-2.53)	NA
Dolasetron+Steroid vs Ramosetron	NA	1.67 (0.71-3.94)	NA
Dolasetron+Steroid vs Ramosetron+Steroid	NA	0.83 (0.30-2.30)	NA
Dolasetron+Steroid vs Placebo	NA	32.55 (11.02-96.12)	NA
Palonosetron vs Palonosetron+Steroid	NA	0.55 (0.30-1.04)	NA
Palonosetron vs Tropisetron	NA	2.40 (1.38-4.18)	NA
Palonosetron vs Tropisetron+Steroid	NA	0.65 (0.23-1.81)	NA
Palonosetron vs Ramosetron	NA	1.36 (0.70-2.66)	NA
Palonosetron vs Ramosetron+Steroid	NA	0.67 (0.28-1.63)	NA
Palonosetron vs Placebo	NA	26.53 (10.41-67.63)	NA
Palonosetron+Steroid vs Tropisetron	NA	4.34 (2.28-8.26)	NA
Palonosetron+Steroid vs Tropisetron+Steroid	NA	1.16 (0.39-3.44)	NA
Palonosetron+Steroid vs Ramosetron	NA	2.45 (1.16-5.16)	NA
Palonosetron+Steroid vs Ramosetron+Steroid	NA	1.22 (0.48-3.05)	NA
Palonosetron+Steroid vs Placebo	NA	47.86 (17.68-129.55)	NA

Tropisetron vs Tropisetron+Steroid	2	0.27 (0.11-0.64)	0.27 (0.13-0.57)[w]
Tropisetron vs Ramosetron	NA	0.57 (0.28-1.12)	NA
Tropisetron vs Ramosetron+Steroid	NA	0.28 (0.11-0.69)	NA
Tropisetron vs Placebo	NA	11.03 (4.28-28.47)	NA
Tropisetron+Steroid vs Ramosetron	NA	2.11 (0.69-6.40)	NA
Tropisetron+Steroid vs Ramosetron+Steroid	NA	1.04 (0.30-3.64)	NA
Tropisetron+Steroid vs Placebo	NA	41.11 (11.34-149.11)	NA
Ramosetron vs Ramosetron+Steroid	1	0.50 (0.24-1.02)	0.53 (0.31-0.89)
Ramosetron vs Placebo	1	19.52 (7.62-50.03)	10.45 (2.21-49.38)
Ramosetron+Steroid vs Placebo	NA	39.39 (12.80-121.23)	NA
Between-study variance estimated in NMA model		0.11	
Design-by-treatment interaction model for inconsistency (χ^2, d.f., P-value, Between-study variance)		(23.93, 17, 0.1214, 0.10)	
**No. of patients without CINV: 27 RCTs and 10.924 adults**			
Ondansetron+Steroid vs Granisetron+Steroid	6	0.97 (0.86-1.09)	1.01 (0.88-1.14)[x]
Ondansetron+Steroid vs Ondansetron	1	2.16 (1.62-2.87)	1.96 (1.12-3.42)
Ondansetron+Steroid vs Granisetron	NA	2.17 (1.73-2.72)	NA
Ondansetron+Steroid vs Palonosetron	NA	2.21 (0.50-9.72)	NA
Ondansetron+Steroid vs Palonosetron+Steroid	2	0.81 (0.67-0.98)	0.63 (0.35-1.12)[y]
Ondansetron+Steroid vs Tropisetron	NA	4.18 (1.99-8.77)	NA
Ondansetron+Steroid vs Ramosetron	NA	1.44 (0.82-2.55)	NA
Ondansetron+Steroid vs Ramosetron+Steroid	1	0.67 (0.34-1.31)	0.20 (0.06-0.72)
Ondansetron+Steroid vs Placebo	NA	365.96 (20.44-6,553.02)	NA
Granisetron+Steroid vs Ondansetron	NA	2.23 (1.69-2.93)	NA
Granisetron+Steroid vs Granisetron	6	2.24 (1.83-2.74)	2.35 (1.88-2.94)[z]
Granisetron+Steroid vs Palonosetron	NA	2.28 (0.52-10.02)	NA
Granisetron+Steroid vs Palonosetron+Steroid	3	0.84 (0.70-0.99)	0.78 (0.54-1.12)[a2]
Granisetron+Steroid vs Tropisetron	NA	4.31 (2.07-9.01)	NA
Granisetron+Steroid vs Ramosetron	NA	1.49 (0.85-2.62)	NA
Granisetron+Steroid vs Ramosetron+Steroid	NA	0.69 (0.35-1.35)	NA
Granisetron+Steroid vs Placebo	NA	378.03 (21.15-6,756.45)	NA
Ondansetron vs Granisetron	6	1.00 (0.81-1.25)	0.95 (0.75-1.20)[b2]
Ondansetron vs Palonosetron	1	1.02 (0.24-4.38)	1.03 (0.24-4.38)
Ondansetron vs Palonosetron+Steroid	NA	0.38 (0.27-0.52)	NA
Ondansetron vs Tropisetron	2	1.94 (0.95-3.95)	2.15 (0.93-4.96)[c2]
Ondansetron vs Ramosetron	1	0.67 (0.40-1.12)	0.84 (0.48-1.47)
Ondansetron vs Ramosetron+Steroid	NA	0.31 (0.16-0.59)	NA
Ondansetron vs Placebo	NA	169.61 (9.48-3,035.34)	NA
Granisetron vs Palonosetron	NA	1.02 (0.23-4.43)	NA
Granisetron vs Palonosetron+Steroid	NA	0.37 (0.29-0.49)	NA
Granisetron vs Tropisetron	2	1.93 (0.94-3.93)	1.77 (0.77-4.08)[d2]
Granisetron vs Ramosetron	NA	0.67 (0.39-1.15)	NA
Granisetron vs Ramosetron+Steroid	NA	0.31 (0.16-0.60)	NA
Granisetron vs Placebo	1	168.85 (9.51-2,996.64)	169.00 (9.52-2,999.80)
Palonosetron vs Palonosetron+Steroid	NA	0.37 (0.08-1.62)	NA
Palonosetron vs Tropisetron	NA	1.89 (0.37-9.52)	NA
Palonosetron vs Ramosetron	NA	0.65 (0.14-3.05)	NA
Palonosetron vs Ramosetron+Steroid	NA	0.30 (0.06-1.48)	NA
Palonosetron vs Placebo	NA	165.47 (6.55-4,182.08)	NA
Palonosetron+Steroid vs Tropisetron	NA	5.16 (2.43-10.97)	NA

Palonosetron+Steroid vs Ramosetron	NA	1.79 (0.99-3.21)	NA
Palonosetron+Steroid vs Ramosetron+Steroid	NA	0.83 (0.42-1.64)	NA
Palonosetron+Steroid vs Placebo	NA	452.19 (25.18-8,121.41)	NA
Tropisetron vs Ramosetron	NA	0.35 (0.14-0.83)	NA
Tropisetron vs Ramosetron+Steroid	NA	0.16 (0.06-0.42)	NA
Tropisetron vs Placebo	NA	87.64 (4.53-1,696.76)	NA
Ramosetron vs Ramosetron+Steroid	1	0.46 (0.29-0.73)	0.55 (0.34-0.89)
Ramosetron vs Placebo	NA	253.30 (13.55-4,734.53)	NA
Ramosetron+Steroid vs Placebo	NA	546.71 (28.58-10,456.90)	NA
Between-study variance estimated in NMA model		0.00	
Design-by-treatment interaction model for inconsistency (χ^2, d.f., P-value, Between-study variance)		(11.95, 6, 0.0631, 0.00)	
**No. of patients experiencing severe vomiting: 11 RCTs and 1,364 adults**			
Ondansetron+Steroid vs Ondansetron	1	0.58 (0.07-4.56)	0.58 (0.07-4.56)
Ondansetron+Steroid vs Granisetron	NA	0.38 (0.05-3.06)	NA
Ondansetron+Steroid vs Ramosetron	NA	0.52 (0.06-4.68)	NA
Ondansetron+Steroid vs Placebo	NA	0.09 (0.01-1.17)	NA
Ondansetron vs Granisetron	4	0.65 (0.45-0.94)	0.63 (0.43-0.93)[e2]
Ondansetron vs Ramosetron	1	0.89 (0.41-1.94)	NA
Ondansetron vs Placebo	3	0.16 (0.04-0.70)	0.16 (0.04-0.70)[f2]
Granisetron vs Ramosetron	1	1.37 (0.64-2.92)	1.17 (0.46-2.96)
Granisetron vs Placebo	NA	0.25 (0.05-1.13)	NA
Ramosetron vs Placebo	NA	0.18 (0.03-0.95)	NA
Ramosetron+Steroid vs Granisetron+Steroid	1	NA	0.97 (0.06-16.16)
Between-study variance estimated in NMA model		NA	
Design-by-treatment interaction model for inconsistency (χ^2, d.f., P-value, Between-study variance)		(0.33, 1, 0.5628, 0.00)	

Abbreviations: CI - confidence interval; CINV - chemotherapy-induced nausea and vomiting; MA - meta-analysis; NA - not applicable; NMA - network meta-analysis; OR - odds ratio; RCT - randomized controlled trial

*** Between-study variance values have been indicated by a letter:** a= 0.00, b= 0.11, c= 0.01, d < 0.0001, e= 0.00, f= 0.51, g= 0.22, h= 1.01, i = 0.00**,** j =0.00, k= 0.28, l= 0.00, m= 0.67, n= 0.12, o= 0.04, p= 0.00, q= 0.15, r=0.00, s=0.00, t=<0.0001, u= 0.00, v= 0.00, w= 0.00, x= 0.00, y= 0.13, z= 0.01, a2 = 0.07, b2, = 0.00, c2= 0.00, d2= 0.00, e2= 0.00, f2= 0.00.

.

Appendix L. No. of patients without nausea – RCTs and adults only

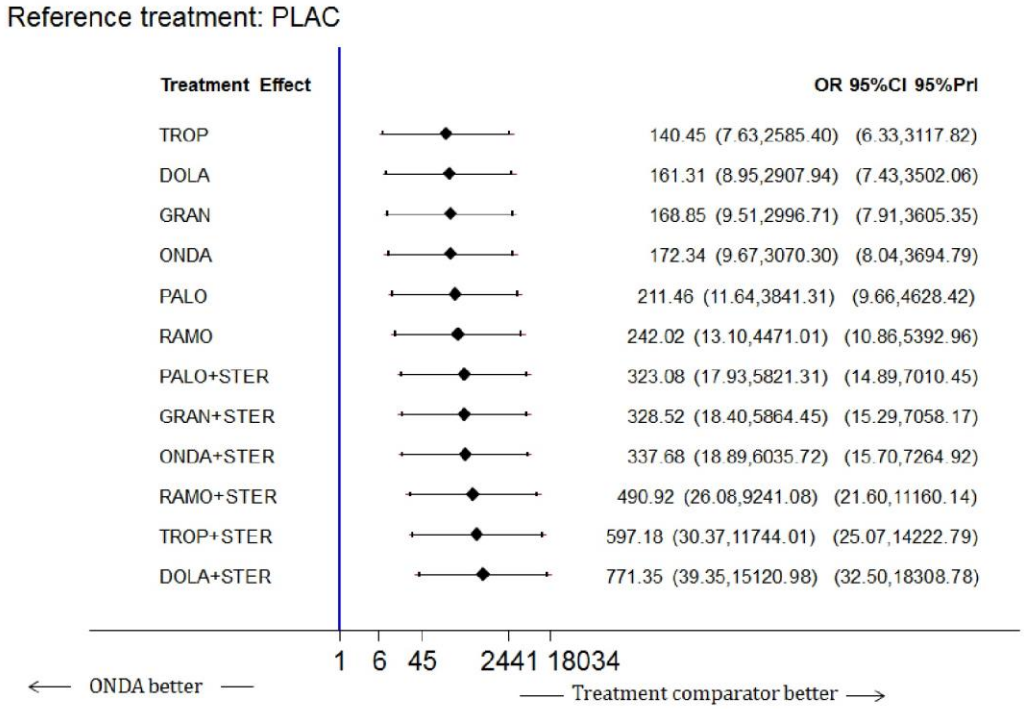

Abbreviations: CI - confidence interval; OR - odds ratio; PrI - prediction interval; RCT - randomized controlled trial

DOLA - dolasetron; GRAN - granisetron; ONDA - ondansetron; PLAC - placebo; PALO - palonosetron; RAMO - ramosetron; STER - steroid; TROP - tropisetron

Appendix M. No. of patients without nausea – RCTs and adults only – SUCRA values for each treatment

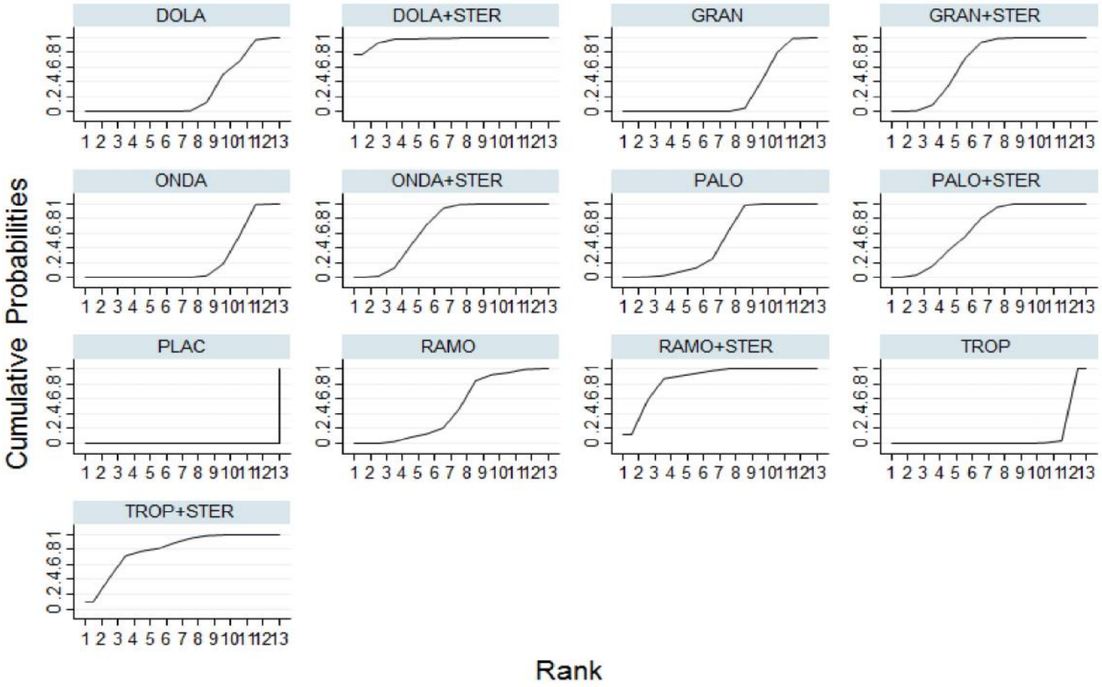

Abbreviation: RCT - randomized controlled trial; SUCRA - surface under the cumulative ranking curve

DOLA - dolasetron; DOLA+STER - dolasetron+steroid; GRAN - granisetron; GRAN+STER - granisetron+steroid; ONDA - ondansetron; ONDA+STER - ondansetron+steroid; PLAC - placebo; PALO - palonosetron; PALO+STER- palonosetron+steroid; RAMO - ramosetron; RAMO+STER - ramosetron+steroid; TROP - tropisetron; TROP+STER - tropisetron+steroid

Appendix N. No. of patients without nausea sub-group analysis for Network meta-analysis

NO. OF PATIENTS WITHOUT NAUSEA	ALL STUDY DESIGNS	ALL RCTs	LOW ROB RANDOMIZATION	CDDP YES (RCTs SGA)	CDDP NO (RCTs SGA)
**Treatment Comparison**	**OR (95% CI)**	**OR (95% CI)**	**OR (95% CI)**	**OR (95% CI)**	**OR (95% CI)**
Ondansetron+Steroid vs Granisetron+Steroid	0.92 (0.74-1.14)	1.01 (0.87-1.17)	1.00 (0.79-1.27)	1.10 (0.93-1.31)	0.92 (0.72-1.18)
Ondansetron+Steroid vs Ondansetron	2.07 (1.59-2.70)	2.02 (1.62-2.52)	1.99 (1.42-2.80)	1.94 (1.50-2.51)	2.62 (1.39-4.94)
Ondansetron+Steroid vs Granisetron	1.93 (1.49-2.51)	1.94 (1.56-2.40)	2.33 (1.63-3.32)	1.92 (1.50-2.46)	2.53 (1.45-4.42)
Ondansetron+Steroid vs Dolasetron	2.14 (1.39-3.32)	1.92 (1.34-2.77)	2.48 (1.14-5.42)	1.87 (1.19-2.94)	3.24 (1.33-7.89)
Ondansetron+Steroid vs Dolasetron+Steroid	0.71 (0.30-1.66)	0.40 (0.18-0.90)	NA	0.39 (0.17-0.90)	2.00 (0.91-4.44)
Ondansetron+Steroid vs Palonosetron	1.32 (0.82-2.12)	1.29 (0.86-1.92)	1.84 (0.95-3.60)	1.99 (0.62-6.43)	0.92 (0.55-1.54)
Ondansetron+Steroid vs Palonosetron+Steroid	0.94 (0.63-1.40)	1.02 (0.77-1.36)	1.05 (0.78-1.43)	NA	NA
Ondansetron+Steroid vs Tropisetron	3.16 (2.02-4.96)	3.19 (2.02-5.06)	1.71 (0.90-3.23)	NA	NA
Ondansetron+Steroid vs Tropisetron+Steroid	0.79 (0.44-1.40)	0.75 (0.34-1.64)	NA	NA	NA
Ondansetron+Steroid vs Ramosetron	1.66 (0.95-2.92)	1.36 (0.80-2.32)	1.39 (0.71-2.73)	1.25 (0.72-2.16)	8.51 (2.11-34.27)
Ondansetron+Steroid vs Ramosetron+Steroid	0.76 (0.38-1.52)	0.67 (0.36-1.25)	0.67 (0.29-1.55)	0.64 (0.34-1.19)	2.36 (0.49-11.46)
Ondansetron+Steroid vs Placebo	326.60 (17.69-6,030.97)	327.24 (18.22-5,877.76)	NA	323.73 (18.05-5,807.37)	4.49 (0.83-24.37)
Granisetron+Steroid vs Ondansetron	2.25 (1.70-2.99)	2.00 (1.60-2.51)	1.99 (1.39-2.85)	1.76 (1.36-2.28)	2.85 (1.59-5.11)
Granisetron+Steroid vs Granisetron	2.10 (1.65-2.68)	1.92 (1.58-2.34)	2.33 (1.66-3.26)	1.74 (1.40-2.17)	2.75 (1.66-4.54)
Granisetron+Steroid vs Dolasetron	2.33 (1.50-3.63)	1.91 (1.33-2.73)	2.48 (1.15-5.37)	1.70 (1.09-2.64)	3.52 (1.49-8.28)
Granisetron+Steroid vs Dolasetron+Steroid	0.77 (0.33-1.82)	0.40 (0.18-0.89)	NA	0.36 (0.15-0.81)	2.18 (1.02-4.64)
Granisetron+Steroid vs Palonosetron	1.43 (0.90-2.30)	1.28 (0.86-1.89)	1.84 (0.96-3.56)	1.81 (0.56-5.83)	1.00 (0.56-1.77)
Granisetron+Steroid vs Palonosetron+Steroid	1.02 (0.70-1.49)	1.02 (0.79-1.31)	1.05 (0.84-1.32)	NA	NA
Granisetron+Steroid vs Tropisetron	3.44 (2.17-5.46)	3.17 (1.99-5.04)	1.71 (0.90-3.26)	NA	NA
Granisetron+Steroid vs Tropisetron+Steroid	0.85 (0.48-1.53)	0.74 (0.34-1.63)	NA	NA	NA
Granisetron+Steroid vs Ramosetron	1.81 (1.03-3.17)	1.35 (0.80-2.28)	1.39 (0.72-2.70)	1.13 (0.67-1.93)	9.24 (2.34-36.42)
Granisetron+Steroid vs Ramosetron+Steroid	0.83 (0.42-1.63)	0.67 (0.36-1.23)	0.67 (0.29-1.53)	0.58 (0.32-1.07)	2.56 (0.54-12.21)
Granisetron+Steroid vs Placebo	354.94 (19.25-6,544.13)	324.91 (18.11-5,828.26)	NA	293.72 (16.41-5,256.75)	4.88 (0.92-26.00)
Ondansetron vs Granisetron	0.93 (0.77-1.13)	0.96 (0.82-1.12)	1.17 (0.88-1.55)	0.99 (0.80-1.21)	0.96 (0.72-1.30)
Ondansetron vs Dolasetron	1.03 (0.71-1.50)	0.95 (0.70-1.30)	1.24 (0.59-2.64)	0.96 (0.63-1.46)	1.23 (0.66-2.31)
Ondansetron vs Dolasetron+Steroid	0.34 (0.15-0.78)	0.20 (0.09-0.44)	NA	0.20 (0.09-0.46)	0.76 (0.40-1.45)
Ondansetron vs Palonosetron	0.64 (0.42-0.97)	0.64 (0.44-0.92)	0.93 (0.49-1.74)	1.03 (0.33-3.22)	0.35 (0.15-0.79)
Ondansetron vs Palonosetron+Steroid	0.45 (0.29-0.71)	0.51 (0.36-0.72)	0.53 (0.35-0.80)	NA	NA
Ondansetron vs Tropisetron	1.53 (1.02-2.28)	1.58 (1.05-2.38)	0.86 (0.50-1.47)	NA	NA

Ondansetron vs Tropisetron+Steroid	0.38 (0.21-0.68)	0.37 (0.17-0.79)	NA	NA	NA
Ondansetron vs Ramosetron	0.80 (0.47-1.37)	0.68 (0.40-1.13)	0.70 (0.37-1.32)	0.64 (0.37-1.11)	3.24 (0.91-11.60)
Ondansetron vs Ramosetron+Steroid	0.37 (0.19-0.73)	0.33 (0.18-0.62)	0.34 (0.15-0.76)	0.33 (0.18-0.62)	0.90 (0.21-3.94)
Ondansetron vs Placebo	157.53 (8.58-2,893.49)	162.14 (9.06-2,901.65)	NA	166.69 (9.32-2,980.39)	1.71 (0.34-8.68)
Granisetron vs Dolasetron	1.11 (0.76-1.62)	0.99 (0.73-1.35)	1.07 (0.53-2.14)	0.98 (0.66-1.44)	1.28 (0.64-2.56)
Granisetron vs Dolasetron+Steroid	0.37 (0.16-0.84)	0.21 (0.09-0.45)	NA	0.20 (0.09-0.46)	0.79 (0.45-1.40)
Granisetron vs Palonosetron	0.68 (0.45-1.04)	0.66 (0.47-0.95)	0.79 (0.45-1.40)	1.04 (0.33-3.32)	0.36 (0.17-0.78)
Granisetron vs Palonosetron+Steroid	0.48 (0.31-0.75)	0.53 (0.38-0.73)	0.45 (0.30-0.67)	NA	NA
Granisetron vs Tropisetron	1.64 (1.08-2.48)	1.65 (1.08-2.52)	0.73 (0.40-1.35)	NA	NA
Granisetron vs Tropisetron+Steroid	0.41 (0.23-0.72)	0.39 (0.18-0.83)	NA	NA	NA
Granisetron vs Ramosetron	0.86 (0.51-1.44)	0.70 (0.43-1.16)	0.60 (0.34-1.06)	0.65 (0.39-1.08)	3.37 (0.94-12.06)
Granisetron vs Ramosetron+Steroid	0.39 (0.20-0.77)	0.35 (0.19-0.63)	0.29 (0.14-0.61)	0.33 (0.18-0.61)	0.93 (0.21-4.09)
Granisetron vs Placebo	168.87 (9.25-3,081.80)	168.85 (9.48-3,008.88)	NA	168.85 (9.51-2,996.74)	1.78 (0.36-8.76)
Dolasetron vs Dolasetron+Steroid	0.33 (0.16-0.70)	0.21 (0.10-0.43)	NA	0.21 (0.10-0.42)	0.62 (0.25-1.51)
Dolasetron vs Palonosetron	0.62 (0.40-0.95)	0.67 (0.47-0.95)	0.74 (0.50-1.12)	1.07 (0.32-3.59)	0.28 (0.10-0.79)
Dolasetron vs Palonosetron+Steroid	0.44 (0.25-0.78)	0.53 (0.34-0.83)	0.43 (0.19-0.95)	NA	NA
Dolasetron vs Tropisetron	1.47 (0.87-2.50)	1.66 (1.00-2.76)	0.69 (0.27-1.74)	NA	NA
Dolasetron vs Tropisetron+Steroid	0.37 (0.19-0.72)	0.39 (0.17-0.88)	NA	NA	NA
Dolasetron vs Ramosetron	0.78 (0.42-1.45)	0.71 (0.40-1.27)	0.56 (0.23-1.38)	0.67 (0.35-1.26)	2.63 (0.63-10.87)
Dolasetron vs Ramosetron+Steroid	0.36 (0.17-0.76)	0.35 (0.18-0.69)	0.27 (0.10-0.76)	0.34 (0.17-0.70)	0.73 (0.15-3.62)
Dolasetron vs Placebo	152.29 (8.14-2,847.65)	170.17 (9.39-3,083.15)	NA	173.01 (9.49-3,152.99)	1.39 (0.24-7.90)
Dolasetron+Steroid vs Palonosetron	1.85 (0.78-4.41)	3.20 (1.44-7.09)	NA	5.10 (1.25-20.73)	0.46 (0.18-1.18)
Dolasetron+Steroid vs Palonosetron+Steroid	1.32 (0.52-3.36)	2.55 (1.10-5.92)	NA	NA	NA
Dolasetron+Steroid vs Tropisetron	4.44 (1.83-10.82)	7.94 (3.30-19.13)	NA	NA	NA
Dolasetron+Steroid vs Tropisetron+Steroid	1.10 (0.41-2.97)	1.86 (0.63-5.50)	NA	NA	NA
Dolasetron+Steroid vs Ramosetron	2.34 (0.90-6.04)	3.39 (1.34-8.55)	NA	3.19 (1.24-8.24)	4.24 (1.05-17.14)
Dolasetron+Steroid vs Ramosetron+Steroid	1.07 (0.38-3.05)	1.67 (0.63-4.48)	NA	1.64 (0.60-4.45)	1.18 (0.24-5.73)
Dolasetron+Steroid vs Placebo	458.86 (22.42-9,389.80)	813.70 (41.14-16,092.52)	NA	827.30 (41.76-16,390.19)	2.24 (0.41-12.18)
Palonosetron vs Palonosetron+Steroid	0.71 (0.39-1.29)	0.80 (0.50-1.27)	0.57 (0.29-1.14)	NA	NA
Palonosetron vs Tropisetron	2.40 (1.35-4.27)	2.48 (1.44-4.30)	0.93 (0.40-2.13)	NA	NA
Palonosetron vs Tropisetron+Steroid	0.60 (0.29-1.20)	0.58 (0.25-1.34)	NA	NA	NA
Palonosetron vs Ramosetron	1.26 (0.65-2.45)	1.06 (0.58-1.95)	0.76 (0.34-1.69)	0.63 (0.18-2.21)	9.27 (2.10-41.04)
Palonosetron vs Ramosetron+Steroid	0.58 (0.26-1.27)	0.52 (0.26-1.05)	0.36 (0.14-0.93)	0.32 (0.09-1.18)	2.57 (0.49-13.58)
Palonosetron vs Placebo	247.54 (13.16-4,656.49)	254.55 (13.98-4,635.90)	NA	162.31 (7.30-3,607.54)	4.90 (0.84-28.72)
Palonosetron+Steroid vs Tropisetron	3.37 (1.87-6.08)	3.12 (1.82-5.35)	1.62 (0.82-3.19)	NA	NA
Palonosetron+Steroid vs Tropisetron+Steroid	0.84 (0.42-1.66)	0.73 (0.32-1.68)	NA	NA	NA
Palonosetron+Steroid vs Ramosetron	1.78 (0.91-3.48)	1.33 (0.74-2.39)	1.32 (0.66-2.65)	NA	NA
Palonosetron+Steroid vs Ramosetron+Steroid	0.81 (0.38-1.76)	0.66 (0.34-1.28)	0.64 (0.27-1.49)	NA	NA
Palonosetron+Steroid vs Placebo	348.38 (18.46-6,572.91)	319.48 (17.61-5,796.89)	NA	NA	NA

Tropisetron vs Tropisetron+Steroid	0.25 (0.14-0.43)	0.23 (0.12-0.44)	NA	NA	NA
Tropisetron vs Ramosetron	0.53 (0.29-0.95)	0.43 (0.22-0.81)	0.82 (0.35-1.88)	NA	NA
Tropisetron vs Ramosetron+Steroid	0.24 (0.12-0.50)	0.21 (0.10-0.44)	0.39 (0.15-1.04)	NA	NA
Tropisetron vs Placebo	103.25 (5.49-1,940.54)	102.44 (5.57-1,883.48)	NA	NA	NA
Tropisetron+Steroid vs Ramosetron	2.12 (1.01-4.43)	1.82 (0.74-4.49)	NA	NA	NA
Tropisetron+Steroid vs Ramosetron+Steroid	0.97 (0.42-2.27)	0.90 (0.34-2.36)	NA	NA	NA
Tropisetron+Steroid vs Placebo	415.69 (21.51-8,031.80)	436.73 (22.20-8,592.11)	NA	NA	NA
Ramosetron vs Ramosetron+Steroid	0.46 (0.26-0.81)	0.49 (0.31-0.79)	0.48 (0.29-0.79)	0.51 (0.32-0.81)	0.28 (0.13-0.59)
Ramosetron vs Placebo	196.25 (10.27-3,749.13)	240.06 (12.91-4,463.94)	NA	259.26 (13.98-4,809.16)	0.53 (0.07-4.07)
Ramosetron+Steroid vs Placebo	427.80 (21.74-8,416.98)	486.00 (25.62-9,217.62)	NA	505.93 (26.80-9,550.84)	1.91 (0.22-16.78)
Between-study variance in NMA	0.04	0.01	0.00	0.00	0.00
Design-by-treatment interaction model for inconsistency (χ^2, d.f., P-value, Between-study variance)	31.11 (17, 0.0194, 0.02)	23.17 (13, 0.0396, 0.00)	0.06 (2, 0.9709, 0.01)	0.39 (3, 0.9425, 0.00)	1.84 (1, 0.1748, 0.00)

Abbreviations: CDDP - cisplatin; CI - confidence interval; NA - not applicable; NMA – network meta-analysis; OR - odds ratio; RCT - randomized controlled trial; ROB – risk of bias; SGA – sub-group analysis

Appendix O. No. of patients without vomiting – RCTs only

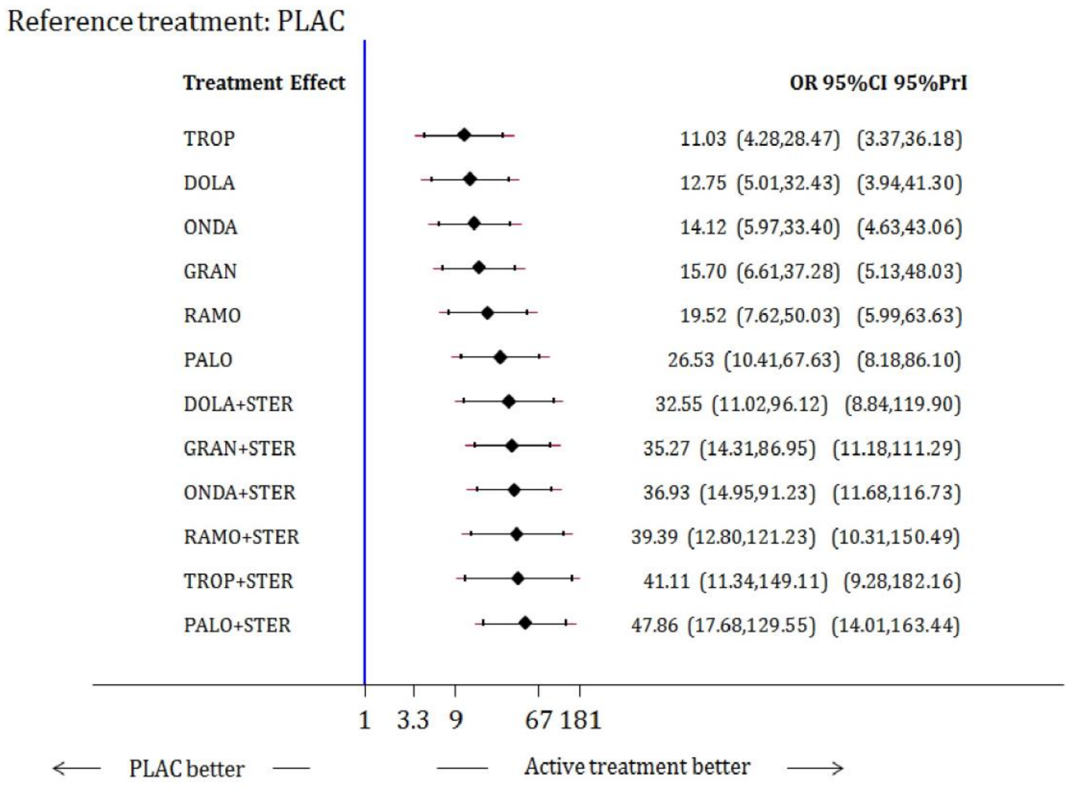

Abbreviations: CI - confidence interval; OR - odds ratio; PrI - prediction interval; RCT - randomized controlled trial

DOLA - dolasetron; GRAN - granisetron; ONDA - ondansetron; PALO - palonosetron; PLAC – placebo; RAMO - ramosetron; STER – steroid; TROP - tropisetron

Appendix P. No. of patients without vomiting – RCTs only – SUCRA values for each treatment

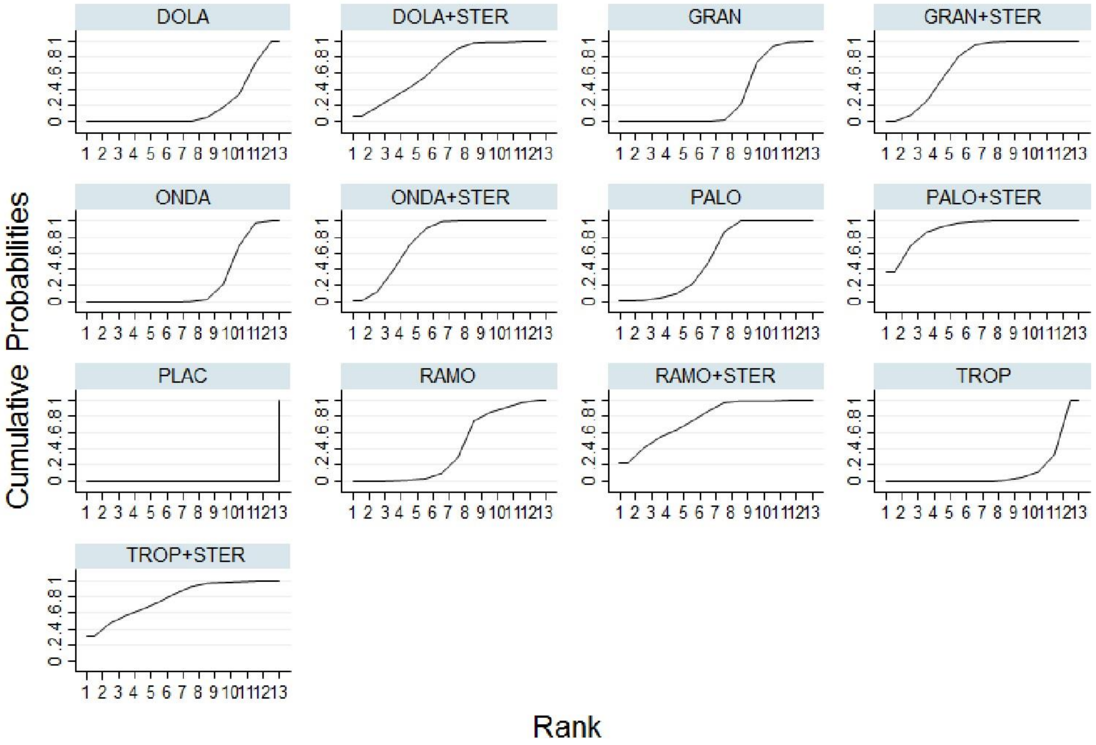

Abbreviation: RCT - randomized controlled trial; SUCRA - surface under the cumulative ranking curve

DOLA - dolasetron; DOLA+STER - dolasetron+steroid; GRAN - granisetron; GRAN+STER - granisetron+steroid; ONDA - ondansetron; ONDA+STER - ondansetron+steroid; PLAC - placebo; PALO - palonosetron; PALO+STER - palonosetron+steroid; RAMO - ramosetron; RAMO+STER - ramosetron+steroid; TROP - tropisetron; TROP+STER - tropisetron+steroid

Appendix Q. No. of patients without vomiting sub-group analysis for Network meta-analysis

NO. OF PATIENTS WITHOUT VOMITING	ALL STUDY DESIGNS	ALL RCTs	LOW ROB RANDOMIZATION	CDDP YES (RCTs SGA)	CDDP NO (RCTs SGA)
**Treatment Comparison**	**OR (95% CI)**	**OR (95% CI)**	**OR (95% CI)**	**OR (95% CI)**	**OR (95% CI)**
Ondansetron+Steroid vs Granisetron+Steroid	1.07 (0.81-1.40)	1.05 (0.78-1.41)	1.09 (0.51-2.31)	1.13 (0.74-1.73)	1.08 (0.43-2.70)
Ondansetron+Steroid vs Ondansetron	2.62 (1.94-3.52)	2.62 (1.93-3.56)	2.82 (1.46-5.45)	2.09 (1.38-3.18)	5.74 (1.33-24.85)
Ondansetron+Steroid vs Granisetron	2.37 (1.74-3.21)	2.35 (1.71-3.23)	2.17 (0.98-4.80)	1.98 (1.26-3.13)	5.07 (1.32-19.46)
Ondansetron+Steroid vs Dolasetron	2.94 (1.87-4.65)	2.90 (1.82-4.60)	2.68 (0.56-12.88)	1.97 (1.02-3.82)	9.76 (1.56-61.08)
Ondansetron+Steroid vs Dolasetron+Steroid	1.33 (0.74-2.41)	1.13 (0.61-2.12)	NA	0.41 (0.11-1.55)	NA
Ondansetron+Steroid vs Palonosetron	1.40 (0.88-2.23)	1.39 (0.87-2.23)	1.47 (0.54-4.01)	1.28 (0.57-2.85)	3.84 (0.71-20.79)
Ondansetron+Steroid vs Palonosetron+Steroid	0.78 (0.50-1.21)	0.77 (0.49-1.21)	0.80 (0.38-1.68)	0.71 (0.31-1.63)	0.61 (0.23-1.60)
Ondansetron+Steroid vs Tropisetron	3.27 (2.09-5.11)	3.35 (2.05-5.47)	4.47 (1.09-18.34)	1.96 (0.64-6.02)	5.43 (0.99-29.74)
Ondansetron+Steroid vs Tropisetron+Steroid	0.77 (0.38-1.55)	0.90 (0.33-2.44)	NA	0.36 (0.06-2.06)	1.99 (0.23-17.04)
Ondansetron+Steroid vs Ramosetron	1.89 (1.06-3.36)	1.89 (1.02-3.52)	1.30 (0.29-5.79)	1.58 (0.77-3.22)	4.80 (0.18-125.95)
Ondansetron+Steroid vs Ramosetron+Steroid	0.94 (0.42-2.11)	0.94 (0.41-2.15)	0.69 (0.10-4.79)	0.85 (0.34-2.13)	NA
Ondansetron+Steroid vs Placebo	36.96 (15.08-90.57)	36.93 (14.95-91.23)	16.37 (0.55-486.26)	23.94 (7.84-73.11)	258.22 (7.44-8,957.84)
Granisetron+Steroid vs Ondansetron	2.46 (1.80-3.36)	2.50 (1.80-3.46)	2.59 (1.17-5.74)	1.86 (1.18-2.91)	5.33 (1.70-16.72)
Granisetron+Steroid vs Granisetron	2.22 (1.68-2.94)	2.25 (1.68-3.00)	2.00 (1.01-3.94)	1.76 (1.20-2.57)	4.70 (1.76-12.60)
Granisetron+Steroid vs Dolasetron	2.76 (1.74-4.39)	2.77 (1.72-4.44)	2.46 (0.52-11.69)	1.75 (0.91-3.37)	9.06 (1.85-44.37)
Granisetron+Steroid vs Dolasetron+Steroid	1.25 (0.66-2.34)	1.08 (0.55-2.12)	NA	0.36 (0.10-1.37)	NA
Granisetron+Steroid vs Palonosetron	1.32 (0.83-2.08)	1.33 (0.83-2.13)	1.35 (0.51-3.61)	1.13 (0.52-2.45)	3.56 (0.86-14.72)
Granisetron+Steroid vs Palonosetron+Steroid	0.73 (0.46-1.16)	0.74 (0.46-1.18)	0.73 (0.31-1.71)	0.63 (0.24-1.60)	0.57 (0.15-2.14)
Granisetron+Steroid vs Tropisetron	3.07 (1.96-4.81)	3.20 (1.95-5.24)	4.11 (0.94-18.06)	1.74 (0.57-5.30)	5.04 (1.20-21.12)
Granisetron+Steroid vs Tropisetron+Steroid	0.72 (0.35-1.46)	0.86 (0.31-2.34)	NA	0.32 (0.05-1.82)	1.85 (0.27-12.88)
Granisetron+Steroid vs Ramosetron	1.78 (1.01-3.13)	1.81 (0.98-3.32)	1.20 (0.28-5.03)	1.40 (0.70-2.77)	4.45 (0.19-102.54)
Granisetron+Steroid vs Ramosetron+Steroid	0.89 (0.40-1.94)	0.90 (0.40-2.01)	0.63 (0.09-4.22)	0.75 (0.32-1.79)	NA
Granisetron+Steroid vs Placebo	34.69 (14.19-84.85)	35.27 (14.31-86.95)	15.04 (0.49-460.13)	21.22 (6.99-64.45)	239.69 (7.79-7,372.36)
Ondansetron vs Granisetron	0.90 (0.73-1.12)	0.90 (0.72-1.12)	0.77 (0.40-1.50)	0.95 (0.67-1.35)	0.88 (0.49-1.59)
Ondansetron vs Dolasetron	1.13 (0.77-1.64)	1.11 (0.76-1.62)	0.95 (0.22-4.18)	0.94 (0.55-1.62)	1.70 (0.57-5.12)
Ondansetron vs Dolasetron+Steroid	0.51 (0.27-0.95)	0.43 (0.22-0.85)	NA	0.20 (0.05-0.70)	NA
Ondansetron vs Palonosetron	0.54 (0.36-0.79)	0.53 (0.36-0.79)	0.52 (0.22-1.23)	0.61 (0.29-1.28)	0.67 (0.21-2.17)
Ondansetron vs Palonosetron+Steroid	0.30 (0.18-0.50)	0.29 (0.17-0.50)	0.28 (0.11-0.71)	0.34 (0.13-0.86)	0.11 (0.02-0.63)
Ondansetron vs Tropisetron	1.25 (0.85-1.83)	1.28 (0.84-1.94)	1.59 (0.46-5.53)	0.94 (0.32-2.71)	0.95 (0.34-2.67)
Ondansetron vs Tropisetron+Steroid	0.29 (0.15-0.59)	0.34 (0.13-0.90)	NA	0.17 (0.03-0.95)	0.35 (0.07-1.84)
Ondansetron vs Ramosetron	0.72 (0.43-1.22)	0.72 (0.41-1.27)	0.46 (0.11-1.93)	0.75 (0.40-1.41)	0.84 (0.04-17.40)
Ondansetron vs Ramosetron+Steroid	0.36 (0.16-0.79)	0.36 (0.16-0.80)	0.24 (0.04-1.62)	0.41 (0.17-0.98)	NA
Ondansetron vs Placebo	14.13 (6.00-33.26)	14.12 (5.97-33.40)	5.80 (0.21-161.61)	11.43 (4.01-32.64)	44.99 (1.78-1,136.95)
Granisetron vs Dolasetron	1.24 (0.84-1.85)	1.23 (0.82-1.84)	1.23 (0.29-5.19)	0.99 (0.56-1.76)	1.93 (0.55-6.72)
Granisetron vs Dolasetron+Steroid	0.56 (0.30-1.06)	0.48 (0.25-0.94)	NA	0.21 (0.06-0.75)	NA
Granisetron vs Palonosetron	0.59 (0.40-0.87)	0.59 (0.40-0.87)	0.68 (0.31-1.47)	0.64 (0.33-1.27)	0.76 (0.27-2.10)
Granisetron vs Palonosetron+Steroid	0.33 (0.20-0.54)	0.33 (0.20-0.55)	0.37 (0.14-0.96)	0.36 (0.14-0.92)	0.12 (0.02-0.63)
Granisetron vs Tropisetron	1.38 (0.93-2.04)	1.42 (0.93-2.18)	2.06 (0.50-8.48)	0.99 (0.34-2.86)	1.07 (0.38-3.04)

NO. OF PATIENTS WITHOUT VOMITING	ALL STUDY DESIGNS	ALL RCTs	LOW ROB RANDOMIZATION	CDDP YES (RCTs SGA)	CDDP NO (RCTs SGA)
**Treatment Comparison**	**OR (95% CI)**	**OR (95% CI)**	**OR (95% CI)**	**OR (95% CI)**	**OR (95% CI)**
Granisetron vs Tropisetron+Steroid	0.32 (0.16-0.65)	0.38 (0.14-1.01)	NA	0.18 (0.03-1.00)	0.39 (0.07-2.10)
Granisetron vs Ramosetron	0.80 (0.48-1.35)	0.80 (0.46-1.41)	0.60 (0.17-2.13)	0.79 (0.42-1.49)	0.95 (0.05-18.60)
Granisetron vs Ramosetron+Steroid	0.40 (0.18-0.87)	0.40 (0.18-0.89)	0.32 (0.05-1.87)	0.43 (0.18-1.02)	NA
Granisetron vs Placebo	15.62 (6.61-36.90)	15.70 (6.61-37.28)	7.54 (0.25-224.32)	12.06 (4.13-35.21)	50.96 (1.91-1,357.86)
Dolasetron vs Dolasetron+Steroid	0.45 (0.23-0.88)	0.39 (0.19-0.79)	NA	0.21 (0.07-0.66)	NA
Dolasetron vs Palonosetron	0.48 (0.30-0.76)	0.48 (0.30-0.77)	0.55 (0.16-1.84)	0.65 (0.27-1.56)	0.39 (0.08-1.97)
Dolasetron vs Palonosetron+Steroid	0.26 (0.14-0.49)	0.27 (0.14-0.50)	0.30 (0.06-1.59)	0.36 (0.12-1.04)	0.06 (0.01-0.51)
Dolasetron vs Tropisetron	1.11 (0.66-1.88)	1.16 (0.66-2.01)	1.67 (0.24-11.58)	1.00 (0.31-3.22)	0.56 (0.12-2.53)
Dolasetron vs Tropisetron+Steroid	0.26 (0.12-0.57)	0.31 (0.11-0.87)	NA	0.18 (0.03-1.08)	0.20 (0.03-1.51)
Dolasetron vs Ramosetron	0.64 (0.34-1.21)	0.65 (0.33-1.27)	0.49 (0.07-3.29)	0.80 (0.36-1.78)	0.49 (0.02-12.41)
Dolasetron vs Ramosetron+Steroid	0.32 (0.14-0.76)	0.32 (0.13-0.78)	0.26 (0.03-2.51)	0.43 (0.16-1.18)	NA
Dolasetron vs Placebo	12.55 (4.97-31.70)	12.75 (5.01-32.43)	6.11 (0.16-233.03)	12.15 (3.78-39.05)	26.45 (0.87-802.40)
Dolasetron+Steroid vs Palonosetron	1.05 (0.52-2.14)	1.23 (0.58-2.58)	NA	3.12 (0.73-13.31)	NA
Dolasetron+Steroid vs Palonosetron+Steroid	0.59 (0.28-1.22)	0.68 (0.32-1.46)	NA	1.73 (0.36-8.32)	NA
Dolasetron+Steroid vs Tropisetron	2.46 (1.20-5.02)	2.95 (1.37-6.37)	NA	4.80 (0.93-24.90)	NA
Dolasetron+Steroid vs Tropisetron+Steroid	0.58 (0.23-1.42)	0.79 (0.25-2.53)	NA	0.87 (0.10-7.33)	NA
Dolasetron+Steroid vs Ramosetron	1.42 (0.64-3.16)	1.67 (0.71-3.94)	NA	3.86 (0.95-15.72)	NA
Dolasetron+Steroid vs Ramosetron+Steroid	0.71 (0.27-1.89)	0.83 (0.30-2.30)	NA	2.08 (0.45-9.61)	NA
Dolasetron+Steroid vs Placebo	27.80 (9.70-79.61)	32.55 (11.02-96.12)	NA	58.60 (11.35-302.66)	NA
Palonosetron vs Palonosetron+Steroid	0.55 (0.30-1.03)	0.55 (0.30-1.04)	0.54 (0.17-1.72)	0.55 (0.17-1.77)	0.16 (0.02-1.11)
Palonosetron vs Tropisetron	2.33 (1.38-3.94)	2.40 (1.38-4.18)	3.04 (0.67-13.78)	1.54 (0.44-5.39)	1.42 (0.33-6.09)
Palonosetron vs Tropisetron+Steroid	0.55 (0.25-1.20)	0.65 (0.23-1.81)	NA	0.28 (0.04-1.76)	0.52 (0.07-3.69)
Palonosetron vs Ramosetron	1.35 (0.72-2.54)	1.36 (0.70-2.66)	0.88 (0.20-3.89)	1.23 (0.49-3.08)	1.25 (0.05-29.11)
Palonosetron vs Ramosetron+Steroid	0.67 (0.29-1.59)	0.67 (0.28-1.63)	0.46 (0.07-3.23)	0.67 (0.22-1.99)	NA
Palonosetron vs Placebo	26.36 (10.41-66.72)	26.53 (10.41-67.63)	11.10 (0.36-344.61)	18.76 (5.33-66.06)	67.28 (2.16-2,093.47)
Palonosetron+Steroid vs Tropisetron	4.20 (2.29-7.71)	4.34 (2.28-8.26)	5.60 (1.18-26.51)	2.78 (0.69-11.26)	8.88 (1.25-63.16)
Palonosetron+Steroid vs Tropisetron+Steroid	0.98 (0.44-2.22)	1.16 (0.39-3.44)	NA	0.51 (0.07-3.53)	3.26 (0.31-34.46)
Palonosetron+Steroid vs Ramosetron	2.43 (1.20-4.92)	2.45 (1.16-5.16)	1.63 (0.33-7.98)	2.23 (0.74-6.71)	7.84 (0.26-236.00)
Palonosetron+Steroid vs Ramosetron+Steroid	1.21 (0.50-2.97)	1.22 (0.48-3.05)	0.86 (0.11-6.47)	1.20 (0.35-4.16)	NA
Palonosetron+Steroid vs Placebo	47.49 (17.71-127.35)	47.86 (17.68-129.55)	20.50 (0.65-648.14)	33.91 (8.40-136.83)	422.04 (10.61-16,795.88)
Tropisetron vs Tropisetron+Steroid	0.23 (0.12-0.46)	0.27 (0.11-0.64)	NA	0.18 (0.05-0.70)	0.37 (0.10-1.36)
Tropisetron vs Ramosetron	0.58 (0.32-1.06)	0.57 (0.28-1.12)	0.29 (0.04-1.94)	0.80 (0.24-2.69)	0.88 (0.04-20.71)
Tropisetron vs Ramosetron+Steroid	0.29 (0.12-0.67)	0.28 (0.11-0.69)	0.15 (0.02-1.48)	0.43 (0.11-1.67)	NA
Tropisetron vs Placebo	11.32 (4.49-28.53)	11.03 (4.28-28.47)	3.66 (0.10-127.83)	12.20 (2.78-53.60)	47.53 (1.60-1,412.75)
Tropisetron+Steroid vs Ramosetron	2.47 (1.06-5.75)	2.11 (0.69-6.40)	NA	4.42 (0.72-27.03)	2.40 (0.08-73.18)
Tropisetron+Steroid vs Ramosetron+Steroid	1.23 (0.44-3.42)	1.04 (0.30-3.64)	NA	2.38 (0.35-16.07)	NA
Tropisetron+Steroid vs Placebo	48.23 (16.13-144.23)	41.11 (11.34-149.11)	NA	67.12 (9.06-497.09)	129.46 (3.41-4,908.53)
Ramosetron vs Ramosetron+Steroid	0.50 (0.25-1.01)	0.50 (0.24-1.02)	0.53 (0.15-1.83)	0.54 (0.25-1.17)	NA
Ramosetron vs Placebo	19.53 (7.75-49.21)	19.52 (7.62-50.03)	12.58 (0.34-470.51)	15.19 (5.08-45.43)	53.83 (0.64-4,528.72)
Ramosetron+Steroid vs Placebo	39.16 (12.88-119.03)	39.39 (12.80-121.23)	23.89 (0.52-1,100.61)	28.19 (7.80-101.89)	NA

**NO. OF PATIENTS WITHOUT VOMITING**	**ALL STUDY DESIGNS**	**ALL RCTs**	**LOW ROB RANDOMIZATION**	**CDDP YES (RCTs SGA)**	**CDDP NO (RCTs SGA)**
**Treatment Comparison**	**OR (95% CI)**	**OR (95% CI)**	**OR (95% CI)**	**OR (95% CI)**	**OR (95% CI)**
Between-study variance in NMA	0.11	0.11	0.11	0.14	0.20
Design-by-treatment interaction model for inconsistency (χ^2, d.f., P-value, Between-study variance)	28.21 (21, 0.1344, 0.10)	23.93 (17, 0.1214, 0.10)	0.96 (3, 0.8118, 0.50)	3.75 (8, 0.8791, 0.24)	0.94 (1, 0.3331, 0.17)

Abbreviations: CDDP - Cisplatin; CI - confidence interval; NA - not applicable; NMA – network meta-analysis; OR - odds ratio; RCT - randomized controlled trial; ROB – risk of bias; SGA – sub-group analysis

Appendix R. No. of patients without CINV – RCTs only

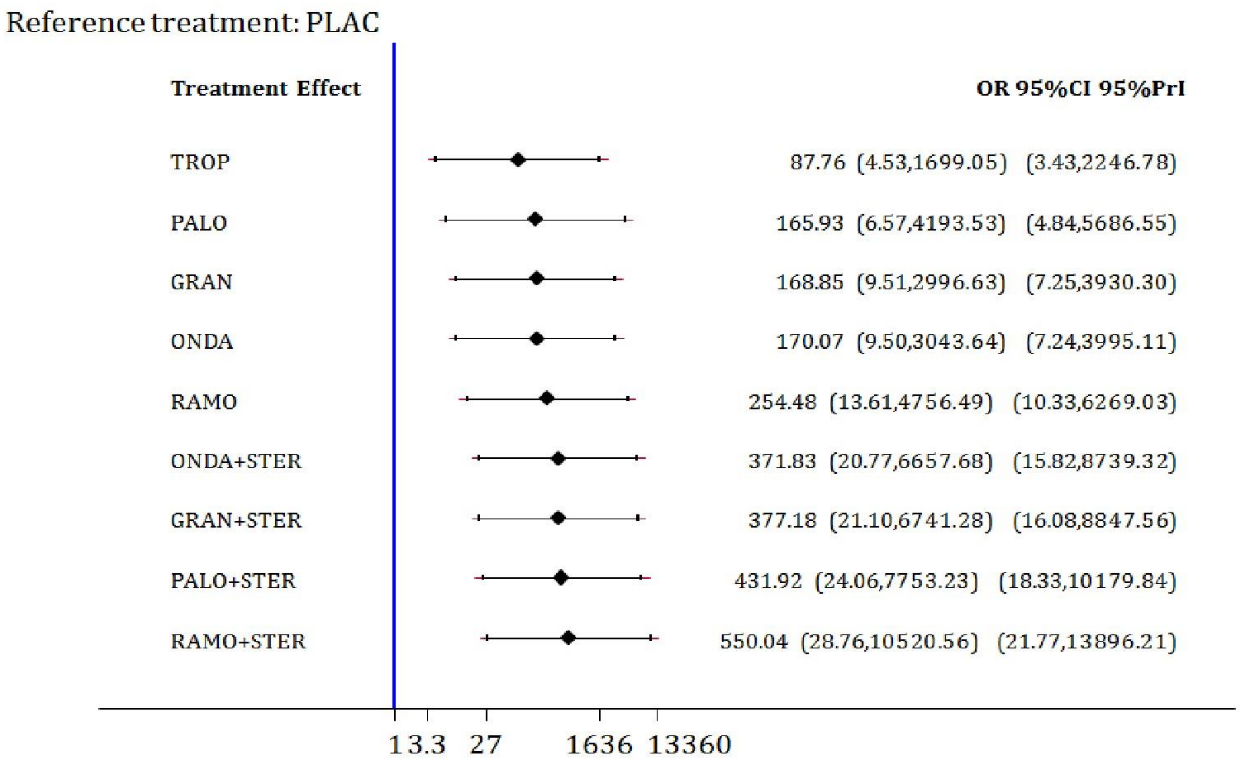

Abbreviations: CI - confidence interval; CINV – chemotherapy-induced nausea and vomiting; OR - odds ratio; PrI - prediction interval; RCT - randomized controlled trial

GRAN - granisetron; PALO - palonosteron; PLAC - placebo; ONDA - ondansetron; STER - steroid; TROP - tropisetron;

Appendix S. No. of patients without CINV – RCTs only – SUCRA values for each treatment

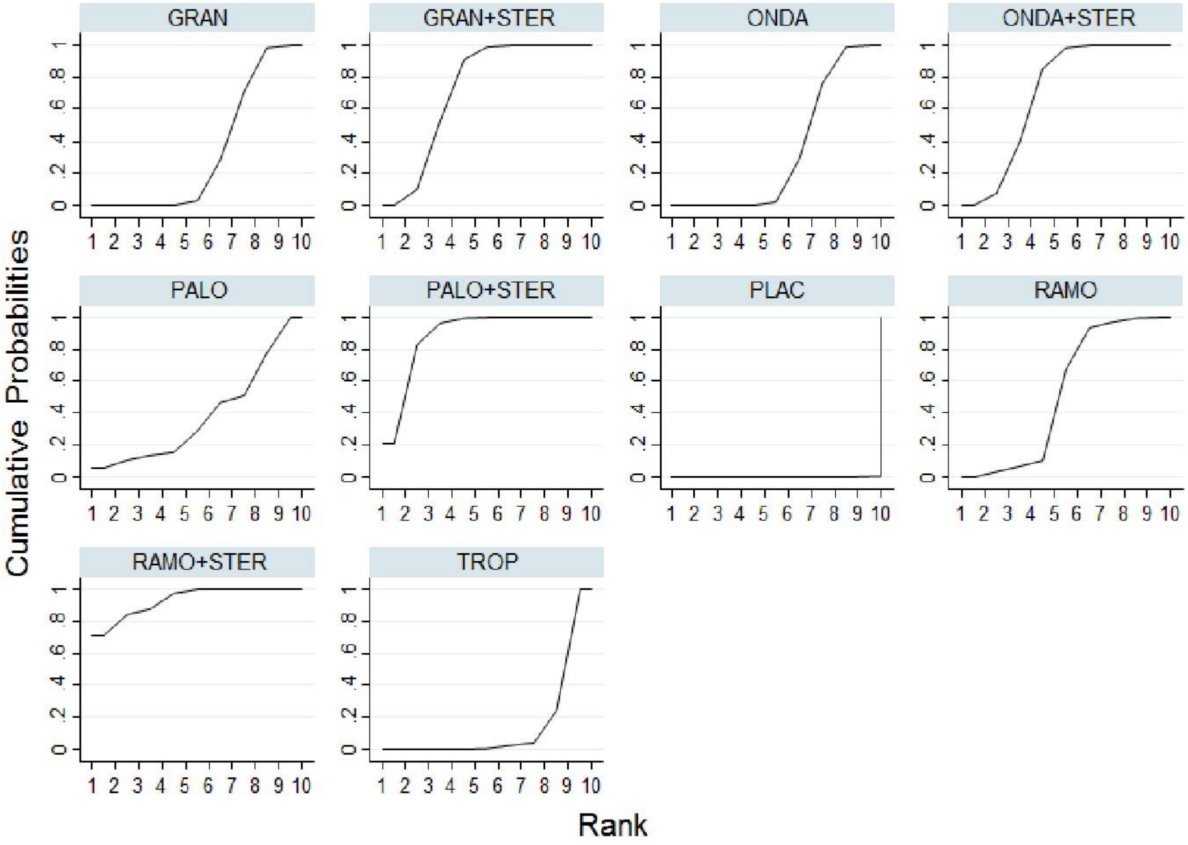

Abbreviations: CINV - chemotherapy-induced nausea and vomiting; RCT - randomized controlled trial; SUCRA – surface under the cumulative ranking curve

GRAN- granisetron; ONDA - ondansetron; PALO - palonosetron; PLAC - placebo; STER- steroid; TROP - tropisetron

Appendix T. No. of patients without CINV sub-group analysis for Network meta-analysis

NO. OF PATIENTS WITHOUT CINV	ALL STUDY DESIGNS	ALL RCTs	LOW ROB RANDOMIZATION	CDDP YES (RCTs SGA)	CDDP NO (RCTs SGA)
**Treatment Comparison**	**OR (95% CI)**	**OR (95% CI)**	**OR (95% CI)**	**OR (95% CI)**	**OR (95% CI)**
Ondansetron+Steroid vs Granisetron+Steroid	0.99 (0.89-1.11)	0.99 (0.88-1.11)	0.98 (0.79-1.22)	1.06 (0.89-1.27)	1.00 (0.76-1.31)
Ondansetron+Steroid vs Ondansetron	2.20 (1.66-2.93)	2.19 (1.64-2.91)	1.96 (1.12-3.42)	2.48 (1.63-3.77)	3.57 (1.55-8.20)
Ondansetron+Steroid vs Granisetron	2.19 (1.75-2.74)	2.20 (1.76-2.76)	2.47 (1.58-3.85)	2.42 (1.77-3.31)	3.14 (1.74-5.66)
Ondansetron+Steroid vs Dolasetron+Steroid	7.46 (1.30-42.94)	NA	NA	NA	NA
Ondansetron+Steroid vs Palonosetron	2.26 (0.51-9.93)	2.24 (0.51-9.85)	NA	2.54 (0.56-11.52)	NA
Ondansetron+Steroid vs Palonosetron+Steroid	0.87 (0.73-1.03)	0.86 (0.72-1.02)	0.93 (0.74-1.17)	0.82 (0.56-1.19)	1.00 (0.70-1.43)
Ondansetron+Steroid vs Tropisetron	2.67 (1.41-5.06)	4.24 (2.02-8.90)	NA	4.57 (1.72-12.16)	6.76 (1.87-24.37)
Ondansetron+Steroid vs Tropisetron+Steroid	0.92 (0.49-1.73)	NA	NA	NA	NA
Ondansetron+Steroid vs Ramosetron	1.82 (1.06-3.13)	1.46 (0.83-2.58)	NA	0.37 (0.10-1.42)	3.01 (1.09-8.27)
Ondansetron+Steroid vs Ramosetron+Steroid	0.99 (0.54-1.80)	0.68 (0.35-1.32)	NA	0.20 (0.06-0.72)	NA
Ondansetron+Steroid vs Placebo	370.51 (20.70-6,632.94)	371.83 (20.77-6,657.68)	NA	408.96 (22.66-7,382.46)	NA
Granisetron+Steroid vs Ondansetron	2.22 (1.68-2.92)	2.22 (1.69-2.92)	1.99 (1.10-3.63)	2.33 (1.60-3.41)	3.57 (1.60-7.94)
Granisetron+Steroid vs Granisetron	2.21 (1.80-2.70)	2.23 (1.83-2.73)	2.51 (1.71-3.70)	2.28 (1.76-2.95)	3.14 (1.86-5.30)
Granisetron+Steroid vs Dolasetron+Steroid	7.50 (1.31-43.02)	NA	NA	NA	NA
Granisetron+Steroid vs Palonosetron	2.27 (0.52-9.96)	2.27 (0.52-9.97)	NA	2.39 (0.53-10.74)	NA
Granisetron+Steroid vs Palonosetron+Steroid	0.87 (0.74-1.02)	0.87 (0.74-1.03)	0.95 (0.76-1.18)	0.77 (0.51-1.17)	1.00 (0.74-1.35)
Granisetron+Steroid vs Tropisetron	2.68 (1.42-5.07)	4.30 (2.06-8.97)	NA	4.30 (1.64-11.27)	6.76 (1.92-23.75)
Granisetron+Steroid vs Tropisetron+Steroid	0.93 (0.49-1.74)	NA	NA	NA	NA
Granisetron+Steroid vs Ramosetron	1.83 (1.07-3.14)	1.48 (0.84-2.61)	NA	0.35 (0.09-1.36)	3.01 (1.12-8.05)
Granisetron+Steroid vs Ramosetron+Steroid	1.00 (0.55-1.81)	0.69 (0.35-1.34)	NA	0.19 (0.05-0.68)	NA
Granisetron+Steroid vs Placebo	372.57 (20.85-6,658.80)	377.18 (21.10-6,741.29)	NA	385.22 (21.46-6,916.04)	NA
Ondansetron vs Granisetron	1.00 (0.80-1.24)	1.01 (0.81-1.25)	1.26 (0.62-2.57)	0.98 (0.74-1.29)	0.88 (0.48-1.61)
Ondansetron vs Dolasetron+Steroid	3.38 (0.58-19.83)	NA	NA	NA	NA
Ondansetron vs Palonosetron	1.02 (0.24-4.38)	1.02 (0.24-4.38)	NA	1.02 (0.24-4.38)	NA
Ondansetron vs Palonosetron+Steroid	0.39 (0.29-0.54)	0.39 (0.29-0.54)	0.48 (0.26-0.87)	0.33 (0.19-0.58)	0.28 (0.12-0.66)
Ondansetron vs Tropisetron	1.21 (0.66-2.22)	1.94 (0.95-3.95)	NA	1.84 (0.73-4.65)	1.89 (0.59-6.05)
Ondansetron vs Tropisetron+Steroid	0.42 (0.21-0.83)	NA	NA	NA	NA
Ondansetron vs Ramosetron	0.83 (0.51-1.34)	0.67 (0.40-1.12)	NA	0.15 (0.04-0.61)	0.84 (0.47-1.50)
Ondansetron vs Ramosetron+Steroid	0.45 (0.26-0.79)	0.31 (0.16-0.59)	NA	0.08 (0.02-0.31)	NA
Ondansetron vs Placebo	168.11 (9.39-3,008.45)	170.07 (9.50-3,043.64)	NA	165.04 (9.17-2,968.80)	NA
Granisetron vs Dolasetron+Steroid	3.40 (0.59-19.72)	NA	NA	NA	NA
Granisetron vs Palonosetron	1.03 (0.24-4.47)	1.02 (0.23-4.42)	NA	1.05 (0.24-4.60)	NA
Granisetron vs Palonosetron+Steroid	0.39 (0.31-0.51)	0.39 (0.30-0.51)	0.38 (0.24-0.59)	0.34 (0.21-0.55)	0.32 (0.17-0.58)
Granisetron vs Tropisetron	1.22 (0.66-2.25)	1.92 (0.94-3.92)	NA	1.89 (0.75-4.77)	2.15 (0.69-6.74)
Granisetron vs Tropisetron+Steroid	0.42 (0.22-0.81)	NA	NA	NA	NA
Granisetron vs Ramosetron	0.83 (0.49-1.39)	0.66 (0.38-1.15)	NA	0.15 (0.04-0.61)	0.96 (0.42-2.20)
Granisetron vs Ramosetron+Steroid	0.45 (0.25-0.81)	0.31 (0.16-0.59)	NA	0.08 (0.02-0.31)	NA
Granisetron vs Placebo	168.85 (9.51-2,996.63)	168.85 (9.51-2,996.63)	NA	168.84 (9.51-2,996.55)	NA
Dolasetron+Steroid vs Palonosetron	0.30 (0.03-2.99)	NA	NA	NA	NA
Dolasetron+Steroid vs Palonosetron+Steroid	0.12 (0.02-0.67)	NA	NA	NA	NA
Dolasetron+Steroid vs Tropisetron	0.36 (0.06-2.30)	NA	NA	NA	NA

NO. OF PATIENTS WITHOUT CINV	ALL STUDY DESIGNS	ALL RCTs	LOW ROB RANDOMIZATION	CDDP YES (RCTs SGA)	CDDP NO (RCTs SGA)
**Treatment Comparison**	**OR (95% CI)**	**OR (95% CI)**	**OR (95% CI)**	**OR (95% CI)**	**OR (95% CI)**
Dolasetron+Steroid vs Tropisetron+Steroid	0.12 (0.02-0.79)	NA	NA	NA	NA
Dolasetron+Steroid vs Ramosetron	0.24 (0.04-1.52)	NA	NA	NA	NA
Dolasetron+Steroid vs Ramosetron+Steroid	0.13 (0.02-0.84)	NA	NA	NA	NA
Dolasetron+Steroid vs Placebo	49.68 (1.71-1,446.36)	NA	NA	NA	NA
Palonosetron vs Palonosetron+Steroid	0.38 (0.09-1.69)	0.38 (0.09-1.70)	NA	0.32 (0.07-1.53)	NA
Palonosetron vs Tropisetron	1.18 (0.24-5.71)	1.89 (0.37-9.54)	NA	1.80 (0.32-10.07)	NA
Palonosetron vs Tropisetron+Steroid	0.41 (0.08-2.03)	NA	NA	NA	NA
Palonosetron vs Ramosetron	0.81 (0.17-3.73)	0.65 (0.14-3.04)	NA	0.15 (0.02-1.10)	NA
Palonosetron vs Ramosetron+Steroid	0.44 (0.09-2.08)	0.30 (0.06-1.47)	NA	0.08 (0.01-0.57)	NA
Palonosetron vs Placebo	164.01 (6.49-4,145.05)	165.93 (6.57-4,193.53)	NA	161.02 (6.34-4,088.18)	NA
Palonosetron+Steroid vs Tropisetron	3.08 (1.60-5.93)	4.92 (2.32-10.44)	NA	5.57 (1.95-15.88)	6.74 (1.85-24.56)
Palonosetron+Steroid vs Tropisetron+Steroid	1.06 (0.56-2.03)	NA	NA	NA	NA
Palonosetron+Steroid vs Ramosetron	2.10 (1.20-3.68)	1.70 (0.95-3.04)	NA	0.45 (0.11-1.83)	3.00 (1.07-8.44)
Palonosetron+Steroid vs Ramosetron+Steroid	1.15 (0.62-2.12)	0.79 (0.40-1.56)	NA	0.25 (0.07-0.92)	NA
Palonosetron+Steroid vs Placebo	427.99 (23.84-7,682.44)	431.92 (24.06-7,753.23)	NA	498.61 (26.97-9,219.77)	NA
Tropisetron vs Tropisetron+Steroid	0.35 (0.14-0.84)	NA	NA	NA	NA
Tropisetron vs Ramosetron	0.68 (0.34-1.36)	0.34 (0.14-0.83)	NA	0.08 (0.02-0.43)	0.45 (0.12-1.63)
Tropisetron vs Ramosetron+Steroid	0.37 (0.19-0.73)	0.16 (0.06-0.41)	NA	0.04 (0.01-0.22)	NA
Tropisetron vs Placebo	138.80 (7.33-2,627.86)	87.76 (4.53-1,699.05)	NA	89.51 (4.36-1,837.70)	NA
Tropisetron+Steroid vs Ramosetron	1.98 (0.87-4.51)	NA	NA	NA	NA
Tropisetron+Steroid vs Ramosetron+Steroid	1.08 (0.45-2.55)	NA	NA	NA	NA
Tropisetron+Steroid vs Placebo	402.21 (21.03-7,692.43)	NA	NA	NA	NA
Ramosetron vs Ramosetron+Steroid	0.54 (0.35-0.84)	0.46 (0.29-0.73)	NA	0.55 (0.34-0.89)	NA
Ramosetron vs Placebo	203.34 (10.94-3,779.59)	254.48 (13.61-4,756.49)	NA	1,107.86 (45.51-26,971.34)	NA
Ramosetron+Steroid vs Placebo	373.77 (19.86-7,033.29)	550.04 (28.76-10,520.55)	NA	2,007.38 (85.54-47,106.68)	NA
Between-study variance in NMA	0.00	0.00	0.00	0.00	0.01
Design-by-treatment interaction model for inconsistency (χ^2, d.f., P-value, Between-study variance)	19.33 (8, 0.0132, 0.00)	12.89 (6, 0.0449, 0.00)	0.09 (1, 0.7615, 0.03)	0.05 (1, 0.8248, 0.00)	3.07(2,0.2157,0.05)

Abbreviations: CDDP - cisplatin; CI - confidence interval; CINV - chemotherapy-induced nausea and vomiting; NA - not applicable; NMA – network meta-analysis; OR - odds ratio; RCT - randomized controlled trial; ROB – risk of bias; SGA – sub-group analysis

Appendix U. No. of patients experiencing severe vomiting – RCTs only

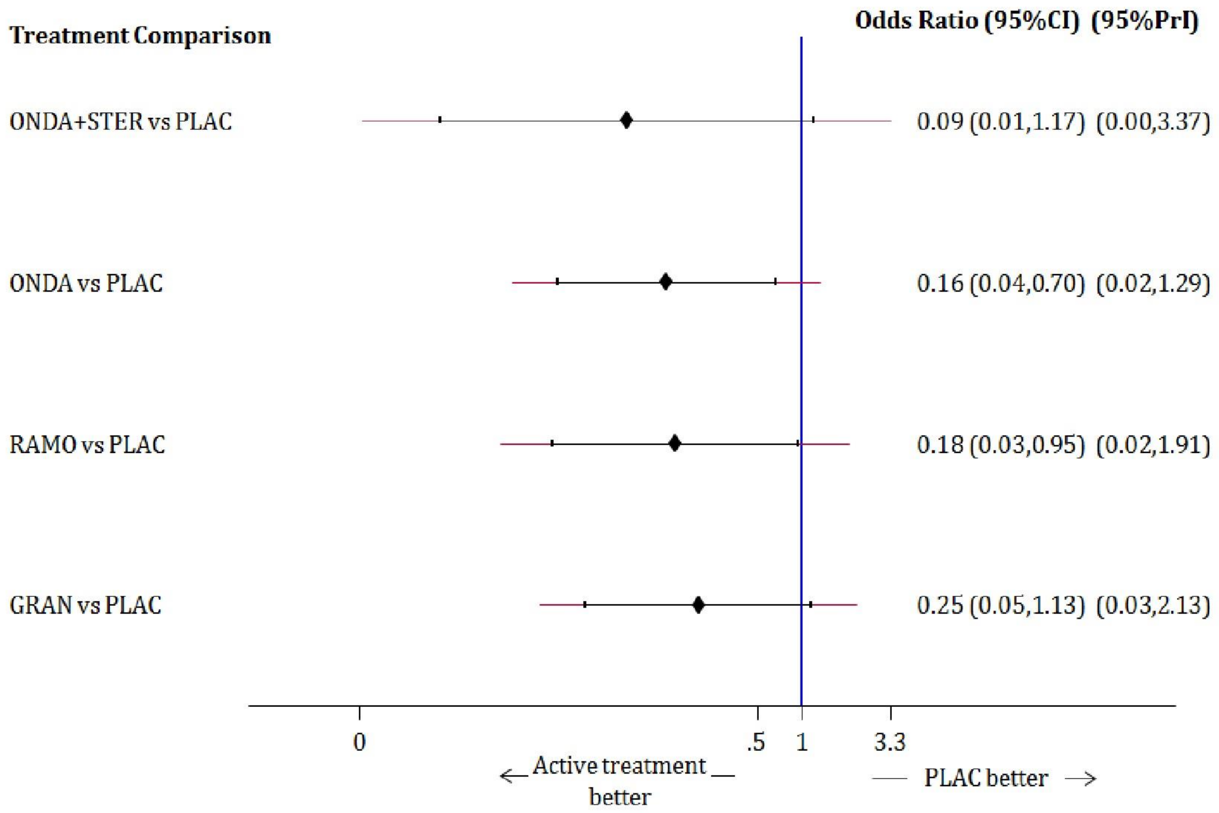

Abbreviations: CI - confidence interval; OR - odds ratio; PrI - prediction interval; RCT - randomized controlled trial

GRAN - granisetron; ONDA - ondansetron; PLAC - placebo; RAMO - ramosetron; STER - steroid

Appendix V. No. of patients experiencing severe vomiting – RCTs only – SUCRA values for each treatment

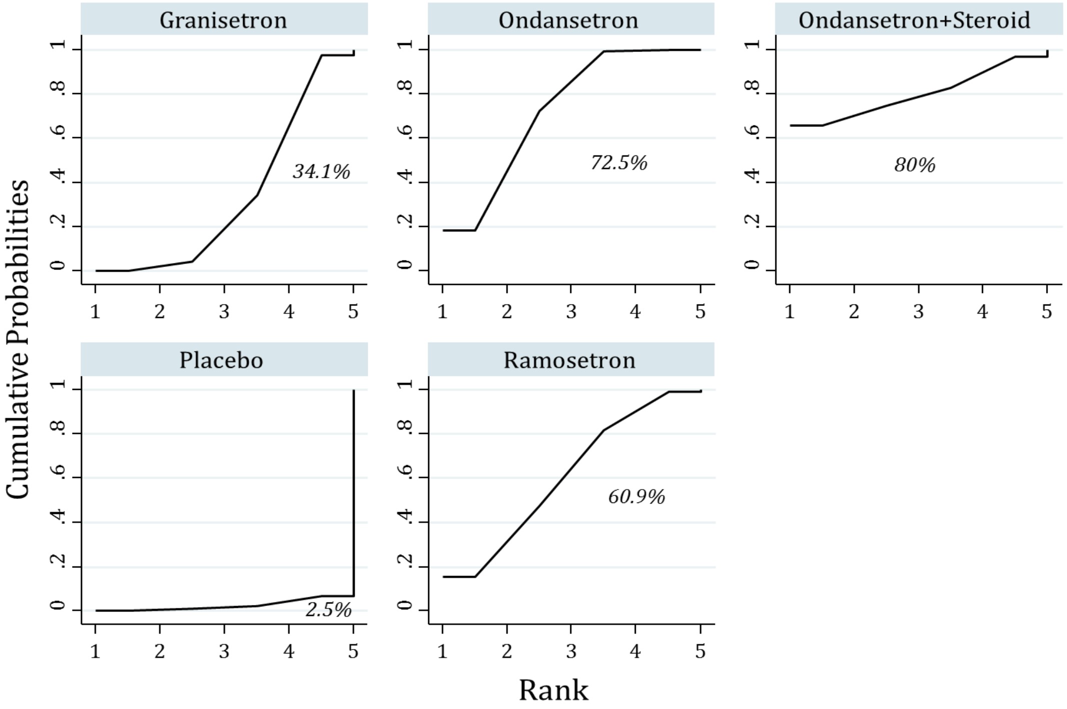

Abbreviations: RCT - randomized controlled trial; SUCRA – surface under the cumulative ranking curve

GRAN- granisetron; ONDA - ondansetron; PLAC - placebo; RAMO - ramosetron; STER - steroid

Appendix W. No. of patients experiencing severe vomiting sub-group analysis for Network meta-analysis

NO. OF PATENTS EXPERIENCING SEVERE VOMITING	ALL STUDY DESIGNS	All RCTs	LOW ROB RANDOMIZATION	CDDP YES (RCTs SGA)	CDDP NO (RCTs SGA)
**Treatment Comparison**	**OR (95% CI)**	**OR (95% CI)**	**OR (95% CI)**	**OR (95% CI)**	**OR (95% CI)**
Ondansetron+Steroid vs Granisetron+Steroid	1.14 (0.18-7.14)	NA	NA	NA	NA
Ondansetron+Steroid vs Ondansetron	0.58 (0.07-4.55)	0.58 (0.07-4.56)	NA	0.58 (0.07-4.98)	NA
Ondansetron+Steroid vs Granisetron	0.38 (0.05-3.03)	0.38 (0.05-3.06)	NA	0.27 (0.02-2.91)	NA
Ondansetron+Steroid vs Tropisetron	0.25 (0.01-6.67)	NA	NA	NA	NA
Ondansetron+Steroid vs Tropisetron+Steroid	2.56 (0.26-25.00)	NA	NA	NA	NA
Ondansetron+Steroid vs Ramosetron	0.52 (0.06-4.76)	0.52 (0.06-4.68)	NA	0.43 (0.04-4.71)	NA
Ondansetron+Steroid vs Ramosetron+Steroid	1.11 (0.04-33.33)	NA	NA	NA	NA
Ondansetron+Steroid vs Placebo	0.09 (0.01-1.18)	0.09 (0.01-1.17)	NA	0.08 (0.00-1.36)	NA
Granisetron+Steroid vs Ondansetron	0.51 (0.03-8.33)	NA	NA	NA	NA
Granisetron+Steroid vs Granisetron	0.33 (0.02-5.26)	NA	NA	NA	NA
Granisetron+Steroid vs Tropisetron	0.22 (0.01-10.00)	NA	NA	NA	NA
Granisetron+Steroid vs Tropisetron+Steroid	2.27 (0.20-25.00)	NA	NA	NA	NA
Granisetron+Steroid vs Ramosetron	0.46 (0.03-7.69)	NA	NA	NA	NA
Granisetron+Steroid vs Ramosetron+Steroid	0.97 (0.06-16.67)	NA	NA	NA	NA
Granisetron+Steroid vs Placebo	0.08 (0.00-1.87)	NA	NA	NA	NA
Ondansetron vs Granisetron	0.65 (0.45-0.94)	0.65 (0.45-0.94)	0.70 (0.45-1.10)	0.46 (0.16-1.32)	0.65 (0.24-1.74)
Ondansetron vs Tropisetron	0.43 (0.03-5.56)	NA	NA	NA	NA
Ondansetron vs Tropisetron+Steroid	4.35 (0.20-100.00)	NA	NA	NA	NA
Ondansetron vs Ramosetron	0.89 (0.41-1.92)	0.89 (0.41-1.94)	0.82 (0.29-2.30)	0.73 (0.24-2.17)	NA
Ondansetron vs Ramosetron+Steroid	1.89 (0.04-100.00)	NA		NA	NA
Ondansetron vs Placebo	0.16 (0.04-0.70)	0.16 (0.04-0.70)	0.14 (0.01-1.40)	0.13 (0.02-0.88)	0.22 (0.02-2.67)
Granisetron vs Tropisetron	0.67 (0.05-8.33)	NA	NA	NA	NA
Granisetron vs Tropisetron+Steroid	6.67 (0.31-100.00)	NA	NA	NA	NA
Granisetron vs Ramosetron	1.37 (0.65-2.94)	1.37 (0.64-2.92)	1.17 (0.46-2.97)	1.60 (0.59-4.30)	NA
Granisetron vs Ramosetron+Steroid	2.94 (0.06-100.00)	NA	NA	NA	NA
Granisetron vs Placebo	0.25 (0.05-1.13)	0.25 (0.05-1.13)	0.20 (0.02-2.08)	0.29 (0.03-2.56)	0.34 (0.02-4.94)
Tropisetron vs Tropisetron+Steroid	10.00 (0.19-562.45)	NA	NA	NA	NA
Tropisetron vs Ramosetron	2.04 (0.18-25.00)	NA	NA	NA	NA
Tropisetron vs Ramosetron+Steroid	4.35 (0.04-478.83)	NA	NA	NA	NA
Tropisetron vs Placebo	0.37 (0.02-7.12)	NA	NA	NA	NA
Tropisetron+Steroid vs Ramosetron	0.20 (0.01-4.76)	NA	NA	NA	NA
Tropisetron+Steroid vs Ramosetron+Steroid	0.43 (0.01-16.67)	NA	NA	NA	NA
Tropisetron+Steroid vs Placebo	0.04 (0.00-1.10)	NA	NA	NA	NA
Ramosetron vs Ramosetron+Steroid	2.13 (0.04-100.00)	NA	NA	NA	NA
Ramosetron vs Placebo	0.18 (0.03-0.95)	0.18 (0.03-0.95)	0.17 (0.01-2.13)	0.18 (0.02-1.62)	NA
Ramosetron+Steroid vs Placebo	0.08 (0.00-5.64)	NA	NA	NA	NA
Ramosetron + Steroid vs Granisetron + Steroid	NA	0.00 (0.00-0.00)	NA	NA	NA
Between-study variance in NMA	0.00	0.00	0.00	0.10	0.00

Design-by-treatment interaction model for inconsistency (χ^2, d.f., P-value, Between-study variance)	0.34 (1, 0.5627, 0.00)	0.33 (1, 0.5628, 0.00)	NA - No closed loops	1.57 (1, 0.2099, 0.00)	NA - No closed loops

Abbreviations: CDDP - cisplatin; CI - confidence interval; NA – not applicable; NMA - network meta-analysis; OR - odds ratio; RCT - randomized controlled trial; ROB - risk of bias, SGA - sub-group analysis

Appendix X. Included studies in our review versus previous systematic reviews

Study	Jordan, 2007	Jantunen, 1996	Billio, 2010 (Cochrane)	Philips, 2010 (Cochrane)	# of patients
Kovacs, 2016	NO	NO	NO	NO	331
Anvari, 2015	NO	NO	NO	NO	117
Gabrail, 2015(a)	NO	NO	NO	NO	45
Gabrail, 2015(b)	NO	NO	NO	NO	35
Karthaus, 2015	NO	NO	NO	NO	739
Kim K, 2015	NO	NO	NO	NO	32
Kim JE, 2015	NO	NO	NO	NO	261
Kimura, 2015	NO	NO	NO	NO	24
Ohzawa, 2015	NO	NO	NO	NO	40
Raftopolous, 2015	NO	NO	NO	NO	1395
Zoto, 2015	NO	NO	NO	NO	155
Kim JS, 2014	NO	NO	NO	NO	180
Murakami, 2014	NO	NO	NO	NO	46
Sanmukhani, 2014	NO	NO	NO	NO	214
Boccia, 2013	NO	NO	NO	NO	639
Huang, 2013	NO	NO	NO	NO	236
Keat, 2013	NO	NO	NO	NO	52
Wenzell, 2013	NO	NO	NO	NO	40
Blazer, 2012	NO	NO	NO	NO	305
Endo, 2012	NO	NO	NO	NO	105
Ithimakin, 2012	NO	NO	NO	NO	162
Noor, 2012	NO	NO	NO	NO	30
Roscoe, 2012	NO	NO	NO	NO	944
Tsuji, 2012	NO	NO	NO	NO	359
Boccia, 2011	NO	NO	NO	NO	621
Dong, 2011	NO	NO	NO	NO	89
Giralt, 2011	NO	NO	NO	NO	73
Pielichowski, 2011	NO	NO	NO	NO	60
Piyush, 2011*	NO	NO	NO	NO	90
Siddique, 2011	NO	NO	NO	NO	60
Tian, 2011	NO	NO	NO	NO	144
Ghosh, 2010	NO	NO	NO	NO	1213
Ho, 2010	NO	NO	NO	NO	285
Kaushal, 2010	NO	NO	NO	NO	30
Mattiuzzi, 2010	NO	NO	NO	NO	143
Keyhanian, 2009	NO	NO	NO	NO	138
Maemondo, 2009	NO	NO	NO	NO	231
Rzepecki, 2009	NO	NO	NO	NO	46
Saito, 2009	NO	NO	YES	NO	1114
Segawa, 2009	NO	NO	NO	NO	204
Taguchi, 2009	NO	NO	NO	NO	59
Yonemura, 2009	NO	NO	NO	NO	179
Yu, 2009	NO	NO	NO	NO	208
Chen, 2008	NO	NO	NO	NO	424
Fabi, 2008	NO	NO	NO	NO	89
Nagel, 2008	NO	NO	NO	NO	25
Sepúlveda-Vildósola, 2008	NO	NO	NO	NO	100
Abali, 2007	NO	NO	NO	NO	158
Berrak, 2007	NO	NO	NO	YES	18
Carreca, 2007*	NO	NO	NO	NO	67
Hamadani, 2007	NO	NO	NO	NO	126
Herrstedt, 2007	NO	NO	NO	NO	82
Kadota, 2007*	NO	NO	NO	NO	60
Pectasides, 2007	NO	NO	NO	NO	134
Shi, 2007	NO	NO	NO	NO	50
Aapro, 2006	YES	NO	YES	NO	667
Adel, 2006 *	NO	NO	NO	NO	176
Pinarli, 2006	NO	NO	NO	NO	38

Shikimori, 2006	NO	NO	NO	NO	63
Tabei, 2006*	NO	NO	NO	NO	204
Trifilio, 2006*	NO	NO	NO	NO	69
Babaoglu, 2005	NO	NO	NO	NO	216
Buyukavci, 2005	NO	NO	NO	NO	22
Cheirsilpa, 2005	NO	NO	NO	NO	73
Corapcioglu, 2005	NO	NO	NO	YES	22
Kurnianda, 2005	NO	NO	NO	NO	39
Lindley, 2005	NO	NO	NO	NO	232
Mandanas, 2005	NO	NO	NO	NO	197
Yano, 2005	NO	NO	NO	NO	39
Dempsey, 2004	YES	NO	NO	NO	224
Eisenberg, 2004	NO	NO	NO	NO	161
Jaing, 2004	NO	NO	NO	YES	66
Kim, 2004	NO	NO	NO	NO	105
Tan, 2004	YES	NO	NO	NO	26
Tantipalakorn, 2004	NO	NO	NO	NO	109
Villalon, 2004	NO	NO	NO	NO	283
Aapro, 2003	NO	NO	NO	NO	267
Bhatia, 2003	NO	NO	NO	NO	80
Carmichael, 2003	NO	NO	NO	NO	41
Eisenberg, 2003	YES	NO	NO	NO	569
Gralla, 2003	YES	NO	NO	NO	563
Koizumi, 2003	NO	NO	NO	NO	30
Matsuoka, 2003	NO	NO	NO	NO	50
Sagae, 2003	NO	NO	NO	NO	138
Steiner, 2003	NO	NO	NO	NO	62
Feng, 2002a	NO	NO	NO	NO	73
Feng, 2002b	NO	NO	NO	NO	111
Kang, 2002	NO	NO	YES	NO	194
Noda, 2002	NO	NO	YES	NO	136
Noda, 2002	NO	NO	NO	NO	84
Raynov, 2002	NO	NO	NO	NO	101
Yamac, 2002	NO	NO	NO	NO	97
Aksoylar, 2001	YES	NO	NO	NO	51
de Wit, 2001	YES	NO	NO	NO	40
Fox-Geiman, 2001	NO	NO	NO	NO	102
Koralewski, 2001	NO	NO	NO	NO	76
Liaw, 2001	NO	NO	NO	NO	721
Ozgur Tan, 2001	NO	NO	NO	NO	25
Parker, 2001	NO	NO	NO	YES	26
Tsavaris, 2001a	YES	NO	NO	NO	106
Tsavaris, 2001b	NO	NO	NO	NO	100
Watters, 2001	NO	NO	NO	NO	274
Zeng, 2001	NO	NO	NO	NO	69
Abang, 2000	NO	NO	NO	NO	60
Barrajon, 2000	YES	NO	NO	NO	120
Chiou, 2000	YES	NO	NO	NO	51
Chua, 2000	YES	NO	NO	NO	89
Fauser , 2000	NO	NO	NO	NO	210
Forni, 2000	YES	NO	NO	NO	90
Friedman, 2000	NO	NO	NO	NO	230
Herrington, 2000	YES	NO	NO	NO	61
Hesketh, 2000	NO	NO	NO	NO	97
Koenig, 2000	NO	NO	NO	NO	40
Oge, 2000	YES	NO	NO	NO	106
Raynov, 2000	NO	NO	NO	NO	72
Roila [The Italian Group for Antiemetic Research], 2000	NO	NO	NO	NO	708
Slaby, 2000	NO	NO	NO	NO	45
Stewart, 2000	NO	NO	NO	NO	16
White, 2000	NO	NO	NO	YES	428
Coppes, 1999a	NO	NO	NO	NO	46
Coppes, 1999b	NO	NO	NO	NO	32
Davidson, 1999	NO	NO	NO	NO	427

Kandemir, 1999	NO	NO	NO	NO	89
Komada, 1999	NO	NO	NO	YES	36
Lazarus, 1999	NO	NO	NO	NO	43
Needles, 1999	NO	NO	NO	NO	357
Orchard, 1999	NO	NO	NO	YES	187
Raynov, 1999	NO	NO	NO	NO	35
Sandoval, 1999	NO	NO	NO	YES	31
Sigsgaard, 1999	NO	NO	NO	NO	223
Stiakaki, 1999	NO	NO	NO	NO	23
Tatsumi, 1999	NO	NO	NO	NO	186
The Italian Multicenter Study Group, 1999	NO	NO	NO	NO	532
Tsavaris, 1999	NO	NO	NO	NO	76
Tsuchida, 1999	NO	NO	NO	YES	44
Uchida, 1999	NO	NO	NO	NO	29
Yalcn, 1999	YES	NO	NO	NO	54
Birch, 1998	NO	NO	NO	NO	70
Brown, 1998	NO	NO	NO	NO	49
Gralla, 1998	YES	NO	YES	NO	1054
Handberg, 1998	NO	NO	NO	NO	90
Kalaycio, 1998	NO	NO	NO	NO	45
Kleisbauer, 1998	NO	NO	NO	NO	308
Kris, 1998	NO	NO	NO	NO	44
Krzakowski, 1998	YES	NO	NO	NO	530
Latreille, 1998	NO	NO	NO	NO	435
Martoni, 1998	YES	NO	NO	NO	198
Perez, 1998	YES	NO	NO	NO	1085
Perez, 1998	NO	NO	NO	NO	573
Poon, 1998	NO	NO	NO	NO	20
Ritter, 1998	NO	NO	NO	NO	393
Scoponi, 1998	NO	NO	NO	NO	134
Sorbe, 1998	NO	NO	NO	NO	282
Spector, 1998	YES	NO	YES	NO	371
Tsavaris, 1998	NO	NO	NO	NO	104
Zeidman, 1998	NO	NO	NO	NO	58
Beck, 1997	NO	NO	NO	NO	402
Chang, 1997	NO	NO	NO	NO	51
Chevallier, 1997	NO	NO	NO	NO	226
Davidson, 1997	NO	NO	NO	NO	406
du Bois, 1997	NO	NO	NO	NO	189
Farley, 1997	NO	NO	NO	NO	119
Fumoleau, 1997	NO	NO	NO	NO	420
Gridelli, 1997	NO	NO	NO	NO	230
Grote, 1997	NO	NO	NO	NO	307
Harris, 1997	NO	NO	NO	NO	608
Kasimis, 1997	NO	NO	NO	NO	30
Kigawa, 1997	NO	NO	NO	NO	85
Kris, 1997	NO	NO	NO	NO	75
Lofters, 1997	YES	NO	NO	NO	696
Mustacchi, 1997	NO	NO	NO	NO	105
Park, 1997	YES	NO	YES	NO	97
Pectasides, 1997	YES	NO	NO	NO	80
Perez, 1997	YES	NO	NO	NO	353
Roila [The Italian Group for Antiemetic Research], 1997	NO	NO	NO	NO	318
Rubenstein, 1997	NO	NO	NO	NO	320
Advani, 1996	NO	NO	NO	NO	91
Audhuy, 1996	YES	NO	YES	NO	474
Barrenetxea, 1996	NO	NO	NO	NO	NR
Belkacemi, 1996	NO	NO	NO	NO	36
Brock, 1996	NO	NO	NO	YES	187
Crucitt, 1996	NO	NO	NO	NO	133
de Wit, 1996	NO	NO	NO	NO	20
Fauser, 1996a	NO	NO	NO	NO	309
Fauser, 1996b	YES	NO	NO	NO	399

Fujimoto, 1996	NO	NO	NO	NO	40
Gaedicke, 1996	NO	NO	NO	NO	49
Hesketh, 1996a	NO	NO	NO	NO	69
Hesketh, 1996b	YES	NO	YES	NO	609
Jorgensen, 1996	NO	NO	NO	NO	100
Leonardi, 1996	NO	NO	NO	NO	118
Martoni, 1996	YES	NO	YES	NO	124
Massida & Ionta, 1996	YES	NO	NO	NO	60
Matsui, 1996	NO	NO	NO	NO	70
Olver, 1996	NO	NO	NO	NO	604
Peterson, 1996	NO	NO	NO	NO	104
Yeilding, 1996	NO	NO	NO	NO	62
Adams, 1995	NO	YES	NO	NO	126
Agura, 1995a	NO	NO	NO	NO	30
Agura, 1995b	NO	NO	NO	NO	30
Alfieri, 1995	NO	NO	NO	NO	28
Alvarez, 1995	NO	NO	NO	YES	34
Bleiberg, 1995	NO	NO	NO	NO	930
Bonneterre, 1995	YES	NO	NO	NO	150
Chiou, 1995	NO	YES	NO	NO	40
Contu, 1995	NO	NO	NO	NO	120
DiBenedetto, 1995	NO	NO	NO	NO	81
Dick, 1995	NO	NO	NO	YES	30
Fedele, 1995	NO	NO	NO	NO	25
Gebbia, 1995	NO	NO	NO	NO	262
Hahlen, 1995	NO	NO	NO	YES	88
Hesketh, 1995	NO	NO	NO	NO	146
Italian Group for Antiemetic Research, 1995	NO	NO	YES	NO	973
Labar, 1995	NO	NO	NO	NO	52
Latreille, 1995	NO	YES	NO	NO	292
Mantovani, 1995	YES	NO	YES	NO	117
Marty, 1995	YES	NO	YES	NO	231
Navari, 1995a	NO	YES	NO	NO	536
Navari, 1995b	YES	NO	YES	NO	987
Stewart, 1995	YES	NO	NO	NO	488
The Italian Group for Antiemetic Research, 1995	NO	YES	NO	NO	408
Tsavaris, 1995a	NO	NO	NO	NO	80
Tsavaris, 1995b	NO	YES	NO	NO	84
Ahn, 1994	NO	NO	NO	NO	49
Carmichael, 1994	NO	YES	NO	NO	278
Chevallier, 1994	NO	NO	NO	NO	101
Chiu, 1994	NO	NO	NO	NO	56
Conroy, 1994	NO	NO	NO	NO	164
Cubeddu, 1994	NO	YES	NO	NO	324
Gebbia, 1994a	YES	NO	YES	NO	166
Gebbia, 1994b	NO	NO	NO	NO	158
Heron, 1994	NO	YES	NO	NO	357
Hesketh, 1994	NO	YES	NO	NO	275
Hulstaert, 1994	NO	NO	NO	NO	445
Joss, 1994	NO	YES	NO	NO	207
Kaizer, 1994	NO	NO	NO	NO	295
Kris, 1994	NO	NO	NO	NO	89
Lim, 1994	NO	NO	NO	NO	55
Merrouche, 1994	NO	NO	NO	NO	25
Navari, 1994	YES	NO	NO	NO	184
Noble, 1994	YES	NO	NO	NO	359
Riviere, 1994	NO	NO	NO	NO	157
Ruff, 1994	YES	NO	YES	NO	496
Sorbe, 1994	NO	NO	NO	NO	63
Suarez, 1994	NO	NO	NO	NO	44
Tsavaris, 1994	NO	NO	NO	NO	90
Tyson, 1994	NO	NO	NO	NO	44

Van Belle, 1994a	NO	NO	NO	NO	143
Van Belle, 1994b	NO	NO	NO	NO	74
Beck, 1993	NO	YES	NO	NO	349
Bosi, 1993	NO	NO	NO	NO	40
Buser, 1993	NO	NO	NO	NO	82
Chevallier, 1993	NO	NO	NO	NO	281
Cunningham, 1993	NO	NO	NO	NO	32
de Wet, 1993	NO	NO	NO	NO	91
Grunberg, 1993	NO	NO	NO	NO	125
Herrstedt, 1993	NO	NO	NO	NO	30
Hunter, 1993	NO	NO	NO	NO	284
Jantunen, 1993	YES	NO	NO	NO	166
Levitt, 1993	NO	YES	NO	NO	164
Madej, 1993	NO	NO	NO	NO	115
Pisters, 1993	NO	NO	NO	NO	22
Rath, 1993	NO	YES	NO	NO	113
Ribiero, 1993	NO	NO	NO	NO	145
Smith (Granisetron Study Group), 1993	NO	NO	NO	NO	504
Beck, 1992	YES	NO	NO	NO	699
Blijham, 1992	NO	NO	NO	NO	909
Bots, 1992	NO	NO	NO	NO	125
Dicato, 1992	NO	NO	NO	NO	324
Dundee, 1992	NO	NO	NO	NO	233
Jantunen, 1992	YES	NO	NO	NO	47
Seynaeve, 1992	YES	NO	NO	NO	535
Sledge, 1992	NO	YES	NO	NO	46
Soukop, 1992	NO	YES	NO	NO	187
Fraschini, 1991	NO	NO	NO	NO	80
Gandara, 1991	NO	NO	NO	NO	274
Hainsworth, 1991	NO	YES	NO	NO	274
Jones, 1991	NO	YES	NO	NO	112
Lemerle, 1991	NO	NO	NO	NO	24
Marschner, 1991	NO	YES	NO	NO	122
Roila, 1991	NO	YES	NO	NO	102
Seynaeve, 1991	NO	NO	NO	NO	35
Smith, 1991	NO	YES	NO	NO	31
Smyth, 1991	NO	YES	NO	NO	100
Warr, 1991	NO	NO	NO	NO	152
Addelman, 1990	NO	NO	NO	NO	24
Bonneterre, 1990	NO	YES	NO	NO	75
Cubeddu, 1990a	NO	NO	NO	NO	20
Cubeddu, 1990b	NO	YES	NO	NO	28
Cupissol, 1990	NO	YES	NO	NO	28
De Mulder, 1990	NO	YES	NO	NO	121
Falkson, 1990	NO	NO	NO	NO	56
Kaasa, 1990	NO	YES	NO	NO	93
Khojasteh, 1990	NO	NO	NO	NO	36
Marty, 1990a	NO	NO	NO	NO	228
Marty, 1990b	NO	YES	NO	NO	97
Cunningham, 1989	NO	NO	NO	NO	16
Grunberg, 1989	NO	NO	NO	NO	43
De Hann, 1988	NO	NO	NO	NO	13
**Total number of patients not included in other review**	44, 840	53, 443	50, 602	57, 215	

***** Includes unpublished data (Adel 2006, Tabei 2006, Trifilio 2006, Carreca 2007, Kadota 2007, Piyush 2011)

Appendix Y. List of excluded studies from previous systematic reviews

Study	Jordan, 2007	Jantunen, 1996	Bilio, 2010 (Cochrane)	Philips, 2010 (Cochrane)	Reason for exclusion
Anderson et al, 1994	NO	YES	NO	NO	No Comparator
Andrews et al, 1992	NO	YES	NO	NO	No relevant population
Andrews et al, 1993	NO	YES	NO	NO	No relevant population
Barrajon et al, 2000	YES	NO	NO	NO	CA >10 years old
Basade et al, 1996	NO	NO	NO	YES	No 5-HT3
Bosnjak et al, 2006	YES	NO	NO	NO	Not a relevant study design
Bremer, 1992	NO	YES	NO	NO	No Comparator
Bubalo et al, 2001	YES	NO	NO	NO	No comparator
Chan et al, 1987	NO	NO	NO	YES	No 5-HT3
Chang et al, 1996	NO	YES	NO	NO	No Comparator
Cooper et al, 1992	NO	YES	NO	NO	Not a relevant study design
Cubeddu et al, 1992	NO	YES	NO	NO	No 5-HT3
Cubeddu et al, 1993	NO	YES	NO	NO	Not a relevant study design
Cunningham et al, 1996	NO	YES	NO	NO	No Comparator
Dalzell et al, 1986	NO	NO	NO	YES	No 5-HT3
De Wit et al, 1996	NO	YES	NO	NO	Not a relevant study design
del Giglio et al, 2000	YES	NO	NO	NO	Not a relevant study design
Doherty, 1999)	YES	NO	NO	NO	Not a relevant study design
du Bois, 1998	YES	NO	NO	NO	Not a relevant study design
Ekert et al, 1979	NO	NO	NO	YES	No 5-HT3
Fetting et al, 1982	NO	YES	NO	NO	No 5-HT3
Freeman et al, 1992	NO	YES	NO	NO	No relevant population
French Ondansetron Study Group, 1995	NO	YES	NO	NO	No Comparator
Graham-Pole et al, 1986	NO	NO	NO	YES	No 5-HT3
Gralla et al, 1981	NO	YES	NO	NO	No 5-HT3
Hirota et al, 1993	NO	NO	NO	YES	NE
Hue at al, 1998	YES	NO	NO	NO	NE
Italian Group for Antiemetic Research in Radiotherapy, 1999	YES	NO	NO	NO	No relevant population
Jantunen et al, 1993	NO	YES	NO	NO	No 5-HT3
Jones et al, 1992	NO	YES	NO	NO	Not a relevant study design
Jordan et al, 2005	YES	NO	NO	NO	Not a relevant study design
Kaiser et al, 2002	YES	NO	NO	NO	No Comparator
Kamanabrou, 1992	YES	NO	NO	NO	Not a relevant study design
Kris et al, 1987	NO	YES	NO	NO	No 5-HT3
None, 2000	YES	NO	NO	NO	Not a relevant study design
None, 2000	YES	NO	NO	NO	Not a relevant study design
Laszlo, 1983	YES	NO	NO	NO	Not a relevant study design
Mabro et al, 2000	NO	NO	NO	YES	NE
Mabro et al, 1999	YES	NO	NO	NO	NE
Mantel et al, 1959	NO	YES	NO	NO	No relevant population
Marshall et al,	NO	NO	NO	YES	No 5-HT3

1989					
Marty, 1992	NO	YES	NO	NO	No Comparator
Medler et al, 1994	NO	YES	NO	NO	CA >10 years old
Mehta et al, 1997	NO	NO	NO	YES	No Comparator
Mehta et al, 1986	NO	NO	NO	YES	No 5-HT3
Mendarte et al, 2000	YES	NO	NO	NO	Not a relevant study design
Miner et al, 1986	NO	YES	NO	NO	No relevant population
Monde et al , 1994	YES	NO	NO	NO	CA >10 years old
Morrow et al, 1995	NO	YES	NO	NO	Not a relevant study design
Multinational Association for Supportive Care in Cancer, 2005	YES	NO	NO	NO	Not a relevant study design
Nakamura et al, 1999	YES	NO	NO	NO	CA >10 years old
Navari et al, 1993	NO	YES	NO	NO	No Comparator
Newberry et al, 1993	YES	NO	NO	NO	No relevant population
Nicolai et al, 1993	NO	YES	NO	NO	No Comparator
Noguera et al, 2001	NO	NO	NO	YES	NE
Osoba et al, 1996	YES	NO	NO	NO	No 5-HT3
Pinkerton et al, 1993	YES	NO	NO	NO	CA >10 years old
Pollera et al, 1989	NO	YES	NO	NO	No 5-HT3
Roila et al, 1988	NO	YES	NO	NO	No 5-HT3
Safonova et al, 1999	NO	NO	NO	YES	NE
Sismondi et al, 1994	NO	YES	NO	NO	CA >10 years old
Sorbe et al, 1994	NO	YES	NO	NO	No Comparator
Sorbe et al, 1994	NO	YES	NO	NO	No Comparator
Suarez et al, 1994	NO	NO	NO	YES	CA >10 years old
Swann et al, 1979	NO	NO	NO	YES	No 5-HT3
Tejedor et al, 1999	NO	NO	NO	YES	Not a relevant study design
The Italian Group for Antiemetic Research, 1993	NO	YES	NO	NO	No Comparator
Tonato et al, 1991	NO	YES	NO	NO	Not a relevant study design
Warr et al, 1992	NO	YES	NO	NO	No Comparator
Whitehead, 2002	YES	NO	NO	NO	Not a relevant study design
Wilder-Smith et al, 1993	NO	YES	NO	NO	Not a relevant study design
Yusuf et al, 1985	YES	NO	NO	NO	No relevant population
Zhang et al, 1996	YES	NO	NO	NO	NE

Abbreviations: CA - conference abstract; NE - non-English; NR - not reported
