# Supplementary material for: Comparative safety and effectiveness of serotonin receptor antagonists in patients undergoing chemotherapy: a systematic review and network meta-analysis
Source: BMC Med. 2016 Dec 23;14:216. doi: 10.1186/s12916-016-0761-9 (PMC5180412; doi:10.1186/s12916-016-0761-9)
Supplement: Additional file 3: — Appendices A–Y (PDF 1123 kb) [file 12916_2016_761_MOESM3_ESM.pdf]

Appendix A. Network plots for patients without nausea

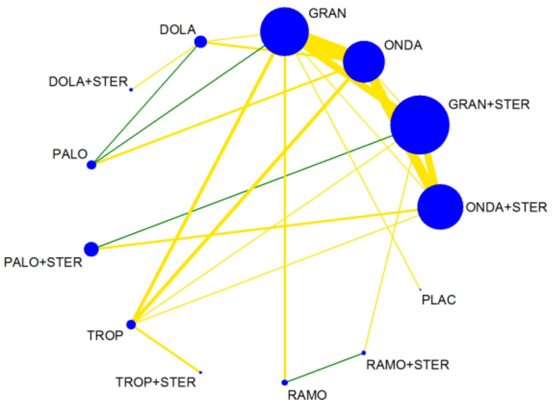

Low ROB for randomization

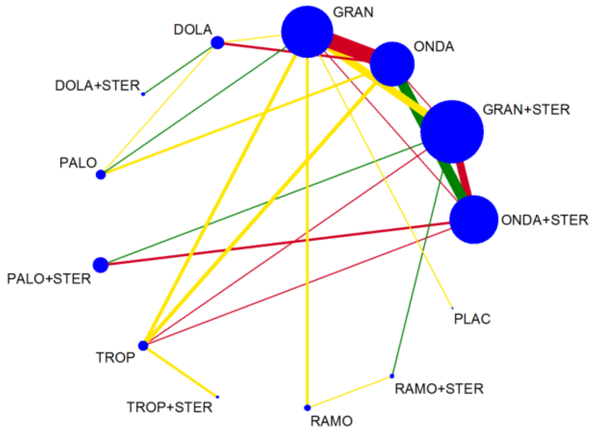

Low ROB for allocation concealment

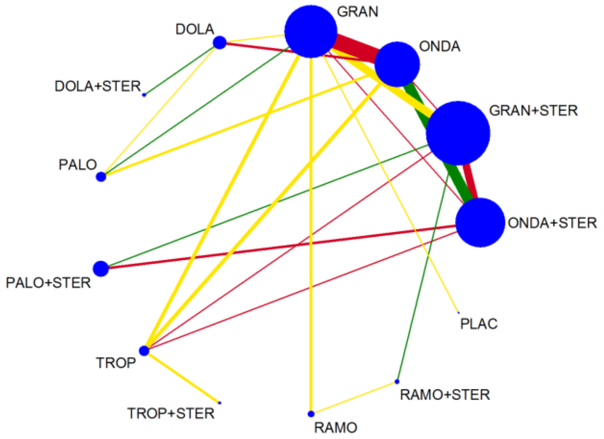

Low ROB for blinding

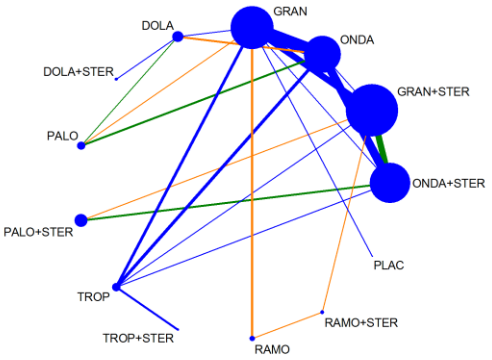

Continent (on average)*

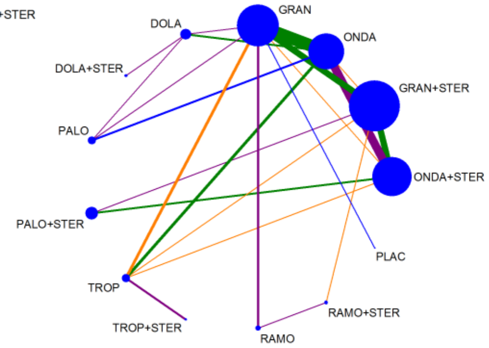

Setting (on average)†

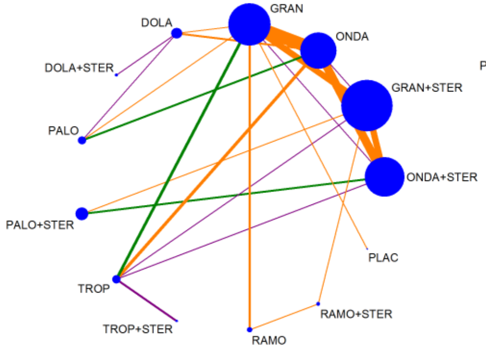

Age (on average)‡

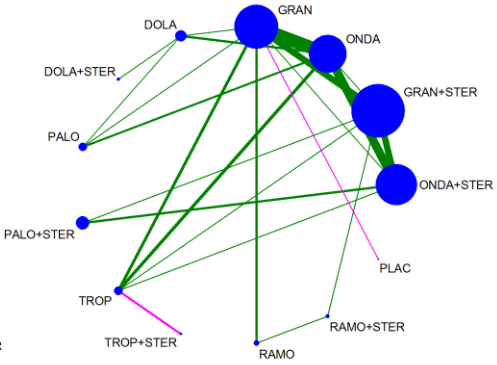

Diagnosis category (on average)§

Abbreviations: ROB - risk of bias
* Blue - Europe; Orange - Asia; Purple - Multi-continent; Green - North America; Red - Not reported
† Blue - Hospital; Orange - Medical Centre; Purple - Multi-Centre; Green - Not reported
‡ Blue - Adults & Elderly (18-99); Orange - Adults (18-65); Purple - All groups; Green - Children (<=18)
§ Brown - Acute Lymphocytic Leukemia; Blue - Acute Lymphocytic Leukemia; Purple – Breast; Red – Lung; Green - Multiple; Magenta - Not reported; Orange - Ovarian

Treatment Abbreviations: DOLA - dolasetron; DOLA+STER - dolasetron+steroid; GRAN - granisetron; GRAN+STER - granisetron+steroid; ONDA - ondansetron; ONDA+STER - ondansetron+steroid; PLAC - placebo; PALO - palonosetron; PALO+STER- palonosetron+steroid; RAMO - ramosetron; RAMO+STER - ramosetron+steroid; TROP - tropisetron; TROP+STER - tropisetron+steroid

Appendix B. Network plots for patients without vomiting

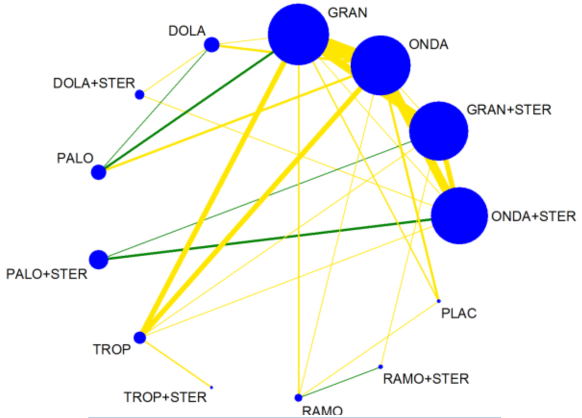

Low ROB for randomization

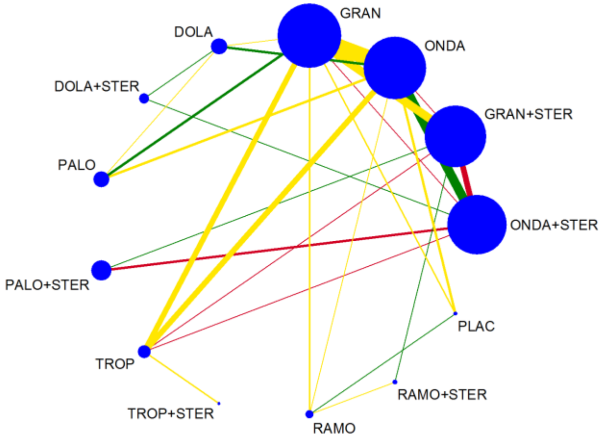

Low ROB for allocation concealment

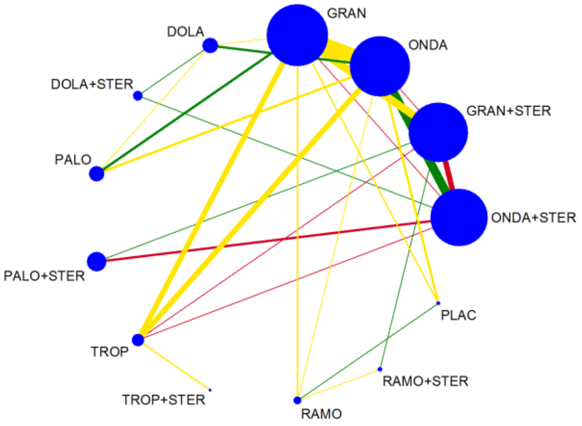

Low ROB for blinding

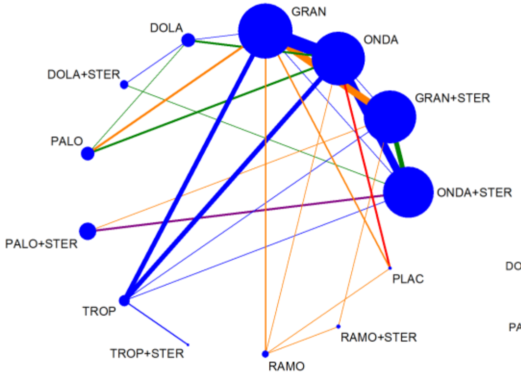

Continent (on average)*

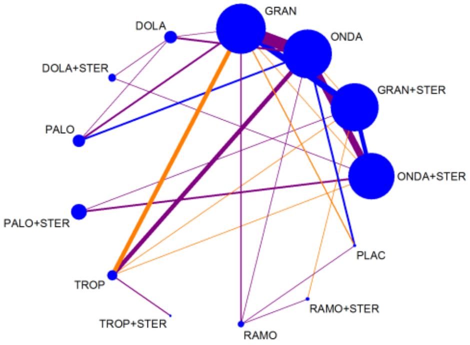

Setting (on average)†

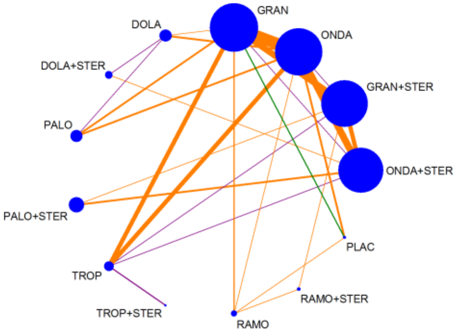

Age (on average)‡

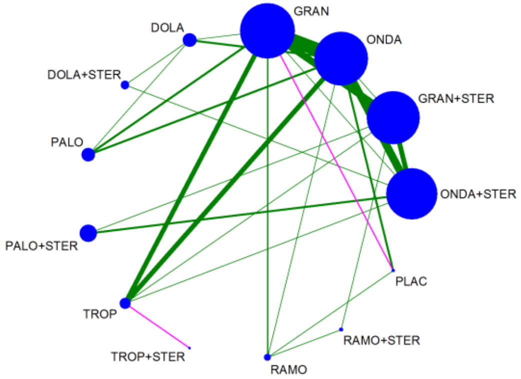

Diagnosis category (on average)§

Abbreviations: ROB - risk of bias

* Blue - Europe; Orange - Asia; Purple - Multi-continent; Green - North America; Red - South America; Magenta - Not reported

† Blue - Hospital; Orange - Medical Centre; Purple - Multi-Centre; Green - Not reported; Red - Single-Centre

‡ Blue - Adults & Elderly (18-99); Orange - Adults (18-65); Purple - All groups; Green - Children (<=18); Red - Adults & Children (0 to <65); Magenta - All groups

§ Brown - Acute Lymphocytic Leukemia; Blue - Acute Myelogenous Leukemia; Purple - Breast; Red - Lung; Green - Multiple; Magenta - Not Reported ; Orange - Ovarian

Treatment Abbreviations: DOLA - dolasetron; DOLA+STER - dolasetron+steroid; GRAN - granisetron; GRAN+STER - granisetron+steroid; ONDA - ondansetron; ONDA+STER - ondansetron+steroid; PLAC - placebo; PALO - palonosetron; PALO+STER- palonosetron+steroid; RAMO - ramosetron; RAMO+STER - ramosetron+steroid; TROP - tropisetron; TROP+STER - tropisetron+steroid

Appendix C. Network plots for patients without CINV

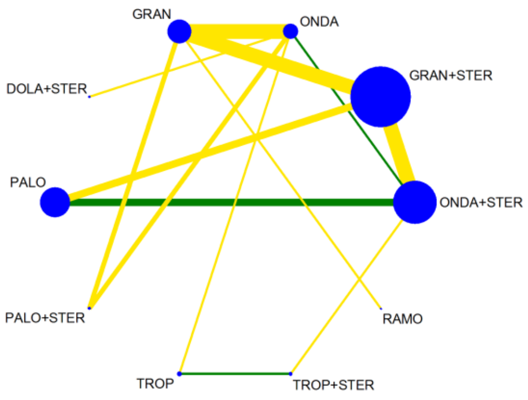

Low ROB for randomization

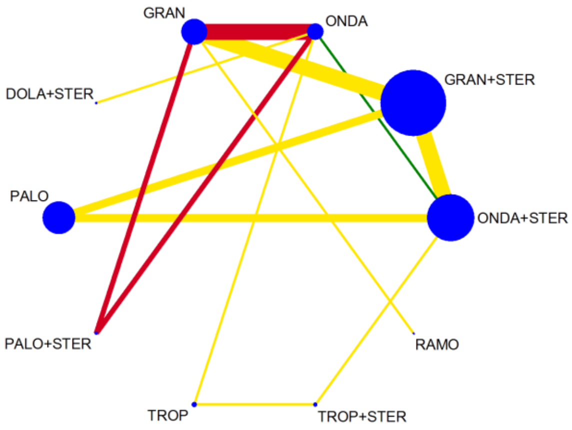

Low ROB for allocation concealment

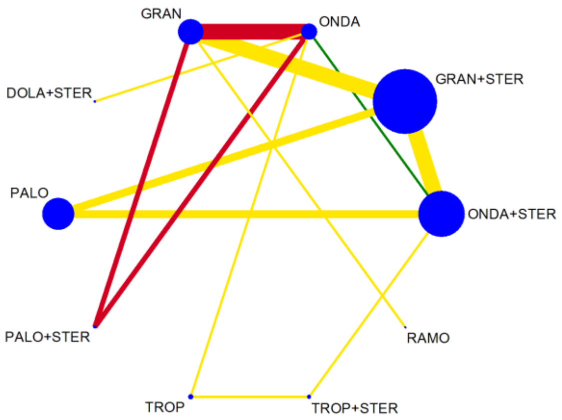

Low ROB for blinding

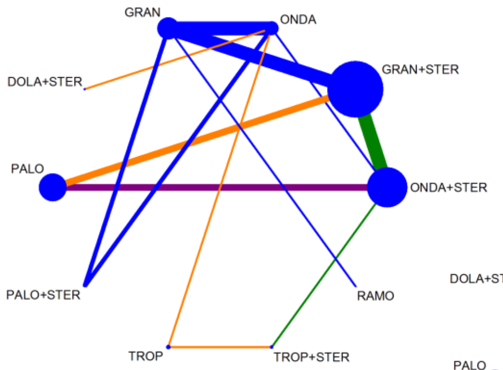

Continent (on average)*

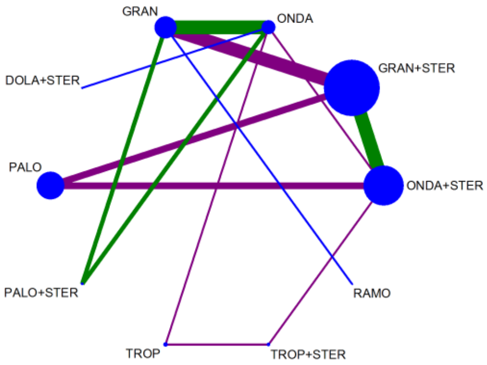

Setting (on average)†

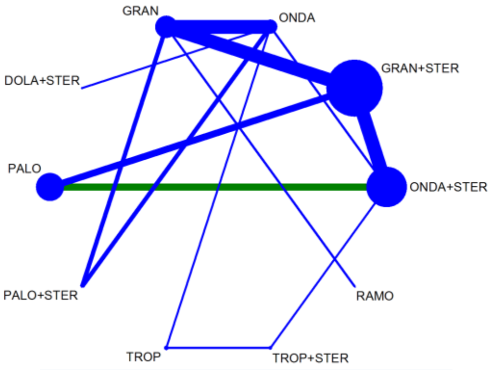

Age (on average)‡

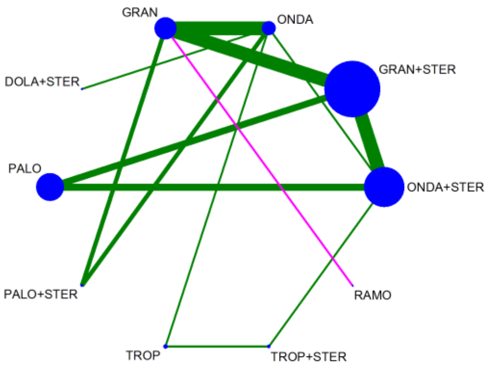

Diagnosis category (on average)§

Abbreviations: CINV – chemotherapy-induced nausea and vomiting; ROB - risk of bias
* Blue - Europe; Orange - Asia; Purple - Multi-continent; Green - North America; Red - Not reported
† Blue - Hospital; Orange - Medical Centre; Purple - Multi-Centre; Green - Not Reported
‡ Blue - Adults & Elderly (18-99); Orange - Adults (18-65); Green - Children (<18)
§ Green - Multiple; Blue - Colorectal Cancer; Magenta - Not reported

Treatment Abbreviations: DOLA+STER - dolasetron+steroid; GRAN - granisetron; GRAN+STER - granisetron+steroid; ONDA - ondansetron; ONDA+STER - ondansetron+steroid; PALO - palonosetron; PALO+STER-palonosetron+steroid; RAMO - ramosetron; TROP - tropisetron; TROP+STER - tropisetron+steroid

Appendix D. Network plots for patients experiencing severe vomiting

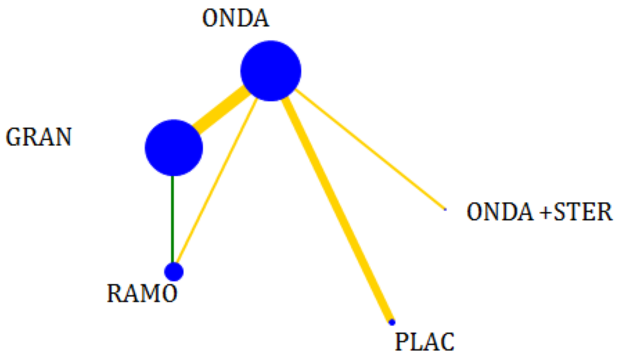

Low ROB for randomization

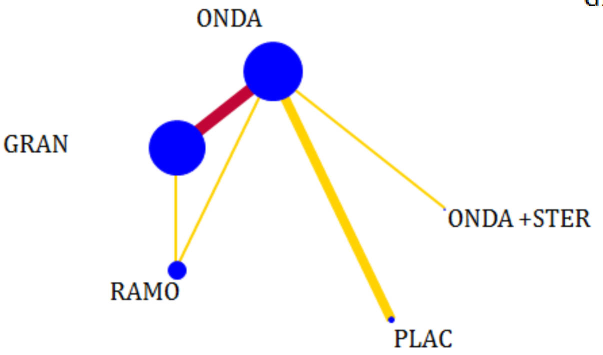

Low ROB for allocation concealment

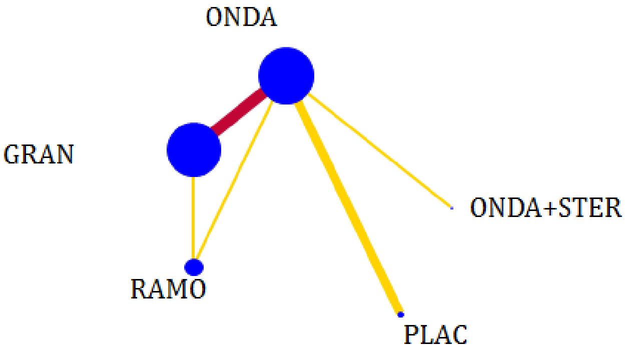

Low ROB for blinding

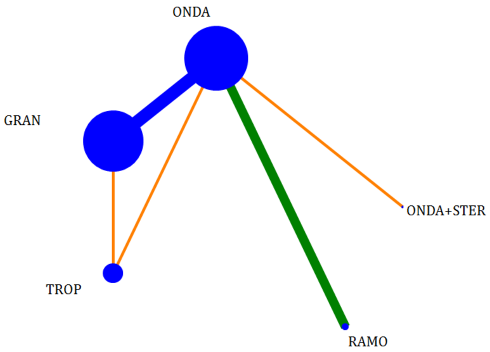

Continent (on average)*

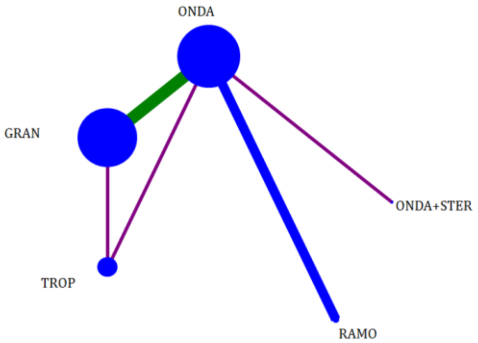

Setting (on average)†

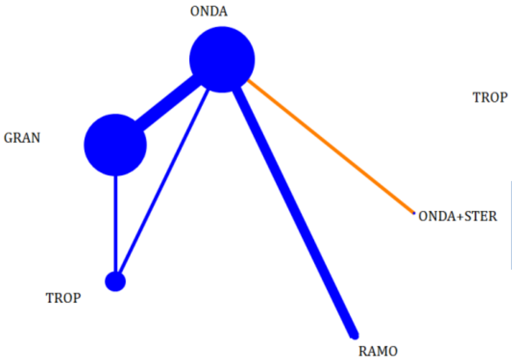

Age (on average)‡

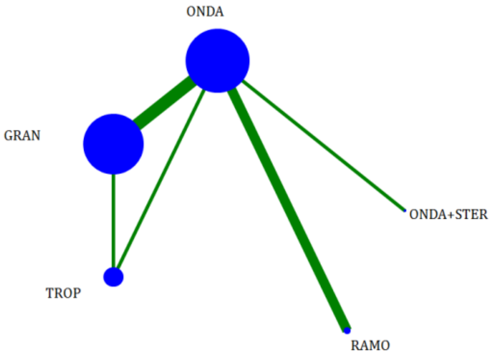

Diagnosis category (on average)§

Abbreviations: ROB - risk of bias
* Blue - Europe; Orange - Asia; Purple - Multi-continent; Green - North America;
† Blue - Hospital; Orange - Medical Centre; Purple - Multi-Centre; Green - Not Reported; Red - Single-Centre
‡ Blue - Adults & Elderly (18-99); Orange - Adults (18-65)
§ Green - Multiple; Magenta - Not reported

Treatment Abbreviations: GRAN - granisetron; ONDA - ondansetron; ONDA+STER - ondansetron+steroid; RAMO - ramosetron; TROP - tropisetron
